# Identification of Bioactive Compounds and Potential Mechanisms of Kuntai Capsule in the Treatment of Polycystic Ovary Syndrome by Integrating Network Pharmacology and Bioinformatics

**DOI:** 10.1155/2022/3145938

**Published:** 2022-04-28

**Authors:** Xiushen Li, Jingxin Ma, Li Guo, Chenle Dong, Guli Zhu, Wenli Hong, Can Chen, Hao Wang, Xueqing Wu

**Affiliations:** ^1^Department of Obstetrics and Gynecology, Shenzhen University General Hospital, Shenzhen, Guangdong, China; ^2^Guangdong Key Laboratory for Biomedical Measurements and Ultrasound Imaging, School of Biomedical Engineering, Shenzhen University Health Science Center, Shenzhen, Guangdong, China; ^3^Shenzhen Key Laboratory, Shenzhen University General Hospital, Shenzhen, Guangdong, China; ^4^School of Biomedical Engineering, Health Science Center, Shenzhen University, Shenzhen, Guangdong, China; ^5^School of Pharmaceutical Sciences, Health Science Center, Shenzhen University, Shenzhen, Guangdong, China; ^6^Clinical Medical Academy, Shenzhen University, Shenzhen, Guangdong, China

## Abstract

**Objective:**

This study elucidates the potential therapeutic targets and molecular mechanisms of KTC in the treatment of PCOS.

**Materials and Methods:**

Using the Traditional Chinese Medicine System Pharmacology Database and Analysis Platform (TCMSP), the active ingredients and potential targets of KTC were obtained. The Gene Expression Omnibus (GEO) database was used to find differentially expressed genes (DEGs) related to PCOS. Search the CTD, DisGeNet, genecards, NCBI, OMIM, and PharmGKB databases for therapeutic targets related to PCOS. The intersection of potential targets, DEGs, and therapeutic targets was submitted to perform bioinformatics analysis by R language. Finally, the analyses' core targets and their corresponding active ingredients were molecularly docked.

**Results:**

88 potential therapeutic targets of KTC for PCOS were discovered by intersecting the potential targets, DEGs, and therapeutic targets. According to bioinformatics analysis, the mechanisms of KTC treatment for PCOS could be linked to IL-17 signaling route, p53 signaling pathway, HIF-1 signaling pathway, etc. The minimal binding energies of the 5 core targets and their corresponding ingredients were all less than -6.5. Further research found that quercetin may replace KTC in the treatment of PCOS. *Discussion and Conclusions*. We explored the active ingredients and molecular mechanisms of KTC in the treatment of PCOS and found that quercetin may be the core ingredient of KTC in the treatment of PCOS.

## 1. Introduction

Polycystic ovary syndrome (PCOS) is one of the most common endocrine and metabolic diseases in gynecology. The main symptom of PCOS is excessive androgen, which also affects ovarian function and causes infertility [1]. At present, the cause of PCOS is still unclear, but recent studies have shown that the predisposing factors of PCOS may be related to the patients' daily life style and psychological factors [2]. Oral contraceptives, antiandrogens, and other hormonal interventional drugs are the clinically recognized therapy options [3, 4]. The efficacy and safety drugs for PCOS, on the other hand, have yet to be discovered.

In recent years, the curative effect of many classic prescriptions of traditional Chinese medicine (TCM) in the treatment of PCOS has been recognized by more and more people [5]. According to Chinese medicine theory, one of the core pathogenic processes of PCOS is kidney shortage and blood stasis [6]. Kuntai Capsule (KTC) nourishes the kidneys and also improves blood circulation, which helps to regulate estrogen levels and promote ovarian function (Zhang H et al. [7]). The mechanisms of KTC in the treatment of PCOS may be related to increasing the patient's sensitivity to insulin, inhibiting oocyte apoptosis, and improving impaired ovarian function, according to the literature (Zhang J et al. [8]; Zhang B et al. [9, 10]). However, the specific mechanisms of KTC in the therapy of PCOS remains unknown.

**Figure 1:**
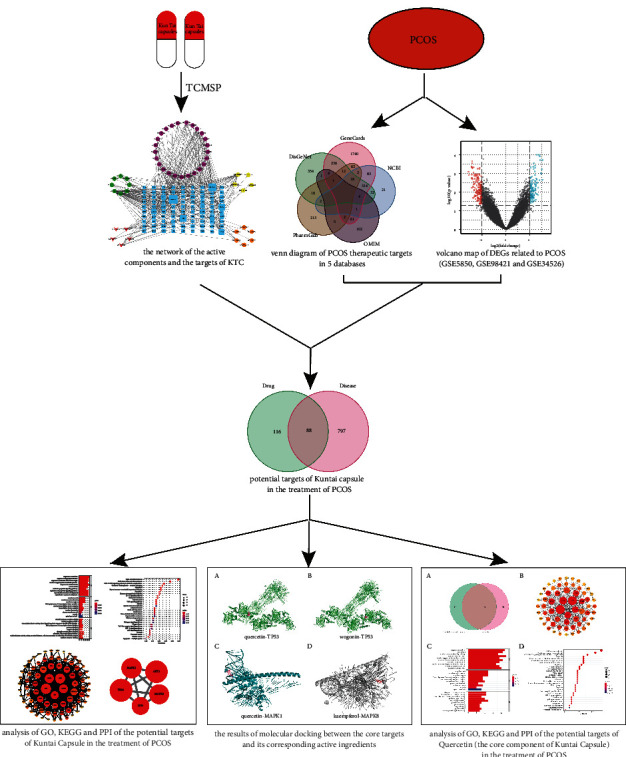
Flow chart.

As one of the cutting-edge methods to explore the mechanisms of drug therapy, network pharmacology has achieved remarkable results in exploring the therapeutic mechanisms of TCM prescriptions and screening the active ingredients and therapeutic targets of TCM ([11]; Gao X et al. [12]). Therefore, we used network pharmacology, bioinformatics, molecular docking, and other methods to reveal the active ingredients, targets, and molecular mechanisms of KTC in the treatment of PCOS. The flow chart of the entire study is shown in Figure 1.

## 2. Materials and Methods

### 2.1. Screen the Active Ingredients and Targets of KTC

We searched the active ingredients of KTC through the TCMSP database (https://old.tcmsp-e.com/tcmsp.php) based on the conditions of drug-like properties ≥ 0.18 and bioavailability ≥ 30% [13]. Then, we searched for the targets of the active ingredients through the TCMSP database and converted the target names to the gene names through the uniprot database (https://www.uniprot.org/). Finally, the Cytoscape 3.7.2 software was used to construct the relationship network between the active ingredients and target genes of KTC.

### 2.2. Collect the Therapeutic Targets of PCOS

The therapeutic targets were attained by searching DisGeNet, genecards, NCBI, OMIM, and PharmGKB with “PCOS” and “polycystic ovary syndrome” as keywords. Then, we converted the target names to the gene names by the uniprot database.

### 2.3. Screen Targets Related to PCOS

We merged the three PCOS-related datasets (GSE5850, GSE98421, GSE34526) found in the Gene Expression Omnibus (GEO) database and further used the R language “sva” and “limma” packages for batch correction and differentially expressed genes (DEGs) screening |log2 (foldchange)| > 1 and *p* value < 0.05).

### 2.4. Potential Therapeutic Targets of KTC in the Treatment of PCOS

Therapeutic targets obtained from CTD, DisGeNet, genecards, NCBI, OMIM, and PharmGKB databases were combined with DEGs from the GEO database and screened for targets appearing in at least two databases. These targets were then intersected with KTC therapeutic targets to identify prospective KTC therapeutic targets for PCOS.

### 2.5. The Analysis of PPInetwork, GO, and KEGG

We obtained the interactions between potential therapeutic targets of KTC through the STRING database. The protein-protein interaction (PPI) network was constructed by the Cytoscape software, and the core therapeutic targets were further screened according to the degree value. To investigate the probable molecular mechanisms of KTC in the treatment of PCOS, R language was used to perform Gene Ontology (GO) and Kyoto Encyclopedia of Genes and Genomes (KEGG) enrichment analysis.

### 2.6. Molecular Docking

Firstly, identify the active ingredients that correlate to KTC's core targets in the treatment of PCOS. The active ingredient's two-dimensional structure was retrieved via the PubChem website and translated into the three-dimensional structure with the lowest free energy by using the ChemBio3D software. Then, the 3D structure of the core target was obtained through the PDB database, and the water molecules and small molecule ligands were deleted through the “PyMOL” software. Next, the “AutoDockTools” software was used to convert the protein and drug ingredient into PDBQT format files and identify active pockets. Finally, we used the “vina” software for molecular docking.

### 2.7. Identify the Core Ingredients of KTC

We intersected the therapeutic targets of all active ingredients in KTC with the therapeutic targets of KTC. The active ingredient with the most overlapping targets was considered to be the core ingredient of KTC. Further bioinformatics analysis of the potential therapeutic targets of the core ingredients was performed.

## 3. Results

### 3.1. The Active Ingredients and Targets of KTC

**Figure 2:**
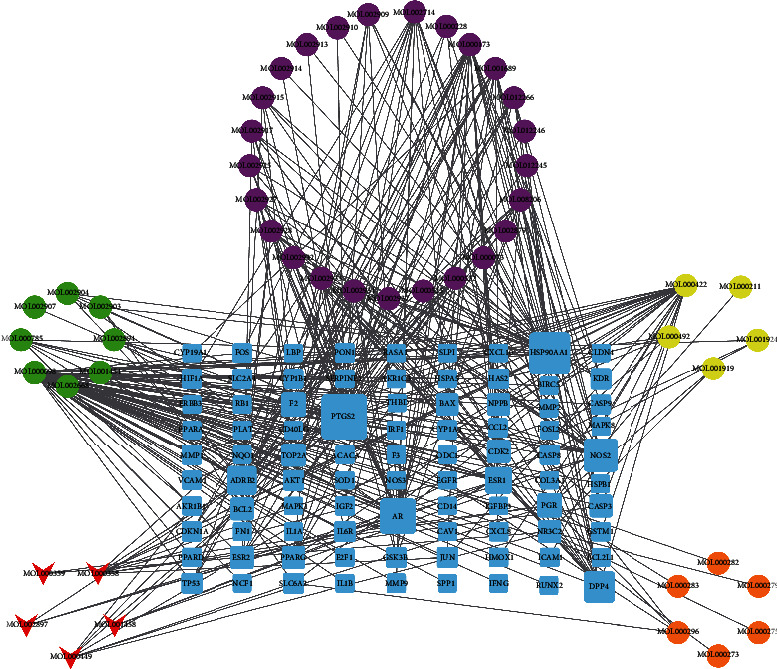
The network of the relationship between the active ingredients and the targets of KTC.

According to the screening conditions, 80 active ingredients and 204 therapeutic targets of KTC were obtained through the TCMSP database (Supplementary Table 1 and Supplementary Table 2). After converting target names to gene names, the KTC regulatory network was constructed though the “Cytoscape” software. As shown in Figure 2, the surrounding circles were the active ingredients of KTC, and different colors represented different drugs. The red triangles and blue rectangles represented the active ingredients shared by various TCM and therapeutic targets, respectively. The degree value represented the number of edges connected to the node in the graph. The top three pharmaceutical ingredients in terms of degree value were quercetin, kaempferol, and wogonin.

### 3.2. Therapeutic Targets for PCOS

We found 988, 2540, 477, 181, and 327 therapeutic targets in the DisGeNet, genecards (relevance score 1), NCBI, OMIM, and PharmGKB databases, respectively, by using keywords “polycystic ovary syndrome” and “PCOS” (supplement table 3).

### 3.3. Targets Related to PCOS

**Figure 3:**
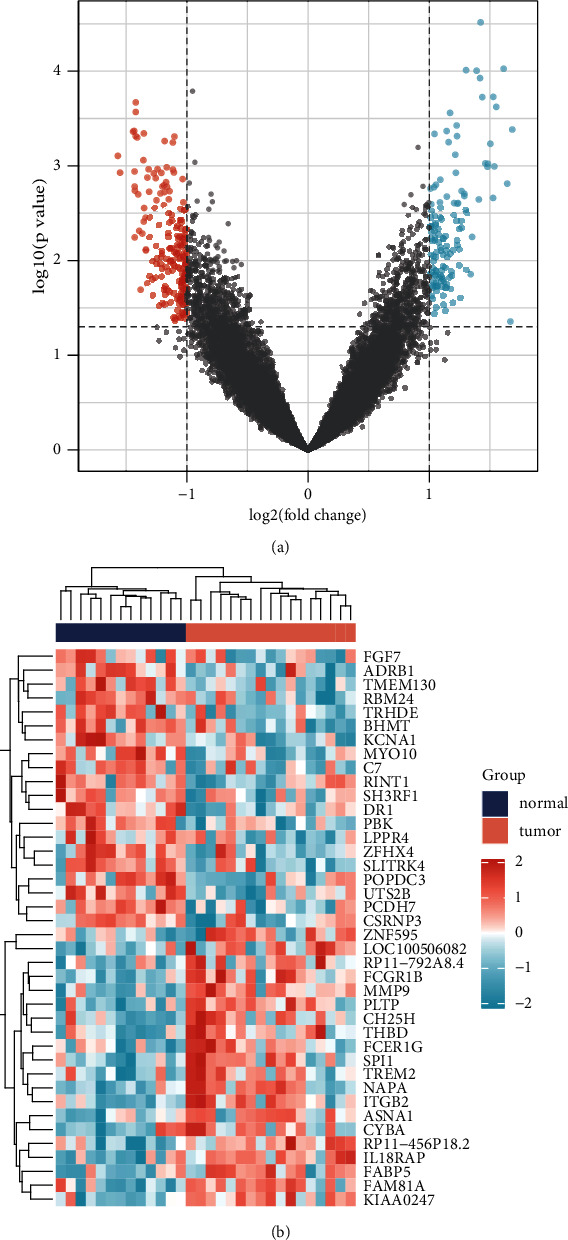
DEGs related to PCOS in the GEO dataset. (a) Volcano map of DEGs related to PCOS (GSE5850, GSE98421, and GSE34526). (b) Heat map of DEGs related to PCOS (GSE5850, GSE98421, and GSE34526).

We utilized the R language "limma" package to detect 315 DEGs after excluding batch effects in three data datasets linked to PCOS (supplement table 3). The red dots on the left represented genes with low expression in PCOS patients, whereas the blue dots on the right represented genes with high expression in PCOS patients (Figure 3(a)).  Figure 3(b) shows the expression of the top 20 DEGs ranked high and low in PCOS patients versus healthy individuals.

### 3.4. Potential Therapeutic Targets of KTC

**Figure 4:**
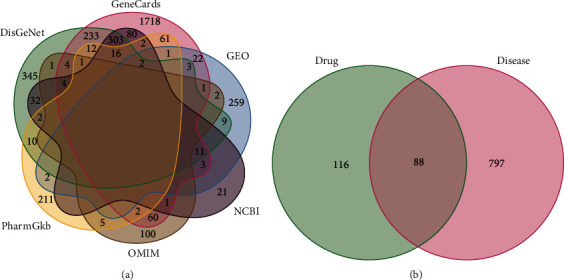
Targets related to PCOS treatment. (a) The Venn diagram of PCOS therapeutic targets in 5 disease databases and GEO data sets. (b) The Venn diagram of the targets in at least two databases in (a) and the therapeutic targets of KTC.

The obtained DEGs from the GEO database were combined with PCOS-related targets from the DisGeNet, genecards, NCBI, OMIM, and PharmGKB databases. Targets that appeared at least twice were screened and intersected with therapeutic targets of KTC, resulting in 88 potential therapeutic targets for PCOS (Figures 4(a) and 4(b); Supplementary Table 4).

### 3.5. Analysis Results of GO, KEGG, and PPI Networks

**Figure 5:**
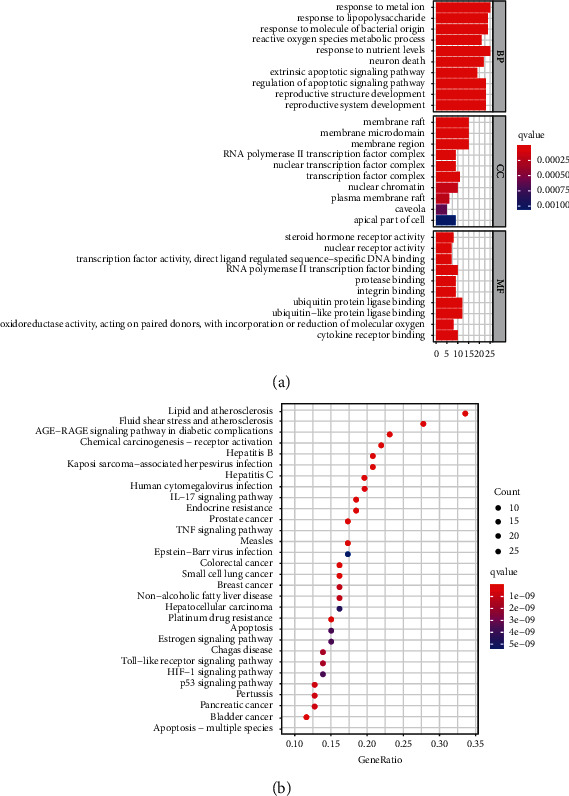
The GO and KEGG enrichment analyses of KTC's therapeutic target. (a) GO enrichment analysis (the top 10 results of BP, CC, MF enrichment analysis respectively). (b) KEGG enrichment analysis of therapeutic targets (the top 30 results).

**Figure 6:**
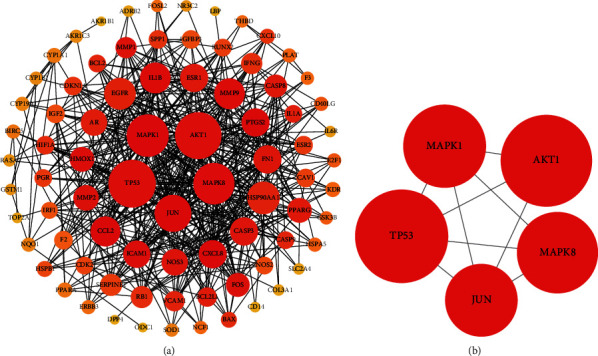
The PPI network of KTC's targets for the treatment of PCOS. (a) Analysis results of PPI network. (b) The core targets of the PPI network.

In order to further explore the mechanisms of KTC in the treatment of PCOS, we performed R language to perform GO and KEGG enrichment analyses of potential therapeutic targets (supplement table 5,1). As shown in Figure 5(a), in terms of biological processes, targets were mostly enriched in reactions with metal ions, lipopolysaccharides, bacteria-derived molecules, nutritional levels, apoptosis, reactive oxygen metabolism, reproductive system, neuronal death, etc. In terms of cell components, targets were mostly enriched in membrane raft, membrane microdomain, membrane region, RNA polymerase II transcription factor complex, nuclear transcription factor complex, and so on. In terms of molecular function, the targets were mostly enriched in the activity of steroid hormone receptors, nuclear receptors, transcription factors, oxidoreductase factors, etc. KEGG enrichment analysis found that the targets were mostly enriched in IL-17 signaling pathway, TNF signaling pathway, p53 signaling pathway, Toll-like receptor signaling pathway, HIF-1 signaling pathway, etc. (Figure 5(b)).  Figure 6(a) illustrates the PPI network of potential therapeutic targets. The darker the color, the larger the node area and the higher the degree and importance (Figure 6(a)). The selected core potential therapeutic targetswere shown in Figure 6(b). The R language scripts used in this study were shown in Supplementary Table 9.

### 3.6. The Results of Molecular Docking

**Figure 7:**
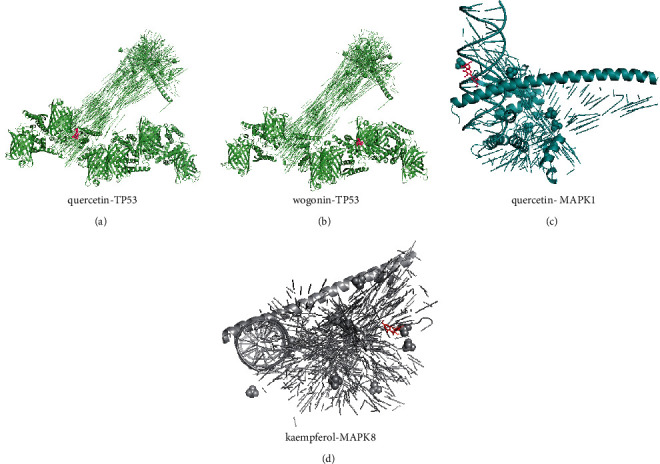
The results of molecular docking between the core targets of the PPI network and their corresponding active ingredients (the four results with the lowest binding energy). (a) Molecular docking results of TP53 and quercetin (binding energy -8.9). (b) Molecular docking results of TP53 and wogonin (binding energy -8.8). (c) Molecular docking results of MAPK1 and quercetin (binding energy -8.7). (d) Molecular docking results of MAPK8 and kaempferol (binding energy -8.7).

By analyzing the PPI network, the five targets with the highest degree of MAPK1, MAPK8, TP53, AKT1, and JUN were identified and further searched for their corresponding active ingredients. Then, following the molecular docking steps described in the methods section, we executed the corresponding operations and acquired the molecular docking data for the targets and their corresponding active ingredients (Supplementary Table 6). We found that the binding energies of all molecular docking results were less than -6.5. The docking results for the four compounds with the lowest binding energies are shown in Figure 7.

### 3.7. The Core Ingredients of KTC

**Figure 8:**
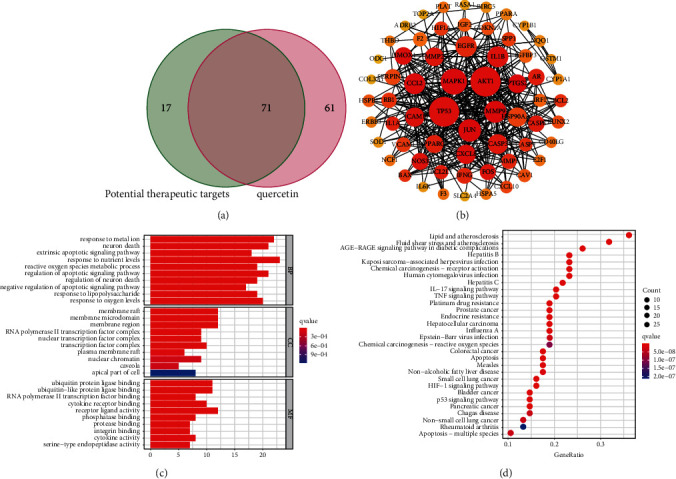
The bioinformatic analysis of quercetin's potential therapeutic target. (a) The Venn diagram of the potential therapeutic targets of quercetin and the therapeutic targets of KTC. (b) PPI network of therapeutic targets. (c) GO enrichment analysis of therapeutic targets (the top 10 results of BP, CC, MF enrichment analysis respectively). (d) KEGG enrichment analysis of therapeutic target (the top 30 results).

By intersecting the targets of each active ingredient with the potential therapeutic targets of KTC (supplement table 7), we finally determined that quercetin was the core ingredient of KTC. Quercetin had 71 targets that overlap with the potential therapeutic targets of KTC for PCOS (Figure 8(a)). We performed PPI network analysis on these 71 targets and found that the 5 core targets were almost the same as those of KTC (Figure 8(b)). As shown in Figure 8(c), the repetition rate of the GO and KEGG enrichment analysis results of quercetin and KTC reached 75%, which were IL-17 signaling pathway, TNF signaling pathway, endocrine resistance, p53 signaling pathway, HIF-1 signaling pathway, apoptosis-multiple species, and so on (Figure 8(d); supplement table 8).

## 4. Discussion

Many TCM formulations have been used for the clinical treatment of PCOS. KTC targets the pathogenesis of PCOS by invigorating the kidney and promoting blood circulation, regulating the level of estrogen, and improving ovarian function. TCM formulations are difficult to examine at the molecular level due to their multi-ingredient and multitarget features. However, the emergence of network pharmacology has made it possible to systematically research TCM formulations. Therefore, this study relies on network pharmacology and bioinformatics to explore the molecular mechanisms of KTC in the treatment of PCOS.

By intersecting the therapeutic targets of KTC and PCOS in the bioinformatics database, 88 potential therapeutic targets of KTC for PCOS were finally obtained. We used potential therapeutic targets to construct a PPI network and further screened out 5 core targets (MAPK1, MAPK8, TP53, AKT1, and JUN). In mammals, the MAPK family participates in a variety of biological processes in the human body. Currently, the 14 MAPK family members that have been identified played important roles in transforming extracellular stimuli into cellular responses [14]. The cascades of MAPK are involved in many steps in the regulation of ovulation, including the recovery of meiosis and the rupture of follicles. MAPK1 plays a significant role in the mechanisms of insulin resistance and ovulation dysfunction in PCOS patients [15]. MAPK8 affects the progress of PCOS by regulating the autophagy of follicular cells [16]. TP53, a transcription factor, stabilizes and induces the transcription of genes related to cell cycle arrest, apoptosis, and metabolism [17]. TP53 participates in the occurrence and progression of PCOS by inducing the apoptosis of ovarian granulosa cells [18]. JUN belongs to the AP-1 transcription factor family, which causes fibrosis and regulates many core cell biological processes [19]. As an important regulator of ovarian function, AKT participates in multiple biological processes including the activation of primordial follicles and the differentiation of granulosa cells [20]. AKT1 is involved in the proliferation of granular cells and follicle formation. The upregulated AKT1 in PCOS patients may be related to granule cell dysfunction [21].

We performed KEGG enrichment analysis on 88 potential therapeutic targets of KTC for PCOS, and found that molecular mechanisms of KTC's treatment of PCOS might be related to IL-17 signaling pathway, TNF signaling pathway, p53 signaling pathway, Toll-like receptor, and so on. IL17A, a proinflammatory cytokine, is mainly secreted by T-helper 17 cells. In PCOS patients, IL17A is abundantly expressed. The activation of the IL17A signaling pathway can result in the release of inflammatory mediators such as TNF, IL-6, and IL-1 ([22]; Gao Q et al. [23]). TNF is a cytokine with a wide range of biological activities, including TNF-*α* and TNF-*β* secreted by macrophages and T lymphocytes, respectively. As an adipokine of systemic inflammation, TNF-*α* is highly expressed in obese PCOS patients ([24]; Zhang Q et al. [25]). TNF-*α* signaling pathway is related to the uptake of glucose in tissues, which may lead to the decline of female fertility [26]. Increased androgen is a common clinical feature of PCOS patients, which can promote the expression of p53 [27]. P53 has previously been linked to cytokines including IL-1, IL-6, and TNF-*α*. The p53 signaling pathway may be involved in ovarian granulosa cell autophagy and death, which could be linked to PCOS pathophysiology [28]. The expression of Toll-like receptors in PCOS patients is significantly increased, which can lead to a decrease in the rate of available embryos in PCOS patients ([29]; Wang Y et al. [30]). Insulin resistance, the significant pathogenic feature of PCOS, is present in almost 85 percent of patients [31]. Recent studies have found that Toll-like receptors activate the NF-*κ*B signaling pathway, leading to insulin resistance in PCOS patients ([32]; Wang D et al. [33]).

By molecular docking of the 5 core targets and their corresponding drug ingredients, we found that wogonin-TP53, kaempferol-MAPK8, quercetin-TP53, and quercetin-MAPK1 have excellent binding efficiency. Wogonin, a naturally occurring flavonoid compound, has anti-inflammatory, antioxidant, anticancer, and antiviral effects [34]. Wogonin regulates the redox process of chondrocytes and inhibits the biological activity of inflammatory mediators produced by macrophages and lymphocytes [35, 36]. In PCOS patients, endoplasmic reticulum stress induces granulosa cell apoptosis through death receptor 5 [37]. By controlling the process of endoplasmic reticulum stress, kaempferol, a natural flavonol active molecule, improves the survival rate of noncancer cells [38, 39]. The core ingredient of KTC was quercetin which had 71 targets that overlap with the potential therapeutic targets of KTC for PCOS. As one of the potential risk factors of PCOS, oxidative stress damages the insulin resistance, lipid metabolism, and follicular development of PCOS. Quercetin works as an antioxidant by lowering free radical generation, preventing lipid peroxidation, and altering antioxidants [40]. Studies have found that quercetin reduces the body weight, cysts, and ovarian diameter and restores healthy follicle function to alleviate the metabolic disorders of PCOS model rats [41]. Oral quercetin has been shown in clinical studies to successfully reduce adiponectin-mediated insulin resistance and hormone abnormalities in PCOS patients [42].

## 5. Conclusion

In this study, we uncovered the targets and molecular mechanisms of KTC in the treatment of PCOS and confirmed that quercetin may replace KTC for the treatment of PCOS patients through network pharmacology, bioinformatics, molecular docking, and other methods. These results may provide evidence for the clinical application of KTC in the treatment of PCOS.

## Data Availability

The data sets used and/or analyzed during the current study are available from the corresponding author on reasonable request.

## Abbreviations

KTC: Kuntai capsule
PCOS: Polycystic ovary syndrome
TCMSP: Traditional Chinese Medicine System Pharmacology Database and Analysis Platform
GEO: Gene Expression Omnibus
TCM: Traditional Chinese medicine
GO: Gene Ontology
KEGG: Kyoto Encyclopedia of Genes and Genomes
PPI: Protein-protein interaction.

## Supplementary Materials

Supplementary 1 Supplement Table 1: effective ingredients of KTC.

Supplementary 2 Supplement Table 2: therapeutic targets of effective ingredients.

Supplementary 3 Supplement Table 3: therapeutic targets of PCOS disease database and GEO database.

Supplementary 4 Supplement Table 4: the intersection of KTC's therapeutic targets and PCOS-related databases.

Supplementary 5 Supplement Table 5: analysis of PPI network, GO, and KEGG of KTC's potential therapeutic targets.

Supplementary 6 Supplement Table 6: molecular docking results of TP53, MAPK1, MAPK8, JUN, AKT1, and their corresponding effective ingredients.

Supplementary 7 Supplement Table 7: potential therapeutic targets of the active ingredients of KTC.

Supplementary 8 Supplement Table 8: analysis of PPI network, GO, and KEGG of quercetin's potential therapeutic targets.

Supplementary 9 Supplement Table 9: the R language scripts used in the study.

## References

[1] Glueck C. J., Goldenberg N. (2019). Characteristics of obesity in polycystic ovary syndrome: etiology, treatment, and genetics. *Metabolism*.

[2] Escobar-Morreale H. F. (2018). Polycystic ovary syndrome: definition, aetiology, diagnosis and treatment. *Nature Reviews. Endocrinology*.

[3] Balen A. H., Morley L. C., Misso M. (2016). The management of anovulatory infertility in women with polycystic ovary syndrome: an analysis of the evidence to support the development of global WHO guidance. *Human Reproduction Update*.

[4] Jin P., Xie Y. (2018). Treatment strategies for women with polycystic ovary syndrome. *Gynecological Endocrinology*.

[5] Shen W., Jin B., Pan Y. (2021). The effects of traditional Chinese medicine-associated complementary and alternative medicine on women with polycystic ovary syndrome. *Evidence-based Complementary and Alternative Medicine: eCAM*.

[6] Qiu Z., Dong J., Xue C. (2020). Liuwei Dihuang Pills alleviate the polycystic ovary syndrome with improved insulin sensitivity through PI3K/Akt signaling pathway. *Journal of Ethnopharmacology*.

[7] Zhang H., Qin F., Liu A. (2019). Retracted: Kuntai capsule attenuates premature ovarian failure through the PI3K/AKT/mTOR pathway. *Journal of Ethnopharmacology*.

[8] Zhang J., Fang L., Shi L. (2017). Protective effects and mechanisms investigation of Kuntai capsule on the ovarian function of a novel model with accelerated aging ovaries. *Journal of Ethnopharmacology*.

[9] Liang R., Liu Z., Li P. (2019). Kuntai capsules improve glucolipid metabolism in patients with polycystic ovary syndrome: a randomized, double-blind, placebo-controlled trial. *Medicine*.

[10] Zhang B., Chu N., Qiu X. M. (2018). Effects of Heyan Kuntai Capsule () on follicular development and oocyte cohesin levels in aged mice. *Chinese Journal of Integrative Medicine*.

[11] Wu Z., Li W., Liu G., Tang Y. (2018). Network-based methods for prediction of drug-target interactions. *Frontiers in Pharmacology*.

[12] Gao X., Li S., Cong C., Wang Y., Xu L. (2021). A network pharmacology approach to estimate potential targets of the active ingredients of epimedium for alleviating mild cognitive impairment and treating Alzheimer’s disease. *Evidence-Based Complementary and Alternative Medicine: eCAM*.

[13] Ru J., Li P., Wang J. (2014). TCMSP: A Database of Systems Pharmacology for Drug Discovery from Herbal Medicines. *Journal of Cheminformatics*.

[14] Yue J., López J. M. (2020). Understanding MAPK signaling pathways in apoptosis. *International Journal of Molecular Sciences*.

[15] Kupreeva M., Diane A., Lehner R. (2019). Effect of metformin and flutamide on insulin, lipogenic and androgen-estrogen signaling, and cardiometabolic risk in a PCOS-prone metabolic syndrome rodent model. *American Journal of Physiology. Endocrinology and Metabolism*.

[16] Kumariya S., Ubba V., Jha R. K., Gayen J. R. (2021). Autophagy in ovary and polycystic ovary syndrome: role, dispute and future perspective. *Autophagy*.

[17] Ranjan A., Iwakuma T. (2016). Non-canonical cell death induced by p 53. *International Journal of Molecular Sciences*.

[18] Yang R., Chen J., Wang L., Deng A. (2019). LncRNA BANCR participates in polycystic ovary syndrome by promoting cell apoptosis. *Molecular Medicine Reports*.

[19] Wernig G., Chen S. Y., Cui L. (2017). Unifying mechanism for different fibrotic diseases. *Proceedings of the National Academy of Sciences of the United States of America*.

[20] Makker A., Goel M. M., Mahdi A. A. (2014). PI3K/PTEN/Akt and TSC/mTOR signaling pathways, ovarian dysfunction, and infertility: an update. *Journal of Molecular Endocrinology*.

[21] Nekoonam S., Naji M., Nashtaei M. S. (2017). Expression of AKT1 along with AKT2 in granulosa-lutein cells of hyperandrogenic PCOS patients. *Archives of Gynecology and Obstetrics*.

[22] Ozcaka O., Buduneli N., Ceyhan B. O. (2013). Is interleukin-17 involved in the interaction between polycystic ovary syndrome and gingival inflammation?. *Journal of Periodontology*.

[23] Gao Q., Zhu H., Dong L. (2019). Integrated proteogenomic characterization of HBV-related hepatocellular carcinoma. *Cell*.

[24] Xie Q., Xiong X., Xiao N. (2019). Mesenchymal stem cells alleviate DHEA-induced polycystic ovary syndrome (PCOS) by inhibiting inflammation in mice. *Stem Cells International*.

[25] Zhang Q., Lou Y., Yang J. (2019). Integrated multiomic analysis reveals comprehensive tumour heterogeneity and novel immunophenotypic classification in hepatocellular carcinomas. *Gut*.

[26] Oróstica L., Astorga I., Plaza-Parrochia F. (2016). Proinflammatory environment and role of TNF-*α* in endometrial function of obese women having polycystic ovarian syndrome. *International Journal of Obesity*.

[27] Xu F., Liu R., Cao X. (2017). Hyperandrogenism stimulates inflammation and promote apoptosis of cumulus cells. *Cellular and Molecular Biology (Noisy-le-Grand, France)*.

[28] Qin Y., Li T., Zhao H., Mao Z., Ding C., Kang Y. (2021). Integrated transcriptomic and epigenetic study of PCOS: impact of Map3k1 and Map1lc3a promoter methylation on autophagy. *Frontiers in Genetics*.

[29] Gu B. X., Wang X., Yin B. L. (2016). Abnormal expression of TLRs may play a role in lower embryo quality of women with polycystic ovary syndrome. *Systems Biology in Reproductive Medicine*.

[30] Wang Y., He J., Yang J. (2018). Eicosapentaenoic acid improves polycystic ovary syndrome in rats via sterol regulatory element-binding protein 1 (SREBP-1)/toll-like receptor 4 (TLR4) pathway. *Medical Science Monitor*.

[31] Stepto N. K., Cassar S., Joham A. E. (2013). Women with polycystic ovary syndrome have intrinsic insulin resistance on euglycaemic-hyperinsulaemic clamp. *Human Reproduction*.

[32] Jiang B., Xue M., Xu D., Song Y., Zhu S. (2020). Retracted article: Upregulation of microRNA-204 improves insulin resistance of polycystic ovarian syndrome via inhibition of HMGB1 and the inactivation of the TLR4/NF-*κ*B pathway. *Cell Cycle*.

[33] Wang D., Weng Y., Zhang Y. (2020). Exposure to hyperandrogen drives ovarian dysfunction and fibrosis by activating the NLRP3 inflammasome in mice. *Science of The Total Environment*.

[34] Sharifi-Rad J., Herrera-Bravo J., Salazar L. A. (2021). The therapeutic potential of wogonin observed in preclinical studies. *Evidence-based Complementary and Alternative Medicine: eCAM*.

[35] Huynh D. L., Ngau T. H., Nguyen N. H., Tran G. B., Nguyen C. T. (2020). Potential therapeutic and pharmacological effects of Wogonin: an updated review. *Molecular Biology Reports*.

[36] Khan N. M., Haseeb A., Ansari M. Y., Devarapalli P., Haynie S., Haqqi T. M. (2017). Wogonin, a plant derived small molecule, exerts potent anti-inflammatory and chondroprotective effects through the activation of ROS/ERK/Nrf2 signaling pathways in human osteoarthritis chondrocytes. *Free Radical Biology & Medicine*.

[37] Azhary J. M. K., Harada M., Takahashi N. (2019). Endoplasmic reticulum stress activated by androgen enhances apoptosis of granulosa cells via induction of death receptor 5 in PCOS. *Endocrinology*.

[38] Ashrafizadeh M., Tavakol S., Ahmadi Z., Roomiani S., Mohammadinejad R., Samarghandian S. (2020). Therapeutic effects of kaempferol affecting autophagy and endoplasmic reticulum stress. *Phytotherapy Research: PTR*.

[39] Cao J., Wang Y., Hu S. (2020). Kaempferol ameliorates secretagogue-induced pseudo-allergic reactions via inhibiting intracellular calcium fluctuation. *The Journal of Pharmacy and Pharmacology*.

[40] Pourteymour Fard Tabrizi F., Hajizadeh-Sharafabad F., Vaezi M., Jafari-Vayghan H., Alizadeh M., Maleki V. (2020). Quercetin and polycystic ovary syndrome, current evidence and future directions: a systematic review. *Journal of Ovarian Research*.

[41] Jahan S., Abid A., Khalid S. (2018). Therapeutic potentials of quercetin in management of polycystic ovarian syndrome using letrozole induced rat model: a histological and a biochemical study. *Journal Of Ovarian Research*.

[42] Rezvan N., Moini A., Janani L. (2017). Effects of quercetin on adiponectin-mediated insulin sensitivity in polycystic ovary syndrome: a randomized placebo-controlled double-blind clinical trial. *Hormone and Metabolic Research*.

