# Supplementary material for: Identification of Bioactive Compounds and Potential Mechanisms of Kuntai Capsule in the Treatment of Polycystic Ovary Syndrome by Integrating Network Pharmacology and Bioinformatics
Source: Oxid Med Cell Longev. 2022 Apr 28;2022:3145938. doi: 10.1155/2022/3145938 (PMC9073551; doi:10.1155/2022/3145938)
Supplement: Supplementary 1 — Supplement Table 1: effective ingredients of KTC. [file 3145938.f1.pdf]

Mol ID	Hdon	Hacc	OB(%)	Caco-2	BBB	DL	FASA-	HL
MOL000359	1	1	36.913906	1.32059	0.87298	0.7512	20.23	5.371091
MOL000449	1	1	43.829852	1.44458	1.00045	0.75665	20.23	5.574595
MOL000422	4	6	41.88225	0.26096	-0.55335	0.24066	111.13	14.743371
MOL000492	5	6	54.826434	-0.03424	-0.72733	0.24164	110.38	0.609577
MOL001928	2	6	66.640769	-0.48821	-0.88431	0.32626	93.06	6.535831
MOL001918	1	6	87.593121	-0.09125	-0.56103	0.36678	82.06	7.451754
MOL001910	2	5	64.773893	0.08725	-0.59468	0.37586	79.29	2.621835
MOL001925	2	6	68.175762	-0.3376	-0.72612	0.39507	85.22	8.810559
MOL001919	2	4	43.556202	-0.00229	-0.40565	0.53276	74.6	4.336052
MOL001930	4	12	31.274473	-0.68923	-1.23618	0.74612	174.35	-1.845084
MOL000359	1	1	36.913906	1.32059	0.87298	0.7512	20.23	5.371091
MOL000358	1	1	36.913906	1.32463	0.98588	0.75123	20.23	5.355491
MOL000211	2	3	55.377073	0.73231	0.21532	0.7761	57.53	8.873708
MOL001924	5	11	53.870375	-1.47259	-1.86121	0.78709	164.37	13.882664
MOL001921	3	10	49.121317	-1.13469	-1.75511	0.79711	140.98	7.26361
MOL000282	1	1	43.507086	1.32195	0.90967	0.71939	20.23	5.113073
MOL000291	3	5	30.524601	-0.07527	-0.86893	0.7463	94.83	8.672073
MOL000292	2	4	38.151358	0.31611	-0.41341	0.74643	74.6	7.725726
MOL000296	1	1	36.913906	1.31876	0.96428	0.75072	20.23	5.347511
MOL000290	3	5	30.606946	-0.1368	-0.92991	0.76152	94.83	8.256824
MOL000279	3	3	37.963828	0.27629	-0.38974	0.77061	60.69	5.312703
MOL000275	2	3	38.7115	0.51877	-0.1423	0.80199	57.53	7.7791
MOL000287	2	3	38.699914	0.6067	-0.04064	0.8095	57.53	6.59447
MOL000289	2	5	33.62792	0.10154	-0.57273	0.81076	83.83	9.266582
MOL000276	2	5	35.105891	0.02743	-0.87172	0.81091	83.83	7.340838
MOL000283	1	3	40.36268	0.84419	0.34096	0.81255	38.69	3.428952
MOL000273	3	4	30.932142	0.01025	-0.7579	0.81281	77.76	6.812918
MOL000280	3	4	31.072057	0.05107	-0.69764	0.81528	77.76	7.418508
MOL000285	2	4	38.255158	0.11551	-0.57069	0.82014	74.6	6.77014
MOL000300	1	3	44.172299	0.38192	-0.1611	0.83458	60.36	7.037371
MOL000622	2	4	63.708884	0.02344	-0.24386	0.18833	66.76	3.173804
MOL008647	3	5	86.712159	0.54599	-0.50741	0.26454	78.79	3.712293
MOL000098	5	7	46.433348	0.04842	-0.7689	0.27525	131.36	14.400548
MOL000785	0	4	64.601113	1.3287	0.37072	0.64524	40.8	2.247782
MOL000762	6	8	35.358188	-0.38394	-1.46802	0.65003	155.52	33.174943
MOL002894	1	4	35.735511	1.06673	0.17408	0.7269	51.8	6.464688
MOL013352	0	7	43.286254	0.00775	-0.4298	0.76724	95.34	-13.044428
MOL002903	0	5	55.366873	1.03841	0.57189	0.77465	40.16	6.413736
MOL002907	3	6	104.95424	-0.911	-1.31348	0.77599	104.06	6.68222
MOL002897	0	4	43.092332	1.16601	0.39823	0.7761	40.8	6.098007
MOL001454	0	4	36.861245	1.24179	0.56718	0.77665	40.8	6.565936
MOL002904	0	6	36.680901	0.97215	0.17347	0.81596	58.92	7.331258
MOL001458	0	4	30.671852	1.21396	0.32474	0.85647	40.8	9.333815
MOL002668	0	4	45.833181	1.22152	0.24208	0.86552	40.8	8.407065
MOL000228	1	4	55.233174	0.87197	0.26171	0.20163	55.76	17.023316
MOL002913	3	5	40.037781	0.56056	0.18159	0.20722	86.99	16.127363
MOL000525	3	5	39.403972	0.59738	-0.17207	0.20723	90.9	16.925553
MOL002714	3	5	33.518919	0.63086	-0.05023	0.20888	90.9	16.249167
MOL002911	4	6	69.039876	-0.07223	-0.32196	0.21994	107.22	21.890109
MOL010415	0	2	39.275344	1.46201	1.241	0.2289	26.3	5.435461
MOL000173	2	5	30.684567	0.78776	0.03762	0.22942	79.9	17.746871
MOL002926	2	5	38.715066	0.71448	0.02549	0.22987	75.99	17.582155
MOL002937	2	5	66.061739	0.67465	0.12743	0.23057	75.99	17.165104

MOL002928	2	5	41.367569	0.76122	0.12657	0.23233	79.9	17.154856
MOL001689	2	5	34.973573	0.67146	-0.04689	0.24082	79.9	17.248472
MOL000073	5	6	48.959841	0.01948	-0.64284	0.24162	110.38	0.626389
MOL002910	4	6	41.150963	0.16103	-0.41926	0.24189	107.22	15.812348
MOL002914	4	6	41.350427	0.04932	-0.66394	0.2436	107.22	15.87634
MOL002925	4	6	37.013487	0.17858	-0.55682	0.24382	111.13	17.996386
MOL008206	1	5	44.08796	1.00657	0.53598	0.25331	68.9	17.018812
MOL012246	3	6	74.23522	0.37328	-0.43273	0.26479	96.22	16.849629
MOL002908	3	6	37.008374	0.75876	-0.06693	0.26546	100.13	16.169995
MOL002933	3	6	36.562005	0.4577	-0.39681	0.26666	100.13	16.929309
MOL012245	3	6	36.626886	0.43274	-0.3189	0.26833	96.22	16.121862
MOL002932	2	6	76.25705	0.84393	0.30644	0.2915	89.13	16.779089
MOL002917	3	7	45.047428	0.47539	-0.11487	0.33057	109.36	16.36913
MOL002915	1	6	49.065926	0.85539	-0.0269	0.33279	78.13	15.866038
MOL001490	0	4	43.593325	0.97947	0.67555	0.34531	52.6	3.02355
MOL000552	2	7	31.712465	0.92784	-0.00472	0.35462	98.36	16.469237
MOL012266	2	7	37.940234	0.64576	-0.12959	0.3663	98.36	16.24749
MOL002879	0	4	43.593325	0.7934	0.26014	0.39247	52.6	3.599307
MOL001506	0	0	33.545943	2.08183	1.73017	0.42161		2.721453
MOL002927	2	8	69.510434	0.6751	-0.0733	0.4379	107.59	16.141974
MOL002934	2	8	104.34461	0.73692	-0.19207	0.43917	107.59	16.49732
MOL002909	4	9	33.815826	0.35427	-0.59001	0.44739	138.82	15.939098
MOL000359	1	1	36.913906	1.32059	0.87298	0.7512	20.23	5.371091
MOL000358	1	1	36.913906	1.32463	0.98588	0.75123	20.23	5.355491
MOL000449	1	1	43.829852	1.44458	1.00045	0.75665	20.23	5.574595
MOL002897	0	4	43.092332	1.16601	0.39823	0.7761	40.8	6.098007
MOL001458	0	4	30.671852	1.21396	0.32474	0.85647	40.8	9.333815
