# Supplementary material for: Identification of Bioactive Compounds and Potential Mechanisms of Kuntai Capsule in the Treatment of Polycystic Ovary Syndrome by Integrating Network Pharmacology and Bioinformatics
Source: Oxid Med Cell Longev. 2022 Apr 28;2022:3145938. doi: 10.1155/2022/3145938 (PMC9073551; doi:10.1155/2022/3145938)
Supplement: Supplementary 2 — Supplement Table 2: therapeutic targets of effective ingredients. [file 3145938.f2.pdf]

Drug	MolId	Symbol
PAEONIAE RADIX ALBA	MOL001919	PGR
PAEONIAE RADIX ALBA	MOL001919	NR3C2
PAEONIAE RADIX ALBA	MOL001924	TNFAIP6
PAEONIAE RADIX ALBA	MOL001924	IL6R
PAEONIAE RADIX ALBA	MOL001924	CD14
PAEONIAE RADIX ALBA	MOL001924	LBP
PAEONIAE RADIX ALBA	MOL000211	PGR
PAEONIAE RADIX ALBA	MOL000358	PGR
PAEONIAE RADIX ALBA	MOL000358	NCOA2
PAEONIAE RADIX ALBA	MOL000358	PTGS1
PAEONIAE RADIX ALBA	MOL000358	PTGS2
PAEONIAE RADIX ALBA	MOL000358	HSP90AA1
PAEONIAE RADIX ALBA	MOL000358	KCNH2
PAEONIAE RADIX ALBA	MOL000358	PRKACA
PAEONIAE RADIX ALBA	MOL000358	DRD1
PAEONIAE RADIX ALBA	MOL000358	CHRM3
PAEONIAE RADIX ALBA	MOL000358	CHRM1
PAEONIAE RADIX ALBA	MOL000358	SCN5A
PAEONIAE RADIX ALBA	MOL000358	CHRM4
PAEONIAE RADIX ALBA	MOL000358	PDE3A
PAEONIAE RADIX ALBA	MOL000358	ADRA1A
PAEONIAE RADIX ALBA	MOL000358	CHRM2
PAEONIAE RADIX ALBA	MOL000358	ADRA1B
PAEONIAE RADIX ALBA	MOL000358	ADRB2
PAEONIAE RADIX ALBA	MOL000358	CHRNA2
PAEONIAE RADIX ALBA	MOL000358	SLC6A4
PAEONIAE RADIX ALBA	MOL000358	OPRM1
PAEONIAE RADIX ALBA	MOL000358	CHRNA7
PAEONIAE RADIX ALBA	MOL000358	BCL2
PAEONIAE RADIX ALBA	MOL000358	BAX
PAEONIAE RADIX ALBA	MOL000358	CASP9
PAEONIAE RADIX ALBA	MOL000358	JUN
PAEONIAE RADIX ALBA	MOL000358	CASP3
PAEONIAE RADIX ALBA	MOL000358	CASP8
PAEONIAE RADIX ALBA	MOL000358	PRKCA
PAEONIAE RADIX ALBA	MOL000358	PON1
PAEONIAE RADIX ALBA	MOL000358	MAP2
PAEONIAE RADIX ALBA	MOL000359	PGR
PAEONIAE RADIX ALBA	MOL000359	NCOA2
PAEONIAE RADIX ALBA	MOL000359	NR3C2
PAEONIAE RADIX ALBA	MOL000422	NOS2
PAEONIAE RADIX ALBA	MOL000422	PTGS1
PAEONIAE RADIX ALBA	MOL000422	AR
PAEONIAE RADIX ALBA	MOL000422	PPARG
PAEONIAE RADIX ALBA	MOL000422	PTGS2
PAEONIAE RADIX ALBA	MOL000422	HSP90AA1
PAEONIAE RADIX ALBA	MOL000422	PRKACA
PAEONIAE RADIX ALBA	MOL000422	NCOA2
PAEONIAE RADIX ALBA	MOL000422	DPP4
PAEONIAE RADIX ALBA	MOL000422	PRSS1
PAEONIAE RADIX ALBA	MOL000422	PGR
PAEONIAE RADIX ALBA	MOL000422	F2
PAEONIAE RADIX ALBA	MOL000422	CHRM1

PAEONIAE RADIX ALBA	MOL000422	ACHE
PAEONIAE RADIX ALBA	MOL000422	SLC6A2
PAEONIAE RADIX ALBA	MOL000422	CHRM2
PAEONIAE RADIX ALBA	MOL000422	ADRA1B
PAEONIAE RADIX ALBA	MOL000422	TOP2A
PAEONIAE RADIX ALBA	MOL000422	F7
PAEONIAE RADIX ALBA	MOL000422	CAMKMT
PAEONIAE RADIX ALBA	MOL000422	RELA
PAEONIAE RADIX ALBA	MOL000422	IKBKB
PAEONIAE RADIX ALBA	MOL000422	AKT1
PAEONIAE RADIX ALBA	MOL000422	BCL2
PAEONIAE RADIX ALBA	MOL000422	BAX
PAEONIAE RADIX ALBA	MOL000422	TNFAIP6
PAEONIAE RADIX ALBA	MOL000422	JUN
PAEONIAE RADIX ALBA	MOL000422	AHSA1
PAEONIAE RADIX ALBA	MOL000422	CASP3
PAEONIAE RADIX ALBA	MOL000422	MAPK8
PAEONIAE RADIX ALBA	MOL000422	MMP1
PAEONIAE RADIX ALBA	MOL000422	STAT1
PAEONIAE RADIX ALBA	MOL000422	CDK1
PAEONIAE RADIX ALBA	MOL000422	PPARG
PAEONIAE RADIX ALBA	MOL000422	HMOX1
PAEONIAE RADIX ALBA	MOL000422	CYP3A4
PAEONIAE RADIX ALBA	MOL000422	NPPB
PAEONIAE RADIX ALBA	MOL000422	CYP1A1
PAEONIAE RADIX ALBA	MOL000422	ICAM1
PAEONIAE RADIX ALBA	MOL000422	SELE
PAEONIAE RADIX ALBA	MOL000422	VCAM1
PAEONIAE RADIX ALBA	MOL000422	NR1I2
PAEONIAE RADIX ALBA	MOL000422	CYP1B1
PAEONIAE RADIX ALBA	MOL000422	ALOX5
PAEONIAE RADIX ALBA	MOL000422	HAS2
PAEONIAE RADIX ALBA	MOL000422	AHR
PAEONIAE RADIX ALBA	MOL000422	PSMD3
PAEONIAE RADIX ALBA	MOL000422	SLC2A4
PAEONIAE RADIX ALBA	MOL000422	NR1I3
PAEONIAE RADIX ALBA	MOL000422	INSRR
PAEONIAE RADIX ALBA	MOL000422	DIO1
PAEONIAE RADIX ALBA	MOL000422	PPP3CA
PAEONIAE RADIX ALBA	MOL000422	GSTM1
PAEONIAE RADIX ALBA	MOL000422	GSTM2
PAEONIAE RADIX ALBA	MOL000422	AKR1C3
PAEONIAE RADIX ALBA	MOL000422	SLPI
PAEONIAE RADIX ALBA	MOL000492	PTGS1
PAEONIAE RADIX ALBA	MOL000492	ESR1
PAEONIAE RADIX ALBA	MOL000492	PTGS2
PAEONIAE RADIX ALBA	MOL000492	HSP90AA1
PAEONIAE RADIX ALBA	MOL000492	DPEP1
PAEONIAE RADIX ALBA	MOL000492	PRKACA
PAEONIAE RADIX ALBA	MOL000492	NCOA2
PAEONIAE RADIX ALBA	MOL000492	CAMKMT
PAEONIAE RADIX ALBA	MOL000492	RXRA
PAEONIAE RADIX ALBA	MOL000492	NPPB
PAEONIAE RADIX ALBA	MOL000492	HAS2

PORID	MOL000273	NR3C2
PORID	MOL000273	NCOA2
PORID	MOL000275	NR3C2
PORID	MOL000279	NR3C2
PORID	MOL000282	PGR
PORID	MOL000283	PGR
PORID	MOL000296	PGR
PORID	MOL000296	NCOA2
PORID	MOL000296	CHRM3
PORID	MOL000296	CHRM1
PORID	MOL000296	CHRM2
PORID	MOL000296	ADRA1B
PORID	MOL000296	GRIA2
PORID	MOL000296	IGHG1
PORID	MOL000296	NPPB
PORID	MOL000296	NPPB
PORID	MOL000296	LYG1
PORID	MOL000296	PTGS1
PORID	MOL000296	SCN5A
PORID	MOL000296	PTGS2
PORID	MOL000296	RXRA
PORID	MOL000296	PDE3A
PORID	MOL000296	SLC6A2
COPTIDISRHIZOMA	MOL001454	NOS2
COPTIDISRHIZOMA	MOL001454	PTGS1
COPTIDISRHIZOMA	MOL001454	KCNH2
COPTIDISRHIZOMA	MOL001454	ESR1
COPTIDISRHIZOMA	MOL001454	AR
COPTIDISRHIZOMA	MOL001454	SCN5A
COPTIDISRHIZOMA	MOL001454	F10
COPTIDISRHIZOMA	MOL001454	PTGS2
COPTIDISRHIZOMA	MOL001454	RXRA
COPTIDISRHIZOMA	MOL001454	ADRB2
COPTIDISRHIZOMA	MOL001454	HSP90AA1
COPTIDISRHIZOMA	MOL001454	PRKACA
COPTIDISRHIZOMA	MOL001454	PRSS1
COPTIDISRHIZOMA	MOL001454	NCOA2
COPTIDISRHIZOMA	MOL001454	PDE10A
COPTIDISRHIZOMA	MOL001454	CAMKMT
COPTIDISRHIZOMA	MOL002894	NOS2
COPTIDISRHIZOMA	MOL002894	PTGS1
COPTIDISRHIZOMA	MOL002894	KCNH2
COPTIDISRHIZOMA	MOL002894	ESR1
COPTIDISRHIZOMA	MOL002894	AR
COPTIDISRHIZOMA	MOL002894	SCN5A
COPTIDISRHIZOMA	MOL002894	PTGS2
COPTIDISRHIZOMA	MOL002894	RXRA
COPTIDISRHIZOMA	MOL002894	PRKACA
COPTIDISRHIZOMA	MOL002894	PRSS1
COPTIDISRHIZOMA	MOL002894	NCOA2
COPTIDISRHIZOMA	MOL002894	CAMKMT
COPTIDISRHIZOMA	MOL002897	NOS2
COPTIDISRHIZOMA	MOL002897	KCNH2
COPTIDISRHIZOMA	MOL002897	ESR1

COPTIDISRHIZOMA	MOL002897	AR
COPTIDISRHIZOMA	MOL002897	PTGS2
COPTIDISRHIZOMA	MOL002897	RXRA
COPTIDISRHIZOMA	MOL002897	PRKACA
COPTIDISRHIZOMA	MOL002897	PRSS1
COPTIDISRHIZOMA	MOL002897	NCOA2
COPTIDISRHIZOMA	MOL002897	PDE10A
COPTIDISRHIZOMA	MOL002903	PTGS1
COPTIDISRHIZOMA	MOL002903	CHRM3
COPTIDISRHIZOMA	MOL002903	KCNH2
COPTIDISRHIZOMA	MOL002903	CHRM1
COPTIDISRHIZOMA	MOL002903	SCN5A
COPTIDISRHIZOMA	MOL002903	F10
COPTIDISRHIZOMA	MOL002903	CHRM5
COPTIDISRHIZOMA	MOL002903	PTGS2
COPTIDISRHIZOMA	MOL002903	ADRA2C
COPTIDISRHIZOMA	MOL002903	CHRM4
COPTIDISRHIZOMA	MOL002903	OPRD1
COPTIDISRHIZOMA	MOL002903	ADRA1B
COPTIDISRHIZOMA	MOL002903	SLC6A3
COPTIDISRHIZOMA	MOL002903	ADRB2
COPTIDISRHIZOMA	MOL002903	ADRA1D
COPTIDISRHIZOMA	MOL002903	SLC6A4
COPTIDISRHIZOMA	MOL002903	OPRM1
COPTIDISRHIZOMA	MOL002903	HSP90AA1
COPTIDISRHIZOMA	MOL002903	PRKACA
COPTIDISRHIZOMA	MOL002903	PDE10A
COPTIDISRHIZOMA	MOL002903	CAMKMT
COPTIDISRHIZOMA	MOL002903	DRD1
COPTIDISRHIZOMA	MOL002903	DRD5
COPTIDISRHIZOMA	MOL002903	RXRA
COPTIDISRHIZOMA	MOL002903	SLC6A2
COPTIDISRHIZOMA	MOL002903	ADRA1A
COPTIDISRHIZOMA	MOL002903	CHRM2
COPTIDISRHIZOMA	MOL002904	NOS2
COPTIDISRHIZOMA	MOL002904	PTGS1
COPTIDISRHIZOMA	MOL002904	CHRM3
COPTIDISRHIZOMA	MOL002904	KCNH2
COPTIDISRHIZOMA	MOL002904	AR
COPTIDISRHIZOMA	MOL002904	SCN5A
COPTIDISRHIZOMA	MOL002904	F10
COPTIDISRHIZOMA	MOL002904	PTGS2
COPTIDISRHIZOMA	MOL002904	F7
COPTIDISRHIZOMA	MOL002904	RXRA
COPTIDISRHIZOMA	MOL002904	ADRA1B
COPTIDISRHIZOMA	MOL002904	ADRB2
COPTIDISRHIZOMA	MOL002904	ADRA1D
COPTIDISRHIZOMA	MOL002904	HSP90AA1
COPTIDISRHIZOMA	MOL002904	PRKACA
COPTIDISRHIZOMA	MOL002904	PRSS1
COPTIDISRHIZOMA	MOL002904	NCOA2
COPTIDISRHIZOMA	MOL002904	CAMKMT
COPTIDISRHIZOMA	MOL002907	NR3C2
COPTIDISRHIZOMA	MOL002907	NCOA2

COPTIDISRHIZOMA	MOL000622	GRIA2
COPTIDISRHIZOMA	MOL000785	NOS2
COPTIDISRHIZOMA	MOL000785	PTGS1
COPTIDISRHIZOMA	MOL000785	KCNH2
COPTIDISRHIZOMA	MOL000785	ESR1
COPTIDISRHIZOMA	MOL000785	AR
COPTIDISRHIZOMA	MOL000785	SCN5A
COPTIDISRHIZOMA	MOL000785	PTGS2
COPTIDISRHIZOMA	MOL000785	RXRA
COPTIDISRHIZOMA	MOL000785	ADRB2
COPTIDISRHIZOMA	MOL000785	ESR2
COPTIDISRHIZOMA	MOL000785	HSP90AA1
COPTIDISRHIZOMA	MOL000785	PRSS1
COPTIDISRHIZOMA	MOL000785	NCOA2
COPTIDISRHIZOMA	MOL000785	CAMKMT
COPTIDISRHIZOMA	MOL000785	PRKACA
COPTIDISRHIZOMA	MOL000785	CDK2
COPTIDISRHIZOMA	MOL000785	F7
COPTIDISRHIZOMA	MOL000098	PTGS1
COPTIDISRHIZOMA	MOL000098	AR
COPTIDISRHIZOMA	MOL000098	PPARG
COPTIDISRHIZOMA	MOL000098	PTGS2
COPTIDISRHIZOMA	MOL000098	HSP90AA1
COPTIDISRHIZOMA	MOL000098	NCOA2
COPTIDISRHIZOMA	MOL000098	DPP4
COPTIDISRHIZOMA	MOL000098	AKR1B1
COPTIDISRHIZOMA	MOL000098	PRSS1
COPTIDISRHIZOMA	MOL000098	TOP2A
COPTIDISRHIZOMA	MOL000098	F2
COPTIDISRHIZOMA	MOL000098	KCNH2
COPTIDISRHIZOMA	MOL000098	SCN5A
COPTIDISRHIZOMA	MOL000098	F10
COPTIDISRHIZOMA	MOL000098	ADRB2
COPTIDISRHIZOMA	MOL000098	MMP3
COPTIDISRHIZOMA	MOL000098	PRKACA
COPTIDISRHIZOMA	MOL000098	F7
COPTIDISRHIZOMA	MOL000098	RXRA
COPTIDISRHIZOMA	MOL000098	ACHE
COPTIDISRHIZOMA	MOL000098	MAOB
COPTIDISRHIZOMA	MOL000098	RELA
COPTIDISRHIZOMA	MOL000098	EGFR
COPTIDISRHIZOMA	MOL000098	AKT1
COPTIDISRHIZOMA	MOL000098	NPPB
COPTIDISRHIZOMA	MOL000098	CCND1
COPTIDISRHIZOMA	MOL000098	BCL2
COPTIDISRHIZOMA	MOL000098	BCL2L1
COPTIDISRHIZOMA	MOL000098	FOS
COPTIDISRHIZOMA	MOL000098	CDKN1A
COPTIDISRHIZOMA	MOL000098	EIF6
COPTIDISRHIZOMA	MOL000098	BAX
COPTIDISRHIZOMA	MOL000098	CASP9
COPTIDISRHIZOMA	MOL000098	PLAU
COPTIDISRHIZOMA	MOL000098	MMP2
COPTIDISRHIZOMA	MOL000098	MMP9

COPTIDISRHIZOMA	MOL000098	MAPK1
COPTIDISRHIZOMA	MOL000098	IL10RA
COPTIDISRHIZOMA	MOL000098	RB1
COPTIDISRHIZOMA	MOL000098	TNFAIP6
COPTIDISRHIZOMA	MOL000098	JUN
COPTIDISRHIZOMA	MOL000098	IL6R
COPTIDISRHIZOMA	MOL000098	AHSA1
COPTIDISRHIZOMA	MOL000098	CASP3
COPTIDISRHIZOMA	MOL000098	TP53
COPTIDISRHIZOMA	MOL000098	ELK1
COPTIDISRHIZOMA	MOL000098	NFKBIA
COPTIDISRHIZOMA	MOL000098	ODC1
COPTIDISRHIZOMA	MOL000098	CASP8
COPTIDISRHIZOMA	MOL000098	TOP1
COPTIDISRHIZOMA	MOL000098	RAF1
COPTIDISRHIZOMA	MOL000098	SOD1
COPTIDISRHIZOMA	MOL000098	PRKCA
COPTIDISRHIZOMA	MOL000098	MMP1
COPTIDISRHIZOMA	MOL000098	HIF1A
COPTIDISRHIZOMA	MOL000098	STAT1
COPTIDISRHIZOMA	MOL000098	RUNX1T1
COPTIDISRHIZOMA	MOL000098	CDK1
COPTIDISRHIZOMA	MOL000098	HSPA5
COPTIDISRHIZOMA	MOL000098	ERBB2
COPTIDISRHIZOMA	MOL000098	PPARG
COPTIDISRHIZOMA	MOL000098	ACACA
COPTIDISRHIZOMA	MOL000098	HMOX1
COPTIDISRHIZOMA	MOL000098	CYP3A4
COPTIDISRHIZOMA	MOL000098	CAV1
COPTIDISRHIZOMA	MOL000098	MYC
COPTIDISRHIZOMA	MOL000098	F3
COPTIDISRHIZOMA	MOL000098	GJA1
COPTIDISRHIZOMA	MOL000098	CYP1A1
COPTIDISRHIZOMA	MOL000098	ICAM1
COPTIDISRHIZOMA	MOL000098	IL1B
COPTIDISRHIZOMA	MOL000098	CCL2
COPTIDISRHIZOMA	MOL000098	SELE
COPTIDISRHIZOMA	MOL000098	VCAM1
COPTIDISRHIZOMA	MOL000098	CXCL8
COPTIDISRHIZOMA	MOL000098	PRKCB
COPTIDISRHIZOMA	MOL000098	BIRC5
COPTIDISRHIZOMA	MOL000098	DUOX2
COPTIDISRHIZOMA	MOL000098	NOS3
COPTIDISRHIZOMA	MOL000098	HSPB1
COPTIDISRHIZOMA	MOL000098	IL2RA
COPTIDISRHIZOMA	MOL000098	NR1I2
COPTIDISRHIZOMA	MOL000098	CYP1B1
COPTIDISRHIZOMA	MOL000098	CCNB1
COPTIDISRHIZOMA	MOL000098	PLAT
COPTIDISRHIZOMA	MOL000098	THBD
COPTIDISRHIZOMA	MOL000098	SERPINE1
COPTIDISRHIZOMA	MOL000098	IFNG
COPTIDISRHIZOMA	MOL000098	ALOX5
COPTIDISRHIZOMA	MOL000098	IL1A

COPTIDISRHIZOMA	MOL000098	MPO
COPTIDISRHIZOMA	MOL000098	TOP2A
COPTIDISRHIZOMA	MOL000098	NCF1
COPTIDISRHIZOMA	MOL000098	ABCG2
COPTIDISRHIZOMA	MOL000098	HAS2
COPTIDISRHIZOMA	MOL000098	NFE2L2
COPTIDISRHIZOMA	MOL000098	NQO1
COPTIDISRHIZOMA	MOL000098	PARP1
COPTIDISRHIZOMA	MOL000098	AHR
COPTIDISRHIZOMA	MOL000098	PSMD3
COPTIDISRHIZOMA	MOL000098	SLC2A4
COPTIDISRHIZOMA	MOL000098	COL3A1
COPTIDISRHIZOMA	MOL000098	CXCL11
COPTIDISRHIZOMA	MOL000098	CXCL2
COPTIDISRHIZOMA	MOL000098	DCAF5
COPTIDISRHIZOMA	MOL000098	NR1I3
COPTIDISRHIZOMA	MOL000098	CHEK2
COPTIDISRHIZOMA	MOL000098	INSRR
COPTIDISRHIZOMA	MOL000098	CLDN4
COPTIDISRHIZOMA	MOL000098	PPARA
COPTIDISRHIZOMA	MOL000098	PPARD
COPTIDISRHIZOMA	MOL000098	HSF1
COPTIDISRHIZOMA	MOL000098	CXCL10
COPTIDISRHIZOMA	MOL000098	CHUK
COPTIDISRHIZOMA	MOL000098	SPP1
COPTIDISRHIZOMA	MOL000098	RUNX2
COPTIDISRHIZOMA	MOL000098	RASSF1
COPTIDISRHIZOMA	MOL000098	E2F1
COPTIDISRHIZOMA	MOL000098	E2F2
COPTIDISRHIZOMA	MOL000098	ACP3
COPTIDISRHIZOMA	MOL000098	CTSD
COPTIDISRHIZOMA	MOL000098	IGFBP3
COPTIDISRHIZOMA	MOL000098	IGF2
COPTIDISRHIZOMA	MOL000098	CD40LG
COPTIDISRHIZOMA	MOL000098	IRF1
COPTIDISRHIZOMA	MOL000098	ERBB3
COPTIDISRHIZOMA	MOL000098	PON1
COPTIDISRHIZOMA	MOL000098	DIO1
COPTIDISRHIZOMA	MOL000098	PCOLCE
COPTIDISRHIZOMA	MOL000098	NPEPPS
COPTIDISRHIZOMA	MOL000098	HK2
COPTIDISRHIZOMA	MOL000098	RASA1
COPTIDISRHIZOMA	MOL000098	GSTM1
COPTIDISRHIZOMA	MOL000098	GSTM2
COPTIDISRHIZOMA	MOL001458	NOS2
COPTIDISRHIZOMA	MOL001458	PTGS1
COPTIDISRHIZOMA	MOL001458	KCNH2
COPTIDISRHIZOMA	MOL001458	ESR1
COPTIDISRHIZOMA	MOL001458	AR
COPTIDISRHIZOMA	MOL001458	SCN5A
COPTIDISRHIZOMA	MOL001458	PTGS2
COPTIDISRHIZOMA	MOL001458	PRSS1
COPTIDISRHIZOMA	MOL002668	NOS2
COPTIDISRHIZOMA	MOL002668	PTGS1

COPTIDISRHIZOMA	MOL002668	ESR1
COPTIDISRHIZOMA	MOL002668	AR
COPTIDISRHIZOMA	MOL002668	PTGS2
COPTIDISRHIZOMA	MOL002668	CHEK1
RADIX SCUTELLARIAE	MOL001689	NOS2
RADIX SCUTELLARIAE	MOL001689	PTGS1
RADIX SCUTELLARIAE	MOL001689	AR
RADIX SCUTELLARIAE	MOL001689	PTGS2
RADIX SCUTELLARIAE	MOL001689	DPP4
RADIX SCUTELLARIAE	MOL001689	HSP90AA1
RADIX SCUTELLARIAE	MOL001689	CDK2
RADIX SCUTELLARIAE	MOL001689	PRKACA
RADIX SCUTELLARIAE	MOL001689	PRSS1
RADIX SCUTELLARIAE	MOL001689	NCOA2
RADIX SCUTELLARIAE	MOL001689	NCOA1
RADIX SCUTELLARIAE	MOL001689	CAMKMT
RADIX SCUTELLARIAE	MOL001689	CHEK1
RADIX SCUTELLARIAE	MOL001689	ADRB2
RADIX SCUTELLARIAE	MOL001689	PDE3A
RADIX SCUTELLARIAE	MOL001689	RELA
RADIX SCUTELLARIAE	MOL001689	BCL2
RADIX SCUTELLARIAE	MOL001689	CDKN1A
RADIX SCUTELLARIAE	MOL001689	BAX
RADIX SCUTELLARIAE	MOL001689	CASP3
RADIX SCUTELLARIAE	MOL001689	TP53
RADIX SCUTELLARIAE	MOL001689	CASP8
RADIX SCUTELLARIAE	MOL001689	FASN
RADIX SCUTELLARIAE	MOL001689	FASLG
RADIX SCUTELLARIAE	MOL001689	CYP19A1
RADIX SCUTELLARIAE	MOL000173	NOS2
RADIX SCUTELLARIAE	MOL000173	PTGS1
RADIX SCUTELLARIAE	MOL000173	ESR1
RADIX SCUTELLARIAE	MOL000173	AR
RADIX SCUTELLARIAE	MOL000173	SCN5A
RADIX SCUTELLARIAE	MOL000173	PPARG
RADIX SCUTELLARIAE	MOL000173	PTGS2
RADIX SCUTELLARIAE	MOL000173	RXRA
RADIX SCUTELLARIAE	MOL000173	PDE3A
RADIX SCUTELLARIAE	MOL000173	DPP4
RADIX SCUTELLARIAE	MOL000173	MAPK14
RADIX SCUTELLARIAE	MOL000173	GSK3B
RADIX SCUTELLARIAE	MOL000173	HSP90AA1
RADIX SCUTELLARIAE	MOL000173	CDK2
RADIX SCUTELLARIAE	MOL000173	CHEK1
RADIX SCUTELLARIAE	MOL000173	PRKACA
RADIX SCUTELLARIAE	MOL000173	PRSS1
RADIX SCUTELLARIAE	MOL000173	CAMKMT
RADIX SCUTELLARIAE	MOL000173	ADRB2
RADIX SCUTELLARIAE	MOL000173	RELA
RADIX SCUTELLARIAE	MOL000173	AKT1
RADIX SCUTELLARIAE	MOL000173	CCND1
RADIX SCUTELLARIAE	MOL000173	BCL2
RADIX SCUTELLARIAE	MOL000173	CDKN1A
RADIX SCUTELLARIAE	MOL000173	EIF6

RADIX SCUTELLARIAE	MOL000173	BAX
RADIX SCUTELLARIAE	MOL000173	CASP9
RADIX SCUTELLARIAE	MOL000173	KDR
RADIX SCUTELLARIAE	MOL000173	TNFAIP6
RADIX SCUTELLARIAE	MOL000173	JUN
RADIX SCUTELLARIAE	MOL000173	IL6R
RADIX SCUTELLARIAE	MOL000173	AHSA1
RADIX SCUTELLARIAE	MOL000173	CASP3
RADIX SCUTELLARIAE	MOL000173	TP53
RADIX SCUTELLARIAE	MOL000173	TEP1
RADIX SCUTELLARIAE	MOL000173	MMP1
RADIX SCUTELLARIAE	MOL000173	CCL2
RADIX SCUTELLARIAE	MOL000173	PRKCD
RADIX SCUTELLARIAE	MOL000173	FN1
RADIX SCUTELLARIAE	MOL000173	CXCL8
RADIX SCUTELLARIAE	MOL000173	MCL1
RADIX SCUTELLARIAE	MOL000228	PTGS1
RADIX SCUTELLARIAE	MOL000228	DRD1
RADIX SCUTELLARIAE	MOL000228	CHRM3
RADIX SCUTELLARIAE	MOL000228	CHRM1
RADIX SCUTELLARIAE	MOL000228	ESR1
RADIX SCUTELLARIAE	MOL000228	SCN5A
RADIX SCUTELLARIAE	MOL000228	PTGS2
RADIX SCUTELLARIAE	MOL000228	RXRA
RADIX SCUTELLARIAE	MOL000228	PDE3A
RADIX SCUTELLARIAE	MOL000228	ADRA1A
RADIX SCUTELLARIAE	MOL000228	ADRA1B
RADIX SCUTELLARIAE	MOL000228	SLC6A3
RADIX SCUTELLARIAE	MOL000228	ADRB2
RADIX SCUTELLARIAE	MOL000228	SLC6A4
RADIX SCUTELLARIAE	MOL000228	HSP90AA1
RADIX SCUTELLARIAE	MOL000228	PRKACA
RADIX SCUTELLARIAE	MOL000228	PKIA
RADIX SCUTELLARIAE	MOL000228	CHRNA7
RADIX SCUTELLARIAE	MOL000228	MAOB
RADIX SCUTELLARIAE	MOL000228	CAMKMT
RADIX SCUTELLARIAE	MOL002714	PTGS1
RADIX SCUTELLARIAE	MOL002714	AR
RADIX SCUTELLARIAE	MOL002714	PTGS2
RADIX SCUTELLARIAE	MOL002714	HSP90AA1
RADIX SCUTELLARIAE	MOL002714	PRKACA
RADIX SCUTELLARIAE	MOL002714	DPP4
RADIX SCUTELLARIAE	MOL002714	PDE3A
RADIX SCUTELLARIAE	MOL002714	PRSS1
RADIX SCUTELLARIAE	MOL002714	NCOA2
RADIX SCUTELLARIAE	MOL002714	NCOA1
RADIX SCUTELLARIAE	MOL002714	CAMKMT
RADIX SCUTELLARIAE	MOL002714	RELA
RADIX SCUTELLARIAE	MOL002714	AKT1
RADIX SCUTELLARIAE	MOL002714	NPPB
RADIX SCUTELLARIAE	MOL002714	BCL2
RADIX SCUTELLARIAE	MOL002714	FOS
RADIX SCUTELLARIAE	MOL002714	BAX
RADIX SCUTELLARIAE	MOL002714	MMP9

RADIX SCUTELLARIAE	MOL002714	CASP3
RADIX SCUTELLARIAE	MOL002714	TP53
RADIX SCUTELLARIAE	MOL002714	HIF1A
RADIX SCUTELLARIAE	MOL002714	FOSL1
RADIX SCUTELLARIAE	MOL002714	FOSL2
RADIX SCUTELLARIAE	MOL002714	CDK1
RADIX SCUTELLARIAE	MOL002714	CCNB1
RADIX SCUTELLARIAE	MOL002714	MPO
RADIX SCUTELLARIAE	MOL002714	AHR
RADIX SCUTELLARIAE	MOL002714	IGF2
RADIX SCUTELLARIAE	MOL002714	CYCS
RADIX SCUTELLARIAE	MOL002714	NFATC1
RADIX SCUTELLARIAE	MOL002714	TDRD7
RADIX SCUTELLARIAE	MOL002714	EGLN1
RADIX SCUTELLARIAE	MOL002714	NOX5
RADIX SCUTELLARIAE	MOL002714	FABP5
RADIX SCUTELLARIAE	MOL002714	APOD
RADIX SCUTELLARIAE	MOL002909	NOS2
RADIX SCUTELLARIAE	MOL002909	F2
RADIX SCUTELLARIAE	MOL002909	AR
RADIX SCUTELLARIAE	MOL002909	F10
RADIX SCUTELLARIAE	MOL002909	PTGS2
RADIX SCUTELLARIAE	MOL002909	PTPN1
RADIX SCUTELLARIAE	MOL002909	TOP2A
RADIX SCUTELLARIAE	MOL002909	DPP4
RADIX SCUTELLARIAE	MOL002909	PYGM
RADIX SCUTELLARIAE	MOL002909	HSP90AA1
RADIX SCUTELLARIAE	MOL002909	PRSS1
RADIX SCUTELLARIAE	MOL002909	NCOA2
RADIX SCUTELLARIAE	MOL002910	PTGS1
RADIX SCUTELLARIAE	MOL002910	PTGS2
RADIX SCUTELLARIAE	MOL002910	HSP90AA1
RADIX SCUTELLARIAE	MOL002910	PRKACA
RADIX SCUTELLARIAE	MOL002913	PTGS1
RADIX SCUTELLARIAE	MOL002913	PTGS2
RADIX SCUTELLARIAE	MOL002913	HSP90AA1
RADIX SCUTELLARIAE	MOL002913	PRKACA
RADIX SCUTELLARIAE	MOL002914	PTGS1
RADIX SCUTELLARIAE	MOL002914	PTGS2
RADIX SCUTELLARIAE	MOL002914	HSP90AA1
RADIX SCUTELLARIAE	MOL002914	PRKACA
RADIX SCUTELLARIAE	MOL002914	NCOA2
RADIX SCUTELLARIAE	MOL002914	PYGM
RADIX SCUTELLARIAE	MOL002914	CAMKMT
RADIX SCUTELLARIAE	MOL002915	NOS2
RADIX SCUTELLARIAE	MOL002915	PTGS1
RADIX SCUTELLARIAE	MOL002915	F2
RADIX SCUTELLARIAE	MOL002915	SCN5A
RADIX SCUTELLARIAE	MOL002915	F10
RADIX SCUTELLARIAE	MOL002915	PTGS2
RADIX SCUTELLARIAE	MOL002915	RXRA
RADIX SCUTELLARIAE	MOL002915	ACHE
RADIX SCUTELLARIAE	MOL002915	ADRA1B
RADIX SCUTELLARIAE	MOL002915	ADRB2

RADIX SCUTELLARIAE	MOL002915	DPP4
RADIX SCUTELLARIAE	MOL002915	HSP90AA1
RADIX SCUTELLARIAE	MOL002915	IGHG1
RADIX SCUTELLARIAE	MOL002915	PRSS1
RADIX SCUTELLARIAE	MOL002915	NCOA2
RADIX SCUTELLARIAE	MOL002915	CAMKMT
RADIX SCUTELLARIAE	MOL002915	F7
RADIX SCUTELLARIAE	MOL002917	NOS2
RADIX SCUTELLARIAE	MOL002917	PTGS1
RADIX SCUTELLARIAE	MOL002917	AR
RADIX SCUTELLARIAE	MOL002917	SCN5A
RADIX SCUTELLARIAE	MOL002917	F10
RADIX SCUTELLARIAE	MOL002917	PTGS2
RADIX SCUTELLARIAE	MOL002917	TOP2A
RADIX SCUTELLARIAE	MOL002917	ESR2
RADIX SCUTELLARIAE	MOL002917	DPP4
RADIX SCUTELLARIAE	MOL002917	HSP90AA1
RADIX SCUTELLARIAE	MOL002917	CDK2
RADIX SCUTELLARIAE	MOL002917	CHEK1
RADIX SCUTELLARIAE	MOL002917	PRSS1
RADIX SCUTELLARIAE	MOL002917	NCOA2
RADIX SCUTELLARIAE	MOL002917	CAMKMT
RADIX SCUTELLARIAE	MOL002925	PTGS1
RADIX SCUTELLARIAE	MOL002925	AR
RADIX SCUTELLARIAE	MOL002925	PTGS2
RADIX SCUTELLARIAE	MOL002925	DPP4
RADIX SCUTELLARIAE	MOL002925	HSP90AA1
RADIX SCUTELLARIAE	MOL002925	PRKACA
RADIX SCUTELLARIAE	MOL002927	NOS2
RADIX SCUTELLARIAE	MOL002927	PTGS1
RADIX SCUTELLARIAE	MOL002927	F2
RADIX SCUTELLARIAE	MOL002927	KCNH2
RADIX SCUTELLARIAE	MOL002927	AR
RADIX SCUTELLARIAE	MOL002927	SCN5A
RADIX SCUTELLARIAE	MOL002927	F10
RADIX SCUTELLARIAE	MOL002927	PTGS2
RADIX SCUTELLARIAE	MOL002927	F7
RADIX SCUTELLARIAE	MOL002927	KDR
RADIX SCUTELLARIAE	MOL002927	CACNA2D1
RADIX SCUTELLARIAE	MOL002927	TOP2A
RADIX SCUTELLARIAE	MOL002927	DPP4
RADIX SCUTELLARIAE	MOL002927	HSP90AA1
RADIX SCUTELLARIAE	MOL002927	IGHG1
RADIX SCUTELLARIAE	MOL002927	PRSS1
RADIX SCUTELLARIAE	MOL002927	NCOA2
RADIX SCUTELLARIAE	MOL002927	NCOA1
RADIX SCUTELLARIAE	MOL002927	CAMKMT
RADIX SCUTELLARIAE	MOL002928	NOS2
RADIX SCUTELLARIAE	MOL002928	PTGS1
RADIX SCUTELLARIAE	MOL002928	AR
RADIX SCUTELLARIAE	MOL002928	SCN5A
RADIX SCUTELLARIAE	MOL002928	PTGS2
RADIX SCUTELLARIAE	MOL002928	RXRA
RADIX SCUTELLARIAE	MOL002928	PDE3A

RADIX SCUTELLARIAE	MOL002928	ADRA1B
RADIX SCUTELLARIAE	MOL002928	ADRB2
RADIX SCUTELLARIAE	MOL002928	DPP4
RADIX SCUTELLARIAE	MOL002928	HSP90AA1
RADIX SCUTELLARIAE	MOL002928	PRKACA
RADIX SCUTELLARIAE	MOL002928	PRSS1
RADIX SCUTELLARIAE	MOL002928	NCOA1
RADIX SCUTELLARIAE	MOL002928	CAMKMT
RADIX SCUTELLARIAE	MOL002928	NCOA2
RADIX SCUTELLARIAE	MOL002928	PKIA
RADIX SCUTELLARIAE	MOL002928	BCL2
RADIX SCUTELLARIAE	MOL002928	IL6R
RADIX SCUTELLARIAE	MOL002928	CASP3
RADIX SCUTELLARIAE	MOL002928	CDK1
RADIX SCUTELLARIAE	MOL002928	NPPB
RADIX SCUTELLARIAE	MOL002928	CCNB1
RADIX SCUTELLARIAE	MOL002928	CDK7
RADIX SCUTELLARIAE	MOL002928	CYP2C9
RADIX SCUTELLARIAE	MOL002932	NOS2
RADIX SCUTELLARIAE	MOL002932	PTGS1
RADIX SCUTELLARIAE	MOL002932	AR
RADIX SCUTELLARIAE	MOL002932	SCN5A
RADIX SCUTELLARIAE	MOL002932	PTGS2
RADIX SCUTELLARIAE	MOL002932	ESR2
RADIX SCUTELLARIAE	MOL002932	DPP4
RADIX SCUTELLARIAE	MOL002932	HSP90AA1
RADIX SCUTELLARIAE	MOL002932	CDK2
RADIX SCUTELLARIAE	MOL002932	CHEK1
RADIX SCUTELLARIAE	MOL002932	PRSS1
RADIX SCUTELLARIAE	MOL002932	CAMKMT
RADIX SCUTELLARIAE	MOL002932	NCOA1
RADIX SCUTELLARIAE	MOL002933	NOS2
RADIX SCUTELLARIAE	MOL002933	PTGS1
RADIX SCUTELLARIAE	MOL002933	ESR1
RADIX SCUTELLARIAE	MOL002933	AR
RADIX SCUTELLARIAE	MOL002933	PPARG
RADIX SCUTELLARIAE	MOL002933	PTGS2
RADIX SCUTELLARIAE	MOL002933	DPP4
RADIX SCUTELLARIAE	MOL002933	PYGM
RADIX SCUTELLARIAE	MOL002933	MAPK14
RADIX SCUTELLARIAE	MOL002933	GSK3B
RADIX SCUTELLARIAE	MOL002933	HSP90AA1
RADIX SCUTELLARIAE	MOL002933	CDK2
RADIX SCUTELLARIAE	MOL002933	CHEK1
RADIX SCUTELLARIAE	MOL002933	PRSS1
RADIX SCUTELLARIAE	MOL002933	NCOA2
RADIX SCUTELLARIAE	MOL002933	CAMKMT
RADIX SCUTELLARIAE	MOL002933	PRKACA
RADIX SCUTELLARIAE	MOL002934	NOS2
RADIX SCUTELLARIAE	MOL002934	F2
RADIX SCUTELLARIAE	MOL002934	KCNH2
RADIX SCUTELLARIAE	MOL002934	ESR1
RADIX SCUTELLARIAE	MOL002934	AR
RADIX SCUTELLARIAE	MOL002934	SCN5A

RADIX SCUTELLARIAE	MOL002934	PPARG
RADIX SCUTELLARIAE	MOL002934	F10
RADIX SCUTELLARIAE	MOL002934	PTGS2
RADIX SCUTELLARIAE	MOL002934	F7
RADIX SCUTELLARIAE	MOL002934	PTPN1
RADIX SCUTELLARIAE	MOL002934	TOP2A
RADIX SCUTELLARIAE	MOL002934	ESR2
RADIX SCUTELLARIAE	MOL002934	DPP4
RADIX SCUTELLARIAE	MOL002934	PYGM
RADIX SCUTELLARIAE	MOL002934	GSK3B
RADIX SCUTELLARIAE	MOL002934	HSP90AA1
RADIX SCUTELLARIAE	MOL002934	CHEK1
RADIX SCUTELLARIAE	MOL002934	PRSS1
RADIX SCUTELLARIAE	MOL002934	NCOA2
RADIX SCUTELLARIAE	MOL002934	CAMKMT
RADIX SCUTELLARIAE	MOL002937	PTGS1
RADIX SCUTELLARIAE	MOL002937	SCN5A
RADIX SCUTELLARIAE	MOL002937	PTGS2
RADIX SCUTELLARIAE	MOL002937	RXRA
RADIX SCUTELLARIAE	MOL002937	PDE3A
RADIX SCUTELLARIAE	MOL002937	ADRA1B
RADIX SCUTELLARIAE	MOL002937	ADRB2
RADIX SCUTELLARIAE	MOL002937	HSP90AA1
RADIX SCUTELLARIAE	MOL002937	PRKACA
RADIX SCUTELLARIAE	MOL002937	CAMKMT
RADIX SCUTELLARIAE	MOL002937	NCOA1
RADIX SCUTELLARIAE	MOL000358	PGR
RADIX SCUTELLARIAE	MOL000358	NCOA2
RADIX SCUTELLARIAE	MOL000358	PTGS1
RADIX SCUTELLARIAE	MOL000358	PTGS2
RADIX SCUTELLARIAE	MOL000358	HSP90AA1
RADIX SCUTELLARIAE	MOL000358	KCNH2
RADIX SCUTELLARIAE	MOL000358	PRKACA
RADIX SCUTELLARIAE	MOL000358	DRD1
RADIX SCUTELLARIAE	MOL000358	CHRM3
RADIX SCUTELLARIAE	MOL000358	CHRM1
RADIX SCUTELLARIAE	MOL000358	SCN5A
RADIX SCUTELLARIAE	MOL000358	CHRM4
RADIX SCUTELLARIAE	MOL000358	PDE3A
RADIX SCUTELLARIAE	MOL000358	ADRA1A
RADIX SCUTELLARIAE	MOL000358	CHRM2
RADIX SCUTELLARIAE	MOL000358	ADRA1B
RADIX SCUTELLARIAE	MOL000358	ADRB2
RADIX SCUTELLARIAE	MOL000358	CHRNA2
RADIX SCUTELLARIAE	MOL000358	SLC6A4
RADIX SCUTELLARIAE	MOL000358	OPRM1
RADIX SCUTELLARIAE	MOL000358	CHRNA7
RADIX SCUTELLARIAE	MOL000358	BCL2
RADIX SCUTELLARIAE	MOL000358	BAX
RADIX SCUTELLARIAE	MOL000358	CASP9
RADIX SCUTELLARIAE	MOL000358	JUN
RADIX SCUTELLARIAE	MOL000358	CASP3
RADIX SCUTELLARIAE	MOL000358	CASP8
RADIX SCUTELLARIAE	MOL000358	PRKCA

RADIX SCUTELLARIAE	MOL000358	PON1
RADIX SCUTELLARIAE	MOL000358	MAP2
RADIX SCUTELLARIAE	MOL000359	PGR
RADIX SCUTELLARIAE	MOL000359	NCOA2
RADIX SCUTELLARIAE	MOL000359	NR3C2
RADIX SCUTELLARIAE	MOL000525	NOS2
RADIX SCUTELLARIAE	MOL000525	PTGS1
RADIX SCUTELLARIAE	MOL000525	AR
RADIX SCUTELLARIAE	MOL000525	PPARG
RADIX SCUTELLARIAE	MOL000525	PTGS2
RADIX SCUTELLARIAE	MOL000525	PDE3A
RADIX SCUTELLARIAE	MOL000525	DPP4
RADIX SCUTELLARIAE	MOL000525	HSP90AA1
RADIX SCUTELLARIAE	MOL000525	CDK2
RADIX SCUTELLARIAE	MOL000525	CHEK1
RADIX SCUTELLARIAE	MOL000525	PRKACA
RADIX SCUTELLARIAE	MOL000552	NOS2
RADIX SCUTELLARIAE	MOL000552	PTGS1
RADIX SCUTELLARIAE	MOL000552	F2
RADIX SCUTELLARIAE	MOL000552	KCNH2
RADIX SCUTELLARIAE	MOL000552	AR
RADIX SCUTELLARIAE	MOL000552	SCN5A
RADIX SCUTELLARIAE	MOL000552	F10
RADIX SCUTELLARIAE	MOL000552	PTGS2
RADIX SCUTELLARIAE	MOL000552	F7
RADIX SCUTELLARIAE	MOL000552	TOP2A
RADIX SCUTELLARIAE	MOL000552	ESR2
RADIX SCUTELLARIAE	MOL000552	DPP4
RADIX SCUTELLARIAE	MOL000552	PPARD
RADIX SCUTELLARIAE	MOL000552	HSP90AA1
RADIX SCUTELLARIAE	MOL000552	PRSS1
RADIX SCUTELLARIAE	MOL000552	NCOA2
RADIX SCUTELLARIAE	MOL000552	CAMKMT
RADIX SCUTELLARIAE	MOL000552	KDR
RADIX SCUTELLARIAE	MOL000552	NCOA1
RADIX SCUTELLARIAE	MOL000073	PTGS1
RADIX SCUTELLARIAE	MOL000073	ESR1
RADIX SCUTELLARIAE	MOL000073	PTGS2
RADIX SCUTELLARIAE	MOL000073	HSP90AA1
RADIX SCUTELLARIAE	MOL000073	DPEP1
RADIX SCUTELLARIAE	MOL000073	PRKACA
RADIX SCUTELLARIAE	MOL000449	PGR
RADIX SCUTELLARIAE	MOL000449	NR3C2
RADIX SCUTELLARIAE	MOL000449	NCOA2
RADIX SCUTELLARIAE	MOL000449	NPPB
RADIX SCUTELLARIAE	MOL000449	IGHG1
RADIX SCUTELLARIAE	MOL000449	RXRA
RADIX SCUTELLARIAE	MOL000449	NCOA1
RADIX SCUTELLARIAE	MOL000449	PTGS1
RADIX SCUTELLARIAE	MOL000449	PTGS2
RADIX SCUTELLARIAE	MOL000449	ADRA2A
RADIX SCUTELLARIAE	MOL000449	SLC6A2
RADIX SCUTELLARIAE	MOL000449	SLC6A3
RADIX SCUTELLARIAE	MOL000449	ADRB2

RADIX SCUTELLARIAE	MOL000449	AKR1B1
RADIX SCUTELLARIAE	MOL000449	PLAU
RADIX SCUTELLARIAE	MOL000449	LTA4H
RADIX SCUTELLARIAE	MOL000449	MAOB
RADIX SCUTELLARIAE	MOL000449	MAOA
RADIX SCUTELLARIAE	MOL000449	PRKACA
RADIX SCUTELLARIAE	MOL000449	CTRB1
RADIX SCUTELLARIAE	MOL000449	CHRM3
RADIX SCUTELLARIAE	MOL000449	CHRM1
RADIX SCUTELLARIAE	MOL000449	ADRB1
RADIX SCUTELLARIAE	MOL000449	SCN5A
RADIX SCUTELLARIAE	MOL000449	ADRA1A
RADIX SCUTELLARIAE	MOL000449	CHRM2
RADIX SCUTELLARIAE	MOL000449	ADRA1B
RADIX SCUTELLARIAE	MOL000449	CHRNA7
RADIX SCUTELLARIAE	MOL001458	NOS2
RADIX SCUTELLARIAE	MOL001458	PTGS1
RADIX SCUTELLARIAE	MOL001458	KCNH2
RADIX SCUTELLARIAE	MOL001458	ESR1
RADIX SCUTELLARIAE	MOL001458	AR
RADIX SCUTELLARIAE	MOL001458	SCN5A
RADIX SCUTELLARIAE	MOL001458	PTGS2
RADIX SCUTELLARIAE	MOL001458	PRSS1
RADIX SCUTELLARIAE	MOL001490	SCN5A
RADIX SCUTELLARIAE	MOL002879	SCN5A
RADIX SCUTELLARIAE	MOL002879	ADRB2
RADIX SCUTELLARIAE	MOL002879	CHRM3
RADIX SCUTELLARIAE	MOL002897	NOS2
RADIX SCUTELLARIAE	MOL002897	KCNH2
RADIX SCUTELLARIAE	MOL002897	ESR1
RADIX SCUTELLARIAE	MOL002897	AR
RADIX SCUTELLARIAE	MOL002897	PTGS2
RADIX SCUTELLARIAE	MOL002897	RXRA
RADIX SCUTELLARIAE	MOL002897	PRKACA
RADIX SCUTELLARIAE	MOL002897	PRSS1
RADIX SCUTELLARIAE	MOL002897	NCOA2
RADIX SCUTELLARIAE	MOL002897	PDE10A
RADIX SCUTELLARIAE	MOL008206	NOS2
RADIX SCUTELLARIAE	MOL008206	PTGS1
RADIX SCUTELLARIAE	MOL008206	F2
RADIX SCUTELLARIAE	MOL008206	AR
RADIX SCUTELLARIAE	MOL008206	SCN5A
RADIX SCUTELLARIAE	MOL008206	PPARG
RADIX SCUTELLARIAE	MOL008206	PTGS2
RADIX SCUTELLARIAE	MOL008206	RXRA
RADIX SCUTELLARIAE	MOL008206	ESR2
RADIX SCUTELLARIAE	MOL008206	DPP4
RADIX SCUTELLARIAE	MOL008206	MAPK14
RADIX SCUTELLARIAE	MOL008206	GSK3B
RADIX SCUTELLARIAE	MOL008206	HSP90AA1
RADIX SCUTELLARIAE	MOL008206	CDK2
RADIX SCUTELLARIAE	MOL008206	CHEK1
RADIX SCUTELLARIAE	MOL008206	PRKACA
RADIX SCUTELLARIAE	MOL008206	PRSS1

RADIX SCUTELLARIAE	MOL008206	NCOA1
RADIX SCUTELLARIAE	MOL008206	CAMKMT
RADIX SCUTELLARIAE	MOL008206	ADRA1B
RADIX SCUTELLARIAE	MOL008206	ADRB2
RADIX SCUTELLARIAE	MOL008206	CHRNA7
RADIX SCUTELLARIAE	MOL010415	NCOA2
RADIX SCUTELLARIAE	MOL012245	PTGS1
RADIX SCUTELLARIAE	MOL012245	PTGS2
RADIX SCUTELLARIAE	MOL012245	CA2
RADIX SCUTELLARIAE	MOL012245	HSP90AA1
RADIX SCUTELLARIAE	MOL012245	PRKACA
RADIX SCUTELLARIAE	MOL012245	CAMKMT
RADIX SCUTELLARIAE	MOL012246	PTGS1
RADIX SCUTELLARIAE	MOL012246	PTGS2
RADIX SCUTELLARIAE	MOL012246	CA2
RADIX SCUTELLARIAE	MOL012246	HSP90AA1
RADIX SCUTELLARIAE	MOL012246	PRKACA
RADIX SCUTELLARIAE	MOL012266	NOS2
RADIX SCUTELLARIAE	MOL012266	PTGS1
RADIX SCUTELLARIAE	MOL012266	F2
RADIX SCUTELLARIAE	MOL012266	KCNH2
RADIX SCUTELLARIAE	MOL012266	AR
RADIX SCUTELLARIAE	MOL012266	SCN5A
RADIX SCUTELLARIAE	MOL012266	F10
RADIX SCUTELLARIAE	MOL012266	PTGS2
RADIX SCUTELLARIAE	MOL012266	CA2
RADIX SCUTELLARIAE	MOL012266	F7
RADIX SCUTELLARIAE	MOL012266	KDR
RADIX SCUTELLARIAE	MOL012266	RXRA
RADIX SCUTELLARIAE	MOL012266	TOP2A
RADIX SCUTELLARIAE	MOL012266	ESR2
RADIX SCUTELLARIAE	MOL012266	DPP4
RADIX SCUTELLARIAE	MOL012266	HSP90AA1
RADIX SCUTELLARIAE	MOL012266	PRSS1
RADIX SCUTELLARIAE	MOL012266	NCOA2
RADIX SCUTELLARIAE	MOL012266	NCOA1
RADIX SCUTELLARIAE	MOL012266	CAMKMT
REHMANNIAE RADIX PRAEPARATA	MOL000359	PGR
REHMANNIAE RADIX PRAEPARATA	MOL000359	NCOA2
REHMANNIAE RADIX PRAEPARATA	MOL000359	NR3C2
REHMANNIAE RADIX PRAEPARATA	MOL000449	PGR
REHMANNIAE RADIX PRAEPARATA	MOL000449	NR3C2
REHMANNIAE RADIX PRAEPARATA	MOL000449	NCOA2
REHMANNIAE RADIX PRAEPARATA	MOL000449	NPPB
REHMANNIAE RADIX PRAEPARATA	MOL000449	IGHG1
REHMANNIAE RADIX PRAEPARATA	MOL000449	RXRA
REHMANNIAE RADIX PRAEPARATA	MOL000449	NCOA1
REHMANNIAE RADIX PRAEPARATA	MOL000449	PTGS1
REHMANNIAE RADIX PRAEPARATA	MOL000449	PTGS2
REHMANNIAE RADIX PRAEPARATA	MOL000449	ADRA2A
REHMANNIAE RADIX PRAEPARATA	MOL000449	SLC6A2
REHMANNIAE RADIX PRAEPARATA	MOL000449	SLC6A3
REHMANNIAE RADIX PRAEPARATA	MOL000449	ADRB2
REHMANNIAE RADIX PRAEPARATA	MOL000449	AKR1B1

REHMANNIAE RADIX PRAEPARATA	MOL000449	PLAU
REHMANNIAE RADIX PRAEPARATA	MOL000449	LTA4H
REHMANNIAE RADIX PRAEPARATA	MOL000449	MAOB
REHMANNIAE RADIX PRAEPARATA	MOL000449	MAOA
REHMANNIAE RADIX PRAEPARATA	MOL000449	PRKACA
REHMANNIAE RADIX PRAEPARATA	MOL000449	CTRB1
REHMANNIAE RADIX PRAEPARATA	MOL000449	CHRM3
REHMANNIAE RADIX PRAEPARATA	MOL000449	CHRM1
REHMANNIAE RADIX PRAEPARATA	MOL000449	ADRB1
REHMANNIAE RADIX PRAEPARATA	MOL000449	SCN5A
REHMANNIAE RADIX PRAEPARATA	MOL000449	ADRA1A
REHMANNIAE RADIX PRAEPARATA	MOL000449	CHRM2
REHMANNIAE RADIX PRAEPARATA	MOL000449	ADRA1B
REHMANNIAE RADIX PRAEPARATA	MOL000449	CHRNA7
