# Supplementary material for: Identification of Bioactive Compounds and Potential Mechanisms of Kuntai Capsule in the Treatment of Polycystic Ovary Syndrome by Integrating Network Pharmacology and Bioinformatics
Source: Oxid Med Cell Longev. 2022 Apr 28;2022:3145938. doi: 10.1155/2022/3145938 (PMC9073551; doi:10.1155/2022/3145938)
Supplement: Supplementary 3 — Supplement Table 3: therapeutic targets of PCOS disease database and GEO database. [file 3145938.f3.pdf]

Genecards database	disgenet database	NCBI database	OMIM database	pharGKB database
PCOS1	CYP19A1	TNF	ACTG2	PCOS1
INS	AKR1C3	IL6	ADAR	CFTR
SHBG	STAR	ADIPOQ	AFF3	ESR1
ADIPOQ	BCL2	AR	AGRN	PKD3
FSHR	HSD3B1	TP53	AHDC1	UBE3A
FST	FSHR	VDR	AKT3	ESR2
AMH	LMNA	PPARG	ALG14	PGR
LEP	GNAS	VEGFA	ALMS1	PKD1P6
DENND1A	GDF9	MTHFR	ANAPC1	PKD1P5
PRL	BMP15	ACE	ANTXR1	PKD1P4
INSR	INS	CRP	ARID1A	PKD1P3
IGF1	LEP	ESR1	ASXL2	PKD1P2
PPARG	HSD3B2	TGFB1	ATAD3A	ST8
IRS1	LIF	SERPINE1	ATP13A2	PKHD1L1
RETN	LHB	PON1	B3GALT6	PKD1P1
CYP19A1	RBP4	IL1B	BBS5	PKD1L1
AR	MMP2	IL10	BCL10	OVCA2
GHRL	AZGP1	FTO	BCL11A	PKDREJ
CYP11A1	MTNR1B	INS	BOLA3	PKD2L2
GNRH1	SRD5A1	HIF1A	BSND	PKD1L2
CYP17A1	GAB1	MMP9	CALM2	PKD1L3
CAPN10	CGB5	PTEN	CASP10	PKD2L1
NAMPT	MALAT1	GHRL	CASP8	PKHD1
IGFBP1	AKT2	BDNF	CCDC28B	VEGFA
H6PD	AKR1C1	AKT1	CCDC88A	LEPR
CGA	CCNB1	IGF1	CD46	RENS2
CYP21A2	MANEA	CXCL8	CDC42	RSS
IRS2	ERRFI1	LEP	CDC73	PKD1
IGF2	CLDN4	RETN	CDCA7	MMP19
THADA	DMD	NOS3	CELA2A	DPH1
POR	PNPLA2	AGER	CENPF	PKD2
GH1	TFRC	IFNG	CEP104	FAM32A
SRD5A1	BAX	IL18	CFH	OCIAD2
GAS5	TNRC6B	CYP17A1	CFHR1	POF18
IGFBP3	RP2	NAMPT	CFHR3	DWS
GDF9	RAB2A	MDM2	CHN1	KSS
TCF7L2	MAPK8IP3	CCL2	CHRM3	LEP
LINC01828	FUT7	MMP2	CHRNA1	RLN2
ENSG00000234940	TMEFF2	NPPB	CKAP2L	PMPS
ENSG00000286145	IQCC	ICAM1	CLCNKA	CDL1
ENSG00000230704	FLT4	AMH	CLCNKB	CDL2
AFA1	LSM5	CYP1A1	CNNM4	MYSA
SERPINE1	RPL37A	IL17A	COL11A1	ICR4
STAR	RRM2	LCN2	COL3A1	THAS
CRP	MAPRE3	HMOX1	COL5A2	AIC
SULT2A1	PWWP2B	IL1RN	COL9A2	ACLS
HSD3B2	TCF15	ESR2	COLEC11	JBS
VEGFA	UBE2H	MIF	CXCR4	BOK
MSR1	AKR1C2	HMGB1	DCAF17	FGS2
IL6	TTLL9	FSHR	DCTN1	FGS3
MT-TL1	TMF1	TNFRSF11B	DDR2	TNR1
TNF	TOP2A	HFE	DDX59	MBS1
HSD11B1	LINC00475	SHBG	DGUOK	ADFN

FTO	PTCRA	INSR	DNMT3A	SCKL3
PON1	TBX1	HLA-G	DVL1	SRPSMCR
APOB	S100P	IL4	EIF2AK2	CAPN5
ACE	SCNN1A	IGF1R	EIF2AK3	WS2B
LHCGR	SCT	APOA1	EN1	RIEG2
RBP4	SFTPC	IRS1	ESPN	MROS
ADIPOR1	DUX4L5	LMNA	F5	DURS1
APOA1	ELAVL3	IGF2	FASLG	ANCR
PPARGC1A	SOX15	TCF7L2	FIGLA	CECR
SLC2A4	DLG4	CYP19A1	FLG2	GTS
MTHFR	TTK	MIR146A	FSHR	DCR
ESR2	LTK	UGT1A1	GATAD2B	WHCR
FBN3	IGHG1	LHCGR	GLI2	MSD
ADIPOR2	NMNAT3	BAX	GNAI3	DGCR
POMC	TONSL	APOB	GNB1	WBS2
AGER	ADGRD2	CXCL10	GPSM2	LCS1
ESR1	S100A7A	LPL	GRHL3	USH1E
GATA6	FOXP3	IL2	HAAO	USH1A
MC4R	PDE3B	WT1	HADHA	GPDS1
REN	HMMR	PCSK9	HFM1	DGCR7
PAEP	BCL11A	LEPR	HHAT	KERSMCR
AKR1C3	ATF1	IL1A	HOXD13	HTC1
VDR	MYBL1	PGR	HS2ST1	PRS
HSD3BP4	LIFR	RARRES2	HSPG2	NFXL1
HSD17B6	MAD2L1	FOXO3	IBA57	WBSCR2
AMHR2	BLOC1S3	PPARGC1A	IFIH1	SMCR9
IL1B	KRT3	DPP4	IL6R	SMCR3
IL18	SPATA21	VCAM1	IRF6	TRESMCR
SRD5A2	ZSWIM9	CD40LG	KCND3	SMCR6
IAPP	ODF3B	CAPN10	KCNH1	MCS
HP	TMEM151B	CHI3L1	KCNJ10	ATD
DPP4	ISYNA1	TNFSF11	KCNN3	DGCR12
PPARD	NRG1	PIK3R1	KIF14	EEC1
PLA2G7	HELLS	CNR1	KIRREL1	EEC2
BMP15	ANLN	TIMP1	KYNU	ERMP1
MIF	PTGER3	GC	LBR	FILIP1L
CCL2	PLEKHG5	ENG	LMNA	DGCR6L
PBX1	REXO1	STK11	LMOD1	MBS3
LEPR	ARHGAP23	HNF1A	LORICRIN	MBS2
F5	SPTBN4	RUNX2	LRIG2	MEHMO
FNDC5	ASPM	MALAT1	LRP2	ADAMTS1
IGFBP2	PRKD2	PTX3	LYST	SMCR2
ANGPTL8	ARHGAP30	ITLN1	MDM4	NOBOX
CHIT1	NPB	FBN1	MDS2	OCIAD1
SERPINA12	PTAFR	DICER1	MPV17	MRXS7
ICAM1	EMSY	KLK3	MPZ	MRXS11
ADAMTS1	TNPO2	HMGA2	MSH2	IMMP2L
PTEN	NOP53	KL	MSH6	GALNS
KLK3	RACGAP1	ANGPTL8	MTOR	DSCR4
ADRB3	LPCAT2	GH1	MTX2	MSH4
INSIG2	CEP55	MC4R	MYCN	PCYT1B
MAP3K2-DT	TRPV6	NGF	MYSM1	CECR3
FAS	GP1BB	MIR27A	NBAS	SOHLH1
PPARA	CMTR2	KISS1	NECTIN4	STRA8

FSHB	PBK	KCNJ11	NFU1	CECR9
STK11	TMEM151A	RBP4	NLRC4	MDS2
RUNX2	ABCB6	ADM	NLRP3	KALP
MIR21	C12orf66	UCP2	NOS1AP	AFP
MIR323A	ZMYM5	IL6R	NOTCH2	FANCG
GNAS	RHPN1	SERPINF1	NPHP1	NO53
ALDH1A3	CAMK2D	TIMP2	NPHP4	KDR
CXCL8	TEAD2	SREBF1	NPHS2	DGCR9
LEPQTL1	CKS2	WNT5A	NRAS	FSHB
AKT2	CNTROB	FNDC5	NRXN1	SDCCAG8
PYY	MGAT5B	HSD11B1	NUP133	CECR7
GCG	ADAMTS7	AHSG	ODC1	DGCR11
AKT1	PDLIM4	SOCS1	ORC1	DGCR10
PCSK9	HACD1	APLN	ORC4	KITLG
KISS1	SRSF10	AVP	PADI3	RIPPLY3
MMP9	RUNX3	GHR	PBX1	USHBP1
GIP	ADARB1	BGLAP	PER3	PKP1
ADRB2	FGF18	PLAT	PHGDH	PRG4
MIR135A1	CCNE2	SAA1	PIGF	RCC1L
SREBF1	ZNF205	CX3CL1	PIGV	GTSCR1
GSTM1	DDX54	CPB2	PIK3CD	MIEF1
KISS1R	RASL10B	NPY	PKP1	METTL27
MIR222	SAP30L	CASP9	PLOD1	ANOS1
MIR320A	LARP4	PRL	POGZ	INHA
HSD11B2	CDC6	EPHX1	POLR1B	TCTN2
ANXA5	TAB1	SRD5A2	POU3F3	WASL
PTX3	BBOF1	SLC2A4	PPP1CB	DSCR10
LOC108964933	JSRP1	ADIPOR1	PREPL	EVC2
UCP2	ZNF430	ADA	PRG4	HYLS1
ITLN1	MORN1	GZMB	PSMB4	DGCR6
ADA	FBXO44	ENPP1	PTCH2	ZNRD2
IDE	CARMIL3	GLP1R	RAB3GAP1	HDHD5
IL17A	DCBLD2	IRS2	RAB3GAP2	HPS3
MIR145	DACT3	IGFBP1	RBM8A	BCS1L
HMGB1	CDH15	CRH	RC3H1	BCL2L1
CGB5	HOOK3	CCN1	RFX5	ANG
NR1H3	LRATD1	MIR203A	RIT1	NHS
MUC1	THAP3	MIR223	RNU4ATAC	ANGPT1
MIR146A	IL34	HDAC3	RYR2	EREG
MIR27A	PGR	MSTN	SATB2	EVC
MIR483	RARRES2	CYP11A1	SCN1A	BBS12
MAPK3	HSD17B7	NR1H3	SDCCAG8	FOXC1
GPER1	NR3C1	DCN	SF3B1	SSNA1
INSL3	ADRB2	LOX	SKI	OCRL
CYP1A1	CD36	INHBA	SLC19A2	CRLF1
GPT	ADRB3	AKT2	SLC25A24	TBL2
MIR486-1	NCOR1	ANGPTL4	SLC2A1	AMMECR1
HOXA10	TNFSF10	NUCB2	SLC5A7	WBSCR23
MIR23A	TH	MTNR1B	SLC9A1	SAMHD1
MIR486-2	DENND1A	MIR222	SOS1	INHBA
INHA	INSR	TNFRSF11A	SOX11	ARVCF
MIR23B	SLPI	NR4A1	SPEN	DSCR8
WT1	THADA	FAAH	SPRTN	NPHP3
PEPD	NGFR	MMP8	ST3GAL5	WHSC1L2P

GPX3	ADRA1D	FABP1	STAMBP	HPRT1
MIR155	ADRA1A	XPA	SUCLG1	AMH
PWRN2	LHCGR	HSP90B1	SYT2	VPS26C
RARRES2	ADRA1B	CSF3	TBCE	LFNG
TLR2	ATP5F1B	INHA	TBX15	DMC1
GRP	PPARG	HMGCR	TCHH	PISRT1
ADO	YAP1	MIR142	TET3	SMCR5
NOTCH3	PTEN	ARF6	TGFB2	VGF
ITGAX	AOPEP	PEPD	TMCO1	XK
MIR30C1	HNF1A	UGT2B7	TMEM237	SMCR8
MIR30C2	ATM	TFAP2A	TPRKB	FGFR2
IGF1R	ERBB4	SLC30A8	TTC7A	ACSBG1
BRCA2	LIPE	NCOR1	USH2A	EIF2B2
BRCA1	HMGA2	VCAN	VANGL1	AGT
TP53	VCAN	GATA6	WDPCP	EIF2B4
PKHD1	CBX2	MIR125B1	WDR26	INHBB
ATM	FOS	HOXA10	WIPF1	NRIP1
MSH2	CAV1	SMAD1	WNT4	CCND2
MSH6	SETD2	FST	YY1AP1	DGCR5
TSC2	PLIN1	FADS1	ZEB2	C8orf37
CEP290	ERBB3	ARNTL		FOXL2
FBN1	SERPINE1	AKR1C3		TAF4
PIK3CA	AGPAT2	SREBF2		EIF2B5
CDH1	IGF1	ACAN		IPW
RAD51D	BAZ1B	ADAMTS1		CEP290
RAD51C	AMH	IDE		TMEM121B
PALB2	ANTXR2	ADIPOR2		PLA2G4A
CHEK2	MAP3K1	AZGP1		ZMPSTE24
NBN	DHH	IGF2BP2		MIEF2
BRAF	IRS2	PAEP		RNASEH2C
MLH1	MTHFR	FADS2		MMP14
KRAS	VCAN-AS1	CCK		CCNE1
BRIP1	TBL2	HCRT		SBDSP1
PTCH1	CRB1	MTNR1A		SSB
PMS2	RFC2	MIR23A		DSCR9
CC2D2A	FST	SLC22A1		TMEM270
FMR1	TICRR	ACVR1		FGF7
APC	PRL	GAB1		EDN2
CTNNB1	FLT1	INSL3		BMPR1B
NF1	IL6	PYY		FRAS1
DICER1	CDK2	TGFBR3		SUGCT
ERCC6	FBN3	FGF19		ESS2
FGFR2	RETN	PRKAA2		SPART
RAD50	CAPN10	GDF9		CECR2
C11orf65	CORIN	KISS1R		BBS10
OFD1	LINC02055	PNPLA2		NSUN5P1
MKS1	GTF2I	TRIB3		GALNT17
BLM	LMNB2	SLC18A2		SIRT1
MUTYH	GCG	SMAD5		NIPA1
GANAB	STON1-GTF2A1L	LUM		GTF2H5
BARD1	CCDC91	GPX3		MKS1
KCNQ1	POR	BMP15		TKT
SEC63	IL1B	ITGAX		MID1
SMAD4	CAVIN1	APLNR		BSND

TMEM67	BSCL2	INHBB	PCNT
DYNC2H1	NR5A1	LHB	IDS
ERBB2	PON1	LIPE	BUD23
NPHP3	GABRB1	GIP	MLF1
PRKCSH	POMC	MT-TL1	SOD1
FOXL2	IL1A	SFRP4	CC2D2A
LMNA	BRD2	MIR23B	ITSN1
JAK2	SULT2A1	HAS2	USH1G
RPGRIP1L	ELN	THADA	HPS5
FLNA	SOX5	GJA4	CLDN4
TSC1	TGFB2	UGT2B15	CFM2
BBS1	SOX3	NRIP1	CFM1
HNF1B	KIF7	SRA1	DSCAM
MRE11	FTO	LEPQTL1	PANK2
MECP2	TNF	CYP2R1	FBN1
LRP5	CYP17A1	SRD5A1	HPS6
SDHD	ESR1	DENND1A	TP53
ALG9	CYP2B6	PRDX4	CFTRP1
NR5A1	SLC2A4	MIR130B	KCNQ1
BBS10	ACE	MIR486-1	ETV2
COL4A5	SOX9	SGTA	BBSS
TP63	DMRT1	MIR483	USH1C
BBS4	CRP	SFRP5	CD177
PTPN11	ZBTB16	MIR423	DCAF17
ALMS1	SRY	H6PD	ETV3L
HRAS	WT1	MIR125B2	CSTF2T
PMM2	TCF7L2	HOXA11	NIPA2
RET	NR0B1	MIR19B1	SBDS
EGFR	ALMS1	MIR30C1	SPG21
STAG3	GTF2IRD1	AMHR2	BAZ1B
MKKS	CLIP2	LPP	ETV6
FRAXA	VEGFA	APPL1	ETV5
ATRX	LIMK1	ADAMTS9	USH2A
TGFBR2	CYP11A1	GYS1	DGCR2
SDHB	VDR	ZNF217	ALG3
RAF1	IKZF3	AQP8	DSCAML1
TMEM216	ADIPOQ	HAS1	TCOF1
FGFR3	CIDEC	ENHO	BBS9
MIR17	MMP14	CCN5	ELN
STAT3	IRS1	BANCR	ADA2
BBS2	MAPRE1	MIR33B	TRPS1
NOTCH1	MSX1	IL18BP	NSD3
XRCC2	AR	WNT2B	CLCF1
BBS12	IRF1-AS1	EPHA7	BBS7
POLE	STOX1	GREB1	HPS4
MTOR	PMEL	NFIC	WRN
POLG	SHBG	HAS3	ETV3
TMEM231	ANGPTL8	MIR30C2	NSUN5P2
MIR15A	DPP4	RAD54B	ETV4
AHI1	AMHR2	STK26	SCHIP1
VHL	KISS1	FBN3	SCNN1B
MAP3K1	IGF2	MIR323A	ETV1
FGFR1	TGFB1	INHBC	PSAP
NUP107	GLP1R	OSGIN1	MECP2

BMPR1A	CYP21A2	MGAM	WAS
NSD1	HSD11B1	PRKAG3	ETV7
ALB	LEPR	ZP4	EBP
CDKN1C	GHRL	PDE8A	OFD1
GLI3	IL10	HSD17B6	BLOC1S3
NPHP1	CXCL8	MIOX	PYGM
GALT	H6PD	FEM1B	HPS1
COL3A1	LDLR	C1QTNF12	ERCC5
TTC21B	FSHB	FEM1A	ATRX
BBS5	IL18	C1QL3	BBS2
IL10	ESR2	MIR486-2	PEX2
IFT140	PPARGC1A	PWRN2	USP10
NOTCH2	PLG	MCF2L2	ALMS1
TCTN2	PIK3CG	NEWENTRY	NSD2
WDR19	CYP1A1	YAP1	WFS1
BBS9	ADIPOR1	APOE	NDN
RAD51	PIK3CD	STAT3	SCN5A
NPHS1	AGRP	HLA-DRB1	BCL2
BBS7	PIK3CB	COMT	BMP4
RB1	ARTN	CD274	TAZ
SOX9	PIK3CA	PCOS1	CD40LG
SUFU	MIR93	NR3C1	TWIST1
SLC2A1	APOA1	GSTM1	BBS1
GJB2	APOB	MIR21	PRNP
PRKAR1A	ICAM1	CD4	GOPC
MITF	GNRHR	ADRB2	CYLD
MIR200B	ADAMTS1	CDKN1A	MKKS
MIR200A	SLC2A1	GSTT1	RPGRIP1L
MIR223	EGF	TLR2	CD74
MEN1	ALB	F2	EEGV1
TGFBR1	CASP3	ATM	BVR1
COL5A1	KLK3	DRD2	RNVU1-11
COL2A1	IL1RN	F5	RNVU1-1
EPCAM	MMP9	GSK3B	RNVU1-7
COL4A3	MC4R	HLA-DQB1	RNU6V
JAG1	MAPK3	SPP1	UBE2V1P1
TGFB2	PDCD4	LGALS3	ERCC6
CDKN1B	FOXO1	AGT	RNVU1-18
SRY	PTH	PDCD1	GLRA1
AIRE	ACTB	EDN1	PF4V1
MAPK1	HSD17B6	SOD2	BBS4
TGFB1	AKT1	MAPK3	TAGLN
UBE3A	F5	CDKN1B	C1QTNF5
POF1B	PCOS1	AGTR1	BLM
FRAS1	CAT	MIR155	CALR
NPHP4	KISS1R	LDLR	MFRP
NRAS	ADIPOR2	ABCA1	FGFR1
ZEB2	MTOR	CYP21A2	KIF17
MIR200C	LPA	HBA1	UBE2V2
CHD7	ENPP1	EGF	ARMC10
NR0B1	STK11	E2F1	THPO
SLC26A4	STS	FOXO1	CLN6
FANCC	CHST3	IGFBP3	UBE2V1
SGO2	GSK3B	C3	DFNM1

WFS1	NGF	HP	CC2D1A
SDCCAG8	PPARA	KCNQ1	BIRC5
COL4A4	HSD17B1	PLAUR	SLC1A3
CPLANE1	GC	TOX3	NOX1
B9D1	GATA6	IL33	
PAX3	INHA	HSPB1	
FLCN	SMAD2	HAMP	
PAX2	HSD17B2	S100A9	
NOBOX	NUCB2	PLA2G7	
IFT172	MIR27A	NRG1	
UGT1A1	MIR155	NQO1	
MET	SMAD3	GDF15	
WDPCP	LCN2	CD36	
HSPG2	APOE	H19	
ARL13B	ZGLP1	NOTCH3	
MT-CYB	IARS1	TNFRSF1B	
MYC	ENHO	TRPV1	
GJA1	KCNJ11	GNB3	
EGF	IFNG	FABP4	
MIR214	MIR223	IAPP	
MRPS22	ITGAX	SUOX	
MMP2	IGFBP1	POSTN	
PDGFRA	STAT3	ERBB4	
BNC1	MIR486-1	FGF21	
PRKCD	MAPK8	PTH	
SOS1	AGTR2	F13A1	
PREPL	CFTR	CD28	
TYROBP	PRKAA2	PKD1	
TTC8	KRR1	TNFRSF10B	
MIR31	DKK1	PECAM1	
NRXN1	GPT	GPER1	
MIR210	EHMT1	MIR29A	
CASP10	CNR1	CD163	
MIRLET7I	ROS1	POMC	
KIF7	AGT	SERPINA12	
HSD17B4	PLA2G7	IL23A	
NIPBL	SIRT1	TGFB2	
SMAD3	PCSK9	WWTR1	
EDN1	PROK1	ANXA5	
COL5A2	ZNF410	REN	
MT-ATP6	HAMP	GGT1	
SETD2	FN1	GNAQ	
THBD	PTGS2	NR5A1	
COL1A1	BGLAP	PDCD4	
CSPP1	FAAH	RAB5B	
MUC16	SOD2	TFAM	
KIT	SOCS3	IL6ST	
CHRNE	VCAM1	IL32	
LRRC56	ACACA	ATF4	
C3	BMP6	IL1R1	
SDHC	CCL2	PRF1	
TGFB3	HLA-DRB1	TNFSF12	
MSH3	UCP2	HSD11B2	
CDK4	TP53	MIR93	

CCND1	CPA1	LIF
DNAJB11	ADA	GAS5
CDKN1A	FBN1	STON1-GTF2A1L
IQCB1	HSD11B2	RAB5A
PIK3R1	APLN	TXNIP
MDM2	EGFR	GNRHR
EIF2B2	CCND2	PON2
FIGLA	HOXA10	XDH
PAX6	HMGB1	XIST
TCTN1	MECP2	ESM1
CHRND	BMPR2	APOC1
LIG4	SMAD7	PRLR
H19	MIR200C	APOA4
MIR429	SRD5A2	SET
MMP14	SPARC	ZFP36L2
EDNRB	SPP1	CHIT1
FREM2	SREBF1	HHEX
APOE	ZNF217	GREM1
ATR	SUOX	CDKAL1
IFT88	WNT3A	HSD17B1
AKT3	TBC1D4	STS
NOS3	TPD52	ADRA2B
TBX5	TPM2	STAR
POLD1	MIR21	MIR320A
SMC1A	CAPN5	STC1
PMS1	MIR204	PAFAH1B1
CASP3	VIM	UTS2
ARID1B	MIR146A	MIR335
CASP8	MIR145	SULT2A1
INVS	VIP	LPIN1
CD40LG	MIR222	MIR27B
TMEM107	TAC3	PLIN1
ERCC4	MIR34A	HSD3B1
DGCR2	MIR320A	CYP11B1
PSMC3IP	CD44	SLC22A2
ARL6	PIK3R1	LBP
KLLN	MIR33B	KIR3DS1
CFI	MIR92B	MC3R
HDAC8	WNT2B	CYP3A7
GPC3	TGFBR1	ATP5F1B
DNMT3A	TLR4	FSHB
ELN	MIR141	ANGPTL3
TYMP	MEF2A	KIR2DS4
COMT	PAPSS2	ANGPTL2
FOS	PAEP	SH2B3
ACTB	PTPA	AQP9
MT-ND1	NPR2	GTF2A1L
LRP2	NPPC	MIR99A
TCTN3	NOTCH1	CARTPT
DSP	FEM1A	FERMT3
IFT80	PRKAA1	MIR135A1
ESCO2	PRKAB1	PRKAR2B
SIX1	AVP	EDN2
GATA3	SQSTM1	HSD17B2

HIF1A
FH
MT-CO1
BDNF
DROSHA
DIAPH2
PGR
CIITA
KCNJ5
B9D2
BMP4
SST
AFP
CHRNA1
FGF2
ANOS1
ALG8
TRAF3IP1
HLA-B
SATB2
PKD1L1
VIM
TMEM138
FAT4
JUN
UGT1A6
SHH
ERBB3
SOX3
CDC73
CDH2
HPRT1
EDNRA
CHRNB1
SCN1A
CCND2
BUB1B
RAB3GAP2
PTGS2
UGT1A8
CFTR
SMC3
MSX1
HAX1
ZNF423
LIMK1
MYH8
WNT5A
MLX
SALL1
BCL2L1
TTR
GAPDH
GLI1
CDKAL1
PKD1
PLAT
PCSK1
PCNA
TMED7
PLXNA2
IL22
SERPINB2
NOS3
BAAT
PSMD9
MIR323A
S100A6
SET
SFRP4
SGTA
CGB8
KITLG
MFAP1
MIR324
COX2
REN
RUNX2
ATF4
MSC
PTX3
MOK
RNR1
RB1
MTNR1A
MUC1
TOX3
NOC2L
INHBA
ELANE
DCTN6
GATA4
RBM45
EDN1
AGTR1
COMT
GPX1
HSPA1A
ZNRD2
PRDX4
RNU1-1
ADM
GPX3
HSPB1
EPHX1
IAPP
INSL3
AGER
SERPINA4
COPA
PLIN3
MIR574
MMP26
C1QTNF3
MIR103A1
AQP7
TACR3
TOR2A
TBC1D4
BCO1
AKAP8
SGK3
MIR136
SORBS1
AOPEP
FGF13
AFM
COL8A1
CAPN5
GPC4
MEP1A
UCN3
ANGPTL6
KLF7
MIR760
SLC5A3
MIR151A
SORCS1
SUMO1P1
ANGPTL1
INSL5
METRNL
USP34
MIR592
LEAP2
HSD3B7
MIR1294
TM4SF4
NENF
MIR518C
CTBP1-AS
LNCSRLR
LINC02402
LNCOC1

AIP	GH1
CYLD	H3P23
TH	TICAM2
NEK8	APRT
SRC	ACAN
CTLA4	AQP8
ESS2	CYP1B1
WNT4	IFI27
GHR	DCN
CFB	AQP7
CAV1	MSTN
BCL2	CGA
CHRNG	INHBB
B2M	CGB3
CSF3	GPR83
SERPINA1	IL17A
ADAMTS13	DBP
DCTN1	KRT16
AGK	RHOG
BAX	FCGR1B
ENG	TMED7-TICAM2
TBX3	ANGPTL2
RUNX1	HIF1A
ALDH3A2	HP
UGT1A9	RAD50
GDF6	AKR1B1
CXCL12	NR4A1
TNXB	LINC01672
DES	FCGR1A
VCAN	HHEX
HNF4A	FKBP4
AFF2	HLA-A
DVL1	MMRN1
NDUFAF2	LPL
RASA1	AQP9
IL2	CCN2
IFNG	HTC2
CEP57	HOTAIR
KRT7	LOX
SNAP29	FCGR1CP
ZMPSTE24	FGF2
AOPEP	FABP4
MPV17	FABP1
KDR	F3
GATA4	LPIN1
SHANK3	THBS1
NSD2	ECI1
NKX2-5	DECR1
H2AC18	TFAM
ATRIP	TFAP2A
BSCL2	SLC47A2
IL1A	CTF1
CXCR4	TLE5
IDH1	DAPK1

GNRHR	DDIT3
PLCG2	TIMP1
PIK3R2	DBH
PAX8	CRISP2
STIM1	C3
BAZ1B	TPT1
ANTXR2	ANGPTL6
PROKR2	CYP51A1
CP	TNFAIP3
FLNB	TNFRSF1B
SEC23B	CYP3A7
TFAP2A	TOP1
NOG	CYP11B1
FLT1	CYC1
TET2	TM4SF4
PROM1	TIMP3
MIR221	TIMP4
GTF2IRD1	TLR2
COL4A1	CTLA4
MYCN	CTNNB1
PSMB8	TPM1
NAGLU	CTRL
HSPB1	ADRA2B
SPP1	CYBB
DYRK1A	SLC35G1
CLU	SLC30A8
MYHAS	S100A1
NR3C1	GREM2
FGF8	FBLN1
HLA-A	F13A1
CEP104	BHMT
FN1	F2
CSF1	BMP4
HLA-DRB1	SLC5A3
GLI2	SLC6A2
GTF2I	NCF1
DYNC2I1	EREG
NEK9	ERCC2
IFT27	ARHGAP9
RAB3GAP1	FBRS
MT-ATP8	SFRP5
SLC2A2	S100A4
AAAS	S100B
AGTR1	TSPAN31
AGT	SCD
FBLN1	NLRP1
RAD21	FGG
GRIN2B	FGF13
RAG1	BLK
ABL1	SELENOP
CASR	ALDH1A3
HNF1A	FAT1
MYO5A	BMPR1A
KITLG	SLC18A2

PRKD1	SLC22A1
NTHL1	DOCK1
TLR4	STC1
ETS1	SULT1E1
PAFAH1B1	DNMT3B
SLC52A2	DNMT1
ITPR1	TAC1
FANCA	TACR3
WNT10A	AFM
BMP6	NQO1
NDUFS4	DHCR7
FGF10	MIR592
RAB23	DRD3
FGF9	PRSS55
FOXC2	ATN1
YAP1	SLC22A3
FOXE1	EPO
C8orf37	SNAI1
ENPP1	EPHB2
DGUOK	EPHA7
EZR	AHSG
DCAF17	EGR3
STAT5B	EDN2
F2	ABCA1
FGF23	SREBF2
TTN	SRF
AHSG	ADAMTS19
MYH9	BEST1
KRT19	PEA15
MAX	HSPB3
NKX2-1	SELENBP1
ADCY6	ACVR1
HMGA2	SORBS1
GSTP1	UCN2
CALB2	CECR
MMP1	CIB1
DHX37	CAP1
ALK	CDX2
MVK	ANGPTL1
BIRC5	CCT
CYP1B1	STUB1
CDH3	CBFA2T2
EDAR	POSTN
CDC45	IGF2BP2
COQ8B	TNFSF12
CLIP2	TCFL5
CYP11B1	CBS
EIF2B4	FGF19
MSLN	FADD
IL1RN	TNFRSF10B
CSF2	POLD3
ITGB3	CCN4
NFKB1	HDAC3
HDAC9	CCK

NF2	ARHGEF7
ERCC3	KHDRBS1
PRKCA	KHDRBS3
E2F1	CBSL
AQP2	SLC33A1
PPP1CB	BANCR
CDC25A	ROCK2
EPO	NR1H3
NGF	MIR3135B
HERPUD1	INSL5
SCNN1A	CD24
PLAU	CD59
SUCLG1	CD68
PARP1	GREB1
THBS1	CD69
SPIDR	USP34
RPGRIP1	PCLAF
CD2AP	BMS1
EIF2B5	TNFRSF8
CSF1R	FADS2
TGFA	ACVR2A
KIF3A	AKAP8
ALPP	CDKN1B
HUWE1	CDKN1A
PIK3CG	IL32
TTC21B-AS1	DHRS9
PACS1	ACVR2B
WASL	NAMPT
LAMA3	KL
POLR3H	FEM1B
CTNNA2	FTMT
CEP164	SFXN1
PTH	PGR-AS1
MAOA	ELMO1
CCR6	PRDX3
TFRC	SERPINA12
CTSD	CPB2
LCN2	COX8A
HSPD1	DSCC1
TIMP1	PWRN2
HGF	DHRS11
HOXD13	LAP
GPC4	PPARGC1B
WFDC2	CALCA
MIR19A	SLCO6A1
TBL1XR1	MAP3K8
MIR199A1	FZD3
SP1	COPA
CD274	SVEP1
USF3	CPT1A
DGCR8	AIR
F3	WFS1
LOX	WNT1
ABCC1	WNT5A

NSDHL	WNT8B
SCT	TMEM120B
MIR143	OR2AG1
UFD1	XK
IGF2R	XRCC1
SLC12A2	YWHAZ
MIR17HG	MKRN3
CRH	CSF3
CTSB	CREBBP
GRN	CPT2
GREM1	TMEM18
CD34	FLAD1
MIR9-1	CYP2R1
PIBF1	TADA1
IL6ST	LEAP2
FBN2	CASR
IL4	SLC5A11
FANCD2	SPZ1
CD27	C1QTNF3
GDNF	SORCS1
AGPAT2	GABARAP
SIX5	ATAD1
ATP6V0A2	LINC02605
NUP93	CHM
ALX4	INSL6
CDKN3	NR0B2
PIK3C2A	CASP9
CDK2	NCOA4
HDAC1	PNPLA3
DMRT3	FGF23
MIR4713HG	DRAM2
PIRC66	MAP1LC3B
ILK	CNP
TNFAIP3	NCOA3
SELE	NRIP1
SYP	SPPL3
MLXIPL	MRO
TXNDC15	IMMP2L
BUB1	CASP7
TBL2	BCAR3
COL7A1	GLIPR1
AQP1	BDNF
AXIN1	MMP1
VCAM1	MUC5AC
GNAQ	MUSK
PTHLH	FAS
CLDN4	SERPINC1
NFKB2	ATD
LBR	NBN
COQ2	HCG26
DCLRE1C	NFIC
HFE	CCN1
TEK	IGFBP6
TKT	IGFBP4

GGT1	NHS
AURKA	NNMT
RPS6KB1	IL1R1
TUBA1A	IL2
PDE6D	ITGA2B
RFC2	ISG20
HBA2	MPG
VWF	MPO
FAN1	IRF1
DMRT1	MIR151A
SLC6A4	CXCL10
MAGI2	MIR335
POU5F1	MT1L
KIAA0319L	IL11
MIR106B	IL7
NDUFS8	IL2RA
PHB	MTR
FEN1	NOS2
FKBP10	IGFBP2
TFAP2B	HSD17B13
SMARCC2	HSP90AA1
DGCR5	HSPB2
PTK2	INSIG2
CCN2	DCTN4
OCIAD1	HSPA9
LIPE	HSPA5
SERPINC1	HSPA4
FBXW7	HSPA1B
SLC34A1	PDE8A
RBPJ	PDGFRA
HLA-DQB1	IL23A
BMP2	GDE1
NPPA	PDK1
CHEK1	DNAJB1
SHOX	TAS2R13
APP	NOTCH3
ADCY10	NPPB
HDAC4	NANOS3
MIR182	ROR1
ARMC5	P2RY2
CD4	IDE
TIMP2	PAFAH1B1
PRDM5	PRDX1
RHO	APOC1
LAMA5	TNC
FGF7	HTR2C
C4A	PAPPA
HMOX1	PAX6
MIR126	PTRH2
MIR141	LUM
CASP9	FADS1
MIR29A	LGALS3
GLIS2	LCT
STAT1	MIR224

MIR140	MIR23A
PSEN1	MIR23B
NCAM1	MIR25
PIK3CD	MIR26B
HSPA5	SUMO1P1
NODAL	MIR28
MIR22	MIR29A
PAPPA	MIR29B1
KL	MIR29B2
IFT20	MIR203A
QRICH1	MIR200B
ABCC8	LYN
HDAC6	MIR106B
WWTR1	MIR130A
LMNB2	MIR130B
ABCG2	ARNTL
PDSS2	MIR142
AGGF1	CIMT
MAPK14	LRP5
CEP250	LPP
MMP7	MIR181A2
PALLD	MIR182
KARS1	MIR186
CST3	MIR19B1
CRYAA	MIR30C1
VEGFC	MIR30C2
PSMB4	LALBA
TUBB	GSTK1
BMPR1B	MCM2
RPGR	MCM7
PRLR	MDM2
SERPINA3	JUN
ERBB4	JAK2
SMAD2	ITK
FLI1	MAP3K4
NQO1	MEP1A
CTTN	ITGB5
AFF4	MELTF
MYH11	ITGB3
MAPK8	MIF
SMARCAL1	MC3R
SMAD7	ARSD
HSP90AA1	C1QL3
GSK3B	MAP3K15
PROK2	MIR99A
SLC6A3	TNFSF12-TNFSF13
DRD2	MAA
TFAM	LIN28B
SPARC	SMAD1
NLGN3	C1QTNF12
PLA2G6	SMAD4
MIR93	SMAD6
TPO	KNG1
LHB	MAGEA11

TOP2A	AREG
APOH	MIP
PCNA	HSD17B4
CFLAR	SLC2A4RG
TNFRSF10B	MIR409
LRPPRC	MIR491
SLC9A1	SERBP1
MUC5AC	ARID1B
SOD1	SEMA6A
RINT1	CFAP97
NBAS	PRPF31
HLA-G	APPL1
SGPL1	GAPDH
ATP2B3	TRIB3
CHGA	RAD54B
TNFRSF1B	ZP4
PTGS1	NEGR1
SLC25A1	MIR451A
MNX1	GATA1
CLDN3	C2orf83
NRG1	FEM1C
LRBA	GGT1
CXCL10	PYY
EIF2AK3	CHPT1
CUX1	ADAMTS9
IFT122	GREM1
DIABLO	GCKR
PLAT	GCK
PER2	PTBP1
ITGA6	FGF21
IFT57	PTGDR
ZFPM2	FBXO8
HOTAIR	ACTA1
TNNT2	SLC37A4
PDE11A	G6PC
MALAT1	FLNA
TUBB3	RNF6
SYNGAP1	RNU1-4
IFT52	GAS5
ADD1	ROCK1
KRT14	TAB2
PLAUR	MCF2L2
MIR142	RPL3
PLIN1	RPL36A
BGLAP	MIR483
CCNE1	FOXO3
INHBA	RPS6KB1
CYP11B2	RPS26
PLEK	FMR1
EFEMP1	RENBP
CACNA1H	PTBP2
NCOA3	NEIL2
SCARF2	RAB5B
MT-TT	RAC1

SOX11	FSHMD1A
CALCA	RAPSN
LGALS3	SGK3
SMAD6	RASA1
CD79A	NUP62
PTH1R	LGR6
PAK4	DICER1
ITGAV	BCL2A1
CDT1	PPP1R13B
HSD3B1	ATF6
LEMD3	PDPK1
XPA	HLA-DQA1
MIR25	BCO1
ARHGAP31	PRKAG3
IL6R	PNN
TBC1D20	NANS
RREB1	ANXA5
ECE1	WNT4
GAD1	HAS3
ITGB1	HAS2
CXCL1	HAS1
MRPS34	PON2
UMOD	GYS2
USP8	CCHCR1
CEP55	PLAUR
ST3GAL5	HMGB2
ITCH	APEX1
AIFM1	ARID4B
MIR30A	SERPINF1
SCN9A	STK26
CD8A	ADA2
LEF1	PF4
NRP1	HRAS
FAM20A	PHB
HSPA4	HNF4A
YBX1	HMOX1
HSPB2	MIR30A
CLUAP1	PIK3R2
PRODH	HMGCR
SOCS3	GTF2H1
HOXA11	PPARD
MIR335	GSTT1
VANGL2	TOR2A
ACACA	SGSM3
NTS	PRKAR2B
SYK	DESI1
XIST	HPGDS
MIR125B1	DMAP1
SRA1	MAPK1
HPSE	AGO2
COQ6	MAPK7
NR2F1	GIP
SLPI	GHR
LIF	PRLR

HADHA
CLTC
GCK
CD28
HSD17B1
NPPB
SIM1
DNAH11
GNB3
DDX59
GSTT1
IFI27
MIR92A1
IL7
CAVIN1
NDUFB11
CREB1
TSHR
LPL
GABBR2
TIMP3
DHH
TRPV1
CCNA2
MIR99A
ABCA4
MC2R
SERPINB5
MIRLET7A1
ACTC1
GJB3
PDGFA
STAG2
CBX2
GNAI3
DGCR11
MICALL2
ACVR2A
CKAP2L
NSMF
ABHD5
GADD45A
IL1R1
ZAP70
FIG4
HLA-C
LOXL3
IGFBP5
SF3B1
SOD2
SMARCD1
ZP3
INS-IGF2
BUD23
PROC
GNAQ
GNB3
PPP1R12C
GSTM1
PYCARD
PPP1R3A
FLVCR1
SLC47A1
CTBP1-AS
PPP5C
PPT1
OR10A4
GPR3
MIOX
ITLN1
MMP26

LEFTY2
TNNI2
STS
HBEGF
AREG
CHRNA7
HTT
TULP1
AMBP
CFL1
CYP3A4
NDUFS2
CD36
SOX5
PRF1
ZNHIT3
SH2B1
PLK1
EIF4EBP1
TUBB2B
CDX2
PTPA
RSPO1
STAT5A
CLASP1
CPT2
CLN3
CYB5A
TNFSF11
FSCN1
TNNI3
BTK
MIR183
CUL1
HTR2A
SGO1
NPY
NOS2
MAPK10
DNAH1
SERPINB2
PRSS8
ACTG2
SELP
ABCA3
ERCC6L2
KCNN4
SLC34A2
MSX2
PIP5K1C
GDF3
HSPA9
CGB3
RPE65

FOXP1
PCSK1
CAMP
CRKL
NRIP1
USP7
GHRH
PRDM16
EIF2AK2
PTK2B
TRIM21
CTCF
P4HB
PECAM1
NCOR1
OPTN
CLDN7
ITGB4
CDCA7
EPRS1
TET3
IGFBP4
RARA
TRIP11
CCND3
ANGPT2
PRPF8
PSEN2
SMARCAD1
TACR3
AQP3
SLC30A7
PSAP
MIR24-1
PCSK2
VEGFD
ANTXR1
CX3CR1
PDE8B
SLC6A2
IKZF1
GDF1
CEBPB
RMI2
HS6ST1
HCRT
CD24
APLN
ABCG5
CDC25C
BCL2L11
FOXO3
OXT
REST

PLXNC1
TF
KLK10
ID2
FGF13
PIGG
CCL5
SLC17A5
RICTOR
TPH1
SSTR3
BRD4
ONECUT1
COL17A1
RPS20
PLAG1
MIR130B
IL4R
EPHX1
MIR181A1
HADHB
TUG1
FXYD2
XRCC1
HOXB7
HTR1A
LPCAT2
SSTR2
CRB1
RPA1
FOXO1
METTL8
DNM1
ZIC3
MUC6
FAM215A
STAT2
CRX
PRKX
IL5
ABCA1
COG4
AIPL1
AGO2
PDE3A
ECHS1
CSNK2B
KRT18
EPPIN
MTR
IHH
CDK5
GAA
MIR16-1

TSG101
PGF
LCA5
CSNK1D
HSD17B10
DCN
LTA
RPS6KA1
MUC2
CYP2C9
LIPC
NR3C2
CCK
ITGA8
BCAR1
SFRP4
HTRA1
GRB2
HMX1
MIR132
BAMBI
IRF1
MKI67
GALNT17
MIR15B
FABP4
SSTR5
LDLR
SAA1
FERMT3
HMGCR
HES7
ADGRG1
CYP2D6
RDH12
ACHE
UGCG
XRCC5
PDCD1
ACP1
CANX
AKAP1
CIDEC
MOGS
FANCL
ATF2
GPR101
CTBP1
BIRC6
SAG
H2AX
TOX3
CDH11
FGF4

INHBB
GDI1
CEP83
PLK4
PRKAA2
GZMB
XDH
SLC5A5
POSTN
SLC9A3R1
TP53BP1
RCC1L
CGB7
CDK10
SRSF2
JUP
ARNTL
SEC61B
CLOCK
GLS
ACADM
ANXA2
ACVR1
ACVRL1
KRT17
NEUROD1
ZNF217
SPRY4-IT1
GALE
CCL3
KRT8
LIG1
PLXNA3
PNPT1
SPATA7
MLANA
ZP2
CD19
CRHR1
DYNC1H1
PRKAR1B
TGIF1
DHX34
CETP
SLC4A2
PHKA2
ITGAM
PRKDC
ACVR1B
SOX8
PPP3CA
PXN
LOC110408762
CNTLN

FGFR4
ODC1
LMNB1
MMP8
IGFBP6
IL15
NR5A2
MIRLET7B
IMPDH1
MCPH1
MMUT
KAT8
ADM
NHEJ1
GSC
PCM1
KIF11
CFAP300
SELL
CD63
KNG1
M6PR
PKN2
CA2
RPS27A
NONO
UNC13D
GDF15
NR1H2
NEDD4
UBA1
PDE4D
CDK5RAP2
NFATC1
GJA4
SERPINA6
AIF1
FMO3
CPT1A
AARS1
ACAN
CYP1A2
SH2B3
UBC
TNFRSF11B
MIR196A1
MIR19B1
TRH
SERPINF1
SLX4
CDK20
FZD4
ANO6
NEB

ANGPT1
MIR203A
ABCB11
TRPC1
TLR3
MDC1
HEY2
SSTR1
EMD
G6PC1
PRKACG
NCOA4
ABCB4
LPIN1
BUB3
BMPR2
NLRP5
GPR161
GRIA1
APRT
CCP110
MB
S100A9
CEP97
TMEM17
LPA
GCM2
CSH1
KRT1
CNR1
PIK3CB
PKD1P6-NPIPP1
ACE2
LAMB1
HNMT
GALNT3
CAT
ADCY5
MIR193B
EML1
PI4KB
CXCR2
DDB1
EGR1
CILK1
TBX2
AGTR2
LTBP1
PARK7
DHDDS
SYNE2
HSD17B2
CHI3L1
KCNA5

MCM2
DLL1
ABCC6
CPB2
FJX1
MEIS1
RIPPLY3
GDF2
TACSTD2
FDXR
TENM3
NEUROD2
SMARCA5
DBH
MERTK
PAX9
RAPGEF3
PTPN1
NGFR
SGK1
AXL
HAND2
IL33
IGF2BP2
POC1B
ITGA4
PPP2R5D
EPAS1
WDR1
UFSP2
ABCA7
HAAO
LOC110806262
PRKAR2B
NR2F2
FAT1
DNAH9
GAB1
MTMR6
TERF2
CUL3
PNPLA2
NEDD4L
CORIN
PLD3
PODXL
CD9
ZFPM2-AS1
BDKRB2
OTOA
MSTN
DRD3
NEK2
GUCY2D

PRKD3
OPRM1
IL11
TUBB6
ITGB6
ACKR3
ABCC3
RPS6
FAM161A
BCHE
HDAC5
PDSS1
UBB
STUB1
JAG2
CD68
ABHD11
CEBPA
CSH2
BAG2
RHEB
MCM3
CD200
ALPK1
UCP1
SPAST
CEP89
FLAD1
PGRMC1
NFE2L2
RAB3IP
ACVR2B
ATF4
MAOB
HYOU1
STAG1
GC
RAB11A
TAC3
SCTR
POC5
NINL
SLC30A8
STON1-GTF2A1L
MAPKAPK2
GPBAR1
CCDC88C
SLC30A9
ADAM12
CHRNA4
CENPC
PRKACB
YWHAZ
CCN1

SEPTIN5
DDC
LTF
POU1F1
RAPGEF4
AVPR1B
DRD5
GLMN
XPO1
LRP1B
HNRNPA2B1
TGFBR3
PRKAA1
CD163
PLXNA1
CD47
RBX1
LPP
IQGAP1
PLXNB2
PMEL
MGAM
FOXJ1
ADAM17
CES1
CYP2B6
KIF24
UBR5
CDH13
POLR1B
FHL2
IKBKB
SET
RBFOX1
CCR3
COL14A1
MIR423
COG2
CNC2
DCT
APOC1
TJP2
HEY1
ACADVL
CEACAM3
VCL
TNFSF12
EIF4E
DSPP
ATXN10
RPTOR
CYP27B1
POU3F3
SSH1

SDC1
VRK2
MAP2
SLC4A4
ALPG
CYP3A7
EME2
BDKRB1
STK36
GHSR
PHKG2
HTR2C
TACR1
UBE2I
OPRK1
NOS1
SMAD1
NCOA2
BIRC3
SCARB1
DCTN2
CXCL11
CXCR1
APPL1
SUMO1
ISL1
TNFRSF11A
AKT1S1
HIBCH
MIR502
MIR185
IL23A
IL34
PF4
GRIK2
IDO1
CROCC
CHKA
MLST8
APOA4
PLA2G4A
YWHAQ
HSP90B1
ABCB7
RIN1
SOCS1
TERF2IP
BCL7B
CAMK2G
GFRA1
LOC110006318
PPIG
DLG4
MSMO1

FBP1
HSD17B3
TNS1
TPM1
ITGAL
PC
LCAT
RAB5A
RPS7
NCR1
ADAMTS9-AS1
TUBG1
NUCB2
CDKAL1
CD14
LATS1
GAL
INPPL1
HOXD9
HEYL
TBX6
MYO1C
PDP1
MPDU1
MTMR2
HDAC3
MRPS16
TTC30B
ANXA4
MTNR1B
HABP2
CITED2
WNT10B
ARSA
PRDM2
COL4A2
CEP20
DRD1
MIR27B
WNT2B
EEF1A1
TJP1
STOX1
IL18R1
WWP2
MIR338
NPAS2
VAMP2
HSD17B12
NPC1
ROCK1
KIR2DS4
KRT6A
TOPBP1

UGT2B7
ZBTB16
KRT5
DNASE1
HDAC7
LOC109623489
ANGPTL4
UCHL1
USF1
BMP7
PRDX1
PROK1
UGGT1
YWHAB
ADCY3
KCNJ16
PPP1R3A
LBP
ANK3
NR4A1
GABRB2
DVL2
EARS2
PGAP1
MED15
SRGAP1
CX3CL1
KRT10
SFN
TUBA1B
SREBF2
SCG5
PDCD4
PTGER2
HPX
SLC18A2
PRKAR2A
ANPEP
ABHD11-AS1
MIR199A2
CLIP1
REEP1
RAD17
MIR9-3
PIFO
PKM
MAPK7
HSPA8
AKAP13
SP100
NR4A2
FZD5
STK4
CA4

EEF1B2
PDPK1
CKB
ITGA1
IL16
EXOSC9
CHRNB2
TUBB4A
LNPEP
CALB1
STIL
HTR2B
HSPE1
CMIP
RPL3
PDS5B
HSPA1A
GAS8
GATA5
SPTBN1
CEACAM7
DMBT1
SERPINF2
GHRHR
RACK1
FADS2
MAP3K14
IBSP
TMEM165
GRIA2
PTPRG
RANBP1
NCL
LOC110006317
TIMELESS
PRKAB1
COL6A3
OLR1
XPNPEP3
SLC29A2
PSMB5
MAP2K5
STK11IP
AFAP1
AKAP6
ALDH1A2
AK9
NCOA1
HTRA2
PTPRD
GJB4
GNPTG
UCP3
FUS

ARRB2
GABRA4
LGR6
SLIT2
GRM1
SCD
SLC22A4
WARS2
F11
GSTA1
ACTR3
GPC1
MIR125B2
FGF18
CXCL5
HOXD12
PRKCB
UBD
ADAMTS9
ERVW-1
MAD2L1
DUSP13
NPPC
ABCA12
MCHR1
FBXO11
MARK1
MIR330
DMRT2
CRHR2
RAB6A
F13A1
TCHP
SERPINH1
ARRB1
PTN
CHAF1A
SMURF1
PIP
EDN2
SGCB
SMN1
LMBRD1
CRAT
SLC5A3
ALDOA
GASK1B-AS1
FGF19
CYBA
DLX2
SLC8A1
ACTR2
PENK
HOXD11

ADRA2B
INPP5J
MMP19
HCST
RB1CC1
PTPRH
EMX2
LAMB3
NCF1
SULT1E1
TRPC3
ACAD9
NEDD9
GNAI1
PON2
GRB7
RAB33B
ACTN1
CPS1
ZNRF3
PSMA3
FBLN7
NCR3
DHRS11
CPOX
MUC7
FDFT1
STRA8
PRKAG3
SAR1A
SRF
USF2
EIF2S1
XPO5
SFRP5
CEP70
CLCN7
AVPR1A
SLC4A1
DNAJC6
RAD54B
RSAD2
PRKCE
AKAP8
HSD17B13
CYSLTR1
CETN2
PRKCZ
TM7SF3
IL37
EYA3
CES2
MEF2A
RRM2

MMP26
CYP2A6
ADH1B
NUPR1
TIA1
C2orf69
EIPR1
ANKAR
SCN7A
FEM1A
IL32
DYNLL1
SYNPO2
TUBB4B
HAS1
TPTE2
NR0B2
DLX1
PRIMPOL
CDK12
CKAP5
PLIN3
MNT
TBC1D21
CDC27
ABCG8
SYTL2
TFAP2A-AS1
ZNF142
FDX1
CCL20
MEIOB
SLC22A2
PDYN
FAAH
GP2
TRPV6
OTUD4
MLKL
CCDC115
GREB1
HIVEP1
PEBP1
CHRNA3
AKR1B1
CCDC120
PNPLA3
AKR1C1
UBE2D3
SACM1L
SULT1A1
CABLES1
LCT
KLF11

HOXD3
NEIL2
SLC25A11
HPGD
PRKRA
KIF19
PDLIM5
POLR3K
PSMB2
SGTA
BCL2L2
AKR1C2
HHIP
PGK1
MTMR10
RUNX1T1
MIR574
SHANK1
NKX2-2
PDXK
IL21
MAN2C1
LOC117152610
WAPL
AQP9
UGT2B4
HCP5
RPA2
STRADB
GPNMB
TRAP1
MBOAT2
PTPRN
HAS2
UNC119B
MAZ
LATS2
MARS2
BMP10
VEGFB
MUC3A
NOP2
FOXL1
FUT8
HBZ
PSMD1
MAL
FEV
RFWD3
MXD1
EIF5A
CDH24
LOC110386951
ESM1

CAPN1
SLC2A6
HTRA3
PIGR
NCK2
SKP1
INHBC
LGR4
GNA15
PROC
CBARP
YWHAH
IST1
RNF4
OPRD1
PIKFYVE
TRIM50
P2RX7
CDIPT
TLK1
ANKK1
PLS3
GTF3A
TLN1
SLC11A1
LGR5
MARK4
GGCX
HBQ1
CCL22
PSMC1
RIF1
SMAD5
MSBP1
CTNNAL1
ZFP36L2
INCENP
RBKS
TFPI
ING5
MARK3
MTNR1A
LCN1
IRS4
ANO1
NRP2
FNDC4
UGT2A3
RPUSD1
PLIN2
ENTPD1
MEPE
LMAN2L
MRPL44

LRIG1
RBM46
NFAT5
B4GALT6
CHRNA2
ENPEP
SLC12A7
EDC4
KRCC1
PRDX2
ACOT11
CD2
ESPL1
GBX2
SCAP
MIR186
BCL7C
SERPINB4
REL
AFM
CPQ
WWC1
CHRNA5
VAMP8
EPHA7
REG1A
DEPTOR
AP2B1
ZNF350
TRIP10
TRPV5
RXRA
ZFHX3
TUBA4A
VPS52
LEFTY1
ULK1
BCO1
ALPI
UNC5C
CALCRL
TNFRSF17
CSN1S1
ACVR1C
DNAJA3
HAS3
NAP1L1
CCDC170
AGPAT1
CALCR
PDE8A
FMN2
B3GALT4
GDF11

NPSR1-AS1
SMC2
FLOT1
SH3BP4
ZNF23
ANXA1
ZNF274
RNH1
DLGAP1
PAF1
LAMC2
ZNF318
UGDH
SLC22A3
CRYZL1
FA2H
ACADL
IL1R2
NME2
SERPINE2
LZTS1
MC3R
SLC5A6
GPAM
USP48
GNB4
NBEAL1
NPY2R
CLCN3
UTS2
IGFL3
PEA15
KRT9
ESYT1
IVD
RAB3IL1
ORMDL1
IGFBP7
TUBB2A
FAM124B
ROCK2
PLA2G10
TOR2A
GSTM3
CYSLTR2
SLIT3
PROKR1
CHP2
FBXO31
PTPN13
DHRS9
RASAL1
TMEM120A
TTC30A

MIR9-2
STK26
CETN1
CXXC4
ELOB
SCAMP1
RCN2
LCLAT1
TRPC4
TXNIP
BAIAP2L1
IQGAP2
RBL2
HELQ
LOC108783649
CSMD1
RCC1
ARF6
FGFR1OP2
HPR
ATAD3B
BST1
CAMKK2
COL16A1
ENHO
EPHX2
MIS18BP1
ATP1A1
PDE4A
GRK3
IGKC
TACC1
COL8A1
CLIC1
RHOB
PPP3R1
BCKDK
MIR136
L2HGDH
RAPH1
RNF212
DBI
HTR5A
SLC2A4RG
PRKG2
ANGPTL2
ODF2
LEPROTL1
TMEM258
TCL1A
PSG1
CHRNB4
ABCC12
LOC109029530

SLC9A3R2
KRT13
SFPQ
NUP35
E2F4
NAGK
ANKRD39
MAGEA11
PPA2
EPHB2
GOLGA5
ADH4
CCAR2
TNFAIP6
REV1
EBF2
SF1
CENPA
GLP1R
ATP5F1B
ADHFE1
FAP
FMO1
FOLH1
HBE1
IGFALS
IVL
CAMK2D
SNX1
LUM
ATG13
NFXL1
LY75
HDLBP
BABAM2
TRIB3
DNAJC14
MGAT1
USO1
CLSPN
KHDRBS3
FOSL2
ATP2A1
TMPRSS11A
NEK11
PARD3B
ZNF169
NNMT
ACP3
SMPD3
ETV7
RAP2A
SPNS1
PRKCI

DDIT4
BRD7
TDP2
C1QTNF12
BCAR3
DKK3
SSTR4
BMP5
PRDX4
DMWD
MT3
CHFR
TRIB2
SEC24B
CARTPT
HMOX2
CIAPIN1
IRF2
PSMA2
GDF7
SLC12A4
PIAS1
SERPINA4
FZD7
NME3
RNLS
MIR760
SLC39A10
ITFG1
TBC1D10C
CD300LD
PDIA2
BANCR
CHRNB3
RRBP1
TIMP4
MIR16-2
INSIG1
RAP1GDS1
MAP3K20
EME1
CST6
RTN4
TLR6
CDK9
KCTD13
CST9L
TSGA10
KRT78
TUBA3D
CCDC92
NFATC3
NRBP1
SLC30A6

TSN
MPG
RPL10L
GUK1
NEK4
DOT1L
MAML3
SLC25A3
SRI
INTS9
P2RY2
CCNT2
LOC110386947
KDM3A
CBR3
ZFX
NPNT
EMILIN1
CYP2R1
PSMD11
ARF3
NDRG4
LOC108863620
LAT
DDAH2
DYNC1LI1
RAB1A
NMI
DUSP2
CCT4
ACAD11
AQP8
APOF
RXFP1
CDC5L
AVEN
CXCL6
D2HGDH
SMC4
TPBG
SORBS2
NPTN-IT1
CHMP4B
BTRC
MIOX
APLNR
PSMB1
STK3
WNT8B
ADCY7
STARD7
ASIC1
UCN
SMPD4

NPRL3
CCNG2
FYCO1
NASP
APLP1
AKR1C4
CAPZB
PREB
MIR218-1
INTS6
ZC3H18
PPP1R7
RPL7A
C1QL3
BMP3
GALNT14
ZP4
TRADD
TREH
CXCL3
DPH2
ANGPTL1
TUBA1C
BCAS1
CASC22
LHX9
UBA6
DNAJC10
ACKR2
MARCHF7
KLKB1
MIR3940
TNNI3K
PDK1
RSL1D1
HAVCR1
GNRHR2
TMEM18
DNAI7
ECT2
BCCIP
TNIP2
PLPPR3
PKMYT1
PRMT5
SCD5
B3GNT5
NOP58
PDXDC1
PSMB3
C19orf33
EHBP1
SLC20A1
C2orf88

TMSB10
RBBP7
RAB36
PDGFC
MMADHC
HGFAC
CHRNA6
SEMA4F
LDHAL6A
FAM136A
BIN1
PTTG2
CNOT11
RALB
E2F6
SGK3
BCAT1
MREG
ORC2
SETD7
GPAT3
NAB1
TENT5A
SHCBP1
PIGF
RAB3C
EIF3C
PSMA7
AGA
HNRNPD
BANP
ACOXL
HES6
IL36A
BOD1L1
PLAC1
BCAR4
SPEG
GNPDA2
GPAT4
HNRNPDL
HSPA6
GCSH
NM
EIF4A1
DYNC1LI2
CHRNA9
ASTL
PLPP1
BPNT1
TP53I3
INPP4B
DOK1
CNNM3

RPL41
C2CD6
GULP1
INSRR
ADGRL3
RPLP2
RCHY1
INHBE
DUSP19
DNAJA1
TRIM2
GORASP2
PRPF4B
GPR1
TNIP3
MAP3K2
GALR2
LRPAP1
GALM
ATG9A
TMEFF2
PPIF
METRN
FKBP7
FARSB
ATP5MF
LIMS2
CSE1L
TCF7L1
USP39
LNPK
HIF1AN
NGEF
CAPG
CAP1
EPHA5
ASPHD1
BAG6
FAM98A
FLOT2
ZFP42
UBXN4
PGAM5
PRPF19
SUPT3H
SMURF2
MMRN1
PTPN18
SPC25
MAP4K4
ACTR1B
GDE1
USP10
ACTR1A

NACA
CCT8
CCT7

GEO（GSE5850,GSE98421,GSE34526）	Genes present in at least two databases
MMP9	CYP19A1
PLTP	AKR1C3
TAPBP	STAR
IL18RAP	BCL2
RP11-456P18.2	HSD3B1
FCGR1B	FSHR
KIAA0247	LMNA
FAM81A	GNAS
KCNA1	GDF9
ITGB2	BMP15
ADRB1	INS
ARAP3	LEP
TMEM176B	HSD3B2
NAPA	LIF
MYO10	LHB
SDHAF2	RBP4
TMEM130	MMP2
DR1	AZGP1
FCGR2A	MTNR1B
NAIP	SRD5A1
PCDH7	GAB1
FGF3	CGB5
UTS2B	MALAT1
KCNT2	AKT2
ZDHHC6	AKR1C1
ST6GALNAC5	CLDN4
CH25H	PNPLA2
B4GALT5	TFRC
TRHDE	BAX
SLITRK4	TMEFF2
THBD	RRM2
FABP5	AKR1C2
ASNA1	TOP2A
SPI1	SCNN1A
SLC13A4	SCT
IL6ST	DLG4
RP11-164P12.3	BCL11A
DDX52	MAD2L1
ZFHX4	BLOC1S3
CSHL1	NRG1
PBK	LPCAT2
XPC	CEP55
CDH19	TRPV6
SLC4A4	CAMK2D
RIMS4	FGF18
RAPH1	IL34
GPR25	PGR
TAGLN2	RARRES2
LOC101929118	NR3C1
FCER1G	ADRB2
VPS28	CD36
PVR	ADRB3
RAB3B	NCOR1

BHMT
LOC284379
PLA2G7
ATF7IP2
POPDC3
CLEC5A
ZNF229
SGPP1
ESR1
RNF11B
NPIPA1
C7
SOCS3
P2RX7
LOC100506082
PIP5K1B
RP11-792A8.4
PRKCI
TMEM45A
LOC389834
TRIM68
RNF213
IL1RN
C3AR1
C11orf87
RBM24
NFKBIE
MAMLD1
ZNF510
SDC3
PRR15
CD44
OCIAD2
LOC101927723
HLA-DPA1
LOC100505812
REPS2
ARL6IP4
PRR16
RELT
FAM78B
DVL3
CCDC85B
LOC541472
LOC145945
MAN1C1
LINC01111
CSRNP3
CD163
FAM26D
NPC1L1
ARNTL
ISM1-AS1
CACNG5
TH
DENND1A
INSR
SLPI
THADA
NGFR
LHCGR
ATP5F1B
PPARG
YAP1
PTEN
AOPEP
HNF1A
ATM
ERBB4
LIPE
HMGA2
VCAN
CBX2
FOS
CAV1
SETD2
PLIN1
ERBB3
SERPINE1
AGPAT2
IGF1
BAZ1B
AMH
ANTXR2
MAP3K1
DHH
IRS2
MTHFR
TBL2
CRB1
RFC2
FST
PRL
FLT1
IL6
CDK2
FBN3
RETN
CAPN10
CORIN
GTF2I
LMNB2
GCG
STON1-GTF2A1L
POR
IL1B
CAVIN1
BSCL2

LOC100130744
TTC32
PABPC1L
LOC646588
CTSA
ALOX12
COL11A1
FHOD1
CBLN4
NAP1L3
UCHL5
SH3RF1
ELOF1
WAS
RINT1
DNM1P35
PYCARD
CDO1
SPRY1
C1orf162
TREM2
LPPR4
CTSE
GATA6
DAGLA
PCDHB1
ATG16L2
LINC00238
SNRNP70
LOC100506851
NINJ1
GPR85
LOC286190
DENND4B
NRN1
TLR6
WDFY4
PPP2CB
PODN
AQP5
DOLK
UBE2M
INHBA
ZNF124
LOC100507600
ZFYVE16
LINC01267
ARHGAP33
LOC101929591
KRT2
PLCXD3
TPH2
AGMAT
TNFRSF11A
NR5A1
PON1
POMC
IL1A
SULT2A1
ELN
SOX5
TGFB2
SOX3
KIF7
FTO
TNF
CYP17A1
ESR1
CYP2B6
SLC2A4
ACE
SOX9
DMRT1
CRP
ZBTB16
SRY
WT1
TCF7L2
NR0B1
ALMS1
GTF2IRD1
CLIP2
VEGFA
LIMK1
CYP11A1
VDR
ADIPOQ
CIDEC
MMP14
IRS1
MSX1
AR
STOX1
PMEL
SHBG
ANGPTL8
DPP4
AMHR2
KISS1
IGF2
TGFB1
GLP1R
CYP21A2
HSD11B1
LEPR
GHRL
IL10
CXCL8

SCUBE3
STON1
C3
MAN1A1
LOC100996654
OXR1
NOTUM
NEUROG2
RP3-496C20.1
ANO4
TRAPPC1
BC041363
ATP13A2
ZNF292
NTN4
LILRA2
BRD7P3
WNT5A
RENBP
RP3-507I15.2
LAPTM5
LOC100506929
TNFSF14
UBE2A
FCGR1A
VWDE
GAREML
BC113958
EPHA7
LINC00842
KCNRG
CTNNA3
C1QTNF2
KRT76
DDAH1
RACGAP1
PHF21B
CTSH
MAP3K14-AS1
GPAT2
OR8D2
HBZ
PKM
TPP1
IGF2BP3
MUC19
C17orf99
ABRA
RNASE1
BCL2A1
ZNF667-AS1
GPR158
LAMP3
AHCYL1
H6PD
LDLR
FSHB
IL18
ESR2
PPARGC1A
PIK3CG
CYP1A1
ADIPOR1
PIK3CD
PIK3CB
PIK3CA
MIR93
APOA1
APOB
ICAM1
GNRHR
ADAMTS1
SLC2A1
EGF
ALB
CASP3
KLK3
IL1RN
MMP9
MC4R
MAPK3
PDCD4
FOXO1
PTH
ACTB
HSD17B6
AKT1
F5
PCOS1
CAT
KISS1R
ADIPOR2
MTOR
LPA
ENPP1
STK11
STS
GSK3B
NGF
PPARA
HSD17B1
GC
GATA6
INHA
SMAD2
HSD17B2
NUCB2
MIR27A

ETS2	MIR155
PAX3	SMAD3
PLEKHO1	LCN2
PTCH1	APOE
RORB	ENHO
CYBA	KCNJ11
TRANK1	IFNG
HSPA5	MIR223
MAGEA4	ITGAX
GRB14	IGFBP1
CPVL	STAT3
DEPTOR	MIR486-1
TSTD2	MAPK8
CXCL17	AGTR2
KIAA1549	CFTR
FCGR2C	PRKAA2
HHIP	GPT
CTTNBP2	CNR1
SERPINI1	AGT
CD14	PLA2G7
GHR	SIRT1
TTLL2	PCSK9
RP11-1L12.3	PROK1
DLGAP5	HAMP
GULP1	FN1
ELTD1	PTGS2
LOC100289495	BGLAP
GPX3	FAAH
LOC101928099	SOD2
LOC100507388	SOCS3
MIR17HG	VCAM1
IFFO2	ACACA
CASP8	BMP6
JADE1	CCL2
FLRT3	HLA-DRB1
MFHAS1	UCP2
WISP1	TP53
MRPS31	ADA
LOC100507006	FBN1
REL	HSD11B2
FOSL2	APLN
THEMIS	EGFR
MYLK4	CCND2
ZCCHC14	HOXA10
LOC101929019	HMGB1
IFITM2	MECP2
UPP1	BMPR2
SHISA2	SMAD7
GAS7	MIR200C
RP11-157E16.1	SRD5A2
DDX11-AS1	SPARC
DTD2	SPP1
RP11-1017G21.4	SREBF1
HCLS1	ZNF217

FGF7	SUOX
GC	TBC1D4
FOXO4	MIR21
PCSK6	CAPN5
EQTN	VIM
SLITRK5	MIR146A
LOC613266	MIR145
CNKSR3	MIR222
NUTM2A-AS1	TAC3
FHL1	MIR320A
1-Mar	PIK3R1
MTSS1L	MIR33B
DUSP21	WNT2B
KDM4D	TGFBR1
LINC00298	TLR4
SERPINB5	MIR141
LHFPL3	MEF2A
LOC101929224	PAEP
TM4SF1	PTPA
CXCL2	NPPC
AMOTL2	NOTCH1
AK5	FEM1A
CXCL3	PRKAA1
NR3C2	PRKAB1
CDH12	AVP
MARVELD3	CDKAL1
SOX5	PKD1
NADK2	PLAT
SNX10	PCSK1
C20orf202	PCNA
EFS	SERPINB2
CCR1	NOS3
FGF5	MIR323A
NDUFA1	SET
RP11-495P10.9	SFRP4
ZNF367	SGTA
PDE5A	KITLG
FAM65B	REN
SOHLH2	RUNX2
CAP2	ATF4
AHR	PTX3
INSC	RB1
LOC101928211	MTNR1A
SLC44A5	MUC1
CTH	TOX3
ZNF595	INHBA
	GATA4
	EDN1
	AGTR1
	COMT
	HSPA1A
	ZNRD2
	PRDX4
	ADM

GPX3
HSPB1
EPHX1
IAPP
INSL3
AGER
GH1
APRT
ACAN
AQP8
CYP1B1
IFI27
DCN
AQP7
MSTN
CGA
INHBB
CGB3
IL17A
ANGPTL2
HIF1A
HP
RAD50
AKR1B1
NR4A1
HHEX
HLA-A
MMRN1
LPL
AQP9
CCN2
HOTAIR
LOX
FGF2
FABP4
FABP1
F3
LPIN1
THBS1
TFAM
TFAP2A
TIMP1
DBH
C3
ANGPTL6
TNFAIP3
TNFRSF1B
CYP3A7
CYP11B1
TM4SF4
TIMP3
TIMP4
TLR2
CTLA4

CTNNB1
TPM1
ADRA2B
SLC30A8
FBLN1
F13A1
F2
BMP4
SLC5A3
SLC6A2
NCF1
EREG
SFRP5
SCD
FGF13
ALDH1A3
FAT1
BMPR1A
SLC18A2
SLC22A1
STC1
SULT1E1
TACR3
AFM
NQO1
MIR592
DRD3
SLC22A3
EPO
EPHB2
EPHA7
AHSG
EDN2
ABCA1
SREBF2
SRF
PEA15
ACVR1
SORBS1
CECR
CAP1
CDX2
ANGPTL1
STUB1
POSTN
IGF2BP2
TNFSF12
FGF19
TNFRSF10B
HDAC3
CCK
KHDRBS3
BANCR
ROCK2

NR1H3
INSL5
CD24
CD68
GREB1
USP34
FADS2
ACVR2A
AKAP8
CDKN1B
CDKN1A
IL32
DHRS9
ACVR2B
NAMPT
KL
FEM1B
SERPINA12
CPB2
PWRN2
DHRS11
CALCA
COPA
CPT1A
WFS1
WNT5A
WNT8B
XK
XRCC1
YWHAZ
CSF3
CPT2
TMEM18
FLAD1
CYP2R1
LEAP2
CASR
C1QTNF3
SORCS1
NR0B2
CASP9
NCOA4
PNPLA3
FGF23
NCOA3
NRIP1
IMMP2L
BCAR3
BDNF
MMP1
MUC5AC
FAS
SERPINC1
ATD

NBN
NFIC
CCN1
IGFBP6
IGFBP4
NHS
NNMT
IL1R1
IL2
MPG
IRF1
MIR151A
CXCL10
MIR335
IL11
IL7
MTR
NOS2
IGFBP2
HSD17B13
HSP90AA1
HSPB2
INSIG2
HSPA9
HSPA5
HSPA4
PDE8A
PDGFRA
IL23A
GDE1
PDK1
NOTCH3
NPPB
P2RY2
IDE
PAFAH1B1
PRDX1
APOC1
HTR2C
PAPPA
PAX6
LUM
FADS1
LGALS3
LCT
MIR23A
MIR23B
MIR25
SUMO1P1
MIR29A
MIR203A
MIR200B
MIR106B
MIR130B

ARNTL
MIR142
LRP5
LPP
MIR182
MIR186
MIR19B1
MIR30C1
MIR30C2
MCM2
MDM2
JUN
JAK2
MEP1A
ITGB3
MIF
MC3R
C1QL3
MIR99A
SMAD1
C1QTNF12
SMAD4
SMAD6
KNG1
MAGEA11
AREG
HSD17B4
SLC2A4RG
ARID1B
APPL1
GAPDH
TRIB3
RAD54B
ZP4
GGT1
PYY
ADAMTS9
GREM1
GCK
FGF21
FLNA
GAS5
ROCK1
MCF2L2
RPL3
MIR483
FOXO3
RPS6KB1
FMR1
NEIL2
RAB5B
SGK3
RASA1
LGR6

DICER1
PDPK1
BCO1
PRKAG3
ANXA5
WNT4
HAS3
HAS2
HAS1
PON2
PLAUR
SERPINF1
STK26
ADA2
PF4
HRAS
PHB
HNF4A
HMOX1
MIR30A
PIK3R2
HMGCR
PPARD
GSTT1
TOR2A
PRKAR2B
MAPK1
AGO2
MAPK7
GIP
GHR
PRLR
PROC
GNAQ
GNB3
GSTM1
PPP1R3A
CTBP1-AS
MIOX
ITLN1
MMP26
IGFBP3
MT-TL1
PBX1
FNDC5
CHIT1
LEPQTL1
MIR135A1
GPER1
MIR486-2
PEPD
IGF1R
PKHD1
MSH2

MSH6
CEP290
CC2D2A
ERCC6
FGFR2
OFD1
MKS1
BLM
KCNQ1
NPHP3
FOXL2
RPGRIP1L
BBS1
BBS10
BBS4
MKKS
ATRX
BBS2
BBS12
FGFR1
NPHP1
COL3A1
BBS5
NOTCH2
TCTN2
BBS9
BBS7
UBE3A
FRAS1
NPHP4
NRAS
ZEB2
SDCCAG8
NOBOX
UGT1A1
WDPCP
HSPG2
SOS1
PREPL
NRXN1
CASP10
COL5A2
EIF2B2
FIGLA
H19
AKT3
CASP8
CD40LG
DGCR2
DNMT3A
LRP2
AFP
CHRNA1
ANOS1

SATB2
PKD1L1
CDC73
HPRT1
SCN1A
RAB3GAP2
BCL2L1
CYLD
ESS2
DCTN1
ENG
DVL1
ZMPSTE24
MPV17
KDR
NSD2
CXCR4
MYCN
CEP104
GLI2
RAB3GAP1
C8orf37
DGUOK
DCAF17
BIRC5
EIF2B4
E2F1
PPP1CB
SUCLG1
EIF2B5
WASL
HOXD13
GPC4
CD274
CRH
IL6ST
IL4
LBR
HFE
TKT
DGCR5
OCIAD1
HLA-DQB1
CD4
TIMP2
FGF7
WWTR1
PSMB4
BMPR1B
DRD2
SLC9A1
SOD1
NBAS
HLA-G

EIF2AK3
CCNE1
SOX11
XPA
IL6R
ST3GAL5
HOXA11
XIST
MIR125B1
SRA1
HADHA
CD28
DDX59
TRPV1
GNAI3
DGCR11
CKAP2L
SF3B1
BUD23
PRF1
TNFSF11
NPY
ACTG2
EIF2AK2
PECAM1
CDCA7
TET3
PSAP
ANTXR1
HCRT
GALNT17
SAA1
FERMT3
PDCD1
GZMB
XDH
RCC1L
ODC1
MMP8
GDF15
GJA4
SH2B3
TNFRSF11B
ANGPT1
S100A9
CHI3L1
RIPPLY3
IL33
HAAO
TGFBR3
CD163
MGAM
POLR1B
MIR423

POU3F3
TNFRSF11A
APOA4
PLA2G4A
HSP90B1
SOCS1
RAB5A
MIR27B
KIR2DS4
UGT2B7
ANGPTL4
LBP
CX3CL1
MIR125B2
MMP19
STRA8
PLIN3
SLC22A2
MIR574
ESM1
INHBC
SMAD5
ZFP36L2
UTS2
TXNIP
ARF6
COL8A1
MIR136
NFXL1
ETV7
CARTPT
SERPINA4
MIR760
APLNR
PIGF
USP10
BSND
MDS2
PKP1
PRG4
USH2A
RACGAP1
PBK
CD44
FCGR1B
FCGR1A
BHMT
RENBP
BCL2A1
PYCARD
PTCH1
PAX3
THBD
MIR17HG

RINT1
SERPINB5
NR3C2
SLC4A4
CD14
PKM
CYBA
HHIP
HBZ
P2RX7
REL
DEPTOR
RAPH1
FOSL2
PRKCI
TLR6
CXCL3
GULP1
ATP13A2
COL11A1
OCIAD2
WAS
