# Supplementary material for: Identification of Bioactive Compounds and Potential Mechanisms of Kuntai Capsule in the Treatment of Polycystic Ovary Syndrome by Integrating Network Pharmacology and Bioinformatics
Source: Oxid Med Cell Longev. 2022 Apr 28;2022:3145938. doi: 10.1155/2022/3145938 (PMC9073551; doi:10.1155/2022/3145938)
Supplement: Supplementary 4 — Supplement Table 4: the intersection of KTC's therapeutic targets and PCOS-related databases. [file 3145938.f4.pdf]

PGR
NR3C2
IL6R
CD14
LBP
PTGS2
HSP90AA1
ADRB2
BCL2
BAX
CASP9
JUN
CASP3
CASP8
PON1
NOS2
AR
PPARG
DPP4
F2
SLC6A2
TOP2A
AKT1
MAPK8
MMP1
HMOX1
NPPB
CYP1A1
ICAM1
VCAM1
CYP1B1
HAS2
SLC2A4
GSTM1
AKR1C3
SLPI
ESR1
ESR2
CDK2
AKR1B1
EGFR
BCL2L1
FOS
CDKN1A
MMP2
MMP9
MAPK1
RB1
TP53
ODC1
SOD1
HIF1A
HSPA5
ACACA

CAV1
F3
IL1B
CCL2
CXCL8
BIRC5
NOS3
HSPB1
PLAT
THBD
SERPINE1
IFNG
IL1A
NCF1
NQO1
COL3A1
CLDN4
PPARA
PPARD
CXCL10
SPP1
RUNX2
E2F1
IGFBP3
IGF2
CD40LG
IRF1
ERBB3
RASA1
CYP19A1
GSK3B
KDR
FN1
FOSL2
