# Supplementary material for: Identification of Bioactive Compounds and Potential Mechanisms of Kuntai Capsule in the Treatment of Polycystic Ovary Syndrome by Integrating Network Pharmacology and Bioinformatics
Source: Oxid Med Cell Longev. 2022 Apr 28;2022:3145938. doi: 10.1155/2022/3145938 (PMC9073551; doi:10.1155/2022/3145938)
Supplement: Supplementary 5 — Supplement Table 5: analysis of PPI network, GO, and KEGG of KTC's potential therapeutic targets. [file 3145938.f5.pdf]

PPI network

node1	node2	node1_stri	node2_string_id
ADRB2	IL1B	9606.ENSF	9606.ENSP00000263341
ADRB2	EGFR	9606.ENSF	9606.ENSP00000275493
ADRB2	F2	9606.ENSF	9606.ENSP00000308541
AKR1B1	AKR1C3	9606.ENSF	9606.ENSP00000369927
AKR1C3	AR	9606.ENSF	9606.ENSP00000363822
AKR1C3	CYP1A1	9606.ENSF	9606.ENSP00000369050
AKR1C3	CYP1B1	9606.ENSF	9606.ENSP00000478561
AKR1C3	CYP19A1	9606.ENSF	9606.ENSP00000379683
AKT1	MAPK1	9606.ENSF	9606.ENSP00000215832
AKT1	HMOX1	9606.ENSF	9606.ENSP00000216117
AKT1	MMP2	9606.ENSF	9606.ENSP00000219070
AKT1	SERPINE1	9606.ENSF	9606.ENSP00000223095
AKT1	CCL2	9606.ENSF	9606.ENSP00000225831
AKT1	HSPB1	9606.ENSF	9606.ENSP00000248553
AKT1	ICAM1	9606.ENSF	9606.ENSP00000264832
AKT1	ERBB3	9606.ENSF	9606.ENSP00000267101
AKT1	RB1	9606.ENSF	9606.ENSP00000267163
AKT1	TP53	9606.ENSF	9606.ENSP00000269305
AKT1	SOD1	9606.ENSF	9606.ENSP00000270142
AKT1	NCF1	9606.ENSF	9606.ENSP00000289473
AKT1	BAX	9606.ENSF	9606.ENSP00000293288
AKT1	VCAM1	9606.ENSF	9606.ENSP00000294728
AKT1	NOS3	9606.ENSF	9606.ENSP00000297494
AKT1	BCL2L1	9606.ENSF	9606.ENSP00000302564
AKT1	FOS	9606.ENSF	9606.ENSP00000306245
AKT1	CXCL8	9606.ENSF	9606.ENSP00000306512
AKT1	CASP3	9606.ENSF	9606.ENSP00000311032
AKT1	SLC2A4	9606.ENSF	9606.ENSP00000320935
AKT1	HSPA5	9606.ENSF	9606.ENSP00000324173
AKT1	GSK3B	9606.ENSF	9606.ENSP00000324806
AKT1	NOS2	9606.ENSF	9606.ENSP00000327251
AKT1	CASP9	9606.ENSF	9606.ENSP00000330237
AKT1	HSP90AA1	9606.ENSF	9606.ENSP00000335153
AKT1	CAV1	9606.ENSF	9606.ENSP00000339191
AKT1	ESR2	9606.ENSF	9606.ENSP00000343925
AKT1	E2F1	9606.ENSF	9606.ENSP00000345571
AKT1	FN1	9606.ENSF	9606.ENSP00000346839
AKT1	CASP8	9606.ENSF	9606.ENSP00000351273
AKT1	PTGS2	9606.ENSF	9606.ENSP00000356438
AKT1	CD40LG	9606.ENSF	9606.ENSP00000359663
AKT1	JUN	9606.ENSF	9606.ENSP00000360266
AKT1	RUNX2	9606.ENSF	9606.ENSP00000360493
AKT1	MMP9	9606.ENSF	9606.ENSP00000361405
AKT1	AR	9606.ENSF	9606.ENSP00000363822
AKT1	IGFBP3	9606.ENSF	9606.ENSP00000370473
AKT1	SPP1	9606.ENSF	9606.ENSP00000378517
AKT1	BCL2	9606.ENSF	9606.ENSP00000381185
AKT1	CDKN1A	9606.ENSF	9606.ENSP00000384849
AKT1	IGF2	9606.ENSF	9606.ENSP00000391826
AKT1	ESR1	9606.ENSF	9606.ENSP00000405330
AKT1	HIF1A	9606.ENSF	9606.ENSP00000437955
AR	MAPK1	9606.ENSF	9606.ENSP00000215832
AR	ERBB3	9606.ENSF	9606.ENSP00000267101
AR	RB1	9606.ENSF	9606.ENSP00000267163
AR	TP53	9606.ENSF	9606.ENSP00000269305
AR	EGFR	9606.ENSF	9606.ENSP00000275493

AR	BCL2L1	9606.ENSI	9606.ENSP00000302564
AR	CASP3	9606.ENSI	9606.ENSP00000311032
AR	GSK3B	9606.ENSI	9606.ENSP00000324806
AR	PGR	9606.ENSI	9606.ENSP00000325120
AR	HSP90AA1	9606.ENSI	9606.ENSP00000335153
AR	CAV1	9606.ENSI	9606.ENSP00000339191
AR	NR3C2	9606.ENSI	9606.ENSP00000350815
AR	CASP8	9606.ENSI	9606.ENSP00000351273
AR	JUN	9606.ENSI	9606.ENSP00000360266
AR	RUNX2	9606.ENSI	9606.ENSP00000360493
AR	MMP9	9606.ENSI	9606.ENSP00000361405
AR	CYP19A1	9606.ENSI	9606.ENSP00000379683
AR	MAPK8	9606.ENSI	9606.ENSP00000378974
BAX	TP53	9606.ENSI	9606.ENSP00000269305
BAX	CASP8	9606.ENSI	9606.ENSP00000351273
BAX	CASP9	9606.ENSI	9606.ENSP00000330237
BAX	CASP3	9606.ENSI	9606.ENSP00000311032
BAX	MAPK8	9606.ENSI	9606.ENSP00000378974
BAX	BCL2	9606.ENSI	9606.ENSP00000381185
BAX	BCL2L1	9606.ENSI	9606.ENSP00000302564
BCL2	MAPK1	9606.ENSI	9606.ENSP00000215832
BCL2	TP53	9606.ENSI	9606.ENSP00000269305
BCL2	SOD1	9606.ENSI	9606.ENSP00000270142
BCL2	BCL2L1	9606.ENSI	9606.ENSP00000302564
BCL2	CASP3	9606.ENSI	9606.ENSP00000311032
BCL2	CASP8	9606.ENSI	9606.ENSP00000351273
BCL2	MAPK8	9606.ENSI	9606.ENSP00000378974
BCL2	ESR1	9606.ENSI	9606.ENSP00000405330
BCL2L1	MAPK1	9606.ENSI	9606.ENSP00000215832
BCL2L1	CDK2	9606.ENSI	9606.ENSP00000266970
BCL2L1	TP53	9606.ENSI	9606.ENSP00000269305
BCL2L1	CXCL8	9606.ENSI	9606.ENSP00000306512
BCL2L1	CASP3	9606.ENSI	9606.ENSP00000311032
BCL2L1	CASP9	9606.ENSI	9606.ENSP00000330237
BCL2L1	MAPK8	9606.ENSI	9606.ENSP00000378974
BCL2L1	CASP8	9606.ENSI	9606.ENSP00000351273
BIRC5	CDK2	9606.ENSI	9606.ENSP00000266970
BIRC5	TP53	9606.ENSI	9606.ENSP00000269305
BIRC5	RASA1	9606.ENSI	9606.ENSP00000274376
BIRC5	CASP3	9606.ENSI	9606.ENSP00000311032
BIRC5	CASP9	9606.ENSI	9606.ENSP00000330237
BIRC5	TOP2A	9606.ENSI	9606.ENSP00000411532
CASP3	MAPK1	9606.ENSI	9606.ENSP00000215832
CASP3	HMOX1	9606.ENSI	9606.ENSP00000216117
CASP3	HSPB1	9606.ENSI	9606.ENSP00000248553
CASP3	IL1B	9606.ENSI	9606.ENSP00000263341
CASP3	CDK2	9606.ENSI	9606.ENSP00000266970
CASP3	RB1	9606.ENSI	9606.ENSP00000267163
CASP3	TP53	9606.ENSI	9606.ENSP00000269305
CASP3	EGFR	9606.ENSI	9606.ENSP00000275493
CASP3	NOS3	9606.ENSI	9606.ENSP00000297494
CASP3	MMP9	9606.ENSI	9606.ENSP00000361405
CASP3	HSPA5	9606.ENSI	9606.ENSP00000324173
CASP3	JUN	9606.ENSI	9606.ENSP00000360266
CASP3	MAPK8	9606.ENSI	9606.ENSP00000378974
CASP3	CASP9	9606.ENSI	9606.ENSP00000330237
CASP3	CDKN1A	9606.ENSI	9606.ENSP00000384849
CASP3	CASP8	9606.ENSI	9606.ENSP00000351273

CASP8	MAPK1	9606.ENSF	9606.ENSP00000215832
CASP8	IL1B	9606.ENSF	9606.ENSP00000263341
CASP8	TP53	9606.ENSF	9606.ENSP00000269305
CASP8	EGFR	9606.ENSF	9606.ENSP00000275493
CASP8	CD14	9606.ENSF	9606.ENSP00000304236
CASP8	COL3A1	9606.ENSF	9606.ENSP00000304408
CASP8	HSPA5	9606.ENSF	9606.ENSP00000324173
CASP8	CASP9	9606.ENSF	9606.ENSP00000330237
CASP8	HSP90AA1	9606.ENSF	9606.ENSP00000335153
CASP8	MAPK8	9606.ENSF	9606.ENSP00000378974
CASP9	MAPK1	9606.ENSF	9606.ENSP00000215832
CASP9	TP53	9606.ENSF	9606.ENSP00000269305
CASP9	MAPK8	9606.ENSF	9606.ENSP00000378974
CASP9	JUN	9606.ENSF	9606.ENSP00000360266
CAV1	KDR	9606.ENSF	9606.ENSP00000263923
CAV1	TP53	9606.ENSF	9606.ENSP00000269305
CAV1	EGFR	9606.ENSF	9606.ENSP00000275493
CAV1	PPARG	9606.ENSF	9606.ENSP00000287820
CAV1	NOS3	9606.ENSF	9606.ENSP00000297494
CAV1	GSK3B	9606.ENSF	9606.ENSP00000324806
CAV1	HSP90AA1	9606.ENSF	9606.ENSP00000335153
CAV1	PTGS2	9606.ENSF	9606.ENSP00000356438
CAV1	ESR1	9606.ENSF	9606.ENSP00000405330
CCL2	MAPK1	9606.ENSF	9606.ENSP00000215832
CCL2	HMOX1	9606.ENSF	9606.ENSP00000216117
CCL2	MMP2	9606.ENSF	9606.ENSP00000219070
CCL2	SERPINE1	9606.ENSF	9606.ENSP00000223095
CCL2	IL1A	9606.ENSF	9606.ENSP00000263339
CCL2	IL6R	9606.ENSF	9606.ENSP00000357470
CCL2	TP53	9606.ENSF	9606.ENSP00000269305
CCL2	F3	9606.ENSF	9606.ENSP00000334145
CCL2	CD40LG	9606.ENSF	9606.ENSP00000359663
CCL2	IFNG	9606.ENSF	9606.ENSP00000229135
CCL2	FN1	9606.ENSF	9606.ENSP00000346839
CCL2	SPP1	9606.ENSF	9606.ENSP00000378517
CCL2	MAPK8	9606.ENSF	9606.ENSP00000378974
CCL2	MMP9	9606.ENSF	9606.ENSP00000361405
CCL2	PTGS2	9606.ENSF	9606.ENSP00000356438
CCL2	MMP1	9606.ENSF	9606.ENSP00000322788
CCL2	ICAM1	9606.ENSF	9606.ENSP00000264832
CCL2	VCAM1	9606.ENSF	9606.ENSP00000294728
CCL2	CXCL10	9606.ENSF	9606.ENSP00000305651
CCL2	IL1B	9606.ENSF	9606.ENSP00000263341
CCL2	FOS	9606.ENSF	9606.ENSP00000306245
CCL2	JUN	9606.ENSF	9606.ENSP00000360266
CCL2	CXCL8	9606.ENSF	9606.ENSP00000306512
CD14	LBP	9606.ENSF	9606.ENSP00000217407
CD40LG	IFNG	9606.ENSF	9606.ENSP00000229135
CD40LG	ICAM1	9606.ENSF	9606.ENSP00000264832
CD40LG	VCAM1	9606.ENSF	9606.ENSP00000294728
CD40LG	MAPK8	9606.ENSF	9606.ENSP00000378974
CDK2	MAPK1	9606.ENSF	9606.ENSP00000215832
CDK2	ESR1	9606.ENSF	9606.ENSP00000405330
CDK2	HSP90AA1	9606.ENSF	9606.ENSP00000335153
CDK2	E2F1	9606.ENSF	9606.ENSP00000345571
CDK2	TP53	9606.ENSF	9606.ENSP00000269305
CDK2	RB1	9606.ENSF	9606.ENSP00000267163
CDK2	CDKN1A	9606.ENSF	9606.ENSP00000384849

CDKN1A	RB1	9606.ENSI	9606.ENSP00000267163
CDKN1A	TP53	9606.ENSI	9606.ENSP00000269305
CDKN1A	HSP90AA1	9606.ENSI	9606.ENSP00000335153
CDKN1A	E2F1	9606.ENSI	9606.ENSP00000345571
CDKN1A	JUN	9606.ENSI	9606.ENSP00000360266
CDKN1A	MAPK8	9606.ENSI	9606.ENSP00000378974
CDKN1A	ESR1	9606.ENSI	9606.ENSP00000405330
CDKN1A	HIF1A	9606.ENSI	9606.ENSP00000437955
COL3A1	MMP2	9606.ENSI	9606.ENSP00000219070
COL3A1	FN1	9606.ENSI	9606.ENSP00000346839
CXCL10	IFNG	9606.ENSI	9606.ENSP00000229135
CXCL10	IRF1	9606.ENSI	9606.ENSP00000245414
CXCL10	IL1B	9606.ENSI	9606.ENSP00000263341
CXCL10	ICAM1	9606.ENSI	9606.ENSP00000264832
CXCL10	MMP9	9606.ENSI	9606.ENSP00000361405
CXCL10	CXCL8	9606.ENSI	9606.ENSP00000306512
CXCL8	MAPK1	9606.ENSI	9606.ENSP00000215832
CXCL8	HMOX1	9606.ENSI	9606.ENSP00000216117
CXCL8	MMP2	9606.ENSI	9606.ENSP00000219070
CXCL8	SERPINE1	9606.ENSI	9606.ENSP00000223095
CXCL8	IFNG	9606.ENSI	9606.ENSP00000229135
CXCL8	IL1A	9606.ENSI	9606.ENSP00000263339
CXCL8	IL1B	9606.ENSI	9606.ENSP00000263341
CXCL8	KDR	9606.ENSI	9606.ENSP00000263923
CXCL8	ICAM1	9606.ENSI	9606.ENSP00000264832
CXCL8	EGFR	9606.ENSI	9606.ENSP00000275493
CXCL8	VCAM1	9606.ENSI	9606.ENSP00000294728
CXCL8	FOS	9606.ENSI	9606.ENSP00000306245
CXCL8	MAPK8	9606.ENSI	9606.ENSP00000378974
CXCL8	MMP1	9606.ENSI	9606.ENSP00000322788
CXCL8	FN1	9606.ENSI	9606.ENSP00000346839
CXCL8	MMP9	9606.ENSI	9606.ENSP00000361405
CXCL8	PTGS2	9606.ENSI	9606.ENSP00000356438
CXCL8	JUN	9606.ENSI	9606.ENSP00000360266
CYP19A1	PGR	9606.ENSI	9606.ENSP00000325120
CYP19A1	ESR2	9606.ENSI	9606.ENSP00000343925
CYP19A1	CYP1A1	9606.ENSI	9606.ENSP00000369050
CYP19A1	ESR1	9606.ENSI	9606.ENSP00000405330
CYP1A1	TP53	9606.ENSI	9606.ENSP00000269305
CYP1A1	GSTM1	9606.ENSI	9606.ENSP00000311469
CYP1A1	NQO1	9606.ENSI	9606.ENSP00000319788
CYP1A1	CYP1B1	9606.ENSI	9606.ENSP00000478561
CYP1A1	PPARA	9606.ENSI	9606.ENSP00000385523
CYP1B1	GSTM1	9606.ENSI	9606.ENSP00000311469
CYP1B1	NQO1	9606.ENSI	9606.ENSP00000319788
CYP1B1	ESR2	9606.ENSI	9606.ENSP00000343925
DPP4	FN1	9606.ENSI	9606.ENSP00000346839
E2F1	RB1	9606.ENSI	9606.ENSP00000267163
E2F1	TP53	9606.ENSI	9606.ENSP00000269305
E2F1	PPARG	9606.ENSI	9606.ENSP00000287820
EGFR	MAPK1	9606.ENSI	9606.ENSP00000215832
EGFR	SERPINE1	9606.ENSI	9606.ENSP00000223095
EGFR	IFNG	9606.ENSI	9606.ENSP00000229135
EGFR	HSPB1	9606.ENSI	9606.ENSP00000248553
EGFR	ERBB3	9606.ENSI	9606.ENSP00000267101
EGFR	TP53	9606.ENSI	9606.ENSP00000269305
EGFR	RASA1	9606.ENSI	9606.ENSP00000274376
EGFR	ESR2	9606.ENSI	9606.ENSP00000343925

EGFR	PPARG	9606.ENSI	9606.ENSP00000287820
EGFR	PGR	9606.ENSI	9606.ENSP00000325120
EGFR	MMP9	9606.ENSI	9606.ENSP00000361405
EGFR	IGF2	9606.ENSI	9606.ENSP00000391826
EGFR	IGFBP3	9606.ENSI	9606.ENSP00000370473
EGFR	PTGS2	9606.ENSI	9606.ENSP00000356438
EGFR	JUN	9606.ENSI	9606.ENSP00000360266
EGFR	FOS	9606.ENSI	9606.ENSP00000306245
EGFR	ESR1	9606.ENSI	9606.ENSP00000405330
EGFR	FN1	9606.ENSI	9606.ENSP00000346839
EGFR	HIF1A	9606.ENSI	9606.ENSP00000437955
EGFR	HSP90AA:	9606.ENSI	9606.ENSP00000335153
ERBB3	IGF2	9606.ENSI	9606.ENSP00000391826
ERBB3	MAPK8	9606.ENSI	9606.ENSP00000378974
ERBB3	HSP90AA:	9606.ENSI	9606.ENSP00000335153
ESR1	MAPK1	9606.ENSI	9606.ENSP00000215832
ESR1	HSPB1	9606.ENSI	9606.ENSP00000248553
ESR1	RB1	9606.ENSI	9606.ENSP00000267163
ESR1	TP53	9606.ENSI	9606.ENSP00000269305
ESR1	NOS3	9606.ENSI	9606.ENSP00000297494
ESR1	FOS	9606.ENSI	9606.ENSP00000306245
ESR1	PGR	9606.ENSI	9606.ENSP00000325120
ESR1	HSP90AA:	9606.ENSI	9606.ENSP00000335153
ESR1	ESR2	9606.ENSI	9606.ENSP00000343925
ESR1	FN1	9606.ENSI	9606.ENSP00000346839
ESR1	PTGS2	9606.ENSI	9606.ENSP00000356438
ESR1	JUN	9606.ENSI	9606.ENSP00000360266
ESR1	RUNX2	9606.ENSI	9606.ENSP00000360493
ESR1	MAPK8	9606.ENSI	9606.ENSP00000378974
ESR1	HIF1A	9606.ENSI	9606.ENSP00000437955
ESR2	MAPK1	9606.ENSI	9606.ENSP00000215832
ESR2	NOS3	9606.ENSI	9606.ENSP00000297494
ESR2	FOS	9606.ENSI	9606.ENSP00000306245
ESR2	HSP90AA:	9606.ENSI	9606.ENSP00000335153
ESR2	MAPK8	9606.ENSI	9606.ENSP00000378974
ESR2	JUN	9606.ENSI	9606.ENSP00000360266
F2	MAPK1	9606.ENSI	9606.ENSP00000215832
F2	PLAT	9606.ENSI	9606.ENSP00000220809
F2	SERPINE1	9606.ENSI	9606.ENSP00000223095
F2	FN1	9606.ENSI	9606.ENSP00000346839
F2	SPP1	9606.ENSI	9606.ENSP00000378517
F2	PPARA	9606.ENSI	9606.ENSP00000385523
F2	IGF2	9606.ENSI	9606.ENSP00000391826
F2	IGFBP3	9606.ENSI	9606.ENSP00000370473
F2	F3	9606.ENSI	9606.ENSP00000334145
F2	THBD	9606.ENSI	9606.ENSP00000366307
F3	PLAT	9606.ENSI	9606.ENSP00000220809
F3	SERPINE1	9606.ENSI	9606.ENSP00000223095
F3	THBD	9606.ENSI	9606.ENSP00000366307
FN1	MAPK1	9606.ENSI	9606.ENSP00000215832
FN1	PLAT	9606.ENSI	9606.ENSP00000220809
FN1	SERPINE1	9606.ENSI	9606.ENSP00000223095
FN1	IL1B	9606.ENSI	9606.ENSP00000263341
FN1	KDR	9606.ENSI	9606.ENSP00000263923
FN1	ICAM1	9606.ENSI	9606.ENSP00000264832
FN1	VCAM1	9606.ENSI	9606.ENSP00000294728
FN1	NOS3	9606.ENSI	9606.ENSP00000297494
FN1	MMP1	9606.ENSI	9606.ENSP00000322788

FN1	PTGS2	9606.ENSI	9606.ENSP00000356438
FN1	JUN	9606.ENSI	9606.ENSP00000360266
FN1	IGF2	9606.ENSI	9606.ENSP00000391826
FN1	SPP1	9606.ENSI	9606.ENSP00000378517
FN1	IGFBP3	9606.ENSI	9606.ENSP00000370473
FOS	MAPK1	9606.ENSI	9606.ENSP00000215832
FOS	HMOX1	9606.ENSI	9606.ENSP00000216117
FOS	IL1A	9606.ENSI	9606.ENSP00000263339
FOS	IL1B	9606.ENSI	9606.ENSP00000263341
FOS	FOSL2	9606.ENSI	9606.ENSP00000264716
FOS	TP53	9606.ENSI	9606.ENSP00000269305
FOS	PPARG	9606.ENSI	9606.ENSP00000287820
FOS	PTGS2	9606.ENSI	9606.ENSP00000356438
FOS	MMP1	9606.ENSI	9606.ENSP00000322788
FOS	MAPK8	9606.ENSI	9606.ENSP00000378974
FOS	JUN	9606.ENSI	9606.ENSP00000360266
FOSL2	MAPK1	9606.ENSI	9606.ENSP00000215832
FOSL2	MAPK8	9606.ENSI	9606.ENSP00000378974
FOSL2	JUN	9606.ENSI	9606.ENSP00000360266
GSK3B	TP53	9606.ENSI	9606.ENSP00000269305
GSK3B	JUN	9606.ENSI	9606.ENSP00000360266
GSTM1	NQO1	9606.ENSI	9606.ENSP00000319788
HIF1A	MAPK1	9606.ENSI	9606.ENSP00000215832
HIF1A	HMOX1	9606.ENSI	9606.ENSP00000216117
HIF1A	MMP2	9606.ENSI	9606.ENSP00000219070
HIF1A	TP53	9606.ENSI	9606.ENSP00000269305
HIF1A	NOS3	9606.ENSI	9606.ENSP00000297494
HIF1A	HSP90AA1	9606.ENSI	9606.ENSP00000335153
HIF1A	JUN	9606.ENSI	9606.ENSP00000360266
HMOX1	MAPK1	9606.ENSI	9606.ENSP00000215832
HMOX1	SOD1	9606.ENSI	9606.ENSP00000270142
HMOX1	PPARG	9606.ENSI	9606.ENSP00000287820
HMOX1	MMP9	9606.ENSI	9606.ENSP00000361405
HMOX1	TP53	9606.ENSI	9606.ENSP00000269305
HMOX1	MAPK8	9606.ENSI	9606.ENSP00000378974
HMOX1	IL1B	9606.ENSI	9606.ENSP00000263341
HMOX1	NOS2	9606.ENSI	9606.ENSP00000327251
HMOX1	PTGS2	9606.ENSI	9606.ENSP00000356438
HMOX1	NOS3	9606.ENSI	9606.ENSP00000297494
HMOX1	NQO1	9606.ENSI	9606.ENSP00000319788
HMOX1	JUN	9606.ENSI	9606.ENSP00000360266
HSP90AA1	MAPK1	9606.ENSI	9606.ENSP00000215832
HSP90AA1	MMP2	9606.ENSI	9606.ENSP00000219070
HSP90AA1	IFNG	9606.ENSI	9606.ENSP00000229135
HSP90AA1	IRF1	9606.ENSI	9606.ENSP00000245414
HSP90AA1	HSPB1	9606.ENSI	9606.ENSP00000248553
HSP90AA1	KDR	9606.ENSI	9606.ENSP00000263923
HSP90AA1	RB1	9606.ENSI	9606.ENSP00000267163
HSP90AA1	TP53	9606.ENSI	9606.ENSP00000269305
HSP90AA1	SOD1	9606.ENSI	9606.ENSP00000270142
HSP90AA1	NOS3	9606.ENSI	9606.ENSP00000297494
HSP90AA1	HSPA5	9606.ENSI	9606.ENSP00000324173
HSP90AA1	PGR	9606.ENSI	9606.ENSP00000325120
HSP90AA1	NOS2	9606.ENSI	9606.ENSP00000327251
HSP90AA1	MAPK8	9606.ENSI	9606.ENSP00000378974
HSP90AA1	PPARA	9606.ENSI	9606.ENSP00000385523
HSP90AA1	NR3C2	9606.ENSI	9606.ENSP00000350815
HSPA5	HSPB1	9606.ENSI	9606.ENSP00000248553

HSPA5	MAPK8	9606.ENSF	9606.ENSP00000378974
HSPB1	MAPK1	9606.ENSF	9606.ENSP00000215832
HSPB1	TP53	9606.ENSF	9606.ENSP00000269305
ICAM1	MAPK1	9606.ENSF	9606.ENSP00000215832
ICAM1	MMP2	9606.ENSF	9606.ENSP00000219070
ICAM1	IFNG	9606.ENSF	9606.ENSP00000229135
ICAM1	IRF1	9606.ENSF	9606.ENSP00000245414
ICAM1	IL1A	9606.ENSF	9606.ENSP00000263339
ICAM1	IL1B	9606.ENSF	9606.ENSP00000263341
ICAM1	NOS3	9606.ENSF	9606.ENSP00000297494
ICAM1	PTGS2	9606.ENSF	9606.ENSP00000356438
ICAM1	PPARG	9606.ENSF	9606.ENSP00000287820
ICAM1	SPP1	9606.ENSF	9606.ENSP00000378517
ICAM1	TP53	9606.ENSF	9606.ENSP00000269305
ICAM1	MMP9	9606.ENSF	9606.ENSP00000361405
ICAM1	JUN	9606.ENSF	9606.ENSP00000360266
ICAM1	MAPK8	9606.ENSF	9606.ENSP00000378974
ICAM1	VCAM1	9606.ENSF	9606.ENSP00000294728
IFNG	IL1A	9606.ENSF	9606.ENSP00000263339
IFNG	MMP9	9606.ENSF	9606.ENSP00000361405
IFNG	IL6R	9606.ENSF	9606.ENSP00000357470
IFNG	NOS2	9606.ENSF	9606.ENSP00000327251
IFNG	IL1B	9606.ENSF	9606.ENSP00000263341
IFNG	JUN	9606.ENSF	9606.ENSP00000360266
IFNG	IRF1	9606.ENSF	9606.ENSP00000245414
IGF2	MMP2	9606.ENSF	9606.ENSP00000219070
IGF2	SERPINE1	9606.ENSF	9606.ENSP00000223095
IGF2	KDR	9606.ENSF	9606.ENSP00000263923
IGF2	TP53	9606.ENSF	9606.ENSP00000269305
IGF2	MMP1	9606.ENSF	9606.ENSP00000322788
IGF2	IGFBP3	9606.ENSF	9606.ENSP00000370473
IGFBP3	MMP2	9606.ENSF	9606.ENSP00000219070
IGFBP3	TP53	9606.ENSF	9606.ENSP00000269305
IGFBP3	PPARG	9606.ENSF	9606.ENSP00000287820
IGFBP3	MMP1	9606.ENSF	9606.ENSP00000322788
IGFBP3	MAPK8	9606.ENSF	9606.ENSP00000378974
IGFBP3	SPP1	9606.ENSF	9606.ENSP00000378517
IL1A	VCAM1	9606.ENSF	9606.ENSP00000294728
IL1A	MMP1	9606.ENSF	9606.ENSP00000322788
IL1A	PTGS2	9606.ENSF	9606.ENSP00000356438
IL1A	JUN	9606.ENSF	9606.ENSP00000360266
IL1A	IL1B	9606.ENSF	9606.ENSP00000263341
IL1B	MAPK1	9606.ENSF	9606.ENSP00000215832
IL1B	MMP2	9606.ENSF	9606.ENSP00000219070
IL1B	IRF1	9606.ENSF	9606.ENSP00000245414
IL1B	SPP1	9606.ENSF	9606.ENSP00000378517
IL1B	NOS3	9606.ENSF	9606.ENSP00000297494
IL1B	MAPK8	9606.ENSF	9606.ENSP00000378974
IL1B	PPARG	9606.ENSF	9606.ENSP00000287820
IL1B	NOS2	9606.ENSF	9606.ENSP00000327251
IL1B	MMP1	9606.ENSF	9606.ENSP00000322788
IL1B	VCAM1	9606.ENSF	9606.ENSP00000294728
IL1B	MMP9	9606.ENSF	9606.ENSP00000361405
IL1B	JUN	9606.ENSF	9606.ENSP00000360266
IL1B	PTGS2	9606.ENSF	9606.ENSP00000356438
IL6R	MAPK1	9606.ENSF	9606.ENSP00000215832
IRF1	NOS2	9606.ENSF	9606.ENSP00000327251
IRF1	TP53	9606.ENSF	9606.ENSP00000269305

IRF1	JUN	9606.ENSF	9606.ENSP00000360266
IRF1	VCAM1	9606.ENSF	9606.ENSP00000294728
JUN	MAPK1	9606.ENSF	9606.ENSP00000215832
JUN	RB1	9606.ENSF	9606.ENSP00000267163
JUN	TP53	9606.ENSF	9606.ENSP00000269305
JUN	PPARG	9606.ENSF	9606.ENSP00000287820
JUN	NOS3	9606.ENSF	9606.ENSP00000297494
JUN	MMP1	9606.ENSF	9606.ENSP00000322788
JUN	NOS2	9606.ENSF	9606.ENSP00000327251
JUN	PTGS2	9606.ENSF	9606.ENSP00000356438
JUN	MMP9	9606.ENSF	9606.ENSP00000361405
JUN	PPARA	9606.ENSF	9606.ENSP00000385523
JUN	MAPK8	9606.ENSF	9606.ENSP00000378974
KDR	TP53	9606.ENSF	9606.ENSP00000269305
KDR	NOS3	9606.ENSF	9606.ENSP00000297494
MAPK1	NCF1	9606.ENSF	9606.ENSP00000289473
MAPK1	MMP1	9606.ENSF	9606.ENSP00000322788
MAPK1	NOS3	9606.ENSF	9606.ENSP00000297494
MAPK1	MMP2	9606.ENSF	9606.ENSP00000219070
MAPK1	PTGS2	9606.ENSF	9606.ENSP00000356438
MAPK1	MMP9	9606.ENSF	9606.ENSP00000361405
MAPK1	RASA1	9606.ENSF	9606.ENSP00000274376
MAPK1	MAPK8	9606.ENSF	9606.ENSP00000378974
MAPK1	PPARA	9606.ENSF	9606.ENSP00000385523
MAPK1	RB1	9606.ENSF	9606.ENSP00000267163
MAPK1	PGR	9606.ENSF	9606.ENSP00000325120
MAPK1	RUNX2	9606.ENSF	9606.ENSP00000360493
MAPK1	TP53	9606.ENSF	9606.ENSP00000269305
MAPK8	MMP2	9606.ENSF	9606.ENSP00000219070
MAPK8	SERPINE1	9606.ENSF	9606.ENSP00000223095
MAPK8	TP53	9606.ENSF	9606.ENSP00000269305
MAPK8	SOD1	9606.ENSF	9606.ENSP00000270142
MAPK8	RASA1	9606.ENSF	9606.ENSP00000274376
MAPK8	PPARG	9606.ENSF	9606.ENSP00000287820
MAPK8	VCAM1	9606.ENSF	9606.ENSP00000294728
MAPK8	SLC2A4	9606.ENSF	9606.ENSP00000320935
MAPK8	MMP1	9606.ENSF	9606.ENSP00000322788
MAPK8	PGR	9606.ENSF	9606.ENSP00000325120
MAPK8	PTGS2	9606.ENSF	9606.ENSP00000356438
MAPK8	RUNX2	9606.ENSF	9606.ENSP00000360493
MAPK8	MMP9	9606.ENSF	9606.ENSP00000361405
MMP1	MMP2	9606.ENSF	9606.ENSP00000219070
MMP1	TP53	9606.ENSF	9606.ENSP00000269305
MMP1	MMP9	9606.ENSF	9606.ENSP00000361405
MMP2	PTGS2	9606.ENSF	9606.ENSP00000356438
MMP2	SERPINE1	9606.ENSF	9606.ENSP00000223095
MMP2	SPP1	9606.ENSF	9606.ENSP00000378517
MMP2	TP53	9606.ENSF	9606.ENSP00000269305
MMP2	NCF1	9606.ENSF	9606.ENSP00000289473
MMP2	MMP9	9606.ENSF	9606.ENSP00000361405
MMP9	PLAT	9606.ENSF	9606.ENSP00000220809
MMP9	SERPINE1	9606.ENSF	9606.ENSP00000223095
MMP9	TP53	9606.ENSF	9606.ENSP00000269305
MMP9	PPARG	9606.ENSF	9606.ENSP00000287820
MMP9	NCF1	9606.ENSF	9606.ENSP00000289473
MMP9	NOS3	9606.ENSF	9606.ENSP00000297494
MMP9	PTGS2	9606.ENSF	9606.ENSP00000356438
MMP9	RUNX2	9606.ENSF	9606.ENSP00000360493

MMP9	SPP1	9606.ENSI	9606.ENSP00000378517
NCF1	VCAM1	9606.ENSI	9606.ENSP00000294728
NOS2	NOS3	9606.ENSI	9606.ENSP00000297494
NOS2	PTGS2	9606.ENSI	9606.ENSP00000356438
NOS2	PPARA	9606.ENSI	9606.ENSP00000385523
NOS3	TP53	9606.ENSI	9606.ENSP00000269305
NOS3	PPARG	9606.ENSI	9606.ENSP00000287820
NOS3	VCAM1	9606.ENSI	9606.ENSP00000294728
NOS3	PTGS2	9606.ENSI	9606.ENSP00000356438
NQO1	ODC1	9606.ENSI	9606.ENSP00000234111
NQO1	TP53	9606.ENSI	9606.ENSP00000269305
NR3C2	PGR	9606.ENSI	9606.ENSP00000325120
PGR	RB1	9606.ENSI	9606.ENSP00000267163
PGR	TP53	9606.ENSI	9606.ENSP00000269305
PLAT	THBD	9606.ENSI	9606.ENSP00000366307
PLAT	SERPINE1	9606.ENSI	9606.ENSP00000223095
PPARA	RB1	9606.ENSI	9606.ENSP00000267163
PPARG	SERPINE1	9606.ENSI	9606.ENSP00000223095
PPARG	RB1	9606.ENSI	9606.ENSP00000267163
PPARG	TP53	9606.ENSI	9606.ENSP00000269305
PPARG	RUNX2	9606.ENSI	9606.ENSP00000360493
PPARG	PTGS2	9606.ENSI	9606.ENSP00000356438
PPARG	SLC2A4	9606.ENSI	9606.ENSP00000320935
PTGS2	TP53	9606.ENSI	9606.ENSP00000269305
PTGS2	VCAM1	9606.ENSI	9606.ENSP00000294728
RB1	RUNX2	9606.ENSI	9606.ENSP00000360493
RB1	TP53	9606.ENSI	9606.ENSP00000269305
RUNX2	TP53	9606.ENSI	9606.ENSP00000269305
RUNX2	SPP1	9606.ENSI	9606.ENSP00000378517
SERPINE1	THBD	9606.ENSI	9606.ENSP00000366307
SERPINE1	TP53	9606.ENSI	9606.ENSP00000269305
SPP1	TP53	9606.ENSI	9606.ENSP00000269305
TOP2A	TP53	9606.ENSI	9606.ENSP00000269305

GO analysis

ONTOLOC	ID	GeneRatic	BgRatio	pvalue	p.adjust	qvalue	Count
BP	GO:00100	25/88	364/1867(	1.54E-22	5.39E-19	2.57E-19	25
BP	GO:00324	24/88	330/1867(	3.17E-22	5.56E-19	2.65E-19	24
BP	GO:00022	24/88	343/1867(	7.94E-22	9.27E-19	4.42E-19	24
BP	GO:00725	21/88	284/1867(	1.27E-19	1.11E-16	5.32E-17	21
BP	GO:00316	25/88	499/1867(	3.30E-19	2.31E-16	1.10E-16	25
BP	GO:00709	22/88	348/1867(	4.56E-19	2.66E-16	1.27E-16	22
BP	GO:00971	19/88	224/1867(	6.32E-19	3.16E-16	1.51E-16	19
BP	GO:20012	23/88	406/1867(	7.38E-19	3.23E-16	1.54E-16	23
BP	GO:00486	23/88	431/1867(	2.78E-18	1.08E-15	5.16E-16	23
BP	GO:00614	23/88	434/1867(	3.24E-18	1.14E-15	5.41E-16	23
BP	GO:00345	20/88	302/1867(	9.15E-18	2.91E-15	1.39E-15	20
BP	GO:00712	11/88	37/18670	1.03E-17	3.02E-15	1.44E-15	11
BP	GO:00447	18/88	222/1867(	1.31E-17	3.52E-15	1.68E-15	18
BP	GO:19012	20/88	313/1867(	1.84E-17	4.61E-15	2.20E-15	20
BP	GO:00075	17/88	192/1867(	2.55E-17	5.95E-15	2.84E-15	17
BP	GO:00003	18/88	232/1867(	2.88E-17	6.30E-15	3.00E-15	18
BP	GO:00485	21/88	385/1867(	6.53E-17	1.35E-14	6.42E-15	21
BP	GO:00714	20/88	339/1867(	8.71E-17	1.69E-14	8.08E-15	20
BP	GO:00704	21/88	394/1867(	1.04E-16	1.92E-14	9.16E-15	21
BP	GO:00069	22/88	451/1867(	1.12E-16	1.96E-14	9.37E-15	22
BP	GO:20012	17/88	230/1867(	5.41E-16	9.02E-14	4.30E-14	17
BP	GO:20003	16/88	195/1867(	8.08E-16	1.27E-13	6.05E-14	16

BP	GO:00712	17/88	236/1867(	8.33E-16	1.27E-13	6.05E-14	17
BP	GO:00092	17/88	243/1867(	1.36E-15	1.98E-13	9.45E-14	17
BP	GO:00346	15/88	168/1867(	1.96E-15	2.74E-13	1.31E-13	15
BP	GO:00016	19/88	359/1867(	4.12E-15	5.55E-13	2.65E-13	19
BP	GO:00712	16/88	217/1867(	4.40E-15	5.71E-13	2.72E-13	16
BP	GO:00466	11/88	63/18670	6.74E-15	8.44E-13	4.02E-13	11
BP	GO:00362	19/88	370/1867(	7.13E-15	8.61E-13	4.11E-13	19
BP	GO:00466	18/88	327/1867(	1.20E-14	1.40E-12	6.65E-13	18
BP	GO:00712	15/88	190/1867(	1.23E-14	1.40E-12	6.65E-13	15
BP	GO:19016	15/88	193/1867(	1.56E-14	1.71E-12	8.14E-13	15
BP	GO:19034	13/88	122/1867(	1.64E-14	1.74E-12	8.29E-13	13
BP	GO:00093	20/88	448/1867(	1.80E-14	1.85E-12	8.85E-13	20
BP	GO:00514	16/88	239/1867(	2.01E-14	2.01E-12	9.58E-13	16
BP	GO:00971	17/88	289/1867(	2.40E-14	2.34E-12	1.11E-12	17
BP	GO:00620	20/88	459/1867(	2.84E-14	2.69E-12	1.28E-12	20
BP	GO:00485	17/88	295/1867(	3.37E-14	3.10E-12	1.48E-12	17
BP	GO:00486	14/88	169/1867(	5.18E-14	4.64E-12	2.21E-12	14
BP	GO:20003	12/88	102/1867(	5.30E-14	4.64E-12	2.21E-12	12
BP	GO:00486	14/88	171/1867(	6.10E-14	5.21E-12	2.49E-12	14
BP	GO:00068	11/88	77/18670	6.94E-14	5.79E-12	2.76E-12	11
BP	GO:00510	18/88	373/1867(	1.16E-13	9.42E-12	4.49E-12	18
BP	GO:00506	19/88	434/1867(	1.26E-13	1.00E-11	4.79E-12	19
BP	GO:00462	11/88	82/18670	1.43E-13	1.11E-11	5.30E-12	11
BP	GO:20010	11/88	85/18670	2.15E-13	1.64E-11	7.80E-12	11
BP	GO:00330	15/88	239/1867(	3.62E-13	2.70E-11	1.29E-11	15
BP	GO:00421	19/88	464/1867(	4.14E-13	3.02E-11	1.44E-11	19
BP	GO:00712	14/88	205/1867(	7.47E-13	5.34E-11	2.55E-11	14
BP	GO:00085	11/88	96/18670	8.52E-13	5.97E-11	2.85E-11	11
BP	GO:00435	14/88	210/1867(	1.04E-12	7.13E-11	3.40E-11	14
BP	GO:00712	14/88	212/1867(	1.18E-12	7.96E-11	3.80E-11	14
BP	GO:00380	10/88	72/18670	1.35E-12	8.76E-11	4.18E-11	10
BP	GO:00971	10/88	72/18670	1.35E-12	8.76E-11	4.18E-11	10
BP	GO:00465	11/88	101/1867(	1.51E-12	9.60E-11	4.58E-11	11
BP	GO:00316	15/88	268/1867(	1.90E-12	1.19E-10	5.65E-11	15
BP	GO:00094	13/88	176/1867(	1.93E-12	1.19E-10	5.65E-11	13
BP	GO:20012	11/88	104/1867(	2.09E-12	1.26E-10	6.02E-11	11
BP	GO:00421	11/88	106/1867(	2.59E-12	1.54E-10	7.32E-11	11
BP	GO:00712	16/88	331/1867(	3.03E-12	1.74E-10	8.30E-11	16
BP	GO:01040	16/88	331/1867(	3.03E-12	1.74E-10	8.30E-11	16
BP	GO:00973	14/88	233/1867(	4.27E-12	2.41E-10	1.15E-10	14
BP	GO:00011	16/88	343/1867(	5.20E-12	2.89E-10	1.38E-10	16
BP	GO:00481	10/88	83/18670	5.88E-12	3.22E-10	1.53E-10	10
BP	GO:00466	11/88	115/1867(	6.40E-12	3.45E-10	1.64E-10	11
BP	GO:00481	10/88	84/18670	6.65E-12	3.53E-10	1.68E-10	10
BP	GO:20012	12/88	155/1867(	8.32E-12	4.35E-10	2.08E-10	12
BP	GO:19030	15/88	304/1867(	1.16E-11	5.97E-10	2.85E-10	15
BP	GO:00086	11/88	124/1867(	1.47E-11	7.46E-10	3.56E-10	11
BP	GO:19012	13/88	208/1867(	1.61E-11	8.07E-10	3.85E-10	13
BP	GO:00601	9/88	64/18670	1.68E-11	8.30E-10	3.96E-10	9
BP	GO:19016	10/88	93/18670	1.88E-11	9.14E-10	4.36E-10	10
BP	GO:19012	10/88	94/18670	2.09E-11	1.01E-09	4.79E-10	10
BP	GO:00506	16/88	378/1867(	2.23E-11	1.06E-09	5.04E-10	16
BP	GO:00511	12/88	169/1867(	2.31E-11	1.08E-09	5.15E-10	12
BP	GO:00075	15/88	321/1867(	2.51E-11	1.16E-09	5.51E-10	15
BP	GO:00084	13/88	217/1867(	2.75E-11	1.25E-09	5.96E-10	13
BP	GO:00075	13/88	219/1867(	3.08E-11	1.38E-09	6.60E-10	13
BP	GO:00451	13/88	223/1867(	3.87E-11	1.71E-09	8.18E-10	13
BP	GO:00071	15/88	337/1867(	4.98E-11	2.18E-09	1.04E-09	15

BP	GO:00108	12/88	182/1867(	5.51E-11	2.39E-09	1.14E-09	12
BP	GO:19040	13/88	230/1867(	5.69E-11	2.43E-09	1.16E-09	13
BP	GO:00352	11/88	143/1867(	6.98E-11	2.88E-09	1.37E-09	11
BP	GO:00508	11/88	143/1867(	6.98E-11	2.88E-09	1.37E-09	11
BP	GO:00977	11/88	143/1867(	6.98E-11	2.88E-09	1.37E-09	11
BP	GO:00351	11/88	144/1867(	7.53E-11	3.07E-09	1.46E-09	11
BP	GO:00063	12/88	188/1867(	8.05E-11	3.24E-09	1.55E-09	12
BP	GO:00316	13/88	237/1867(	8.27E-11	3.29E-09	1.57E-09	13
BP	GO:00106	15/88	351/1867(	8.81E-11	3.47E-09	1.65E-09	15
BP	GO:00160	17/88	484/1867(	9.19E-11	3.57E-09	1.70E-09	17
BP	GO:00015	16/88	416/1867(	9.27E-11	3.57E-09	1.70E-09	16
BP	GO:00102	11/88	147/1867(	9.41E-11	3.57E-09	1.70E-09	11
BP	GO:00488	17/88	485/1867(	9.48E-11	3.57E-09	1.70E-09	17
BP	GO:00901	15/88	354/1867(	9.93E-11	3.70E-09	1.76E-09	15
BP	GO:19040	10/88	111/1867(	1.12E-10	4.13E-09	1.97E-09	10
BP	GO:19013	16/88	422/1867(	1.14E-10	4.18E-09	1.99E-09	16
BP	GO:00901	15/88	360/1867(	1.26E-10	4.53E-09	2.16E-09	15
BP	GO:00018	11/88	152/1867(	1.35E-10	4.83E-09	2.30E-09	11
BP	GO:00421	11/88	153/1867(	1.45E-10	5.08E-09	2.42E-09	11
BP	GO:00620	11/88	153/1867(	1.45E-10	5.08E-09	2.42E-09	11
BP	GO:00509	17/88	499/1867(	1.47E-10	5.11E-09	2.44E-09	17
BP	GO:00063	13/88	249/1867(	1.53E-10	5.24E-09	2.50E-09	13
BP	GO:00713	13/88	250/1867(	1.60E-10	5.44E-09	2.59E-09	13
BP	GO:00510	16/88	432/1867(	1.62E-10	5.44E-09	2.59E-09	16
BP	GO:00487	16/88	434/1867(	1.73E-10	5.77E-09	2.75E-09	16
BP	GO:00356	15/88	369/1867(	1.77E-10	5.85E-09	2.79E-09	15
BP	GO:00108	10/88	117/1867(	1.89E-10	6.20E-09	2.96E-09	10
BP	GO:00457	12/88	204/1867(	2.08E-10	6.74E-09	3.22E-09	12
BP	GO:19010	7/88	35/18670	2.45E-10	7.87E-09	3.75E-09	7
BP	GO:00096	12/88	210/1867(	2.90E-10	9.25E-09	4.41E-09	12
BP	GO:00457	15/88	383/1867(	2.97E-10	9.38E-09	4.47E-09	15
BP	GO:20012	11/88	165/1867(	3.27E-10	1.02E-08	4.88E-09	11
BP	GO:00075	13/88	270/1867(	4.15E-10	1.28E-08	6.12E-09	13
BP	GO:00016	16/88	461/1867(	4.18E-10	1.28E-08	6.12E-09	16
BP	GO:19030	12/88	218/1867(	4.47E-10	1.36E-08	6.49E-09	12
BP	GO:00018	16/88	464/1867(	4.59E-10	1.39E-08	6.61E-09	16
BP	GO:00106	11/88	171/1867(	4.79E-10	1.43E-08	6.84E-09	11
BP	GO:00030	11/88	173/1867(	5.42E-10	1.61E-08	7.68E-09	11
BP	GO:00224	15/88	403/1867(	6.00E-10	1.75E-08	8.35E-09	15
BP	GO:00457	15/88	403/1867(	6.00E-10	1.75E-08	8.35E-09	15
BP	GO:00327	8/88	64/18670	6.28E-10	1.82E-08	8.68E-09	8
BP	GO:20012	11/88	179/1867(	7.80E-10	2.24E-08	1.07E-08	11
BP	GO:00714	12/88	234/1867(	1.00E-09	2.86E-08	1.36E-08	12
BP	GO:20000	11/88	184/1867(	1.05E-09	2.95E-08	1.41E-08	11
BP	GO:00486	9/88	101/1867(	1.11E-09	3.10E-08	1.48E-08	9
BP	GO:00094	10/88	141/1867(	1.20E-09	3.32E-08	1.59E-08	10
BP	GO:00320	9/88	103/1867(	1.32E-09	3.64E-08	1.74E-08	9
BP	GO:19010	7/88	44/18670	1.35E-09	3.69E-08	1.76E-08	7
BP	GO:00321	14/88	365/1867(	1.62E-09	4.39E-08	2.09E-08	14
BP	GO:00301	14/88	368/1867(	1.79E-09	4.81E-08	2.29E-08	14
BP	GO:00435	10/88	147/1867(	1.80E-09	4.81E-08	2.29E-08	10
BP	GO:00602	15/88	437/1867(	1.82E-09	4.83E-08	2.30E-08	15
BP	GO:00069	7/88	47/18670	2.19E-09	5.72E-08	2.73E-08	7
BP	GO:00616	7/88	47/18670	2.19E-09	5.72E-08	2.73E-08	7
BP	GO:00346	13/88	312/1867(	2.41E-09	6.26E-08	2.99E-08	13
BP	GO:00469	8/88	76/18670	2.56E-09	6.59E-08	3.14E-08	8
BP	GO:00508	13/88	314/1867(	2.61E-09	6.66E-08	3.18E-08	13
BP	GO:00224	12/88	255/1867(	2.67E-09	6.77E-08	3.23E-08	12

BP	GO:19028	11/88	202/1867(	2.80E-09	7.06E-08	3.37E-08	11
BP	GO:00512	7/88	49/18670	2.97E-09	7.42E-08	3.54E-08	7
BP	GO:19049	15/88	456/1867(	3.24E-09	8.05E-08	3.84E-08	15
BP	GO:00321	13/88	323/1867(	3.66E-09	9.04E-08	4.31E-08	13
BP	GO:19038	13/88	324/1867(	3.80E-09	9.27E-08	4.42E-08	13
BP	GO:00076	11/88	208/1867(	3.81E-09	9.27E-08	4.42E-08	11
BP	GO:00075	7/88	51/18670	3.97E-09	9.45E-08	4.51E-08	7
BP	GO:00481	7/88	51/18670	3.97E-09	9.45E-08	4.51E-08	7
BP	GO:00509	7/88	51/18670	3.97E-09	9.45E-08	4.51E-08	7
BP	GO:00082	13/88	331/1867(	4.92E-09	1.16E-07	5.55E-08	13
BP	GO:00018	6/88	30/18670	5.00E-09	1.18E-07	5.61E-08	6
BP	GO:00433	11/88	217/1867(	5.93E-09	1.38E-07	6.59E-08	11
BP	GO:00343	5/88	15/18670	6.00E-09	1.38E-07	6.59E-08	5
BP	GO:00973	7/88	54/18670	6.00E-09	1.38E-07	6.59E-08	7
BP	GO:00025	11/88	220/1867(	6.85E-09	1.57E-07	7.47E-08	11
BP	GO:00086	8/88	86/18670	6.93E-09	1.57E-07	7.47E-08	8
BP	GO:00905	8/88	86/18670	6.93E-09	1.57E-07	7.47E-08	8
BP	GO:00192	14/88	410/1867(	7.14E-09	1.60E-07	7.65E-08	14
BP	GO:00507	15/88	485/1867(	7.44E-09	1.65E-07	7.87E-08	15
BP	GO:00512	15/88	485/1867(	7.44E-09	1.65E-07	7.87E-08	15
BP	GO:00103	7/88	56/18670	7.80E-09	1.72E-07	8.19E-08	7
BP	GO:00486	13/88	347/1867(	8.64E-09	1.89E-07	9.02E-08	13
BP	GO:00512	6/88	33/18670	9.22E-09	2.01E-07	9.57E-08	6
BP	GO:00018	11/88	227/1867(	9.48E-09	2.05E-07	9.78E-08	11
BP	GO:00316	7/88	58/18670	1.00E-08	2.16E-07	1.03E-07	7
BP	GO:00430	14/88	422/1867(	1.03E-08	2.20E-07	1.05E-07	14
BP	GO:00015	13/88	354/1867(	1.10E-08	2.33E-07	1.11E-07	13
BP	GO:00018	7/88	59/18670	1.13E-08	2.39E-07	1.14E-07	7
BP	GO:00106	12/88	291/1867(	1.17E-08	2.46E-07	1.17E-07	12
BP	GO:00487	10/88	179/1867(	1.21E-08	2.50E-07	1.19E-07	10
BP	GO:19030	10/88	179/1867(	1.21E-08	2.50E-07	1.19E-07	10
BP	GO:00094	12/88	292/1867(	1.22E-08	2.51E-07	1.20E-07	12
BP	GO:19021	7/88	60/18670	1.28E-08	2.62E-07	1.25E-07	7
BP	GO:00070	9/88	134/1867(	1.36E-08	2.78E-07	1.32E-07	9
BP	GO:00082	10/88	182/1867(	1.41E-08	2.87E-07	1.37E-07	10
BP	GO:00900	12/88	298/1867(	1.53E-08	3.07E-07	1.47E-07	12
BP	GO:00181	12/88	299/1867(	1.58E-08	3.17E-07	1.51E-07	12
BP	GO:19026	7/88	62/18670	1.62E-08	3.22E-07	1.53E-07	7
BP	GO:00346	9/88	137/1867(	1.66E-08	3.28E-07	1.56E-07	9
BP	GO:19054	10/88	187/1867(	1.83E-08	3.61E-07	1.72E-07	10
BP	GO:00357	7/88	64/18670	2.03E-08	3.97E-07	1.89E-07	7
BP	GO:00421	11/88	248/1867(	2.37E-08	4.61E-07	2.20E-07	11
BP	GO:00308	9/88	143/1867(	2.41E-08	4.66E-07	2.22E-07	9
BP	GO:00459	11/88	249/1867(	2.47E-08	4.75E-07	2.26E-07	11
BP	GO:00454	7/88	66/18670	2.52E-08	4.77E-07	2.28E-07	7
BP	GO:19021	7/88	66/18670	2.52E-08	4.77E-07	2.28E-07	7
BP	GO:19057	7/88	66/18670	2.52E-08	4.77E-07	2.28E-07	7
BP	GO:00026	13/88	380/1867(	2.54E-08	4.78E-07	2.28E-07	13
BP	GO:19020	6/88	39/18670	2.66E-08	4.98E-07	2.37E-07	6
BP	GO:00094	12/88	314/1867(	2.72E-08	5.07E-07	2.42E-07	12
BP	GO:00421	7/88	67/18670	2.80E-08	5.20E-07	2.48E-07	7
BP	GO:00026	10/88	196/1867(	2.87E-08	5.28E-07	2.52E-07	10
BP	GO:00551	9/88	146/1867(	2.88E-08	5.29E-07	2.52E-07	9
BP	GO:00086	8/88	104/1867(	3.14E-08	5.73E-07	2.73E-07	8
BP	GO:00610	9/88	148/1867(	3.25E-08	5.89E-07	2.81E-07	9
BP	GO:00426	7/88	69/18670	3.45E-08	6.23E-07	2.97E-07	7
BP	GO:00182	12/88	322/1867(	3.59E-08	6.45E-07	3.08E-07	12
BP	GO:19028	6/88	41/18670	3.63E-08	6.49E-07	3.10E-07	6

BP	GO:00508	10/88	202/1867(	3.81E-08	6.78E-07	3.23E-07	10
BP	GO:00508	13/88	394/1867(	3.88E-08	6.86E-07	3.27E-07	13
BP	GO:00513	8/88	107/1867(	3.93E-08	6.91E-07	3.30E-07	8
BP	GO:00105	12/88	327/1867(	4.25E-08	7.45E-07	3.55E-07	12
BP	GO:00015	13/88	398/1867(	4.36E-08	7.61E-07	3.63E-07	13
BP	GO:00611	7/88	72/18670	4.66E-08	8.08E-07	3.85E-07	7
BP	GO:00020	10/88	207/1867(	4.81E-08	8.25E-07	3.93E-07	10
BP	GO:00714	10/88	207/1867(	4.81E-08	8.25E-07	3.93E-07	10
BP	GO:00086	6/88	43/18670	4.89E-08	8.31E-07	3.96E-07	6
BP	GO:00454	6/88	43/18670	4.89E-08	8.31E-07	3.96E-07	6
BP	GO:00712	10/88	209/1867(	5.26E-08	8.90E-07	4.25E-07	10
BP	GO:00512	12/88	334/1867(	5.36E-08	9.03E-07	4.31E-07	12
BP	GO:00434	11/88	269/1867(	5.44E-08	9.11E-07	4.35E-07	11
BP	GO:19044	6/88	44/18670	5.64E-08	9.41E-07	4.49E-07	6
BP	GO:00075	12/88	336/1867(	5.73E-08	9.51E-07	4.53E-07	12
BP	GO:00466	11/88	272/1867(	6.09E-08	1.01E-06	4.80E-07	11
BP	GO:00329	11/88	274/1867(	6.56E-08	1.08E-06	5.14E-07	11
BP	GO:00075	12/88	341/1867(	6.73E-08	1.10E-06	5.25E-07	12
BP	GO:00508	12/88	342/1867(	6.95E-08	1.13E-06	5.40E-07	12
BP	GO:00319	9/88	162/1867(	7.10E-08	1.15E-06	5.49E-07	9
BP	GO:00362	10/88	217/1867(	7.49E-08	1.20E-06	5.74E-07	10
BP	GO:00509	10/88	217/1867(	7.49E-08	1.20E-06	5.74E-07	10
BP	GO:00000	11/88	279/1867(	7.88E-08	1.26E-06	6.01E-07	11
BP	GO:00301	7/88	79/18670	8.93E-08	1.41E-06	6.72E-07	7
BP	GO:00435	7/88	79/18670	8.93E-08	1.41E-06	6.72E-07	7
BP	GO:00712	7/88	79/18670	8.93E-08	1.41E-06	6.72E-07	7
BP	GO:19000	7/88	80/18670	9.75E-08	1.53E-06	7.29E-07	7
BP	GO:00513	11/88	285/1867(	9.77E-08	1.53E-06	7.29E-07	11
BP	GO:19054	8/88	122/1867(	1.10E-07	1.70E-06	8.10E-07	8
BP	GO:00350	6/88	49/18670	1.10E-07	1.70E-06	8.10E-07	6
BP	GO:00420	11/88	290/1867(	1.17E-07	1.80E-06	8.57E-07	11
BP	GO:00094	6/88	50/18670	1.24E-07	1.90E-06	9.05E-07	6
BP	GO:00511	8/88	124/1867(	1.24E-07	1.90E-06	9.05E-07	8
BP	GO:00313	12/88	361/1867(	1.25E-07	1.90E-06	9.05E-07	12
BP	GO:00069	7/88	83/18670	1.26E-07	1.90E-06	9.05E-07	7
BP	GO:19007	5/88	26/18670	1.26E-07	1.90E-06	9.05E-07	5
BP	GO:19007	5/88	26/18670	1.26E-07	1.90E-06	9.05E-07	5
BP	GO:20001	8/88	125/1867(	1.32E-07	1.98E-06	9.45E-07	8
BP	GO:00508	7/88	84/18670	1.37E-07	2.04E-06	9.73E-07	7
BP	GO:00424	10/88	232/1867(	1.40E-07	2.08E-06	9.90E-07	10
BP	GO:00068	12/88	365/1867(	1.41E-07	2.08E-06	9.92E-07	12
BP	GO:00019	9/88	176/1867(	1.44E-07	2.13E-06	1.01E-06	9
BP	GO:00448	11/88	298/1867(	1.53E-07	2.24E-06	1.07E-06	11
BP	GO:00706	11/88	298/1867(	1.53E-07	2.24E-06	1.07E-06	11
BP	GO:00705	7/88	86/18670	1.61E-07	2.34E-06	1.12E-06	7
BP	GO:00714	9/88	180/1867(	1.75E-07	2.53E-06	1.21E-06	9
BP	GO:00105	9/88	181/1867(	1.83E-07	2.64E-06	1.26E-06	9
BP	GO:00603	11/88	304/1867(	1.87E-07	2.69E-06	1.28E-06	11
BP	GO:00971	7/88	88/18670	1.89E-07	2.69E-06	1.28E-06	7
BP	GO:19040	7/88	88/18670	1.89E-07	2.69E-06	1.28E-06	7
BP	GO:19028	8/88	131/1867(	1.90E-07	2.69E-06	1.28E-06	8
BP	GO:00525	13/88	452/1867(	1.91E-07	2.69E-06	1.28E-06	13
BP	GO:19046	6/88	54/18670	1.99E-07	2.80E-06	1.33E-06	6
BP	GO:00420	9/88	184/1867(	2.11E-07	2.94E-06	1.40E-06	9
BP	GO:00434	9/88	184/1867(	2.11E-07	2.94E-06	1.40E-06	9
BP	GO:19030	7/88	90/18670	2.21E-07	3.06E-06	1.46E-06	7
BP	GO:00106	4/88	12/18670	2.22E-07	3.06E-06	1.46E-06	4
BP	GO:00015	6/88	55/18670	2.22E-07	3.06E-06	1.46E-06	6

BP	GO:00105	5/88	29/18670	2.25E-07	3.09E-06	1.48E-06	5
BP	GO:00485	8/88	134/1867(	2.26E-07	3.10E-06	1.48E-06	8
BP	GO:00303	9/88	186/1867(	2.31E-07	3.15E-06	1.50E-06	9
BP	GO:00066	12/88	383/1867(	2.36E-07	3.21E-06	1.53E-06	12
BP	GO:00509	8/88	135/1867(	2.40E-07	3.24E-06	1.55E-06	8
BP	GO:19016	12/88	385/1867(	2.50E-07	3.36E-06	1.60E-06	12
BP	GO:00726	8/88	137/1867(	2.69E-07	3.60E-06	1.72E-06	8
BP	GO:00435	6/88	57/18670	2.76E-07	3.67E-06	1.75E-06	6
BP	GO:19034	6/88	57/18670	2.76E-07	3.67E-06	1.75E-06	6
BP	GO:00332	7/88	93/18670	2.77E-07	3.67E-06	1.75E-06	7
BP	GO:00457	12/88	389/1867(	2.79E-07	3.69E-06	1.76E-06	12
BP	GO:00019	9/88	191/1867(	2.90E-07	3.80E-06	1.81E-06	9
BP	GO:00425	9/88	191/1867(	2.90E-07	3.80E-06	1.81E-06	9
BP	GO:00331	8/88	139/1867(	3.00E-07	3.92E-06	1.87E-06	8
BP	GO:00327	6/88	58/18670	3.07E-07	3.98E-06	1.90E-06	6
BP	GO:19020	6/88	58/18670	3.07E-07	3.98E-06	1.90E-06	6
BP	GO:00109	12/88	394/1867(	3.20E-07	4.13E-06	1.97E-06	12
BP	GO:00705	8/88	141/1867(	3.35E-07	4.31E-06	2.06E-06	8
BP	GO:00977	6/88	59/18670	3.40E-07	4.35E-06	2.07E-06	6
BP	GO:20003	6/88	59/18670	3.40E-07	4.35E-06	2.07E-06	6
BP	GO:00068	10/88	256/1867(	3.48E-07	4.43E-06	2.11E-06	10
BP	GO:00159	7/88	97/18670	3.70E-07	4.69E-06	2.24E-06	7
BP	GO:00108	12/88	400/1867(	3.76E-07	4.75E-06	2.27E-06	12
BP	GO:00459	5/88	32/18670	3.78E-07	4.76E-06	2.27E-06	5
BP	GO:20012	7/88	98/18670	3.97E-07	4.98E-06	2.37E-06	7
BP	GO:20012	6/88	61/18670	4.16E-07	5.21E-06	2.48E-06	6
BP	GO:00016	11/88	330/1867(	4.24E-07	5.29E-06	2.52E-06	11
BP	GO:00513	8/88	146/1867(	4.37E-07	5.43E-06	2.59E-06	8
BP	GO:00468	6/88	62/18670	4.59E-07	5.68E-06	2.71E-06	6
BP	GO:00303	11/88	334/1867(	4.78E-07	5.90E-06	2.81E-06	11
BP	GO:00140	8/88	148/1867(	4.85E-07	5.96E-06	2.84E-06	8
BP	GO:19034	7/88	101/1867(	4.87E-07	5.97E-06	2.85E-06	7
BP	GO:00092	8/88	149/1867(	5.11E-07	6.21E-06	2.96E-06	8
BP	GO:00140	8/88	149/1867(	5.11E-07	6.21E-06	2.96E-06	8
BP	GO:00105	5/88	34/18670	5.18E-07	6.28E-06	2.99E-06	5
BP	GO:00459	11/88	338/1867(	5.38E-07	6.49E-06	3.10E-06	11
BP	GO:00061	9/88	206/1867(	5.48E-07	6.53E-06	3.11E-06	9
BP	GO:00506	9/88	206/1867(	5.48E-07	6.53E-06	3.11E-06	9
BP	GO:00069	13/88	496/1867(	5.48E-07	6.53E-06	3.11E-06	13
BP	GO:00619	13/88	496/1867(	5.48E-07	6.53E-06	3.11E-06	13
BP	GO:00506	9/88	208/1867(	5.94E-07	7.05E-06	3.36E-06	9
BP	GO:00018	5/88	35/18670	6.02E-07	7.13E-06	3.40E-06	5
BP	GO:00725	6/88	65/18670	6.10E-07	7.19E-06	3.43E-06	6
BP	GO:00329	9/88	209/1867(	6.18E-07	7.27E-06	3.47E-06	9
BP	GO:00435	10/88	273/1867(	6.27E-07	7.35E-06	3.51E-06	10
BP	GO:00331	7/88	105/1867(	6.35E-07	7.42E-06	3.54E-06	7
BP	GO:00098	12/88	423/1867(	6.79E-07	7.91E-06	3.77E-06	12
BP	GO:00105	5/88	36/18670	6.97E-07	8.03E-06	3.83E-06	5
BP	GO:19010	5/88	36/18670	6.97E-07	8.03E-06	3.83E-06	5
BP	GO:20012	5/88	36/18670	6.97E-07	8.03E-06	3.83E-06	5
BP	GO:00435	8/88	156/1867(	7.24E-07	8.32E-06	3.97E-06	8
BP	GO:00486	6/88	67/18670	7.31E-07	8.37E-06	3.99E-06	6
BP	GO:20001	11/88	349/1867(	7.37E-07	8.41E-06	4.01E-06	11
BP	GO:19059	8/88	157/1867(	7.60E-07	8.65E-06	4.12E-06	8
BP	GO:00432	9/88	215/1867(	7.83E-07	8.88E-06	4.23E-06	9
BP	GO:00305	10/88	280/1867(	7.90E-07	8.93E-06	4.26E-06	10
BP	GO:00434	8/88	158/1867(	7.98E-07	8.99E-06	4.29E-06	8
BP	GO:00100	9/88	218/1867(	8.79E-07	9.87E-06	4.71E-06	9

BP	GO:00349	10/88	285/1867(	9.28E-07	1.04E-05	4.95E-06	10
BP	GO:00706	9/88	222/1867(	1.02E-06	1.14E-05	5.44E-06	9
BP	GO:00316	4/88	17/18670	1.05E-06	1.16E-05	5.55E-06	4
BP	GO:00458	11/88	363/1867(	1.08E-06	1.20E-05	5.73E-06	11
BP	GO:00070	8/88	165/1867(	1.11E-06	1.22E-05	5.81E-06	8
BP	GO:00026	7/88	114/1867(	1.11E-06	1.22E-05	5.81E-06	7
BP	GO:00420	7/88	114/1867(	1.11E-06	1.22E-05	5.81E-06	7
BP	GO:00713	10/88	291/1867(	1.12E-06	1.23E-05	5.85E-06	10
BP	GO:00518	6/88	72/18670	1.12E-06	1.23E-05	5.85E-06	6
BP	GO:19019	12/88	444/1867(	1.13E-06	1.23E-05	5.86E-06	12
BP	GO:00329	7/88	115/1867(	1.18E-06	1.28E-05	6.09E-06	7
BP	GO:00068	6/88	73/18670	1.22E-06	1.32E-05	6.29E-06	6
BP	GO:01500	6/88	75/18670	1.43E-06	1.54E-05	7.36E-06	6
BP	GO:00162	8/88	171/1867(	1.45E-06	1.56E-05	7.43E-06	8
BP	GO:00610	6/88	76/18670	1.55E-06	1.66E-05	7.91E-06	6
BP	GO:00226	7/88	120/1867(	1.57E-06	1.67E-05	7.99E-06	7
BP	GO:00097	9/88	235/1867(	1.64E-06	1.75E-05	8.32E-06	9
BP	GO:00070	9/88	237/1867(	1.76E-06	1.87E-05	8.90E-06	9
BP	GO:00420	7/88	123/1867(	1.85E-06	1.96E-05	9.34E-06	7
BP	GO:00512	11/88	384/1867(	1.87E-06	1.98E-05	9.42E-06	11
BP	GO:20001	9/88	239/1867(	1.88E-06	1.98E-05	9.45E-06	9
BP	GO:00140	7/88	124/1867(	1.95E-06	2.04E-05	9.72E-06	7
BP	GO:00421	7/88	124/1867(	1.95E-06	2.04E-05	9.72E-06	7
BP	GO:00423	12/88	468/1867(	1.96E-06	2.04E-05	9.72E-06	12
BP	GO:00454	7/88	125/1867(	2.06E-06	2.14E-05	1.02E-05	7
BP	GO:00435	8/88	180/1867(	2.13E-06	2.20E-05	1.05E-05	8
BP	GO:00713	8/88	180/1867(	2.13E-06	2.20E-05	1.05E-05	8
BP	GO:00480	8/88	181/1867(	2.22E-06	2.29E-05	1.09E-05	8
BP	GO:00323	7/88	127/1867(	2.29E-06	2.36E-05	1.12E-05	7
BP	GO:00488	9/88	246/1867(	2.39E-06	2.45E-05	1.17E-05	9
BP	GO:00105	7/88	128/1867(	2.42E-06	2.46E-05	1.17E-05	7
BP	GO:00326	6/88	82/18670	2.42E-06	2.46E-05	1.17E-05	6
BP	GO:00069	5/88	46/18670	2.44E-06	2.46E-05	1.17E-05	5
BP	GO:00226	5/88	46/18670	2.44E-06	2.46E-05	1.17E-05	5
BP	GO:00457	5/88	46/18670	2.44E-06	2.46E-05	1.17E-05	5
BP	GO:00604	5/88	46/18670	2.44E-06	2.46E-05	1.17E-05	5
BP	GO:00480	8/88	184/1867(	2.51E-06	2.52E-05	1.20E-05	8
BP	GO:00400	11/88	396/1867(	2.52E-06	2.52E-05	1.20E-05	11
BP	GO:19019	12/88	480/1867(	2.54E-06	2.53E-05	1.21E-05	12
BP	GO:00347	7/88	129/1867(	2.55E-06	2.53E-05	1.21E-05	7
BP	GO:00550	4/88	21/18670	2.59E-06	2.57E-05	1.23E-05	4
BP	GO:00459	6/88	83/18670	2.60E-06	2.58E-05	1.23E-05	6
BP	GO:00506	7/88	130/1867(	2.68E-06	2.65E-05	1.26E-05	7
BP	GO:20012	5/88	47/18670	2.72E-06	2.68E-05	1.28E-05	5
BP	GO:00329	7/88	131/1867(	2.82E-06	2.77E-05	1.32E-05	7
BP	GO:19047	6/88	85/18670	2.99E-06	2.92E-05	1.39E-05	6
BP	GO:19908	6/88	85/18670	2.99E-06	2.92E-05	1.39E-05	6
BP	GO:00901	5/88	48/18670	3.03E-06	2.95E-05	1.40E-05	5
BP	GO:00519	9/88	254/1867(	3.11E-06	3.01E-05	1.44E-05	9
BP	GO:00108	4/88	22/18670	3.16E-06	3.05E-05	1.45E-05	4
BP	GO:00713	4/88	22/18670	3.16E-06	3.05E-05	1.45E-05	4
BP	GO:00424	6/88	86/18670	3.21E-06	3.09E-05	1.47E-05	6
BP	GO:00323	7/88	134/1867(	3.28E-06	3.14E-05	1.50E-05	7
BP	GO:00466	7/88	134/1867(	3.28E-06	3.14E-05	1.50E-05	7
BP	GO:00507	9/88	256/1867(	3.31E-06	3.16E-05	1.51E-05	9
BP	GO:00973	5/88	49/18670	3.36E-06	3.20E-05	1.52E-05	5
BP	GO:00018	6/88	87/18670	3.43E-06	3.26E-05	1.55E-05	6
BP	GO:00512	10/88	330/1867(	3.46E-06	3.28E-05	1.56E-05	10

BP	GO:00901	10/88	332/1867(	3.65E-06	3.45E-05	1.64E-05	10
BP	GO:00510	9/88	261/1867(	3.88E-06	3.65E-05	1.74E-05	9
BP	GO:00326	6/88	89/18670	3.92E-06	3.68E-05	1.76E-05	6
BP	GO:00085	7/88	138/1867(	3.99E-06	3.74E-05	1.78E-05	7
BP	GO:00465	7/88	139/1867(	4.18E-06	3.90E-05	1.86E-05	7
BP	GO:00706	7/88	139/1867(	4.18E-06	3.90E-05	1.86E-05	7
BP	GO:00512	11/88	418/1867(	4.23E-06	3.93E-05	1.88E-05	11
BP	GO:00310	8/88	198/1867(	4.32E-06	4.01E-05	1.91E-05	8
BP	GO:00149	6/88	91/18670	4.46E-06	4.10E-05	1.96E-05	6
BP	GO:00456	6/88	91/18670	4.46E-06	4.10E-05	1.96E-05	6
BP	GO:00603	6/88	91/18670	4.46E-06	4.10E-05	1.96E-05	6
BP	GO:00343	8/88	199/1867(	4.49E-06	4.11E-05	1.96E-05	8
BP	GO:00468	9/88	266/1867(	4.52E-06	4.14E-05	1.97E-05	9
BP	GO:00466	4/88	24/18670	4.56E-06	4.16E-05	1.98E-05	4
BP	GO:00313	9/88	268/1867(	4.81E-06	4.37E-05	2.09E-05	9
BP	GO:00381	7/88	142/1867(	4.82E-06	4.37E-05	2.09E-05	7
BP	GO:00525	11/88	425/1867(	4.96E-06	4.49E-05	2.14E-05	11
BP	GO:00301	5/88	53/18670	4.98E-06	4.50E-05	2.14E-05	5
BP	GO:00364	6/88	93/18670	5.06E-06	4.56E-05	2.17E-05	6
BP	GO:00485	11/88	428/1867(	5.30E-06	4.76E-05	2.27E-05	11
BP	GO:00352	8/88	204/1867(	5.39E-06	4.83E-05	2.30E-05	8
BP	GO:00019	11/88	429/1867(	5.42E-06	4.84E-05	2.31E-05	11
BP	GO:00027	5/88	54/18670	5.47E-06	4.86E-05	2.32E-05	5
BP	GO:19000	5/88	54/18670	5.47E-06	4.86E-05	2.32E-05	5
BP	GO:00719	7/88	145/1867(	5.53E-06	4.91E-05	2.34E-05	7
BP	GO:00425	7/88	146/1867(	5.79E-06	5.11E-05	2.44E-05	7
BP	GO:00458	7/88	146/1867(	5.79E-06	5.11E-05	2.44E-05	7
BP	GO:00432	9/88	275/1867(	5.92E-06	5.21E-05	2.49E-05	9
BP	GO:00192	6/88	96/18670	6.09E-06	5.33E-05	2.54E-05	6
BP	GO:00480	6/88	96/18670	6.09E-06	5.33E-05	2.54E-05	6
BP	GO:00301	4/88	26/18670	6.36E-06	5.53E-05	2.64E-05	4
BP	GO:00328	4/88	26/18670	6.36E-06	5.53E-05	2.64E-05	4
BP	GO:19000	4/88	26/18670	6.36E-06	5.53E-05	2.64E-05	4
BP	GO:00315	10/88	354/1867(	6.43E-06	5.58E-05	2.66E-05	10
BP	GO:00604	9/88	278/1867(	6.47E-06	5.59E-05	2.67E-05	9
BP	GO:00069	5/88	56/18670	6.56E-06	5.66E-05	2.70E-05	5
BP	GO:00975	8/88	210/1867(	6.67E-06	5.74E-05	2.74E-05	8
BP	GO:00199	11/88	439/1867(	6.74E-06	5.79E-05	2.76E-05	11
BP	GO:00301	9/88	281/1867(	7.05E-06	6.04E-05	2.88E-05	9
BP	GO:00508	5/88	57/18670	7.16E-06	6.07E-05	2.90E-05	5
BP	GO:00713	5/88	57/18670	7.16E-06	6.07E-05	2.90E-05	5
BP	GO:00724	5/88	57/18670	7.16E-06	6.07E-05	2.90E-05	5
BP	GO:19024	5/88	57/18670	7.16E-06	6.07E-05	2.90E-05	5
BP	GO:00468	4/88	27/18670	7.44E-06	6.27E-05	2.99E-05	4
BP	GO:00508	4/88	27/18670	7.44E-06	6.27E-05	2.99E-05	4
BP	GO:19021	4/88	27/18670	7.44E-06	6.27E-05	2.99E-05	4
BP	GO:00192	5/88	58/18670	7.81E-06	6.54E-05	3.12E-05	5
BP	GO:19037	5/88	58/18670	7.81E-06	6.54E-05	3.12E-05	5
BP	GO:00301	7/88	153/1867(	7.88E-06	6.57E-05	3.13E-05	7
BP	GO:00458	7/88	153/1867(	7.88E-06	6.57E-05	3.13E-05	7
BP	GO:00181	10/88	363/1867(	8.01E-06	6.67E-05	3.18E-05	10
BP	GO:00000	8/88	216/1867(	8.19E-06	6.80E-05	3.24E-05	8
BP	GO:00060	10/88	364/1867(	8.21E-06	6.80E-05	3.24E-05	10
BP	GO:00724	5/88	59/18670	8.50E-06	6.99E-05	3.33E-05	5
BP	GO:19024	5/88	59/18670	8.50E-06	6.99E-05	3.33E-05	5
BP	GO:19024	5/88	59/18670	8.50E-06	6.99E-05	3.33E-05	5
BP	GO:00182	10/88	366/1867(	8.61E-06	7.05E-05	3.36E-05	10
BP	GO:00327	4/88	28/18670	8.65E-06	7.05E-05	3.36E-05	4

BP	GO:00362	4/88	28/18670	8.65E-06	7.05E-05	3.36E-05	4
BP	GO:00427	4/88	28/18670	8.65E-06	7.05E-05	3.36E-05	4
BP	GO:00072	7/88	156/1867(	8.95E-06	7.26E-05	3.46E-05	7
BP	GO:00421	7/88	156/1867(	8.95E-06	7.26E-05	3.46E-05	7
BP	GO:00432	7/88	157/1867(	9.33E-06	7.55E-05	3.60E-05	7
BP	GO:00148	6/88	104/1867(	9.67E-06	7.77E-05	3.71E-05	6
BP	GO:00468	6/88	104/1867(	9.67E-06	7.77E-05	3.71E-05	6
BP	GO:00718	6/88	104/1867(	9.67E-06	7.77E-05	3.71E-05	6
BP	GO:00903	5/88	61/18670	1.00E-05	8.04E-05	3.83E-05	5
BP	GO:00017	10/88	373/1867(	1.02E-05	8.12E-05	3.87E-05	10
BP	GO:00466	7/88	160/1867(	1.06E-05	8.43E-05	4.02E-05	7
BP	GO:00305	8/88	224/1867(	1.07E-05	8.50E-05	4.06E-05	8
BP	GO:00447	6/88	106/1867(	1.08E-05	8.57E-05	4.09E-05	6
BP	GO:00022	5/88	62/18670	1.09E-05	8.59E-05	4.10E-05	5
BP	GO:00702	5/88	62/18670	1.09E-05	8.59E-05	4.10E-05	5
BP	GO:00071	8/88	225/1867(	1.10E-05	8.70E-05	4.15E-05	8
BP	GO:00157	7/88	162/1867(	1.15E-05	9.01E-05	4.30E-05	7
BP	GO:00017	4/88	30/18670	1.15E-05	9.01E-05	4.30E-05	4
BP	GO:00714	4/88	30/18670	1.15E-05	9.01E-05	4.30E-05	4
BP	GO:00315	5/88	63/18670	1.18E-05	9.17E-05	4.37E-05	5
BP	GO:00448	5/88	63/18670	1.18E-05	9.17E-05	4.37E-05	5
BP	GO:00016	3/88	10/18670	1.19E-05	9.19E-05	4.38E-05	3
BP	GO:00320	3/88	10/18670	1.19E-05	9.19E-05	4.38E-05	3
BP	GO:00519	3/88	10/18670	1.19E-05	9.19E-05	4.38E-05	3
BP	GO:00459	7/88	163/1867(	1.19E-05	9.22E-05	4.40E-05	7
BP	GO:00432	11/88	467/1867(	1.20E-05	9.29E-05	4.43E-05	11
BP	GO:00358	7/88	164/1867(	1.24E-05	9.55E-05	4.55E-05	7
BP	GO:00105	8/88	229/1867(	1.25E-05	9.63E-05	4.59E-05	8
BP	GO:00447	5/88	64/18670	1.27E-05	9.74E-05	4.64E-05	5
BP	GO:00101	4/88	31/18670	1.32E-05	0.0001	4.78E-05	4
BP	GO:00346	4/88	31/18670	1.32E-05	0.0001	4.78E-05	4
BP	GO:19028	4/88	31/18670	1.32E-05	0.0001	4.78E-05	4
BP	GO:00507	11/88	472/1867(	1.33E-05	0.000101	4.82E-05	11
BP	GO:00161	6/88	110/1867(	1.33E-05	0.000101	4.83E-05	6
BP	GO:00976	7/88	166/1867(	1.34E-05	0.000102	4.84E-05	7
BP	GO:00091	10/88	386/1867(	1.37E-05	0.000103	4.92E-05	10
BP	GO:19015	7/88	167/1867(	1.40E-05	0.000105	5.01E-05	7
BP	GO:00098	9/88	308/1867(	1.47E-05	0.00011	5.27E-05	9
BP	GO:00019	6/88	112/1867(	1.48E-05	0.000111	5.29E-05	6
BP	GO:19012	10/88	390/1867(	1.49E-05	0.000112	5.33E-05	10
BP	GO:00507	7/88	169/1867(	1.51E-05	0.000113	5.37E-05	7
BP	GO:00064	8/88	235/1867(	1.51E-05	0.000113	5.37E-05	8
BP	GO:00432	6/88	113/1867(	1.56E-05	0.000116	5.52E-05	6
BP	GO:00197	5/88	67/18670	1.59E-05	0.000118	5.63E-05	5
BP	GO:00468	3/88	11/18670	1.62E-05	0.00012	5.72E-05	3
BP	GO:00607	3/88	11/18670	1.62E-05	0.00012	5.72E-05	3
BP	GO:00468	9/88	312/1867(	1.63E-05	0.00012	5.72E-05	9
BP	GO:00066	6/88	114/1867(	1.64E-05	0.00012	5.73E-05	6
BP	GO:00427	6/88	114/1867(	1.64E-05	0.00012	5.73E-05	6
BP	GO:00100	4/88	33/18670	1.70E-05	0.000124	5.94E-05	4
BP	GO:00199	5/88	68/18670	1.71E-05	0.000125	5.97E-05	5
BP	GO:00075	6/88	116/1867(	1.81E-05	0.000132	6.28E-05	6
BP	GO:19001	6/88	116/1867(	1.81E-05	0.000132	6.28E-05	6
BP	GO:00105	6/88	117/1867(	1.90E-05	0.000137	6.55E-05	6
BP	GO:00109	6/88	117/1867(	1.90E-05	0.000137	6.55E-05	6
BP	GO:19003	6/88	117/1867(	1.90E-05	0.000137	6.55E-05	6
BP	GO:00432	4/88	34/18670	1.92E-05	0.000139	6.61E-05	4
BP	GO:00518	7/88	176/1867(	1.96E-05	0.000141	6.74E-05	7

BP	GO:00341	5/88	70/18670	1.97E-05	0.000142	6.75E-05	5
BP	GO:00507	5/88	70/18670	1.97E-05	0.000142	6.75E-05	5
BP	GO:00518	8/88	244/1867(	1.98E-05	0.000142	6.77E-05	8
BP	GO:00308	6/88	118/1867(	1.99E-05	0.000142	6.79E-05	6
BP	GO:00484	7/88	177/1867(	2.03E-05	0.000145	6.92E-05	7
BP	GO:00099	9/88	322/1867(	2.09E-05	0.000149	7.09E-05	9
BP	GO:00069	3/88	12/18670	2.16E-05	0.000153	7.30E-05	3
BP	GO:00450	3/88	12/18670	2.16E-05	0.000153	7.30E-05	3
BP	GO:00972	8/88	247/1867(	2.16E-05	0.000153	7.30E-05	8
BP	GO:00510	7/88	179/1867(	2.19E-05	0.000154	7.34E-05	7
BP	GO:00713	7/88	179/1867(	2.19E-05	0.000154	7.34E-05	7
BP	GO:00030	6/88	120/1867(	2.19E-05	0.000154	7.34E-05	6
BP	GO:00067	6/88	120/1867(	2.19E-05	0.000154	7.34E-05	6
BP	GO:19019	8/88	248/1867(	2.23E-05	0.000156	7.44E-05	8
BP	GO:19037	5/88	72/18670	2.26E-05	0.000158	7.55E-05	5
BP	GO:00027	11/88	500/1867(	2.27E-05	0.000158	7.55E-05	11
BP	GO:00193	8/88	249/1867(	2.29E-05	0.00016	7.61E-05	8
BP	GO:00096	9/88	326/1867(	2.30E-05	0.00016	7.63E-05	9
BP	GO:00190	9/88	328/1867(	2.41E-05	0.000167	7.95E-05	9
BP	GO:00436	5/88	73/18670	2.42E-05	0.000167	7.95E-05	5
BP	GO:00724	5/88	73/18670	2.42E-05	0.000167	7.95E-05	5
BP	GO:00724	5/88	73/18670	2.42E-05	0.000167	7.95E-05	5
BP	GO:19035	5/88	73/18670	2.42E-05	0.000167	7.95E-05	5
BP	GO:00723	5/88	74/18670	2.59E-05	0.000177	8.44E-05	5
BP	GO:19001	5/88	74/18670	2.59E-05	0.000177	8.44E-05	5
BP	GO:19030	5/88	74/18670	2.59E-05	0.000177	8.44E-05	5
BP	GO:00300	10/88	416/1867(	2.60E-05	0.000177	8.46E-05	10
BP	GO:00328	4/88	37/18670	2.70E-05	0.000183	8.71E-05	4
BP	GO:00344	4/88	37/18670	2.70E-05	0.000183	8.71E-05	4
BP	GO:00430	4/88	37/18670	2.70E-05	0.000183	8.71E-05	4
BP	GO:00459	4/88	37/18670	2.70E-05	0.000183	8.71E-05	4
BP	GO:20002	4/88	37/18670	2.70E-05	0.000183	8.71E-05	4
BP	GO:00719	9/88	334/1867(	2.78E-05	0.000187	8.93E-05	9
BP	GO:00306	3/88	13/18670	2.80E-05	0.000187	8.93E-05	3
BP	GO:00903	3/88	13/18670	2.80E-05	0.000187	8.93E-05	3
BP	GO:00905	3/88	13/18670	2.80E-05	0.000187	8.93E-05	3
BP	GO:19029	3/88	13/18670	2.80E-05	0.000187	8.93E-05	3
BP	GO:00336	8/88	257/1867(	2.87E-05	0.000192	9.16E-05	8
BP	GO:00423	5/88	76/18670	2.94E-05	0.000196	9.37E-05	5
BP	GO:00323	4/88	38/18670	3.01E-05	0.0002	9.56E-05	4
BP	GO:00486	5/88	77/18670	3.14E-05	0.000209	9.95E-05	5
BP	GO:00026	6/88	128/1867(	3.16E-05	0.00021	1.00E-04	6
BP	GO:00345	8/88	262/1867(	3.30E-05	0.000218	0.000104	8
BP	GO:00510	10/88	428/1867(	3.31E-05	0.000219	0.000104	10
BP	GO:00714	7/88	191/1867(	3.31E-05	0.000219	0.000104	7
BP	GO:00723	5/88	78/18670	3.34E-05	0.00022	0.000105	5
BP	GO:00510	10/88	429/1867(	3.37E-05	0.000222	0.000106	10
BP	GO:00723	9/88	343/1867(	3.43E-05	0.000224	0.000107	9
BP	GO:00507	7/88	192/1867(	3.43E-05	0.000224	0.000107	7
BP	GO:00519	3/88	14/18670	3.55E-05	0.000232	0.000111	3
BP	GO:00714	4/88	40/18670	3.70E-05	0.000241	0.000115	4
BP	GO:00423	10/88	434/1867(	3.72E-05	0.000242	0.000115	10
BP	GO:19019	8/88	267/1867(	3.77E-05	0.000245	0.000117	8
BP	GO:00434	10/88	436/1867(	3.87E-05	0.000251	0.00012	10
BP	GO:00066	7/88	196/1867(	3.91E-05	0.000253	0.000121	7
BP	GO:00427	6/88	133/1867(	3.92E-05	0.000253	0.000121	6
BP	GO:00487	5/88	81/18670	4.01E-05	0.000259	0.000123	5
BP	GO:00109	7/88	197/1867(	4.04E-05	0.00026	0.000124	7

BP	GO:00459	8/88	270/1867(	4.08E-05	0.000262	0.000125	8
BP	GO:00425	4/88	41/18670	4.08E-05	0.000262	0.000125	4
BP	GO:00468	7/88	198/1867(	4.17E-05	0.000267	0.000127	7
BP	GO:19016	8/88	271/1867(	4.19E-05	0.000268	0.000128	8
BP	GO:00326	5/88	82/18670	4.25E-05	0.00027	0.000129	5
BP	GO:00346	5/88	82/18670	4.25E-05	0.00027	0.000129	5
BP	GO:00711	5/88	82/18670	4.25E-05	0.00027	0.000129	5
BP	GO:00300	9/88	353/1867(	4.28E-05	0.000272	0.00013	9
BP	GO:00328	8/88	272/1867(	4.30E-05	0.000272	0.00013	8
BP	GO:00170	7/88	199/1867(	4.31E-05	0.000272	0.00013	7
BP	GO:00307	3/88	15/18670	4.42E-05	0.000277	0.000132	3
BP	GO:00323	3/88	15/18670	4.42E-05	0.000277	0.000132	3
BP	GO:00450	3/88	15/18670	4.42E-05	0.000277	0.000132	3
BP	GO:00517	3/88	15/18670	4.42E-05	0.000277	0.000132	3
BP	GO:00464	6/88	137/1867(	4.63E-05	0.00029	0.000138	6
BP	GO:00149	5/88	84/18670	4.78E-05	0.000298	0.000142	5
BP	GO:00977	5/88	84/18670	4.78E-05	0.000298	0.000142	5
BP	GO:00067	4/88	43/18670	4.94E-05	0.000307	0.000146	4
BP	GO:00328	4/88	43/18670	4.94E-05	0.000307	0.000146	4
BP	GO:19046	4/88	43/18670	4.94E-05	0.000307	0.000146	4
BP	GO:00018	8/88	278/1867(	5.02E-05	0.000311	0.000148	8
BP	GO:00067	6/88	139/1867(	5.02E-05	0.000311	0.000148	6
BP	GO:00025	7/88	204/1867(	5.04E-05	0.000311	0.000148	7
BP	GO:00347	7/88	204/1867(	5.04E-05	0.000311	0.000148	7
BP	GO:00458	9/88	363/1867(	5.32E-05	0.000326	0.000155	9
BP	GO:00017	5/88	86/18670	5.35E-05	0.000326	0.000155	5
BP	GO:00069	5/88	86/18670	5.35E-05	0.000326	0.000155	5
BP	GO:00346	5/88	86/18670	5.35E-05	0.000326	0.000155	5
BP	GO:00466	4/88	44/18670	5.42E-05	0.000326	0.000155	4
BP	GO:00702	4/88	44/18670	5.42E-05	0.000326	0.000155	4
BP	GO:00020	3/88	16/18670	5.42E-05	0.000326	0.000155	3
BP	GO:00108	3/88	16/18670	5.42E-05	0.000326	0.000155	3
BP	GO:00420	3/88	16/18670	5.42E-05	0.000326	0.000155	3
BP	GO:00459	3/88	16/18670	5.42E-05	0.000326	0.000155	3
BP	GO:00507	3/88	16/18670	5.42E-05	0.000326	0.000155	3
BP	GO:00609	3/88	16/18670	5.42E-05	0.000326	0.000155	3
BP	GO:00702	3/88	16/18670	5.42E-05	0.000326	0.000155	3
BP	GO:00713	3/88	16/18670	5.42E-05	0.000326	0.000155	3
BP	GO:19004	3/88	16/18670	5.42E-05	0.000326	0.000155	3
BP	GO:00705	7/88	207/1867(	5.53E-05	0.000332	0.000158	7
BP	GO:00106	6/88	142/1867(	5.65E-05	0.000337	0.000161	6
BP	GO:19005	6/88	142/1867(	5.65E-05	0.000337	0.000161	6
BP	GO:00026	5/88	87/18670	5.66E-05	0.000337	0.000161	5
BP	GO:00140	5/88	87/18670	5.66E-05	0.000337	0.000161	5
BP	GO:00324	8/88	283/1867(	5.69E-05	0.000338	0.000161	8
BP	GO:00902	7/88	208/1867(	5.70E-05	0.000338	0.000161	7
BP	GO:00217	8/88	284/1867(	5.83E-05	0.000345	0.000165	8
BP	GO:00060	7/88	209/1867(	5.87E-05	0.000347	0.000165	7
BP	GO:00517	7/88	209/1867(	5.87E-05	0.000347	0.000165	7
BP	GO:00066	6/88	143/1867(	5.88E-05	0.000347	0.000165	6
BP	GO:00508	5/88	88/18670	5.97E-05	0.000352	0.000168	5
BP	GO:00069	9/88	369/1867(	6.03E-05	0.000355	0.000169	9
BP	GO:19040	6/88	144/1867(	6.11E-05	0.000359	0.000171	6
BP	GO:00442	8/88	286/1867(	6.12E-05	0.000359	0.000171	8
BP	GO:00230	10/88	462/1867(	6.28E-05	0.000367	0.000175	10
BP	GO:00463	10/88	462/1867(	6.28E-05	0.000367	0.000175	10
BP	GO:00464	5/88	89/18670	6.31E-05	0.000368	0.000175	5
BP	GO:00160	10/88	463/1867(	6.40E-05	0.000372	0.000178	10

BP	GO:00308	4/88	46/18670	6.46E-05	0.000374	0.000178	4
BP	GO:19003	4/88	46/18670	6.46E-05	0.000374	0.000178	4
BP	GO:19040	4/88	46/18670	6.46E-05	0.000374	0.000178	4
BP	GO:00305	3/88	17/18670	6.56E-05	0.000377	0.00018	3
BP	GO:00702	3/88	17/18670	6.56E-05	0.000377	0.00018	3
BP	GO:20012	3/88	17/18670	6.56E-05	0.000377	0.00018	3
BP	GO:00071	8/88	289/18670	6.59E-05	0.000377	0.00018	8
BP	GO:00022	6/88	146/18670	6.60E-05	0.000377	0.00018	6
BP	GO:00061	6/88	146/18670	6.60E-05	0.000377	0.00018	6
BP	GO:00352	6/88	146/18670	6.60E-05	0.000377	0.00018	6
BP	GO:19048	6/88	146/18670	6.60E-05	0.000377	0.00018	6
BP	GO:00022	6/88	147/18670	6.85E-05	0.000391	0.000186	6
BP	GO:00108	7/88	215/18670	7.02E-05	0.0004	0.000191	7
BP	GO:00488	4/88	47/18670	7.04E-05	0.0004	0.000191	4
BP	GO:00702	4/88	47/18670	7.04E-05	0.0004	0.000191	4
BP	GO:00059	8/88	292/18670	7.08E-05	0.000401	0.000191	8
BP	GO:00515	6/88	148/18670	7.12E-05	0.000403	0.000192	6
BP	GO:00720	8/88	293/18670	7.25E-05	0.00041	0.000195	8
BP	GO:19048	5/88	92/18670	7.39E-05	0.000417	0.000199	5
BP	GO:00162	8/88	295/18670	7.60E-05	0.000428	0.000204	8
BP	GO:00519	4/88	48/18670	7.65E-05	0.00043	0.000205	4
BP	GO:00083	6/88	150/18670	7.67E-05	0.00043	0.000205	6
BP	GO:00421	9/88	381/18670	7.71E-05	0.000432	0.000206	9
BP	GO:00108	3/88	18/18670	7.84E-05	0.000438	0.000209	3
BP	GO:00607	3/88	18/18670	7.84E-05	0.000438	0.000209	3
BP	GO:00613	3/88	18/18670	7.84E-05	0.000438	0.000209	3
BP	GO:00487	7/88	219/18670	7.88E-05	0.000439	0.000209	7
BP	GO:00026	4/88	49/18670	8.30E-05	0.00046	0.000219	4
BP	GO:00308	4/88	49/18670	8.30E-05	0.00046	0.000219	4
BP	GO:00327	4/88	49/18670	8.30E-05	0.00046	0.000219	4
BP	GO:00061	8/88	300/18670	8.55E-05	0.000473	0.000225	8
BP	GO:00903	6/88	153/18670	8.56E-05	0.000473	0.000225	6
BP	GO:00421	5/88	95/18670	8.61E-05	0.000473	0.000225	5
BP	GO:00421	5/88	95/18670	8.61E-05	0.000473	0.000225	5
BP	GO:00508	5/88	95/18670	8.61E-05	0.000473	0.000225	5
BP	GO:19030	5/88	95/18670	8.61E-05	0.000473	0.000225	5
BP	GO:00454	7/88	223/18670	8.82E-05	0.000484	0.000231	7
BP	GO:00029	4/88	50/18670	8.99E-05	0.000492	0.000235	4
BP	GO:00708	10/88	483/18670	9.08E-05	0.000496	0.000237	10
BP	GO:00105	3/88	19/18670	9.28E-05	0.000501	0.000239	3
BP	GO:00108	3/88	19/18670	9.28E-05	0.000501	0.000239	3
BP	GO:00331	3/88	19/18670	9.28E-05	0.000501	0.000239	3
BP	GO:00515	3/88	19/18670	9.28E-05	0.000501	0.000239	3
BP	GO:00601	3/88	19/18670	9.28E-05	0.000501	0.000239	3
BP	GO:00609	3/88	19/18670	9.28E-05	0.000501	0.000239	3
BP	GO:19021	3/88	19/18670	9.28E-05	0.000501	0.000239	3
BP	GO:19037	3/88	19/18670	9.28E-05	0.000501	0.000239	3
BP	GO:00432	5/88	97/18670	9.51E-05	0.000511	0.000244	5
BP	GO:00447	5/88	97/18670	9.51E-05	0.000511	0.000244	5
BP	GO:00308	6/88	156/18670	9.52E-05	0.000511	0.000244	6
BP	GO:00347	6/88	156/18670	9.52E-05	0.000511	0.000244	6
BP	GO:00193	4/88	51/18670	9.72E-05	0.000521	0.000248	4
BP	GO:00315	6/88	157/18670	9.86E-05	0.000528	0.000252	6
BP	GO:00507	5/88	98/18670	9.98E-05	0.000533	0.000254	5
BP	GO:00725	10/88	489/18670	0.000101	0.000536	0.000256	10
BP	GO:00510	7/88	228/18670	0.000101	0.00054	0.000257	7
BP	GO:00421	8/88	310/18670	0.000107	0.00057	0.000272	8
BP	GO:00425	3/88	20/18670	0.000109	0.000576	0.000275	3

BP	GO:00517	3/88	20/18670	0.000109	0.000576	0.000275	3
BP	GO:00517	3/88	20/18670	0.000109	0.000576	0.000275	3
BP	GO:19035	9/88	399/1867(	0.000109	0.000579	0.000276	9
BP	GO:00458	4/88	53/18670	0.000113	0.000597	0.000285	4
BP	GO:00725	8/88	313/1867(	0.000115	0.000604	0.000288	8
BP	GO:00310	8/88	315/1867(	0.00012	0.00063	0.0003	8
BP	GO:00105	5/88	102/1867(	0.000121	0.000633	0.000302	5
BP	GO:00312	6/88	163/1867(	0.000121	0.000635	0.000303	6
BP	GO:00511	6/88	163/1867(	0.000121	0.000635	0.000303	6
BP	GO:00105	4/88	54/18670	0.000122	0.000636	0.000303	4
BP	GO:00513	4/88	54/18670	0.000122	0.000636	0.000303	4
BP	GO:00439	9/88	405/1867(	0.000123	0.000639	0.000305	9
BP	GO:00703	8/88	317/1867(	0.000125	0.000651	0.000311	8
BP	GO:00307	3/88	21/18670	0.000127	0.000655	0.000312	3
BP	GO:00364	3/88	21/18670	0.000127	0.000655	0.000312	3
BP	GO:00468	3/88	21/18670	0.000127	0.000655	0.000312	3
BP	GO:00709	3/88	21/18670	0.000127	0.000655	0.000312	3
BP	GO:00506	6/88	165/1867(	0.00013	0.000669	0.000319	6
BP	GO:00716	6/88	165/1867(	0.00013	0.000669	0.000319	6
BP	GO:00015	5/88	104/1867(	0.000132	0.00068	0.000324	5
BP	GO:00305	5/88	104/1867(	0.000132	0.00068	0.000324	5
BP	GO:00161	6/88	166/1867(	0.000134	0.000688	0.000328	6
BP	GO:00075	9/88	410/1867(	0.000134	0.000689	0.000329	9
BP	GO:00313	7/88	239/1867(	0.000136	0.000695	0.000332	7
BP	GO:00312	4/88	56/18670	0.00014	0.000718	0.000342	4
BP	GO:00508	6/88	168/1867(	0.000143	0.00073	0.000348	6
BP	GO:00312	3/88	22/18670	0.000146	0.00074	0.000353	3
BP	GO:00358	3/88	22/18670	0.000146	0.00074	0.000353	3
BP	GO:00423	3/88	22/18670	0.000146	0.00074	0.000353	3
BP	GO:00434	3/88	22/18670	0.000146	0.00074	0.000353	3
BP	GO:19015	3/88	22/18670	0.000146	0.00074	0.000353	3
BP	GO:00020	4/88	57/18670	0.00015	0.000758	0.000362	4
BP	GO:00312	4/88	57/18670	0.00015	0.000758	0.000362	4
BP	GO:00459	4/88	57/18670	0.00015	0.000758	0.000362	4
BP	GO:19005	4/88	57/18670	0.00015	0.000758	0.000362	4
BP	GO:00303	5/88	107/1867(	0.000151	0.00076	0.000362	5
BP	GO:00620	5/88	107/1867(	0.000151	0.00076	0.000362	5
BP	GO:00511	8/88	326/1867(	0.000152	0.000761	0.000363	8
BP	GO:00711	5/88	108/1867(	0.000158	0.00079	0.000377	5
BP	GO:20002	5/88	108/1867(	0.000158	0.00079	0.000377	5
BP	GO:00619	4/88	58/18670	0.000161	0.000804	0.000384	4
BP	GO:00215	6/88	172/1867(	0.000163	0.000811	0.000387	6
BP	GO:00346	3/88	23/18670	0.000167	0.000834	0.000398	3
BP	GO:00016	6/88	173/1867(	0.000168	0.000834	0.000398	6
BP	GO:00104	6/88	173/1867(	0.000168	0.000834	0.000398	6
BP	GO:00433	4/88	59/18670	0.000172	0.000853	0.000407	4
BP	GO:00335	5/88	110/1867(	0.000172	0.000853	0.000407	5
BP	GO:00160	8/88	333/1867(	0.000175	0.000866	0.000413	8
BP	GO:00321	8/88	333/1867(	0.000175	0.000866	0.000413	8
BP	GO:00300	7/88	250/1867(	0.000179	0.000884	0.000422	7
BP	GO:00069	6/88	176/1867(	0.000184	0.000906	0.000432	6
BP	GO:00507	6/88	176/1867(	0.000184	0.000906	0.000432	6
BP	GO:00020	5/88	112/1867(	0.000187	0.000917	0.000437	5
BP	GO:00432	5/88	112/1867(	0.000187	0.000917	0.000437	5
BP	GO:19907	5/88	112/1867(	0.000187	0.000917	0.000437	5
BP	GO:00323	4/88	61/18670	0.000196	0.000956	0.000456	4
BP	GO:00713	4/88	61/18670	0.000196	0.000956	0.000456	4
BP	GO:19059	4/88	61/18670	0.000196	0.000956	0.000456	4

BP	GO:00068	9/88	434/1867(	0.000206	0.001002	0.000478	9
BP	GO:00076	7/88	256/1867(	0.000207	0.001008	0.000481	7
BP	GO:00700	4/88	62/18670	0.000209	0.001014	0.000483	4
BP	GO:19040	8/88	342/1867(	0.00021	0.00102	0.000486	8
BP	GO:19043	5/88	115/1867(	0.000212	0.001026	0.000489	5
BP	GO:00069	8/88	343/1867(	0.000214	0.001035	0.000493	8
BP	GO:00092	3/88	25/18670	0.000216	0.001035	0.000493	3
BP	GO:00194	3/88	25/18670	0.000216	0.001035	0.000493	3
BP	GO:00324	3/88	25/18670	0.000216	0.001035	0.000493	3
BP	GO:00509	3/88	25/18670	0.000216	0.001035	0.000493	3
BP	GO:00604	3/88	25/18670	0.000216	0.001035	0.000493	3
BP	GO:00605	3/88	25/18670	0.000216	0.001035	0.000493	3
BP	GO:20003	3/88	25/18670	0.000216	0.001035	0.000493	3
BP	GO:00107	8/88	344/1867(	0.000219	0.001046	0.000499	8
BP	GO:00217	5/88	116/1867(	0.000221	0.001053	0.000502	5
BP	GO:00219	5/88	116/1867(	0.000221	0.001053	0.000502	5
BP	GO:00511	8/88	346/1867(	0.000227	0.001084	0.000517	8
BP	GO:00027	5/88	117/1867(	0.00023	0.001093	0.000521	5
BP	GO:00486	6/88	184/1867(	0.000234	0.001114	0.000531	6
BP	GO:00456	4/88	64/18670	0.000236	0.001118	0.000533	4
BP	GO:20003	4/88	64/18670	0.000236	0.001118	0.000533	4
BP	GO:19902	5/88	118/1867(	0.000239	0.001131	0.000539	5
BP	GO:19037	6/88	185/1867(	0.000241	0.001141	0.000544	6
BP	GO:00509	3/88	26/18670	0.000243	0.001148	0.000548	3
BP	GO:00442	9/88	445/1867(	0.000248	0.001167	0.000557	9
BP	GO:00019	5/88	119/1867(	0.000248	0.00117	0.000558	5
BP	GO:00025	4/88	65/18670	0.000251	0.001176	0.000561	4
BP	GO:00069	4/88	65/18670	0.000251	0.001176	0.000561	4
BP	GO:00400	4/88	66/18670	0.000266	0.001243	0.000593	4
BP	GO:00517	4/88	66/18670	0.000266	0.001243	0.000593	4
BP	GO:00519	4/88	66/18670	0.000266	0.001243	0.000593	4
BP	GO:00723	7/88	267/1867(	0.000268	0.00125	0.000596	7
BP	GO:00436	5/88	121/1867(	0.000268	0.001252	0.000597	5
BP	GO:00096	3/88	27/18670	0.000273	0.00126	0.000601	3
BP	GO:00359	3/88	27/18670	0.000273	0.00126	0.000601	3
BP	GO:00459	3/88	27/18670	0.000273	0.00126	0.000601	3
BP	GO:00481	3/88	27/18670	0.000273	0.00126	0.000601	3
BP	GO:00714	3/88	27/18670	0.000273	0.00126	0.000601	3
BP	GO:00714	3/88	27/18670	0.000273	0.00126	0.000601	3
BP	GO:20001	3/88	27/18670	0.000273	0.00126	0.000601	3
BP	GO:00507	7/88	268/1867(	0.000274	0.001264	0.000603	7
BP	GO:00069	8/88	356/1867(	0.000276	0.00127	0.000606	8
BP	GO:00190	5/88	122/1867(	0.000279	0.001284	0.000612	5
BP	GO:00072	7/88	269/1867(	0.00028	0.001288	0.000614	7
BP	GO:00519	5/88	123/1867(	0.00029	0.001328	0.000633	5
BP	GO:00716	5/88	123/1867(	0.00029	0.001328	0.000633	5
BP	GO:00431	6/88	192/1867(	0.000295	0.001349	0.000643	6
BP	GO:00162	4/88	68/18670	0.000298	0.001356	0.000647	4
BP	GO:00336	4/88	68/18670	0.000298	0.001356	0.000647	4
BP	GO:00359	4/88	68/18670	0.000298	0.001356	0.000647	4
BP	GO:00509	4/88	68/18670	0.000298	0.001356	0.000647	4
BP	GO:00712	4/88	68/18670	0.000298	0.001356	0.000647	4
BP	GO:19021	7/88	272/1867(	0.0003	0.001361	0.000649	7
BP	GO:00192	5/88	124/1867(	0.000301	0.001364	0.000651	5
BP	GO:00109	8/88	361/1867(	0.000303	0.001371	0.000654	8
BP	GO:00015	3/88	28/18670	0.000304	0.001372	0.000654	3
BP	GO:00082	3/88	28/18670	0.000304	0.001372	0.000654	3
BP	GO:00335	3/88	28/18670	0.000304	0.001372	0.000654	3

BP	GO:00464	3/88	28/18670	0.000304	0.001372	0.000654	3
BP	GO:00702	4/88	69/18670	0.000315	0.00142	0.000677	4
BP	GO:00181	5/88	126/1867(	0.000324	0.001456	0.000694	5
BP	GO:00026	9/88	463/1867(	0.000331	0.001487	0.000709	9
BP	GO:00436	5/88	127/1867(	0.000336	0.001506	0.000718	5
BP	GO:00022	6/88	197/1867(	0.000338	0.001508	0.000719	6
BP	GO:00003	3/88	29/18670	0.000338	0.001508	0.000719	3
BP	GO:00059	3/88	29/18670	0.000338	0.001508	0.000719	3
BP	GO:00072	3/88	29/18670	0.000338	0.001508	0.000719	3
BP	GO:00109	3/88	29/18670	0.000338	0.001508	0.000719	3
BP	GO:00513	9/88	466/1867(	0.000347	0.001545	0.000737	9
BP	GO:00336	4/88	71/18670	0.000352	0.001562	0.000745	4
BP	GO:00714	4/88	71/18670	0.000352	0.001562	0.000745	4
BP	GO:00071	6/88	199/1867(	0.000357	0.001578	0.000752	6
BP	GO:00359	6/88	199/1867(	0.000357	0.001578	0.000752	6
BP	GO:00508	6/88	199/1867(	0.000357	0.001578	0.000752	6
BP	GO:00714	5/88	129/1867(	0.000361	0.001594	0.00076	5
BP	GO:00025	4/88	72/18670	0.000371	0.001638	0.000781	4
BP	GO:00003	3/88	30/18670	0.000375	0.001641	0.000782	3
BP	GO:00463	3/88	30/18670	0.000375	0.001641	0.000782	3
BP	GO:00481	3/88	30/18670	0.000375	0.001641	0.000782	3
BP	GO:00488	3/88	30/18670	0.000375	0.001641	0.000782	3
BP	GO:00715	3/88	30/18670	0.000375	0.001641	0.000782	3
BP	GO:00902	3/88	30/18670	0.000375	0.001641	0.000782	3
BP	GO:00301	5/88	131/1867(	0.000387	0.001689	0.000806	5
BP	GO:00468	5/88	131/1867(	0.000387	0.001689	0.000806	5
BP	GO:00987	5/88	131/1867(	0.000387	0.001689	0.000806	5
BP	GO:00311	4/88	73/18670	0.000391	0.001705	0.000813	4
BP	GO:00302	6/88	203/1867(	0.000397	0.001725	0.000822	6
BP	GO:00159	7/88	285/1867(	0.000397	0.001725	0.000822	7
BP	GO:00305	5/88	132/1867(	0.000401	0.001737	0.000828	5
BP	GO:00432	5/88	132/1867(	0.000401	0.001737	0.000828	5
BP	GO:00455	5/88	132/1867(	0.000401	0.001737	0.000828	5
BP	GO:00514	7/88	286/1867(	0.000405	0.001752	0.000836	7
BP	GO:00316	4/88	74/18670	0.000412	0.001775	0.000846	4
BP	GO:00326	4/88	74/18670	0.000412	0.001775	0.000846	4
BP	GO:00105	3/88	31/18670	0.000413	0.001775	0.000846	3
BP	GO:00331	3/88	31/18670	0.000413	0.001775	0.000846	3
BP	GO:00434	3/88	31/18670	0.000413	0.001775	0.000846	3
BP	GO:00457	3/88	31/18670	0.000413	0.001775	0.000846	3
BP	GO:00027	7/88	288/1867(	0.000422	0.001811	0.000864	7
BP	GO:00067	5/88	134/1867(	0.00043	0.001829	0.000872	5
BP	GO:00182	5/88	134/1867(	0.00043	0.001829	0.000872	5
BP	GO:00302	5/88	134/1867(	0.00043	0.001829	0.000872	5
BP	GO:00444	5/88	134/1867(	0.00043	0.001829	0.000872	5
BP	GO:00518	5/88	134/1867(	0.00043	0.001829	0.000872	5
BP	GO:00518	5/88	134/1867(	0.00043	0.001829	0.000872	5
BP	GO:19032	4/88	75/18670	0.000434	0.001845	0.00088	4
BP	GO:00300	6/88	207/1867(	0.00044	0.001868	0.000891	6
BP	GO:00347	9/88	483/1867(	0.00045	0.00191	0.000911	9
BP	GO:00082	3/88	32/18670	0.000455	0.001916	0.000914	3
BP	GO:00381	3/88	32/18670	0.000455	0.001916	0.000914	3
BP	GO:00423	3/88	32/18670	0.000455	0.001916	0.000914	3
BP	GO:00466	3/88	32/18670	0.000455	0.001916	0.000914	3
BP	GO:00519	3/88	32/18670	0.000455	0.001916	0.000914	3
BP	GO:00226	9/88	484/1867(	0.000457	0.001925	0.000918	9
BP	GO:00072	5/88	136/1867(	0.00046	0.001934	0.000922	5
BP	GO:00511	7/88	293/1867(	0.000468	0.001967	0.000938	7

BP	GO:19012	4/88	77/18670	0.00048	0.002012	0.00096	4
BP	GO:00508	7/88	296/1867(	0.000498	0.002076	0.00099	7
BP	GO:00162	3/88	33/18670	0.000498	0.002076	0.00099	3
BP	GO:00360	3/88	33/18670	0.000498	0.002076	0.00099	3
BP	GO:00606	3/88	33/18670	0.000498	0.002076	0.00099	3
BP	GO:19011	3/88	33/18670	0.000498	0.002076	0.00099	3
BP	GO:20003	3/88	33/18670	0.000498	0.002076	0.00099	3
BP	GO:00002	4/88	78/18670	0.000504	0.002094	0.000998	4
BP	GO:00310	4/88	78/18670	0.000504	0.002094	0.000998	4
BP	GO:00072	6/88	214/1867(	0.000524	0.002171	0.001036	6
BP	GO:00160	6/88	214/1867(	0.000524	0.002171	0.001036	6
BP	GO:00457	6/88	214/1867(	0.000524	0.002171	0.001036	6
BP	GO:00015	4/88	79/18670	0.000529	0.002184	0.001042	4
BP	GO:00061	4/88	79/18670	0.000529	0.002184	0.001042	4
BP	GO:00323	6/88	215/1867(	0.000537	0.002218	0.001058	6
BP	GO:00703	7/88	300/1867(	0.000539	0.002221	0.001059	7
BP	GO:00975	5/88	141/1867(	0.000542	0.002232	0.001064	5
BP	GO:00425	3/88	34/18670	0.000545	0.002237	0.001067	3
BP	GO:00425	3/88	34/18670	0.000545	0.002237	0.001067	3
BP	GO:00328	6/88	216/1867(	0.000551	0.002259	0.001077	6
BP	GO:00226	4/88	80/18670	0.000555	0.002272	0.001084	4
BP	GO:00197	6/88	218/1867(	0.000578	0.002366	0.001128	6
BP	GO:00308	4/88	81/18670	0.000581	0.002374	0.001132	4
BP	GO:19029	4/88	81/18670	0.000581	0.002374	0.001132	4
BP	GO:00302	3/88	35/18670	0.000594	0.00241	0.001149	3
BP	GO:00327	3/88	35/18670	0.000594	0.00241	0.001149	3
BP	GO:00708	3/88	35/18670	0.000594	0.00241	0.001149	3
BP	GO:00713	3/88	35/18670	0.000594	0.00241	0.001149	3
BP	GO:19040	3/88	35/18670	0.000594	0.00241	0.001149	3
BP	GO:19021	5/88	144/1867(	0.000597	0.002419	0.001154	5
BP	GO:19059	4/88	82/18670	0.000609	0.002466	0.001176	4
BP	GO:00000	5/88	145/1867(	0.000616	0.002491	0.001188	5
BP	GO:00439	6/88	222/1867(	0.000636	0.00257	0.001225	6
BP	GO:00712	4/88	83/18670	0.000638	0.002573	0.001227	4
BP	GO:00425	3/88	36/18670	0.000646	0.002599	0.00124	3
BP	GO:00457	3/88	36/18670	0.000646	0.002599	0.00124	3
BP	GO:00193	5/88	147/1867(	0.000655	0.002632	0.001255	5
BP	GO:00193	5/88	147/1867(	0.000655	0.002632	0.001255	5
BP	GO:00105	4/88	84/18670	0.000667	0.002664	0.001271	4
BP	GO:00431	4/88	84/18670	0.000667	0.002664	0.001271	4
BP	GO:00454	4/88	84/18670	0.000667	0.002664	0.001271	4
BP	GO:00468	4/88	84/18670	0.000667	0.002664	0.001271	4
BP	GO:19058	4/88	84/18670	0.000667	0.002664	0.001271	4
BP	GO:00605	8/88	408/1867(	0.00068	0.002712	0.001293	8
BP	GO:00016	6/88	225/1867(	0.000682	0.002716	0.001295	6
BP	GO:00331	6/88	225/1867(	0.000682	0.002716	0.001295	6
BP	GO:00510	5/88	149/1867(	0.000697	0.002743	0.001308	5
BP	GO:20010	5/88	149/1867(	0.000697	0.002743	0.001308	5
BP	GO:00140	4/88	85/18670	0.000698	0.002743	0.001308	4
BP	GO:00343	4/88	85/18670	0.000698	0.002743	0.001308	4
BP	GO:00069	3/88	37/18670	0.0007	0.002743	0.001308	3
BP	GO:00140	3/88	37/18670	0.0007	0.002743	0.001308	3
BP	GO:00149	3/88	37/18670	0.0007	0.002743	0.001308	3
BP	GO:00302	3/88	37/18670	0.0007	0.002743	0.001308	3
BP	GO:00332	3/88	37/18670	0.0007	0.002743	0.001308	3
BP	GO:00380	3/88	37/18670	0.0007	0.002743	0.001308	3
BP	GO:00423	3/88	37/18670	0.0007	0.002743	0.001308	3
BP	GO:00460	3/88	37/18670	0.0007	0.002743	0.001308	3

BP	GO:00900	3/88	37/18670	0.0007	0.002743	0.001308	3
BP	GO:20001	3/88	37/18670	0.0007	0.002743	0.001308	3
BP	GO:00725	5/88	150/1867(	0.000718	0.00281	0.00134	5
BP	GO:00327	4/88	86/18670	0.000729	0.002847	0.001357	4
BP	GO:00420	4/88	86/18670	0.000729	0.002847	0.001357	4
BP	GO:00169	5/88	151/1867(	0.00074	0.002886	0.001376	5
BP	GO:00320	3/88	38/18670	0.000758	0.002939	0.001402	3
BP	GO:00468	3/88	38/18670	0.000758	0.002939	0.001402	3
BP	GO:00480	3/88	38/18670	0.000758	0.002939	0.001402	3
BP	GO:19040	3/88	38/18670	0.000758	0.002939	0.001402	3
BP	GO:19047	3/88	38/18670	0.000758	0.002939	0.001402	3
BP	GO:00097	6/88	230/1867(	0.000765	0.002965	0.001414	6
BP	GO:00507	5/88	153/1867(	0.000785	0.003038	0.001449	5
BP	GO:00341	4/88	88/18670	0.000795	0.003059	0.001459	4
BP	GO:00434	4/88	88/18670	0.000795	0.003059	0.001459	4
BP	GO:00700	4/88	88/18670	0.000795	0.003059	0.001459	4
BP	GO:19004	4/88	88/18670	0.000795	0.003059	0.001459	4
BP	GO:19035	4/88	88/18670	0.000795	0.003059	0.001459	4
BP	GO:00713	7/88	321/1867(	0.000803	0.003088	0.001472	7
BP	GO:00605	7/88	322/1867(	0.000818	0.003131	0.001493	7
BP	GO:00106	3/88	39/18670	0.000818	0.003131	0.001493	3
BP	GO:00609	3/88	39/18670	0.000818	0.003131	0.001493	3
BP	GO:19045	3/88	39/18670	0.000818	0.003131	0.001493	3
BP	GO:00973	4/88	89/18670	0.000829	0.003171	0.001512	4
BP	GO:00609	5/88	155/1867(	0.000832	0.003179	0.001516	5
BP	GO:00467	6/88	235/1867(	0.000856	0.003265	0.001557	6
BP	GO:00323	8/88	423/1867(	0.000859	0.003275	0.001562	8
BP	GO:00424	4/88	90/18670	0.000865	0.003289	0.001568	4
BP	GO:00716	4/88	90/18670	0.000865	0.003289	0.001568	4
BP	GO:00512	3/88	40/18670	0.000881	0.003345	0.001595	3
BP	GO:00715	3/88	40/18670	0.000881	0.003345	0.001595	3
BP	GO:00328	6/88	237/1867(	0.000894	0.003386	0.001615	6
BP	GO:00431	6/88	237/1867(	0.000894	0.003386	0.001615	6
BP	GO:00518	4/88	91/18670	0.000901	0.003405	0.001624	4
BP	GO:19019	4/88	91/18670	0.000901	0.003405	0.001624	4
BP	GO:00604	5/88	158/1867(	0.000907	0.003424	0.001633	5
BP	GO:00164	7/88	328/1867(	0.000911	0.003434	0.001638	7
BP	GO:00703	6/88	239/1867(	0.000934	0.003517	0.001677	6
BP	GO:00458	4/88	92/18670	0.000939	0.003528	0.001683	4
BP	GO:20001	4/88	92/18670	0.000939	0.003528	0.001683	4
BP	GO:00507	3/88	41/18670	0.000948	0.003543	0.001689	3
BP	GO:00508	3/88	41/18670	0.000948	0.003543	0.001689	3
BP	GO:01500	3/88	41/18670	0.000948	0.003543	0.001689	3
BP	GO:19001	3/88	41/18670	0.000948	0.003543	0.001689	3
BP	GO:19030	3/88	41/18670	0.000948	0.003543	0.001689	3
BP	GO:00302	6/88	240/1867(	0.000954	0.003559	0.001697	6
BP	GO:00506	6/88	240/1867(	0.000954	0.003559	0.001697	6
BP	GO:00161	2/88	10/18670	0.000964	0.003564	0.001699	2
BP	GO:00306	2/88	10/18670	0.000964	0.003564	0.001699	2
BP	GO:00312	2/88	10/18670	0.000964	0.003564	0.001699	2
BP	GO:00334	2/88	10/18670	0.000964	0.003564	0.001699	2
BP	GO:00600	2/88	10/18670	0.000964	0.003564	0.001699	2
BP	GO:00703	2/88	10/18670	0.000964	0.003564	0.001699	2
BP	GO:00712	2/88	10/18670	0.000964	0.003564	0.001699	2
BP	GO:19037	2/88	10/18670	0.000964	0.003564	0.001699	2
BP	GO:19038	2/88	10/18670	0.000964	0.003564	0.001699	2
BP	GO:00030	4/88	93/18670	0.000977	0.003604	0.001719	4
BP	GO:19011	4/88	93/18670	0.000977	0.003604	0.001719	4

BP	GO:00158	7/88	333/1867(	0.000995	0.00366	0.001745	7
BP	GO:00469	7/88	333/1867(	0.000995	0.00366	0.001745	7
BP	GO:00109	3/88	42/18670	0.001017	0.003735	0.001781	3
BP	GO:00421	3/88	42/18670	0.001017	0.003735	0.001781	3
BP	GO:00508	8/88	436/1867(	0.001044	0.003828	0.001826	8
BP	GO:00450	4/88	95/18670	0.001058	0.003873	0.001847	4
BP	GO:00511	4/88	95/18670	0.001058	0.003873	0.001847	4
BP	GO:00991	8/88	437/1867(	0.001059	0.003873	0.001847	8
BP	GO:00066	5/88	164/1867(	0.001072	0.003914	0.001866	5
BP	GO:00066	3/88	43/18670	0.00109	0.00396	0.001888	3
BP	GO:00066	3/88	43/18670	0.00109	0.00396	0.001888	3
BP	GO:00312	3/88	43/18670	0.00109	0.00396	0.001888	3
BP	GO:00324	3/88	43/18670	0.00109	0.00396	0.001888	3
BP	GO:00341	3/88	43/18670	0.00109	0.00396	0.001888	3
BP	GO:00106	4/88	96/18670	0.0011	0.003986	0.001901	4
BP	GO:00973	4/88	96/18670	0.0011	0.003986	0.001901	4
BP	GO:20011	4/88	96/18670	0.0011	0.003986	0.001901	4
BP	GO:00515	4/88	97/18670	0.001144	0.004125	0.001967	4
BP	GO:19028	4/88	97/18670	0.001144	0.004125	0.001967	4
BP	GO:19908	4/88	97/18670	0.001144	0.004125	0.001967	4
BP	GO:19908	4/88	97/18670	0.001144	0.004125	0.001967	4
BP	GO:00715	6/88	249/1867(	0.001153	0.004156	0.001982	6
BP	GO:00330	7/88	342/1867(	0.001161	0.004167	0.001987	7
BP	GO:00140	3/88	44/18670	0.001165	0.004167	0.001987	3
BP	GO:00302	3/88	44/18670	0.001165	0.004167	0.001987	3
BP	GO:00329	3/88	44/18670	0.001165	0.004167	0.001987	3
BP	GO:00341	3/88	44/18670	0.001165	0.004167	0.001987	3
BP	GO:00903	3/88	44/18670	0.001165	0.004167	0.001987	3
BP	GO:00193	2/88	11/18670	0.001175	0.004167	0.001987	2
BP	GO:00316	2/88	11/18670	0.001175	0.004167	0.001987	2
BP	GO:00336	2/88	11/18670	0.001175	0.004167	0.001987	2
BP	GO:00436	2/88	11/18670	0.001175	0.004167	0.001987	2
BP	GO:00458	2/88	11/18670	0.001175	0.004167	0.001987	2
BP	GO:00519	2/88	11/18670	0.001175	0.004167	0.001987	2
BP	GO:00725	2/88	11/18670	0.001175	0.004167	0.001987	2
BP	GO:00726	2/88	11/18670	0.001175	0.004167	0.001987	2
BP	GO:19904	2/88	11/18670	0.001175	0.004167	0.001987	2
BP	GO:20012	2/88	11/18670	0.001175	0.004167	0.001987	2
BP	GO:00109	6/88	250/1867(	0.001177	0.00417	0.001988	6
BP	GO:00020	4/88	98/18670	0.001188	0.004203	0.002004	4
BP	GO:00215	6/88	251/1867(	0.001202	0.004243	0.002023	6
BP	GO:00456	6/88	251/1867(	0.001202	0.004243	0.002023	6
BP	GO:00303	5/88	169/1867(	0.001225	0.00432	0.00206	5
BP	GO:00193	4/88	99/18670	0.001234	0.004343	0.002071	4
BP	GO:19015	4/88	99/18670	0.001234	0.004343	0.002071	4
BP	GO:00310	3/88	45/18670	0.001244	0.004359	0.002079	3
BP	GO:00325	3/88	45/18670	0.001244	0.004359	0.002079	3
BP	GO:00359	3/88	45/18670	0.001244	0.004359	0.002079	3
BP	GO:00427	3/88	45/18670	0.001244	0.004359	0.002079	3
BP	GO:00485	3/88	45/18670	0.001244	0.004359	0.002079	3
BP	GO:00106	4/88	100/1867(	0.00128	0.004481	0.002137	4
BP	GO:00604	5/88	171/1867(	0.00129	0.004511	0.002151	5
BP	GO:00715	6/88	255/1867(	0.001303	0.00455	0.00217	6
BP	GO:00071	7/88	349/1867(	0.001304	0.00455	0.00217	7
BP	GO:00450	8/88	452/1867(	0.001313	0.004578	0.002183	8
BP	GO:00059	3/88	46/18670	0.001327	0.004589	0.002188	3
BP	GO:00092	3/88	46/18670	0.001327	0.004589	0.002188	3
BP	GO:00108	3/88	46/18670	0.001327	0.004589	0.002188	3

BP	GO:00336	3/88	46/18670	0.001327	0.004589	0.002188	3
BP	GO:00351	3/88	46/18670	0.001327	0.004589	0.002188	3
BP	GO:00607	3/88	46/18670	0.001327	0.004589	0.002188	3
BP	GO:19909	3/88	46/18670	0.001327	0.004589	0.002188	3
BP	GO:00326	4/88	101/1867(	0.001328	0.004589	0.002188	4
BP	GO:00344	4/88	101/1867(	0.001328	0.004589	0.002188	4
BP	GO:00488	6/88	257/1867(	0.001356	0.004679	0.002231	6
BP	GO:00000	4/88	102/1867(	0.001378	0.004745	0.002263	4
BP	GO:00988	4/88	102/1867(	0.001378	0.004745	0.002263	4
BP	GO:00065	2/88	12/18670	0.001406	0.004791	0.002285	2
BP	GO:00108	2/88	12/18670	0.001406	0.004791	0.002285	2
BP	GO:00312	2/88	12/18670	0.001406	0.004791	0.002285	2
BP	GO:00313	2/88	12/18670	0.001406	0.004791	0.002285	2
BP	GO:00423	2/88	12/18670	0.001406	0.004791	0.002285	2
BP	GO:00515	2/88	12/18670	0.001406	0.004791	0.002285	2
BP	GO:00702	2/88	12/18670	0.001406	0.004791	0.002285	2
BP	GO:00704	2/88	12/18670	0.001406	0.004791	0.002285	2
BP	GO:00972	2/88	12/18670	0.001406	0.004791	0.002285	2
BP	GO:19004	2/88	12/18670	0.001406	0.004791	0.002285	2
BP	GO:19038	2/88	12/18670	0.001406	0.004791	0.002285	2
BP	GO:00902	6/88	259/1867(	0.00141	0.004799	0.002288	6
BP	GO:00305	3/88	47/18670	0.001412	0.004799	0.002288	3
BP	GO:00708	3/88	47/18670	0.001412	0.004799	0.002288	3
BP	GO:00442	4/88	103/1867(	0.001428	0.004847	0.002311	4
BP	GO:00461	5/88	175/1867(	0.001429	0.004847	0.002311	5
BP	GO:00091	6/88	261/1867(	0.001467	0.004969	0.00237	6
BP	GO:00104	6/88	262/1867(	0.001495	0.005056	0.002411	6
BP	GO:00158	6/88	262/1867(	0.001495	0.005056	0.002411	6
BP	GO:00308	3/88	48/18670	0.001501	0.005067	0.002416	3
BP	GO:00459	3/88	48/18670	0.001501	0.005067	0.002416	3
BP	GO:00019	5/88	177/1867(	0.001503	0.005067	0.002416	5
BP	GO:00070	4/88	105/1867(	0.001533	0.005164	0.002463	4
BP	GO:00109	5/88	178/1867(	0.001541	0.005179	0.00247	5
BP	GO:20012	5/88	178/1867(	0.001541	0.005179	0.00247	5
BP	GO:00085	8/88	464/1867(	0.00155	0.005205	0.002482	8
BP	GO:00313	6/88	264/1867(	0.001554	0.005215	0.002487	6
BP	GO:00030	8/88	465/1867(	0.001571	0.005266	0.002511	8
BP	GO:19019	4/88	106/1867(	0.001588	0.005312	0.002533	4
BP	GO:19040	4/88	106/1867(	0.001588	0.005312	0.002533	4
BP	GO:00107	3/88	49/18670	0.001594	0.005313	0.002533	3
BP	GO:00485	3/88	49/18670	0.001594	0.005313	0.002533	3
BP	GO:19047	3/88	49/18670	0.001594	0.005313	0.002533	3
BP	GO:19900	3/88	49/18670	0.001594	0.005313	0.002533	3
BP	GO:00016	7/88	362/1867(	0.001608	0.005353	0.002553	7
BP	GO:00107	2/88	13/18670	0.001656	0.005464	0.002605	2
BP	GO:00108	2/88	13/18670	0.001656	0.005464	0.002605	2
BP	GO:00316	2/88	13/18670	0.001656	0.005464	0.002605	2
BP	GO:00319	2/88	13/18670	0.001656	0.005464	0.002605	2
BP	GO:00421	2/88	13/18670	0.001656	0.005464	0.002605	2
BP	GO:00435	2/88	13/18670	0.001656	0.005464	0.002605	2
BP	GO:00450	2/88	13/18670	0.001656	0.005464	0.002605	2
BP	GO:00605	2/88	13/18670	0.001656	0.005464	0.002605	2
BP	GO:00610	2/88	13/18670	0.001656	0.005464	0.002605	2
BP	GO:19028	2/88	13/18670	0.001656	0.005464	0.002605	2
BP	GO:00228	6/88	268/1867(	0.001677	0.005527	0.002636	6
BP	GO:00017	3/88	50/18670	0.00169	0.005559	0.002651	3
BP	GO:19000	3/88	50/18670	0.00169	0.005559	0.002651	3
BP	GO:00062	4/88	108/1867(	0.001701	0.005583	0.002663	4

BP	GO:19046	4/88	108/1867(	0.001701	0.005583	0.002663	4
BP	GO:01500	7/88	366/1867(	0.001711	0.005613	0.002677	7
BP	GO:00380	5/88	183/1867(	0.00174	0.005701	0.002719	5
BP	GO:00451	6/88	270/1867(	0.001742	0.005701	0.002719	6
BP	GO:00016	4/88	109/1867(	0.001759	0.005754	0.002744	4
BP	GO:00507	8/88	474/1867(	0.001772	0.00579	0.002761	8
BP	GO:00310	3/88	51/18670	0.001789	0.005826	0.002778	3
BP	GO:00311	3/88	51/18670	0.001789	0.005826	0.002778	3
BP	GO:00709	3/88	51/18670	0.001789	0.005826	0.002778	3
BP	GO:00973	3/88	51/18670	0.001789	0.005826	0.002778	3
BP	GO:19037	8/88	475/1867(	0.001795	0.00584	0.002785	8
BP	GO:00069	4/88	110/1867(	0.001819	0.005911	0.002819	4
BP	GO:00463	5/88	185/1867(	0.001825	0.005924	0.002825	5
BP	GO:00488	7/88	371/1867(	0.001848	0.005994	0.002858	7
BP	GO:00726	4/88	111/1867(	0.00188	0.006093	0.002906	4
BP	GO:00327	3/88	52/18670	0.001893	0.006122	0.002919	3
BP	GO:19900	3/88	52/18670	0.001893	0.006122	0.002919	3
BP	GO:00160	6/88	275/1867(	0.00191	0.006163	0.002939	6
BP	GO:00463	6/88	275/1867(	0.00191	0.006163	0.002939	6
BP	GO:00302	2/88	14/18670	0.001927	0.006163	0.002939	2
BP	GO:00323	2/88	14/18670	0.001927	0.006163	0.002939	2
BP	GO:00358	2/88	14/18670	0.001927	0.006163	0.002939	2
BP	GO:00362	2/88	14/18670	0.001927	0.006163	0.002939	2
BP	GO:00425	2/88	14/18670	0.001927	0.006163	0.002939	2
BP	GO:00433	2/88	14/18670	0.001927	0.006163	0.002939	2
BP	GO:00456	2/88	14/18670	0.001927	0.006163	0.002939	2
BP	GO:00515	2/88	14/18670	0.001927	0.006163	0.002939	2
BP	GO:00517	2/88	14/18670	0.001927	0.006163	0.002939	2
BP	GO:20012	2/88	14/18670	0.001927	0.006163	0.002939	2
BP	GO:00086	4/88	112/1867(	0.001943	0.006199	0.002956	4
BP	GO:00324	4/88	112/1867(	0.001943	0.006199	0.002956	4
BP	GO:00518	4/88	112/1867(	0.001943	0.006199	0.002956	4
BP	GO:00316	3/88	53/18670	0.001999	0.006351	0.003029	3
BP	GO:00329	3/88	53/18670	0.001999	0.006351	0.003029	3
BP	GO:00456	3/88	53/18670	0.001999	0.006351	0.003029	3
BP	GO:00702	3/88	53/18670	0.001999	0.006351	0.003029	3
BP	GO:00193	5/88	189/1867(	0.002004	0.006351	0.003029	5
BP	GO:00464	5/88	189/1867(	0.002004	0.006351	0.003029	5
BP	GO:00466	4/88	113/1867(	0.002007	0.006351	0.003029	4
BP	GO:00486	4/88	113/1867(	0.002007	0.006351	0.003029	4
BP	GO:00609	4/88	113/1867(	0.002007	0.006351	0.003029	4
BP	GO:00607	5/88	190/1867(	0.00205	0.006482	0.003091	5
BP	GO:00059	4/88	114/1867(	0.002073	0.006541	0.003119	4
BP	GO:00157	4/88	114/1867(	0.002073	0.006541	0.003119	4
BP	GO:00091	6/88	280/1867(	0.002091	0.006593	0.003144	6
BP	GO:00066	3/88	54/18670	0.00211	0.006623	0.003158	3
BP	GO:00301	3/88	54/18670	0.00211	0.006623	0.003158	3
BP	GO:00326	3/88	54/18670	0.00211	0.006623	0.003158	3
BP	GO:00433	3/88	54/18670	0.00211	0.006623	0.003158	3
BP	GO:00509	3/88	54/18670	0.00211	0.006623	0.003158	3
BP	GO:00060	4/88	115/1867(	0.00214	0.006705	0.003197	4
BP	GO:00326	4/88	115/1867(	0.00214	0.006705	0.003197	4
BP	GO:00309	7/88	381/1867(	0.002147	0.006721	0.003205	7
BP	GO:00067	4/88	116/1867(	0.002208	0.006858	0.00327	4
BP	GO:00342	4/88	116/1867(	0.002208	0.006858	0.00327	4
BP	GO:00308	2/88	15/18670	0.002216	0.006858	0.00327	2
BP	GO:00356	2/88	15/18670	0.002216	0.006858	0.00327	2
BP	GO:00423	2/88	15/18670	0.002216	0.006858	0.00327	2

BP	GO:00458	2/88	15/18670	0.002216	0.006858	0.00327	2
BP	GO:00463	2/88	15/18670	0.002216	0.006858	0.00327	2
BP	GO:00480	2/88	15/18670	0.002216	0.006858	0.00327	2
BP	GO:00510	2/88	15/18670	0.002216	0.006858	0.00327	2
BP	GO:00511	2/88	15/18670	0.002216	0.006858	0.00327	2
BP	GO:19001	2/88	15/18670	0.002216	0.006858	0.00327	2
BP	GO:20004	2/88	15/18670	0.002216	0.006858	0.00327	2
BP	GO:20010	2/88	15/18670	0.002216	0.006858	0.00327	2
BP	GO:00108	3/88	55/18670	0.002224	0.006864	0.003273	3
BP	GO:00423	3/88	55/18670	0.002224	0.006864	0.003273	3
BP	GO:00464	3/88	55/18670	0.002224	0.006864	0.003273	3
BP	GO:00316	6/88	284/1867(	0.002245	0.006922	0.003301	6
BP	GO:00426	7/88	385/1867(	0.002276	0.007001	0.003339	7
BP	GO:00601	4/88	117/1867(	0.002278	0.007001	0.003339	4
BP	GO:00609	4/88	117/1867(	0.002278	0.007001	0.003339	4
BP	GO:19012	4/88	117/1867(	0.002278	0.007001	0.003339	4
BP	GO:00725	5/88	195/1867(	0.002295	0.007047	0.003361	5
BP	GO:00326	3/88	56/18670	0.002342	0.007171	0.00342	3
BP	GO:19030	3/88	56/18670	0.002342	0.007171	0.00342	3
BP	GO:19032	3/88	56/18670	0.002342	0.007171	0.00342	3
BP	GO:00718	5/88	196/1867(	0.002347	0.00718	0.003424	5
BP	GO:00071	4/88	119/1867(	0.002423	0.007394	0.003526	4
BP	GO:00428	4/88	119/1867(	0.002423	0.007394	0.003526	4
BP	GO:00511	4/88	119/1867(	0.002423	0.007394	0.003526	4
BP	GO:00147	7/88	390/1867(	0.002447	0.00746	0.003557	7
BP	GO:00427	3/88	57/18670	0.002464	0.007498	0.003576	3
BP	GO:19004	3/88	57/18670	0.002464	0.007498	0.003576	3
BP	GO:00161	2/88	16/18670	0.002525	0.007645	0.003646	2
BP	GO:00193	2/88	16/18670	0.002525	0.007645	0.003646	2
BP	GO:00424	2/88	16/18670	0.002525	0.007645	0.003646	2
BP	GO:00432	2/88	16/18670	0.002525	0.007645	0.003646	2
BP	GO:19020	2/88	16/18670	0.002525	0.007645	0.003646	2
BP	GO:19020	2/88	16/18670	0.002525	0.007645	0.003646	2
BP	GO:00108	4/88	121/1867(	0.002574	0.007773	0.003707	4
BP	GO:00309	4/88	121/1867(	0.002574	0.007773	0.003707	4
BP	GO:00467	4/88	121/1867(	0.002574	0.007773	0.003707	4
BP	GO:00902	6/88	292/1867(	0.002578	0.007778	0.003709	6
BP	GO:00305	3/88	58/18670	0.002589	0.007786	0.003713	3
BP	GO:00713	3/88	58/18670	0.002589	0.007786	0.003713	3
BP	GO:00989	3/88	58/18670	0.002589	0.007786	0.003713	3
BP	GO:19045	3/88	58/18670	0.002589	0.007786	0.003713	3
BP	GO:00092	6/88	293/1867(	0.002622	0.007877	0.003756	6
BP	GO:00197	4/88	122/1867(	0.002652	0.007939	0.003786	4
BP	GO:00341	4/88	122/1867(	0.002652	0.007939	0.003786	4
BP	GO:00435	4/88	122/1867(	0.002652	0.007939	0.003786	4
BP	GO:19035	4/88	122/1867(	0.002652	0.007939	0.003786	4
BP	GO:00097	5/88	202/1867(	0.002674	0.007998	0.003814	5
BP	GO:00516	7/88	397/1867(	0.002702	0.008075	0.003851	7
BP	GO:00507	6/88	295/1867(	0.002712	0.008098	0.003862	6
BP	GO:00311	3/88	59/18670	0.002719	0.008099	0.003862	3
BP	GO:00327	3/88	59/18670	0.002719	0.008099	0.003862	3
BP	GO:19028	3/88	59/18670	0.002719	0.008099	0.003862	3
BP	GO:00313	5/88	204/1867(	0.002789	0.008302	0.003959	5
BP	GO:19035	6/88	297/1867(	0.002804	0.008338	0.003976	6
BP	GO:00140	4/88	124/1867(	0.002812	0.008355	0.003985	4
BP	GO:00027	6/88	298/1867(	0.002851	0.00837	0.003991	6
BP	GO:19028	3/88	60/18670	0.002852	0.00837	0.003991	3
BP	GO:00060	2/88	17/18670	0.002853	0.00837	0.003991	2

BP	GO:00069	2/88	17/18670	0.002853	0.00837	0.003991	2
BP	GO:00102	2/88	17/18670	0.002853	0.00837	0.003991	2
BP	GO:00157	2/88	17/18670	0.002853	0.00837	0.003991	2
BP	GO:00329	2/88	17/18670	0.002853	0.00837	0.003991	2
BP	GO:00450	2/88	17/18670	0.002853	0.00837	0.003991	2
BP	GO:00457	2/88	17/18670	0.002853	0.00837	0.003991	2
BP	GO:00506	2/88	17/18670	0.002853	0.00837	0.003991	2
BP	GO:00517	2/88	17/18670	0.002853	0.00837	0.003991	2
BP	GO:00606	2/88	17/18670	0.002853	0.00837	0.003991	2
BP	GO:00612	2/88	17/18670	0.002853	0.00837	0.003991	2
BP	GO:00718	2/88	17/18670	0.002853	0.00837	0.003991	2
BP	GO:20008	2/88	17/18670	0.002853	0.00837	0.003991	2
BP	GO:00068	4/88	125/1867(	0.002895	0.008486	0.004047	4
BP	GO:00463	6/88	300/1867(	0.002947	0.008631	0.004116	6
BP	GO:00067	5/88	207/1867(	0.00297	0.008684	0.004141	5
BP	GO:00342	5/88	207/1867(	0.00297	0.008684	0.004141	5
BP	GO:00460	4/88	126/1867(	0.002979	0.008704	0.004151	4
BP	GO:00068	3/88	61/18670	0.00299	0.008713	0.004155	3
BP	GO:00308	3/88	61/18670	0.00299	0.008713	0.004155	3
BP	GO:00451	3/88	61/18670	0.00299	0.008713	0.004155	3
BP	GO:00507	5/88	208/1867(	0.003032	0.008829	0.00421	5
BP	GO:00352	4/88	127/1867(	0.003065	0.008918	0.004253	4
BP	GO:00106	3/88	62/18670	0.003131	0.009079	0.00433	3
BP	GO:00316	3/88	62/18670	0.003131	0.009079	0.00433	3
BP	GO:00443	3/88	62/18670	0.003131	0.009079	0.00433	3
BP	GO:00454	3/88	62/18670	0.003131	0.009079	0.00433	3
BP	GO:00025	4/88	128/1867(	0.003153	0.009127	0.004353	4
BP	GO:00347	4/88	128/1867(	0.003153	0.009127	0.004353	4
BP	GO:00507	5/88	210/1867(	0.003159	0.009138	0.004358	5
BP	GO:00107	2/88	18/18670	0.0032	0.009172	0.004374	2
BP	GO:00319	2/88	18/18670	0.0032	0.009172	0.004374	2
BP	GO:00359	2/88	18/18670	0.0032	0.009172	0.004374	2
BP	GO:00396	2/88	18/18670	0.0032	0.009172	0.004374	2
BP	GO:00427	2/88	18/18670	0.0032	0.009172	0.004374	2
BP	GO:00457	2/88	18/18670	0.0032	0.009172	0.004374	2
BP	GO:00468	2/88	18/18670	0.0032	0.009172	0.004374	2
BP	GO:00702	2/88	18/18670	0.0032	0.009172	0.004374	2
BP	GO:00708	2/88	18/18670	0.0032	0.009172	0.004374	2
BP	GO:00713	2/88	18/18670	0.0032	0.009172	0.004374	2
BP	GO:00900	2/88	18/18670	0.0032	0.009172	0.004374	2
BP	GO:19030	4/88	129/1867(	0.003242	0.009286	0.004428	4
BP	GO:19043	3/88	63/18670	0.003276	0.009369	0.004468	3
BP	GO:20004	3/88	63/18670	0.003276	0.009369	0.004468	3
BP	GO:20010	5/88	214/1867(	0.003424	0.009773	0.004661	5
BP	GO:19004	3/88	64/18670	0.003426	0.009773	0.004661	3
BP	GO:19036	3/88	64/18670	0.003426	0.009773	0.004661	3
BP	GO:00703	5/88	215/1867(	0.003493	0.009956	0.004748	5
BP	GO:19907	6/88	311/1867(	0.003517	0.01001	0.004773	6
BP	GO:00031	4/88	132/1867(	0.00352	0.01001	0.004773	4
BP	GO:00424	4/88	132/1867(	0.00352	0.01001	0.004773	4
BP	GO:00025	2/88	19/18670	0.003565	0.010047	0.004791	2
BP	GO:00026	2/88	19/18670	0.003565	0.010047	0.004791	2
BP	GO:00030	2/88	19/18670	0.003565	0.010047	0.004791	2
BP	GO:00309	2/88	19/18670	0.003565	0.010047	0.004791	2
BP	GO:00329	2/88	19/18670	0.003565	0.010047	0.004791	2
BP	GO:00341	2/88	19/18670	0.003565	0.010047	0.004791	2
BP	GO:00450	2/88	19/18670	0.003565	0.010047	0.004791	2
BP	GO:00602	2/88	19/18670	0.003565	0.010047	0.004791	2

BP	GO:00607	2/88	19/18670	0.003565	0.010047	0.004791	2
BP	GO:00902	2/88	19/18670	0.003565	0.010047	0.004791	2
BP	GO:19026	2/88	19/18670	0.003565	0.010047	0.004791	2
BP	GO:00327	3/88	65/18670	0.00358	0.010072	0.004803	3
BP	GO:00726	3/88	65/18670	0.00358	0.010072	0.004803	3
BP	GO:00442	5/88	217/1867(	0.003633	0.010215	0.004871	5
BP	GO:00435	7/88	419/1867(	0.003638	0.010219	0.004873	7
BP	GO:00430	6/88	314/1867(	0.003686	0.010346	0.004934	6
BP	GO:00061	4/88	134/1867(	0.003715	0.010418	0.004968	4
BP	GO:00519	6/88	315/1867(	0.003743	0.01049	0.005003	6
BP	GO:00068	6/88	316/1867(	0.003802	0.010645	0.005077	6
BP	GO:00018	4/88	135/1867(	0.003815	0.010673	0.00509	4
BP	GO:00469	4/88	136/1867(	0.003916	0.010949	0.005221	4
BP	GO:00028	2/88	20/18670	0.003949	0.010953	0.005224	2
BP	GO:00065	2/88	20/18670	0.003949	0.010953	0.005224	2
BP	GO:00100	2/88	20/18670	0.003949	0.010953	0.005224	2
BP	GO:00161	2/88	20/18670	0.003949	0.010953	0.005224	2
BP	GO:00193	2/88	20/18670	0.003949	0.010953	0.005224	2
BP	GO:00302	2/88	20/18670	0.003949	0.010953	0.005224	2
BP	GO:00343	2/88	20/18670	0.003949	0.010953	0.005224	2
BP	GO:00602	2/88	20/18670	0.003949	0.010953	0.005224	2
BP	GO:00977	2/88	20/18670	0.003949	0.010953	0.005224	2
BP	GO:19029	2/88	20/18670	0.003949	0.010953	0.005224	2
BP	GO:00022	6/88	319/1867(	0.003981	0.011023	0.005257	6
BP	GO:00072	6/88	319/1867(	0.003981	0.011023	0.005257	6
BP	GO:00508	3/88	68/18670	0.004066	0.01125	0.005365	3
BP	GO:00091	4/88	138/1867(	0.004125	0.011369	0.005422	4
BP	GO:00091	4/88	138/1867(	0.004125	0.011369	0.005422	4
BP	GO:00093	4/88	138/1867(	0.004125	0.011369	0.005422	4
BP	GO:00466	4/88	138/1867(	0.004125	0.011369	0.005422	4
BP	GO:00610	4/88	138/1867(	0.004125	0.011369	0.005422	4
BP	GO:00507	4/88	139/1867(	0.004232	0.011648	0.005555	4
BP	GO:00080	3/88	69/18670	0.004236	0.011648	0.005555	3
BP	GO:00159	3/88	69/18670	0.004236	0.011648	0.005555	3
BP	GO:00018	4/88	140/1867(	0.004341	0.011835	0.005644	4
BP	GO:00091	4/88	140/1867(	0.004341	0.011835	0.005644	4
BP	GO:00346	4/88	140/1867(	0.004341	0.011835	0.005644	4
BP	GO:19033	4/88	140/1867(	0.004341	0.011835	0.005644	4
BP	GO:00108	2/88	21/18670	0.004352	0.011835	0.005644	2
BP	GO:00170	2/88	21/18670	0.004352	0.011835	0.005644	2
BP	GO:00351	2/88	21/18670	0.004352	0.011835	0.005644	2
BP	GO:00363	2/88	21/18670	0.004352	0.011835	0.005644	2
BP	GO:00467	2/88	21/18670	0.004352	0.011835	0.005644	2
BP	GO:00514	2/88	21/18670	0.004352	0.011835	0.005644	2
BP	GO:00519	2/88	21/18670	0.004352	0.011835	0.005644	2
BP	GO:00705	2/88	21/18670	0.004352	0.011835	0.005644	2
BP	GO:00714	2/88	21/18670	0.004352	0.011835	0.005644	2
BP	GO:19048	2/88	21/18670	0.004352	0.011835	0.005644	2
BP	GO:00330	3/88	70/18670	0.004411	0.011969	0.005708	3
BP	GO:00519	3/88	70/18670	0.004411	0.011969	0.005708	3
BP	GO:20012	3/88	70/18670	0.004411	0.011969	0.005708	3
BP	GO:00082	4/88	141/1867(	0.004452	0.012071	0.005757	4
BP	GO:19044	3/88	71/18670	0.00459	0.012435	0.00593	3
BP	GO:00310	4/88	143/1867(	0.00468	0.012659	0.006037	4
BP	GO:00443	4/88	143/1867(	0.00468	0.012659	0.006037	4
BP	GO:00427	6/88	330/1867(	0.00469	0.012678	0.006046	6
BP	GO:19033	5/88	231/1867(	0.004732	0.012774	0.006092	5
BP	GO:00324	2/88	22/18670	0.004772	0.012774	0.006092	2

BP	GO:00329	2/88	22/18670	0.004772	0.012774	0.006092	2
BP	GO:00355	2/88	22/18670	0.004772	0.012774	0.006092	2
BP	GO:00420	2/88	22/18670	0.004772	0.012774	0.006092	2
BP	GO:00456	2/88	22/18670	0.004772	0.012774	0.006092	2
BP	GO:00457	2/88	22/18670	0.004772	0.012774	0.006092	2
BP	GO:00510	2/88	22/18670	0.004772	0.012774	0.006092	2
BP	GO:00511	2/88	22/18670	0.004772	0.012774	0.006092	2
BP	GO:00600	2/88	22/18670	0.004772	0.012774	0.006092	2
BP	GO:00605	2/88	22/18670	0.004772	0.012774	0.006092	2
BP	GO:00903	2/88	22/18670	0.004772	0.012774	0.006092	2
BP	GO:00066	3/88	72/18670	0.004773	0.012774	0.006092	3
BP	GO:00076	4/88	145/1867(	0.004915	0.013133	0.006263	4
BP	GO:00076	4/88	145/1867(	0.004915	0.013133	0.006263	4
BP	GO:00335	3/88	73/18670	0.004961	0.013236	0.006312	3
BP	GO:00618	3/88	73/18670	0.004961	0.013236	0.006312	3
BP	GO:00485	5/88	234/1867(	0.004996	0.013318	0.006351	5
BP	GO:00605	5/88	235/1867(	0.005086	0.013547	0.00646	5
BP	GO:00059	3/88	74/18670	0.005153	0.013676	0.006522	3
BP	GO:00140	3/88	74/18670	0.005153	0.013676	0.006522	3
BP	GO:00301	3/88	74/18670	0.005153	0.013676	0.006522	3
BP	GO:00325	3/88	74/18670	0.005153	0.013676	0.006522	3
BP	GO:00996	3/88	74/18670	0.005153	0.013676	0.006522	3
BP	GO:00712	4/88	147/1867(	0.005158	0.013678	0.006523	4
BP	GO:00434	6/88	337/1867(	0.005188	0.013724	0.006545	6
BP	GO:00341	2/88	23/18670	0.005211	0.013724	0.006545	2
BP	GO:00360	2/88	23/18670	0.005211	0.013724	0.006545	2
BP	GO:00458	2/88	23/18670	0.005211	0.013724	0.006545	2
BP	GO:00509	2/88	23/18670	0.005211	0.013724	0.006545	2
BP	GO:00510	2/88	23/18670	0.005211	0.013724	0.006545	2
BP	GO:00513	2/88	23/18670	0.005211	0.013724	0.006545	2
BP	GO:00518	2/88	23/18670	0.005211	0.013724	0.006545	2
BP	GO:20006	2/88	23/18670	0.005211	0.013724	0.006545	2
BP	GO:00072	7/88	448/1867(	0.005228	0.013758	0.006561	7
BP	GO:00086	4/88	148/1867(	0.005283	0.013893	0.006625	4
BP	GO:00482	7/88	449/1867(	0.00529	0.013903	0.00663	7
BP	GO:00060	3/88	75/18670	0.00535	0.014028	0.00669	3
BP	GO:00074	3/88	75/18670	0.00535	0.014028	0.00669	3
BP	GO:00440	3/88	75/18670	0.00535	0.014028	0.00669	3
BP	GO:00302	5/88	238/1867(	0.005362	0.014039	0.006695	5
BP	GO:00516	5/88	238/1867(	0.005362	0.014039	0.006695	5
BP	GO:19039	4/88	149/1867(	0.005409	0.014151	0.006748	4
BP	GO:00082	4/88	150/1867(	0.005538	0.014455	0.006893	4
BP	GO:00487	4/88	150/1867(	0.005538	0.014455	0.006893	4
BP	GO:00717	4/88	150/1867(	0.005538	0.014455	0.006893	4
BP	GO:00019	3/88	76/18670	0.005551	0.014458	0.006895	3
BP	GO:00074	3/88	76/18670	0.005551	0.014458	0.006895	3
BP	GO:00454	3/88	76/18670	0.005551	0.014458	0.006895	3
BP	GO:00380	5/88	241/1867(	0.00565	0.014651	0.006987	5
BP	GO:00425	5/88	241/1867(	0.00565	0.014651	0.006987	5
BP	GO:00091	2/88	24/18670	0.005667	0.014651	0.006987	2
BP	GO:00421	2/88	24/18670	0.005667	0.014651	0.006987	2
BP	GO:00509	2/88	24/18670	0.005667	0.014651	0.006987	2
BP	GO:00601	2/88	24/18670	0.005667	0.014651	0.006987	2
BP	GO:00716	2/88	24/18670	0.005667	0.014651	0.006987	2
BP	GO:00900	2/88	24/18670	0.005667	0.014651	0.006987	2
BP	GO:20002	2/88	24/18670	0.005667	0.014651	0.006987	2
BP	GO:20010	2/88	24/18670	0.005667	0.014651	0.006987	2
BP	GO:00335	5/88	242/1867(	0.005748	0.014849	0.007081	5

BP	GO:00001	4/88	152/1867(	0.005801	0.014963	0.007136	4
BP	GO:00162	4/88	152/1867(	0.005801	0.014963	0.007136	4
BP	GO:00060	4/88	154/1867(	0.006072	0.015652	0.007464	4
BP	GO:00094	2/88	25/18670	0.006141	0.015691	0.007483	2
BP	GO:00190	2/88	25/18670	0.006141	0.015691	0.007483	2
BP	GO:00308	2/88	25/18670	0.006141	0.015691	0.007483	2
BP	GO:00316	2/88	25/18670	0.006141	0.015691	0.007483	2
BP	GO:00456	2/88	25/18670	0.006141	0.015691	0.007483	2
BP	GO:00456	2/88	25/18670	0.006141	0.015691	0.007483	2
BP	GO:00511	2/88	25/18670	0.006141	0.015691	0.007483	2
BP	GO:00603	2/88	25/18670	0.006141	0.015691	0.007483	2
BP	GO:00603	2/88	25/18670	0.006141	0.015691	0.007483	2
BP	GO:00900	2/88	25/18670	0.006141	0.015691	0.007483	2
BP	GO:19001	2/88	25/18670	0.006141	0.015691	0.007483	2
BP	GO:20006	2/88	25/18670	0.006141	0.015691	0.007483	2
BP	GO:00550	3/88	79/18670	0.006182	0.015771	0.007521	3
BP	GO:19000	3/88	79/18670	0.006182	0.015771	0.007521	3
BP	GO:19018	4/88	155/1867(	0.006211	0.015823	0.007546	4
BP	GO:19026	4/88	155/1867(	0.006211	0.015823	0.007546	4
BP	GO:00486	4/88	156/1867(	0.006352	0.01617	0.007711	4
BP	GO:00464	5/88	248/1867(	0.006363	0.016186	0.007719	5
BP	GO:00140	3/88	81/18670	0.006625	0.016715	0.007971	3
BP	GO:00305	3/88	81/18670	0.006625	0.016715	0.007971	3
BP	GO:00324	3/88	81/18670	0.006625	0.016715	0.007971	3
BP	GO:00023	2/88	26/18670	0.006633	0.016715	0.007971	2
BP	GO:00063	2/88	26/18670	0.006633	0.016715	0.007971	2
BP	GO:00220	2/88	26/18670	0.006633	0.016715	0.007971	2
BP	GO:00322	2/88	26/18670	0.006633	0.016715	0.007971	2
BP	GO:00435	2/88	26/18670	0.006633	0.016715	0.007971	2
BP	GO:00605	2/88	26/18670	0.006633	0.016715	0.007971	2
BP	GO:00607	2/88	26/18670	0.006633	0.016715	0.007971	2
BP	GO:00607	2/88	26/18670	0.006633	0.016715	0.007971	2
BP	GO:00716	2/88	26/18670	0.006633	0.016715	0.007971	2
BP	GO:19035	2/88	26/18670	0.006633	0.016715	0.007971	2
BP	GO:00091	4/88	158/1867(	0.00664	0.016722	0.007974	4
BP	GO:00026	4/88	159/1867(	0.006787	0.01708	0.008145	4
BP	GO:00514	6/88	357/1867(	0.006824	0.017162	0.008184	6
BP	GO:00512	3/88	82/18670	0.006853	0.017196	0.008201	3
BP	GO:00517	3/88	82/18670	0.006853	0.017196	0.008201	3
BP	GO:00900	3/88	82/18670	0.006853	0.017196	0.008201	3
BP	GO:20000	5/88	253/1867(	0.006909	0.017324	0.008262	5
BP	GO:00067	4/88	160/1867(	0.006937	0.017357	0.008277	4
BP	GO:00075	4/88	160/1867(	0.006937	0.017357	0.008277	4
BP	GO:00326	4/88	160/1867(	0.006937	0.017357	0.008277	4
BP	GO:00425	3/88	83/18670	0.007086	0.017673	0.008428	3
BP	GO:19038	3/88	83/18670	0.007086	0.017673	0.008428	3
BP	GO:20001	3/88	83/18670	0.007086	0.017673	0.008428	3
BP	GO:00326	4/88	161/1867(	0.007088	0.017673	0.008428	4
BP	GO:00359	4/88	161/1867(	0.007088	0.017673	0.008428	4
BP	GO:00020	2/88	27/18670	0.007141	0.017705	0.008443	2
BP	GO:00028	2/88	27/18670	0.007141	0.017705	0.008443	2
BP	GO:00067	2/88	27/18670	0.007141	0.017705	0.008443	2
BP	GO:00723	2/88	27/18670	0.007141	0.017705	0.008443	2
BP	GO:19016	2/88	27/18670	0.007141	0.017705	0.008443	2
BP	GO:19052	2/88	27/18670	0.007141	0.017705	0.008443	2
BP	GO:19055	2/88	27/18670	0.007141	0.017705	0.008443	2
BP	GO:20001	2/88	27/18670	0.007141	0.017705	0.008443	2
BP	GO:00905	5/88	256/1867(	0.007252	0.017966	0.008568	5

BP	GO:00326	4/88	163/1867(	0.007398	0.018302	0.008728	4
BP	GO:19035	4/88	163/1867(	0.007398	0.018302	0.008728	4
BP	GO:00434	5/88	258/1867(	0.007487	0.01851	0.008827	5
BP	GO:00070	4/88	164/1867(	0.007556	0.018665	0.008901	4
BP	GO:00020	3/88	85/18670	0.007566	0.018665	0.008901	3
BP	GO:00488	3/88	85/18670	0.007566	0.018665	0.008901	3
BP	GO:00030	5/88	259/1867(	0.007607	0.018743	0.008938	5
BP	GO:00031	2/88	28/18670	0.007667	0.018743	0.008938	2
BP	GO:00090	2/88	28/18670	0.007667	0.018743	0.008938	2
BP	GO:00331	2/88	28/18670	0.007667	0.018743	0.008938	2
BP	GO:00511	2/88	28/18670	0.007667	0.018743	0.008938	2
BP	GO:00602	2/88	28/18670	0.007667	0.018743	0.008938	2
BP	GO:00605	2/88	28/18670	0.007667	0.018743	0.008938	2
BP	GO:00711	2/88	28/18670	0.007667	0.018743	0.008938	2
BP	GO:19005	2/88	28/18670	0.007667	0.018743	0.008938	2
BP	GO:19020	2/88	28/18670	0.007667	0.018743	0.008938	2
BP	GO:19029	2/88	28/18670	0.007667	0.018743	0.008938	2
BP	GO:19907	2/88	28/18670	0.007667	0.018743	0.008938	2
BP	GO:20001	2/88	28/18670	0.007667	0.018743	0.008938	2
BP	GO:00157	7/88	482/1867(	0.007702	0.018816	0.008973	7
BP	GO:00509	4/88	165/1867(	0.007717	0.018837	0.008983	4
BP	GO:00324	5/88	260/1867(	0.007728	0.018839	0.008984	5
BP	GO:00726	5/88	260/1867(	0.007728	0.018839	0.008984	5
BP	GO:00072	3/88	86/18670	0.007813	0.01902	0.009071	3
BP	GO:00480	3/88	86/18670	0.007813	0.01902	0.009071	3
BP	GO:00350	4/88	167/1867(	0.008044	0.019565	0.00933	4
BP	GO:00061	3/88	87/18670	0.008065	0.019565	0.00933	3
BP	GO:00458	3/88	87/18670	0.008065	0.019565	0.00933	3
BP	GO:00486	3/88	87/18670	0.008065	0.019565	0.00933	3
BP	GO:00517	3/88	87/18670	0.008065	0.019565	0.00933	3
BP	GO:00325	6/88	370/1867(	0.008072	0.019568	0.009331	6
BP	GO:00000	2/88	29/18670	0.00821	0.019728	0.009408	2
BP	GO:00082	2/88	29/18670	0.00821	0.019728	0.009408	2
BP	GO:00140	2/88	29/18670	0.00821	0.019728	0.009408	2
BP	GO:00310	2/88	29/18670	0.00821	0.019728	0.009408	2
BP	GO:00356	2/88	29/18670	0.00821	0.019728	0.009408	2
BP	GO:00440	2/88	29/18670	0.00821	0.019728	0.009408	2
BP	GO:00459	2/88	29/18670	0.00821	0.019728	0.009408	2
BP	GO:00726	2/88	29/18670	0.00821	0.019728	0.009408	2
BP	GO:19000	2/88	29/18670	0.00821	0.019728	0.009408	2
BP	GO:19026	2/88	29/18670	0.00821	0.019728	0.009408	2
BP	GO:00019	4/88	168/1867(	0.008211	0.019728	0.009408	4
BP	GO:00513	4/88	168/1867(	0.008211	0.019728	0.009408	4
BP	GO:00717	4/88	168/1867(	0.008211	0.019728	0.009408	4
BP	GO:01400	5/88	264/1867(	0.008225	0.019749	0.009418	5
BP	GO:00610	3/88	88/18670	0.008322	0.019953	0.009515	3
BP	GO:19018	3/88	88/18670	0.008322	0.019953	0.009515	3
BP	GO:00380	4/88	169/1867(	0.00838	0.020066	0.009569	4
BP	GO:00605	4/88	169/1867(	0.00838	0.020066	0.009569	4
BP	GO:00434	4/88	170/1867(	0.008552	0.020463	0.009759	4
BP	GO:00069	4/88	171/1867(	0.008726	0.020851	0.009943	4
BP	GO:00092	4/88	171/1867(	0.008726	0.020851	0.009943	4
BP	GO:00107	2/88	30/18670	0.00877	0.020856	0.009946	2
BP	GO:00327	2/88	30/18670	0.00877	0.020856	0.009946	2
BP	GO:00427	2/88	30/18670	0.00877	0.020856	0.009946	2
BP	GO:00459	2/88	30/18670	0.00877	0.020856	0.009946	2
BP	GO:00701	2/88	30/18670	0.00877	0.020856	0.009946	2
BP	GO:00900	2/88	30/18670	0.00877	0.020856	0.009946	2

BP	GO:00974	2/88	30/18670	0.00877	0.020856	0.009946	2
BP	GO:00140	3/88	90/18670	0.008849	0.020988	0.010009	3
BP	GO:00326	3/88	90/18670	0.008849	0.020988	0.010009	3
BP	GO:00457	3/88	90/18670	0.008849	0.020988	0.010009	3
BP	GO:00468	3/88	90/18670	0.008849	0.020988	0.010009	3
BP	GO:00091	4/88	172/1867(	0.008902	0.021085	0.010055	4
BP	GO:00303	4/88	172/1867(	0.008902	0.021085	0.010055	4
BP	GO:00165	4/88	173/1867(	0.009081	0.021493	0.01025	4
BP	GO:00450	6/88	381/1867(	0.009249	0.021877	0.010433	6
BP	GO:20012	4/88	174/1867(	0.009262	0.021892	0.01044	4
BP	GO:00019	2/88	31/18670	0.009346	0.021973	0.010479	2
BP	GO:00026	2/88	31/18670	0.009346	0.021973	0.010479	2
BP	GO:00357	2/88	31/18670	0.009346	0.021973	0.010479	2
BP	GO:00400	2/88	31/18670	0.009346	0.021973	0.010479	2
BP	GO:00457	2/88	31/18670	0.009346	0.021973	0.010479	2
BP	GO:00458	2/88	31/18670	0.009346	0.021973	0.010479	2
BP	GO:19022	2/88	31/18670	0.009346	0.021973	0.010479	2
BP	GO:19022	2/88	31/18670	0.009346	0.021973	0.010479	2
BP	GO:20001	4/88	175/1867(	0.009445	0.02219	0.010582	4
BP	GO:00303	4/88	176/1867(	0.009631	0.022611	0.010783	4
BP	GO:00064	3/88	93/18670	0.009677	0.022644	0.010798	3
BP	GO:00075	3/88	93/18670	0.009677	0.022644	0.010798	3
BP	GO:00320	3/88	93/18670	0.009677	0.022644	0.010798	3
BP	GO:00352	3/88	93/18670	0.009677	0.022644	0.010798	3
BP	GO:00466	3/88	93/18670	0.009677	0.022644	0.010798	3
BP	GO:00092	4/88	177/1867(	0.009818	0.02296	0.010949	4
BP	GO:00019	2/88	32/18670	0.009939	0.023088	0.01101	2
BP	GO:00031	2/88	32/18670	0.009939	0.023088	0.01101	2
BP	GO:00396	2/88	32/18670	0.009939	0.023088	0.01101	2
BP	GO:00425	2/88	32/18670	0.009939	0.023088	0.01101	2
BP	GO:00455	2/88	32/18670	0.009939	0.023088	0.01101	2
BP	GO:00513	2/88	32/18670	0.009939	0.023088	0.01101	2
BP	GO:00550	2/88	32/18670	0.009939	0.023088	0.01101	2
BP	GO:00606	2/88	32/18670	0.009939	0.023088	0.01101	2
BP	GO:19007	2/88	32/18670	0.009939	0.023088	0.01101	2
BP	GO:19016	2/88	32/18670	0.009939	0.023088	0.01101	2
BP	GO:00701	3/88	94/18670	0.009962	0.023126	0.011029	3
BP	GO:00083	4/88	179/1867(	0.010201	0.023666	0.011286	4
BP	GO:00421	3/88	95/18670	0.010253	0.023737	0.01132	3
BP	GO:00516	3/88	95/18670	0.010253	0.023737	0.01132	3
BP	GO:20000	3/88	95/18670	0.010253	0.023737	0.01132	3
BP	GO:19017	4/88	180/1867(	0.010396	0.024055	0.011471	4
BP	GO:00327	3/88	96/18670	0.010548	0.024277	0.011577	3
BP	GO:00902	3/88	96/18670	0.010548	0.024277	0.011577	3
BP	GO:00027	2/88	33/18670	0.010548	0.024277	0.011577	2
BP	GO:00457	2/88	33/18670	0.010548	0.024277	0.011577	2
BP	GO:00511	2/88	33/18670	0.010548	0.024277	0.011577	2
BP	GO:00702	2/88	33/18670	0.010548	0.024277	0.011577	2
BP	GO:19018	2/88	33/18670	0.010548	0.024277	0.011577	2
BP	GO:19026	2/88	33/18670	0.010548	0.024277	0.011577	2
BP	GO:00022	4/88	181/1867(	0.010594	0.024335	0.011605	4
BP	GO:00224	4/88	181/1867(	0.010594	0.024335	0.011605	4
BP	GO:00511	4/88	181/1867(	0.010594	0.024335	0.011605	4
BP	GO:00091	4/88	182/1867(	0.010794	0.024762	0.011808	4
BP	GO:00611	4/88	182/1867(	0.010794	0.024762	0.011808	4
BP	GO:00070	3/88	97/18670	0.010848	0.024869	0.01186	3
BP	GO:00165	5/88	283/1867(	0.010893	0.024957	0.011902	5
BP	GO:00020	4/88	183/1867(	0.010996	0.025127	0.011983	4

BP	GO:00091	4/88	183/1867(	0.010996	0.025127	0.011983	4
BP	GO:00091	4/88	183/1867(	0.010996	0.025127	0.011983	4
BP	GO:00431	4/88	183/1867(	0.010996	0.025127	0.011983	4
BP	GO:00313	3/88	98/18670	0.011153	0.025399	0.012112	3
BP	GO:00007	2/88	34/18670	0.011173	0.025399	0.012112	2
BP	GO:00342	2/88	34/18670	0.011173	0.025399	0.012112	2
BP	GO:00450	2/88	34/18670	0.011173	0.025399	0.012112	2
BP	GO:00708	2/88	34/18670	0.011173	0.025399	0.012112	2
BP	GO:00714	2/88	34/18670	0.011173	0.025399	0.012112	2
BP	GO:01060	2/88	34/18670	0.011173	0.025399	0.012112	2
BP	GO:19040	2/88	34/18670	0.011173	0.025399	0.012112	2
BP	GO:00073	4/88	185/1867(	0.011408	0.025899	0.012351	4
BP	GO:19022	4/88	185/1867(	0.011408	0.025899	0.012351	4
BP	GO:00229	4/88	186/1867(	0.011618	0.026358	0.01257	4
BP	GO:00072	3/88	100/1867(	0.011778	0.026652	0.01271	3
BP	GO:00303	3/88	100/1867(	0.011778	0.026652	0.01271	3
BP	GO:00550	3/88	100/1867(	0.011778	0.026652	0.01271	3
BP	GO:00600	3/88	100/1867(	0.011778	0.026652	0.01271	3
BP	GO:00341	2/88	35/18670	0.011815	0.026684	0.012725	2
BP	GO:00602	2/88	35/18670	0.011815	0.026684	0.012725	2
BP	GO:19029	2/88	35/18670	0.011815	0.026684	0.012725	2
BP	GO:19013	4/88	187/1867(	0.01183	0.026701	0.012733	4
BP	GO:00067	6/88	403/1867(	0.011966	0.02699	0.012871	6
BP	GO:00091	4/88	188/1867(	0.012045	0.027098	0.012923	4
BP	GO:00439	4/88	188/1867(	0.012045	0.027098	0.012923	4
BP	GO:00517	4/88	188/1867(	0.012045	0.027098	0.012923	4
BP	GO:19012	4/88	188/1867(	0.012045	0.027098	0.012923	4
BP	GO:00326	3/88	101/1867(	0.012098	0.027183	0.012963	3
BP	GO:00466	3/88	101/1867(	0.012098	0.027183	0.012963	3
BP	GO:00027	2/88	36/18670	0.012472	0.027738	0.013228	2
BP	GO:00030	2/88	36/18670	0.012472	0.027738	0.013228	2
BP	GO:00107	2/88	36/18670	0.012472	0.027738	0.013228	2
BP	GO:00302	2/88	36/18670	0.012472	0.027738	0.013228	2
BP	GO:00309	2/88	36/18670	0.012472	0.027738	0.013228	2
BP	GO:00343	2/88	36/18670	0.012472	0.027738	0.013228	2
BP	GO:00343	2/88	36/18670	0.012472	0.027738	0.013228	2
BP	GO:00519	2/88	36/18670	0.012472	0.027738	0.013228	2
BP	GO:00713	2/88	36/18670	0.012472	0.027738	0.013228	2
BP	GO:00716	2/88	36/18670	0.012472	0.027738	0.013228	2
BP	GO:00900	2/88	36/18670	0.012472	0.027738	0.013228	2
BP	GO:00903	2/88	36/18670	0.012472	0.027738	0.013228	2
BP	GO:19026	2/88	36/18670	0.012472	0.027738	0.013228	2
BP	GO:19031	2/88	36/18670	0.012472	0.027738	0.013228	2
BP	GO:20003	2/88	36/18670	0.012472	0.027738	0.013228	2
BP	GO:20011	2/88	36/18670	0.012472	0.027738	0.013228	2
BP	GO:00002	6/88	407/1867(	0.012514	0.027815	0.013265	6
BP	GO:00326	3/88	103/1867(	0.012752	0.028291	0.013492	3
BP	GO:00347	3/88	103/1867(	0.012752	0.028291	0.013492	3
BP	GO:00356	3/88	103/1867(	0.012752	0.028291	0.013492	3
BP	GO:00018	5/88	296/1867(	0.013028	0.028883	0.013774	5
BP	GO:00192	3/88	104/1867(	0.013087	0.028942	0.013802	3
BP	GO:00324	3/88	104/1867(	0.013087	0.028942	0.013802	3
BP	GO:00987	3/88	104/1867(	0.013087	0.028942	0.013802	3
BP	GO:19018	3/88	104/1867(	0.013087	0.028942	0.013802	3
BP	GO:00016	2/88	37/18670	0.013144	0.028995	0.013827	2
BP	GO:00457	2/88	37/18670	0.013144	0.028995	0.013827	2
BP	GO:00715	2/88	37/18670	0.013144	0.028995	0.013827	2
BP	GO:19053	2/88	37/18670	0.013144	0.028995	0.013827	2

BP	GO:00085	3/88	105/1867(	0.013427	0.029582	0.014107	3
BP	GO:00451	3/88	105/1867(	0.013427	0.029582	0.014107	3
BP	GO:00706	5/88	299/1867(	0.013558	0.02985	0.014235	5
BP	GO:00022	3/88	106/1867(	0.013772	0.030284	0.014442	3
BP	GO:00022	2/88	38/18670	0.013832	0.030284	0.014442	2
BP	GO:00067	2/88	38/18670	0.013832	0.030284	0.014442	2
BP	GO:00109	2/88	38/18670	0.013832	0.030284	0.014442	2
BP	GO:00165	2/88	38/18670	0.013832	0.030284	0.014442	2
BP	GO:00482	2/88	38/18670	0.013832	0.030284	0.014442	2
BP	GO:00716	2/88	38/18670	0.013832	0.030284	0.014442	2
BP	GO:19058	2/88	38/18670	0.013832	0.030284	0.014442	2
BP	GO:20002	2/88	38/18670	0.013832	0.030284	0.014442	2
BP	GO:00017	4/88	196/1867(	0.013852	0.030308	0.014453	4
BP	GO:00091	4/88	197/1867(	0.014089	0.030789	0.014683	4
BP	GO:00097	4/88	197/1867(	0.014089	0.030789	0.014683	4
BP	GO:00019	3/88	107/1867(	0.014122	0.030841	0.014708	3
BP	GO:00605	4/88	198/1867(	0.014329	0.031274	0.014914	4
BP	GO:00090	3/88	108/1867(	0.014477	0.031529	0.015036	3
BP	GO:00469	3/88	108/1867(	0.014477	0.031529	0.015036	3
BP	GO:00995	3/88	108/1867(	0.014477	0.031529	0.015036	3
BP	GO:00022	2/88	39/18670	0.014536	0.031529	0.015036	2
BP	GO:00082	2/88	39/18670	0.014536	0.031529	0.015036	2
BP	GO:00190	2/88	39/18670	0.014536	0.031529	0.015036	2
BP	GO:00331	2/88	39/18670	0.014536	0.031529	0.015036	2
BP	GO:00335	2/88	39/18670	0.014536	0.031529	0.015036	2
BP	GO:00725	2/88	39/18670	0.014536	0.031529	0.015036	2
BP	GO:19022	2/88	39/18670	0.014536	0.031529	0.015036	2
BP	GO:00160	4/88	199/1867(	0.014572	0.031587	0.015063	4
BP	GO:00217	3/88	109/1867(	0.014837	0.032142	0.015328	3
BP	GO:00613	3/88	110/1867(	0.015202	0.032912	0.015695	3
BP	GO:00198	2/88	40/18670	0.015254	0.032945	0.015711	2
BP	GO:00420	2/88	40/18670	0.015254	0.032945	0.015711	2
BP	GO:00425	2/88	40/18670	0.015254	0.032945	0.015711	2
BP	GO:00451	2/88	40/18670	0.015254	0.032945	0.015711	2
BP	GO:00220	3/88	111/1867(	0.015572	0.033609	0.016028	3
BP	GO:00159	3/88	112/1867(	0.015947	0.034318	0.016366	3
BP	GO:00423	3/88	112/1867(	0.015947	0.034318	0.016366	3
BP	GO:00426	3/88	112/1867(	0.015947	0.034318	0.016366	3
BP	GO:19035	3/88	112/1867(	0.015947	0.034318	0.016366	3
BP	GO:00063	2/88	41/18670	0.015988	0.034318	0.016366	2
BP	GO:00308	2/88	41/18670	0.015988	0.034318	0.016366	2
BP	GO:00432	2/88	41/18670	0.015988	0.034318	0.016366	2
BP	GO:00610	2/88	41/18670	0.015988	0.034318	0.016366	2
BP	GO:00971	2/88	41/18670	0.015988	0.034318	0.016366	2
BP	GO:00971	4/88	205/1867(	0.016082	0.034497	0.016451	4
BP	GO:00326	3/88	113/1867(	0.016327	0.035002	0.016692	3
BP	GO:00302	3/88	114/1867(	0.016712	0.035706	0.017027	3
BP	GO:00302	3/88	114/1867(	0.016712	0.035706	0.017027	3
BP	GO:00454	3/88	114/1867(	0.016712	0.035706	0.017027	3
BP	GO:00065	2/88	42/18670	0.016737	0.035706	0.017027	2
BP	GO:00106	2/88	42/18670	0.016737	0.035706	0.017027	2
BP	GO:00421	2/88	42/18670	0.016737	0.035706	0.017027	2
BP	GO:00511	2/88	42/18670	0.016737	0.035706	0.017027	2
BP	GO:19031	2/88	42/18670	0.016737	0.035706	0.017027	2
BP	GO:00091	4/88	208/1867(	0.016872	0.035972	0.017155	4
BP	GO:00019	2/88	43/18670	0.0175	0.03713	0.017707	2
BP	GO:00108	2/88	43/18670	0.0175	0.03713	0.017707	2
BP	GO:00331	2/88	43/18670	0.0175	0.03713	0.017707	2

BP	GO:00420	2/88	43/18670	0.0175	0.03713	0.017707	2
BP	GO:00436	2/88	43/18670	0.0175	0.03713	0.017707	2
BP	GO:00457	2/88	43/18670	0.0175	0.03713	0.017707	2
BP	GO:00516	2/88	43/18670	0.0175	0.03713	0.017707	2
BP	GO:00713	2/88	43/18670	0.0175	0.03713	0.017707	2
BP	GO:00313	3/88	117/1867(	0.017898	0.03793	0.018088	3
BP	GO:00511	3/88	117/1867(	0.017898	0.03793	0.018088	3
BP	GO:00031	2/88	44/18670	0.018278	0.038571	0.018394	2
BP	GO:00380	2/88	44/18670	0.018278	0.038571	0.018394	2
BP	GO:00429	2/88	44/18670	0.018278	0.038571	0.018394	2
BP	GO:00617	2/88	44/18670	0.018278	0.038571	0.018394	2
BP	GO:00708	2/88	44/18670	0.018278	0.038571	0.018394	2
BP	GO:00716	2/88	44/18670	0.018278	0.038571	0.018394	2
BP	GO:19035	2/88	44/18670	0.018278	0.038571	0.018394	2
BP	GO:00019	2/88	45/18670	0.01907	0.040146	0.019145	2
BP	GO:00323	2/88	45/18670	0.01907	0.040146	0.019145	2
BP	GO:00484	2/88	45/18670	0.01907	0.040146	0.019145	2
BP	GO:19002	2/88	45/18670	0.01907	0.040146	0.019145	2
BP	GO:00026	4/88	216/1867(	0.019098	0.04018	0.019161	4
BP	GO:00170	3/88	120/1867(	0.019131	0.040201	0.019171	3
BP	GO:19030	3/88	120/1867(	0.019131	0.040201	0.019171	3
BP	GO:00017	3/88	121/1867(	0.019552	0.041036	0.019569	3
BP	GO:00486	3/88	121/1867(	0.019552	0.041036	0.019569	3
BP	GO:00071	2/88	46/18670	0.019877	0.041494	0.019788	2
BP	GO:00140	2/88	46/18670	0.019877	0.041494	0.019788	2
BP	GO:00433	2/88	46/18670	0.019877	0.041494	0.019788	2
BP	GO:00440	2/88	46/18670	0.019877	0.041494	0.019788	2
BP	GO:00513	2/88	46/18670	0.019877	0.041494	0.019788	2
BP	GO:00713	2/88	46/18670	0.019877	0.041494	0.019788	2
BP	GO:00716	2/88	46/18670	0.019877	0.041494	0.019788	2
BP	GO:00977	2/88	46/18670	0.019877	0.041494	0.019788	2
BP	GO:19000	2/88	46/18670	0.019877	0.041494	0.019788	2
BP	GO:00324	3/88	122/1867(	0.019978	0.041656	0.019865	3
BP	GO:19038	3/88	122/1867(	0.019978	0.041656	0.019865	3
BP	GO:00149	2/88	47/18670	0.020697	0.042952	0.020483	2
BP	GO:00319	2/88	47/18670	0.020697	0.042952	0.020483	2
BP	GO:00357	2/88	47/18670	0.020697	0.042952	0.020483	2
BP	GO:00425	2/88	47/18670	0.020697	0.042952	0.020483	2
BP	GO:00480	2/88	47/18670	0.020697	0.042952	0.020483	2
BP	GO:00504	2/88	47/18670	0.020697	0.042952	0.020483	2
BP	GO:19007	2/88	47/18670	0.020697	0.042952	0.020483	2
BP	GO:19035	2/88	47/18670	0.020697	0.042952	0.020483	2
BP	GO:00512	3/88	124/1867(	0.020845	0.043233	0.020617	3
BP	GO:00026	6/88	458/1867(	0.021135	0.043782	0.020879	6
BP	GO:00068	6/88	458/1867(	0.021135	0.043782	0.020879	6
BP	GO:00017	2/88	48/18670	0.021532	0.044342	0.021146	2
BP	GO:00022	2/88	48/18670	0.021532	0.044342	0.021146	2
BP	GO:00075	2/88	48/18670	0.021532	0.044342	0.021146	2
BP	GO:00080	2/88	48/18670	0.021532	0.044342	0.021146	2
BP	GO:00352	2/88	48/18670	0.021532	0.044342	0.021146	2
BP	GO:00468	2/88	48/18670	0.021532	0.044342	0.021146	2
BP	GO:00603	2/88	48/18670	0.021532	0.044342	0.021146	2
BP	GO:00726	2/88	48/18670	0.021532	0.044342	0.021146	2
BP	GO:01010	2/88	48/18670	0.021532	0.044342	0.021146	2
BP	GO:19055	2/88	48/18670	0.021532	0.044342	0.021146	2
BP	GO:00512	3/88	126/1867(	0.021733	0.044704	0.021319	3
BP	GO:00901	3/88	126/1867(	0.021733	0.044704	0.021319	3
BP	GO:00344	4/88	226/1867(	0.022126	0.045485	0.021691	4

BP	GO:00025	2/88	49/18670	0.02238	0.045793	0.021838	2
BP	GO:00030	2/88	49/18670	0.02238	0.045793	0.021838	2
BP	GO:00032	2/88	49/18670	0.02238	0.045793	0.021838	2
BP	GO:00431	2/88	49/18670	0.02238	0.045793	0.021838	2
BP	GO:00619	2/88	49/18670	0.02238	0.045793	0.021838	2
BP	GO:00713	2/88	49/18670	0.02238	0.045793	0.021838	2
BP	GO:00717	2/88	49/18670	0.02238	0.045793	0.021838	2
BP	GO:19015	2/88	49/18670	0.02238	0.045793	0.021838	2
BP	GO:00064	4/88	227/1867(	0.022444	0.045896	0.021887	4
BP	GO:00512	3/88	128/1867(	0.022641	0.046273	0.022067	3
BP	GO:00032	3/88	129/1867(	0.023103	0.047162	0.022491	3
BP	GO:00508	3/88	129/1867(	0.023103	0.047162	0.022491	3
BP	GO:00074	6/88	468/1867(	0.023205	0.047281	0.022548	6
BP	GO:00140	2/88	50/18670	0.023242	0.047281	0.022548	2
BP	GO:00455	2/88	50/18670	0.023242	0.047281	0.022548	2
BP	GO:00706	2/88	50/18670	0.023242	0.047281	0.022548	2
BP	GO:00707	2/88	50/18670	0.023242	0.047281	0.022548	2
BP	GO:01061	2/88	50/18670	0.023242	0.047281	0.022548	2
BP	GO:00550	6/88	471/1867(	0.023852	0.048493	0.023125	6
BP	GO:00512	3/88	131/1867(	0.024042	0.048824	0.023283	3
BP	GO:00550	3/88	131/1867(	0.024042	0.048824	0.023283	3
BP	GO:00019	2/88	51/18670	0.024118	0.04892	0.023329	2
BP	GO:20012	2/88	51/18670	0.024118	0.04892	0.023329	2
BP	GO:00723	3/88	132/1867(	0.02452	0.049706	0.023704	3
BP	GO:00031	2/88	52/18670	0.025007	0.050606	0.024133	2
BP	GO:00315	2/88	52/18670	0.025007	0.050606	0.024133	2
BP	GO:20006	2/88	52/18670	0.025007	0.050606	0.024133	2
BP	GO:00512	5/88	351/1867(	0.025164	0.050895	0.024271	5
BP	GO:00072	3/88	134/1867(	0.02549	0.051494	0.024556	3
BP	GO:00083	3/88	134/1867(	0.02549	0.051494	0.024556	3
BP	GO:00430	6/88	479/1867(	0.025636	0.051759	0.024683	6
BP	GO:00001	2/88	53/18670	0.025909	0.052101	0.024846	2
BP	GO:00069	2/88	53/18670	0.025909	0.052101	0.024846	2
BP	GO:00380	2/88	53/18670	0.025909	0.052101	0.024846	2
BP	GO:00433	2/88	53/18670	0.025909	0.052101	0.024846	2
BP	GO:00457	2/88	53/18670	0.025909	0.052101	0.024846	2
BP	GO:00605	2/88	53/18670	0.025909	0.052101	0.024846	2
BP	GO:19055	2/88	53/18670	0.025909	0.052101	0.024846	2
BP	GO:00603	3/88	135/1867(	0.025982	0.052188	0.024888	3
BP	GO:19033	3/88	135/1867(	0.025982	0.052188	0.024888	3
BP	GO:00510	4/88	238/1867(	0.026127	0.052449	0.025012	4
BP	GO:00224	5/88	357/1867(	0.026816	0.053787	0.02565	5
BP	GO:00326	2/88	54/18670	0.026825	0.053787	0.02565	2
BP	GO:19036	3/88	137/1867(	0.026983	0.054073	0.025787	3
BP	GO:00343	4/88	241/1867(	0.027191	0.05446	0.025971	4
BP	GO:00423	2/88	55/18670	0.027753	0.05549	0.026462	2
BP	GO:00480	2/88	55/18670	0.027753	0.05549	0.026462	2
BP	GO:00610	2/88	55/18670	0.027753	0.05549	0.026462	2
BP	GO:00027	3/88	139/1867(	0.028004	0.055928	0.026671	3
BP	GO:00455	3/88	139/1867(	0.028004	0.055928	0.026671	3
BP	GO:00463	3/88	140/1867(	0.028522	0.056865	0.027118	3
BP	GO:00600	3/88	140/1867(	0.028522	0.056865	0.027118	3
BP	GO:00975	3/88	140/1867(	0.028522	0.056865	0.027118	3
BP	GO:00067	2/88	56/18670	0.028694	0.057111	0.027235	2
BP	GO:00606	2/88	56/18670	0.028694	0.057111	0.027235	2
BP	GO:00901	2/88	56/18670	0.028694	0.057111	0.027235	2
BP	GO:00725	6/88	493/1867(	0.028969	0.057625	0.027481	6
BP	GO:19033	4/88	247/1867(	0.029396	0.058442	0.02787	4

BP	GO:00017	2/88	57/18670	0.029648	0.058842	0.028061	2
BP	GO:00480	2/88	57/18670	0.029648	0.058842	0.028061	2
BP	GO:00510	2/88	57/18670	0.029648	0.058842	0.028061	2
BP	GO:01061	3/88	143/1867(	0.030107	0.059686	0.028463	3
BP	GO:01201	3/88	143/1867(	0.030107	0.059686	0.028463	3
BP	GO:00421	6/88	498/1867(	0.030226	0.059887	0.028559	6
BP	GO:00024	6/88	499/1867(	0.030481	0.060359	0.028784	6
BP	GO:00600	3/88	144/1867(	0.030645	0.06065	0.028923	3
BP	GO:00018	2/88	59/18670	0.031593	0.062349	0.029733	2
BP	GO:00105	2/88	59/18670	0.031593	0.062349	0.029733	2
BP	GO:00328	2/88	59/18670	0.031593	0.062349	0.029733	2
BP	GO:00336	2/88	59/18670	0.031593	0.062349	0.029733	2
BP	GO:00610	2/88	59/18670	0.031593	0.062349	0.029733	2
BP	GO:00181	2/88	60/18670	0.032584	0.064233	0.030632	2
BP	GO:00330	2/88	60/18670	0.032584	0.064233	0.030632	2
BP	GO:00082	3/88	149/1867(	0.033414	0.065758	0.031359	3
BP	GO:00485	3/88	149/1867(	0.033414	0.065758	0.031359	3
BP	GO:20000	3/88	149/1867(	0.033414	0.065758	0.031359	3
BP	GO:00031	2/88	61/18670	0.033587	0.065914	0.031433	2
BP	GO:00074	2/88	61/18670	0.033587	0.065914	0.031433	2
BP	GO:00305	2/88	61/18670	0.033587	0.065914	0.031433	2
BP	GO:00341	2/88	61/18670	0.033587	0.065914	0.031433	2
BP	GO:00486	2/88	61/18670	0.033587	0.065914	0.031433	2
BP	GO:00163	3/88	150/1867(	0.033983	0.066616	0.031768	3
BP	GO:00507	3/88	150/1867(	0.033983	0.066616	0.031768	3
BP	GO:00550	3/88	151/1867(	0.034557	0.06749	0.032185	3
BP	GO:19031	3/88	151/1867(	0.034557	0.06749	0.032185	3
BP	GO:00065	2/88	62/18670	0.034602	0.06749	0.032185	2
BP	GO:00108	2/88	62/18670	0.034602	0.06749	0.032185	2
BP	GO:00326	2/88	62/18670	0.034602	0.06749	0.032185	2
BP	GO:00328	2/88	62/18670	0.034602	0.06749	0.032185	2
BP	GO:00466	2/88	62/18670	0.034602	0.06749	0.032185	2
BP	GO:00488	2/88	62/18670	0.034602	0.06749	0.032185	2
BP	GO:00550	2/88	62/18670	0.034602	0.06749	0.032185	2
BP	GO:00309	3/88	152/1867(	0.035136	0.068455	0.032645	3
BP	GO:00326	3/88	152/1867(	0.035136	0.068455	0.032645	3
BP	GO:00072	4/88	262/1867(	0.035364	0.068861	0.032839	4
BP	GO:00075	2/88	63/18670	0.03563	0.069147	0.032975	2
BP	GO:00323	2/88	63/18670	0.03563	0.069147	0.032975	2
BP	GO:00323	2/88	63/18670	0.03563	0.069147	0.032975	2
BP	GO:00429	2/88	63/18670	0.03563	0.069147	0.032975	2
BP	GO:00466	2/88	63/18670	0.03563	0.069147	0.032975	2
BP	GO:00901	2/88	63/18670	0.03563	0.069147	0.032975	2
BP	GO:00342	3/88	153/1867(	0.03572	0.069284	0.033041	3
BP	GO:00550	3/88	154/1867(	0.036309	0.070349	0.033548	3
BP	GO:19908	3/88	154/1867(	0.036309	0.070349	0.033548	3
BP	GO:00364	2/88	64/18670	0.036668	0.07081	0.033768	2
BP	GO:00435	2/88	64/18670	0.036668	0.07081	0.033768	2
BP	GO:00456	2/88	64/18670	0.036668	0.07081	0.033768	2
BP	GO:00456	2/88	64/18670	0.036668	0.07081	0.033768	2
BP	GO:00482	2/88	64/18670	0.036668	0.07081	0.033768	2
BP	GO:00509	2/88	64/18670	0.036668	0.07081	0.033768	2
BP	GO:00448	4/88	266/1867(	0.037066	0.071538	0.034116	4
BP	GO:00148	2/88	65/18670	0.037719	0.072718	0.034678	2
BP	GO:00511	2/88	65/18670	0.037719	0.072718	0.034678	2
BP	GO:00466	2/88	66/18670	0.038781	0.074683	0.035615	2
BP	GO:19052	2/88	66/18670	0.038781	0.074683	0.035615	2
BP	GO:20005	2/88	67/18670	0.039854	0.076708	0.036581	2

BP	GO:00028	3/88	160/1867(	0.039948	0.076805	0.036627	3
BP	GO:00302	3/88	160/1867(	0.039948	0.076805	0.036627	3
BP	GO:00614	4/88	273/1867(	0.040157	0.077163	0.036798	4
BP	GO:00109	3/88	161/1867(	0.040572	0.077876	0.037138	3
BP	GO:00991	3/88	161/1867(	0.040572	0.077876	0.037138	3
BP	GO:00062	4/88	274/1867(	0.04061	0.077906	0.037152	4
BP	GO:00108	2/88	68/18670	0.040939	0.078322	0.037351	2
BP	GO:00313	2/88	68/18670	0.040939	0.078322	0.037351	2
BP	GO:00359	2/88	68/18670	0.040939	0.078322	0.037351	2
BP	GO:00463	2/88	68/18670	0.040939	0.078322	0.037351	2
BP	GO:00712	2/88	68/18670	0.040939	0.078322	0.037351	2
BP	GO:00435	5/88	405/1867(	0.042535	0.081333	0.038786	5
BP	GO:00070	2/88	70/18670	0.043141	0.08231	0.039253	2
BP	GO:00105	2/88	70/18670	0.043141	0.08231	0.039253	2
BP	GO:00603	2/88	70/18670	0.043141	0.08231	0.039253	2
BP	GO:00720	2/88	70/18670	0.043141	0.08231	0.039253	2
BP	GO:00022	3/88	166/1867(	0.043766	0.083412	0.039778	3
BP	GO:00902	3/88	166/1867(	0.043766	0.083412	0.039778	3
BP	GO:00424	2/88	71/18670	0.044258	0.084259	0.040182	2
BP	GO:00425	2/88	71/18670	0.044258	0.084259	0.040182	2
BP	GO:19901	3/88	168/1867(	0.045077	0.085146	0.040605	3
BP	GO:00320	2/88	72/18670	0.045386	0.085146	0.040605	2
BP	GO:00508	2/88	72/18670	0.045386	0.085146	0.040605	2
BP	GO:00601	2/88	72/18670	0.045386	0.085146	0.040605	2
BP	GO:00456	3/88	169/1867(	0.045741	0.085146	0.040605	3
BP	GO:00022	1/88	10/18670	0.046158	0.085146	0.040605	1
BP	GO:00071	1/88	10/18670	0.046158	0.085146	0.040605	1
BP	GO:00107	1/88	10/18670	0.046158	0.085146	0.040605	1
BP	GO:00107	1/88	10/18670	0.046158	0.085146	0.040605	1
BP	GO:00109	1/88	10/18670	0.046158	0.085146	0.040605	1
BP	GO:00140	1/88	10/18670	0.046158	0.085146	0.040605	1
BP	GO:00190	1/88	10/18670	0.046158	0.085146	0.040605	1
BP	GO:00215	1/88	10/18670	0.046158	0.085146	0.040605	1
BP	GO:00303	1/88	10/18670	0.046158	0.085146	0.040605	1
BP	GO:00320	1/88	10/18670	0.046158	0.085146	0.040605	1
BP	GO:00320	1/88	10/18670	0.046158	0.085146	0.040605	1
BP	GO:00323	1/88	10/18670	0.046158	0.085146	0.040605	1
BP	GO:00340	1/88	10/18670	0.046158	0.085146	0.040605	1
BP	GO:00343	1/88	10/18670	0.046158	0.085146	0.040605	1
BP	GO:00358	1/88	10/18670	0.046158	0.085146	0.040605	1
BP	GO:00365	1/88	10/18670	0.046158	0.085146	0.040605	1
BP	GO:00436	1/88	10/18670	0.046158	0.085146	0.040605	1
BP	GO:00443	1/88	10/18670	0.046158	0.085146	0.040605	1
BP	GO:00445	1/88	10/18670	0.046158	0.085146	0.040605	1
BP	GO:00453	1/88	10/18670	0.046158	0.085146	0.040605	1
BP	GO:00456	1/88	10/18670	0.046158	0.085146	0.040605	1
BP	GO:00457	1/88	10/18670	0.046158	0.085146	0.040605	1
BP	GO:00457	1/88	10/18670	0.046158	0.085146	0.040605	1
BP	GO:00457	1/88	10/18670	0.046158	0.085146	0.040605	1
BP	GO:00459	1/88	10/18670	0.046158	0.085146	0.040605	1
BP	GO:00463	1/88	10/18670	0.046158	0.085146	0.040605	1
BP	GO:00511	1/88	10/18670	0.046158	0.085146	0.040605	1
BP	GO:00519	1/88	10/18670	0.046158	0.085146	0.040605	1
BP	GO:00522	1/88	10/18670	0.046158	0.085146	0.040605	1
BP	GO:00603	1/88	10/18670	0.046158	0.085146	0.040605	1
BP	GO:00605	1/88	10/18670	0.046158	0.085146	0.040605	1
BP	GO:00607	1/88	10/18670	0.046158	0.085146	0.040605	1
BP	GO:00612	1/88	10/18670	0.046158	0.085146	0.040605	1

BP	GO:00700	1/88	10/18670	0.046158	0.085146	0.040605	1
BP	GO:00703	1/88	10/18670	0.046158	0.085146	0.040605	1
BP	GO:00703	1/88	10/18670	0.046158	0.085146	0.040605	1
BP	GO:00709	1/88	10/18670	0.046158	0.085146	0.040605	1
BP	GO:00712	1/88	10/18670	0.046158	0.085146	0.040605	1
BP	GO:00721	1/88	10/18670	0.046158	0.085146	0.040605	1
BP	GO:00903	1/88	10/18670	0.046158	0.085146	0.040605	1
BP	GO:00905	1/88	10/18670	0.046158	0.085146	0.040605	1
BP	GO:01060	1/88	10/18670	0.046158	0.085146	0.040605	1
BP	GO:01100	1/88	10/18670	0.046158	0.085146	0.040605	1
BP	GO:01400	1/88	10/18670	0.046158	0.085146	0.040605	1
BP	GO:19001	1/88	10/18670	0.046158	0.085146	0.040605	1
BP	GO:19002	1/88	10/18670	0.046158	0.085146	0.040605	1
BP	GO:19021	1/88	10/18670	0.046158	0.085146	0.040605	1
BP	GO:19033	1/88	10/18670	0.046158	0.085146	0.040605	1
BP	GO:19038	1/88	10/18670	0.046158	0.085146	0.040605	1
BP	GO:19043	1/88	10/18670	0.046158	0.085146	0.040605	1
BP	GO:19047	1/88	10/18670	0.046158	0.085146	0.040605	1
BP	GO:20004	1/88	10/18670	0.046158	0.085146	0.040605	1
BP	GO:20010	1/88	10/18670	0.046158	0.085146	0.040605	1
BP	GO:20012	1/88	10/18670	0.046158	0.085146	0.040605	1
BP	GO:00060	3/88	170/1867(	0.046409	0.085563	0.040804	3
BP	GO:00456	2/88	73/18670	0.046525	0.085687	0.040863	2
BP	GO:19019	2/88	73/18670	0.046525	0.085687	0.040863	2
BP	GO:00032	3/88	171/1867(	0.047082	0.086667	0.04133	3
BP	GO:00485	4/88	288/1867(	0.047262	0.086953	0.041466	4
BP	GO:00018	2/88	74/18670	0.047674	0.087528	0.041741	2
BP	GO:00060	2/88	74/18670	0.047674	0.087528	0.041741	2
BP	GO:00090	2/88	74/18670	0.047674	0.087528	0.041741	2
BP	GO:19000	2/88	74/18670	0.047674	0.087528	0.041741	2
BP	GO:00328	3/88	172/1867(	0.047759	0.087638	0.041793	3
BP	GO:00431	5/88	419/1867(	0.047984	0.088005	0.041968	5
BP	GO:00343	4/88	290/1867(	0.048259	0.088462	0.042186	4
BP	GO:00703	3/88	173/1867(	0.048442	0.088751	0.042324	3
BP	GO:00019	2/88	75/18670	0.048834	0.089375	0.042622	2
BP	GO:00066	2/88	75/18670	0.048834	0.089375	0.042622	2
BP	GO:00026	3/88	175/1867(	0.049821	0.090581	0.043197	3
BP	GO:00109	3/88	175/1867(	0.049821	0.090581	0.043197	3
CC	GO:00451	15/88	315/19717	8.98E-12	1.13E-09	9.14E-10	15
CC	GO:00988	15/88	316/19717	9.39E-12	1.13E-09	9.14E-10	15
CC	GO:00985	15/88	328/19717	1.59E-11	1.28E-09	1.04E-09	15
CC	GO:00905	9/88	163/19717	4.72E-08	2.83E-06	2.30E-06	9
CC	GO:00447	9/88	201/19717	2.83E-07	1.36E-05	1.10E-05	9
CC	GO:00056	11/88	365/19717	6.74E-07	2.70E-05	2.19E-05	11
CC	GO:00007	10/88	377/19717	6.95E-06	0.000238	0.000193	10
CC	GO:00448	6/88	109/19717	9.32E-06	0.00028	0.000227	6
CC	GO:00059	5/88	80/19717	2.92E-05	0.000778	0.000631	5
CC	GO:00451	9/88	384/19717	5.41E-05	0.001297	0.001053	9
CC	GO:00098	9/88	393/19717	6.46E-05	0.001409	0.001143	9
CC	GO:00620	9/88	406/19717	8.28E-05	0.001657	0.001344	9
CC	GO:00099	3/88	34/19717	0.000465	0.007981	0.006476	3
CC	GO:00057	7/88	309/19717	0.000466	0.007981	0.006476	7
CC	GO:01201	4/88	84/19717	0.000544	0.008283	0.006721	4
CC	GO:00163	7/88	318/19717	0.000552	0.008283	0.006721	7
CC	GO:00319	7/88	339/19717	0.000805	0.011361	0.009219	7
CC	GO:00003	3/88	42/19717	0.00087	0.011594	0.009408	3
CC	GO:00057	5/88	178/19717	0.001213	0.015322	0.012432	5
CC	GO:00432	3/88	49/19717	0.001365	0.016374	0.013286	3

CC	GO:00451	3/88	51/19717	0.001532	0.017514	0.014211	3
CC	GO:00319	5/88	201/19717	0.00207	0.020318	0.016486	5
CC	GO:19041	3/88	57/19717	0.002112	0.020318	0.016486	3
CC	GO:00198	5/88	203/19717	0.002161	0.020318	0.016486	5
CC	GO:00059	7/88	405/19717	0.002231	0.020318	0.016486	7
CC	GO:00059	7/88	408/19717	0.002325	0.020318	0.016486	7
CC	GO:00301	6/88	303/19717	0.002366	0.020318	0.016486	6
CC	GO:00328	5/88	208/19717	0.002401	0.020318	0.016486	5
CC	GO:00300	7/88	412/19717	0.002455	0.020318	0.016486	7
CC	GO:00163	2/88	18/19717	0.002876	0.023011	0.018671	2
CC	GO:00347	6/88	321/19717	0.003148	0.024371	0.019775	6
CC	GO:00310	3/88	67/19717	0.003349	0.025117	0.02038	3
CC	GO:00602	6/88	338/19717	0.00405	0.029458	0.023902	6
CC	GO:00469	2/88	23/19717	0.004688	0.032876	0.026675	2
CC	GO:00057	6/88	350/19717	0.004794	0.032876	0.026675	6
CC	GO:00312	2/88	24/19717	0.005099	0.033996	0.027585	2
CC	GO:00430	7/88	497/19717	0.006801	0.044111	0.035792	7
CC	GO:19025	3/88	88/19717	0.007174	0.045307	0.036762	3
CC	GO:00310	3/88	91/19717	0.007866	0.048407	0.039277	3
CC	GO:00328	2/88	32/19717	0.008955	0.053729	0.043596	2
MF	GO:00037	8/88	56/17697	3.16E-10	1.10E-07	6.78E-08	8
MF	GO:00048	7/88	47/17697	3.16E-09	3.65E-07	2.26E-07	7
MF	GO:00985	7/88	47/17697	3.16E-09	3.65E-07	2.26E-07	7
MF	GO:00010	10/88	155/17697	4.99E-09	4.33E-07	2.68E-07	10
MF	GO:00020	9/88	128/17697	1.44E-08	9.99E-07	6.18E-07	9
MF	GO:00051	9/88	132/17697	1.89E-08	1.00E-06	6.21E-07	9
MF	GO:00316	12/88	290/17697	2.02E-08	1.00E-06	6.21E-07	12
MF	GO:00443	12/88	308/17697	3.93E-08	1.71E-06	1.06E-06	12
MF	GO:00167	8/88	159/17697	1.24E-06	4.80E-05	2.97E-05	8
MF	GO:00051	10/88	286/17697	1.54E-06	5.36E-05	3.32E-05	10
MF	GO:00199	8/88	185/17697	3.86E-06	0.000122	7.54E-05	8
MF	GO:00480	12/88	482/17697	4.57E-06	0.00013	8.06E-05	12
MF	GO:00200	7/88	135/17697	4.88E-06	0.00013	8.06E-05	7
MF	GO:00054	6/88	95/17697	7.76E-06	0.000181	0.000112	6
MF	GO:00469	7/88	145/17697	7.82E-06	0.000181	0.000112	7
MF	GO:00044	6/88	99/17697	9.84E-06	0.000213	0.000132	6
MF	GO:00012	11/88	439/17697	1.11E-05	0.000225	0.000139	11
MF	GO:00510	6/88	102/17697	1.17E-05	0.000225	0.000139	6
MF	GO:00051	8/88	220/17697	1.37E-05	0.00023	0.000142	8
MF	GO:00514	3/88	10/17697	1.39E-05	0.00023	0.000142	3
MF	GO:00705	3/88	10/17697	1.39E-05	0.00023	0.000142	3
MF	GO:00042	7/88	160/17697	1.49E-05	0.000235	0.000145	7
MF	GO:00977	4/88	33/17697	2.09E-05	0.000316	0.000195	4
MF	GO:00166	3/88	13/17697	3.28E-05	0.000474	0.000293	3
MF	GO:00082	7/88	182/17697	3.41E-05	0.000474	0.000293	7
MF	GO:00171	7/88	186/17697	3.92E-05	0.000523	0.000324	7
MF	GO:00167	4/88	39/17697	4.11E-05	0.000528	0.000327	4
MF	GO:00041	10/88	427/17697	5.07E-05	0.000619	0.000383	10
MF	GO:00971	3/88	15/17697	5.18E-05	0.000619	0.000383	3
MF	GO:00198	6/88	137/17697	6.21E-05	0.000718	0.000445	6
MF	GO:00199	6/88	140/17697	7.01E-05	0.000784	0.000485	6
MF	GO:00010	3/88	20/17697	0.000127	0.001382	0.000855	3
MF	GO:00508	10/88	499/17697	0.000183	0.001923	0.00119	10
MF	GO:00166	5/88	107/17697	0.000193	0.001974	0.001222	5
MF	GO:00428	5/88	111/17697	0.00023	0.002277	0.001409	5
MF	GO:00198	6/88	180/17697	0.000277	0.002668	0.001651	6
MF	GO:00310	5/88	119/17697	0.000317	0.002975	0.001841	5
MF	GO:00704	4/88	71/17697	0.00043	0.003928	0.002431	4

MF	GO:00167	3/88	32/17697	0.000531	0.004493	0.002781	3
MF	GO:00302	3/88	32/17697	0.000531	0.004493	0.002781	3
MF	GO:00517	3/88	32/17697	0.000531	0.004493	0.002781	3
MF	GO:00192	6/88	207/17697	0.000582	0.004808	0.002975	6
MF	GO:00055	3/88	34/17697	0.000636	0.005132	0.003176	3
MF	GO:00511	4/88	80/17697	0.000677	0.005228	0.003235	4
MF	GO:00011	3/88	35/17697	0.000693	0.005228	0.003235	3
MF	GO:00015	3/88	35/17697	0.000693	0.005228	0.003235	3
MF	GO:00198	3/88	36/17697	0.000753	0.005562	0.003442	3
MF	GO:00512	4/88	83/17697	0.000778	0.005621	0.003479	4
MF	GO:00336	4/88	85/17697	0.00085	0.006013	0.003721	4
MF	GO:00083	3/88	38/17697	0.000884	0.006013	0.003721	3
MF	GO:00162	3/88	38/17697	0.000884	0.006013	0.003721	3
MF	GO:00717	2/88	10/17697	0.001072	0.007153	0.004427	2
MF	GO:00518	3/88	41/17697	0.001105	0.007233	0.004476	3
MF	GO:00452	2/88	11/17697	0.001306	0.008239	0.005099	2
MF	GO:00526	2/88	11/17697	0.001306	0.008239	0.005099	2
MF	GO:00328	3/88	46/17697	0.001545	0.009509	0.005885	3
MF	GO:00048	2/88	12/17697	0.001562	0.009509	0.005885	2
MF	GO:00040	2/88	13/17697	0.00184	0.010913	0.006754	2
MF	GO:00080	3/88	49/17697	0.001856	0.010913	0.006754	3
MF	GO:00047	2/88	14/17697	0.00214	0.012173	0.007533	2
MF	GO:00360	2/88	14/17697	0.00214	0.012173	0.007533	2
MF	GO:00154	3/88	52/17697	0.002202	0.012324	0.007627	3
MF	GO:00514	5/88	185/17697	0.0023	0.012416	0.007684	5
MF	GO:00011	3/88	53/17697	0.002326	0.012416	0.007684	3
MF	GO:00050	3/88	53/17697	0.002326	0.012416	0.007684	3
MF	GO:00090	4/88	114/17697	0.002513	0.013211	0.008176	4
MF	GO:00314	5/88	193/17697	0.002762	0.014099	0.008725	5
MF	GO:00051	2/88	16/17697	0.002804	0.014099	0.008725	2
MF	GO:00101	2/88	16/17697	0.002804	0.014099	0.008725	2
MF	GO:00055	3/88	59/17697	0.003159	0.015264	0.009446	3
MF	GO:00971	3/88	59/17697	0.003159	0.015264	0.009446	3
MF	GO:00051	2/88	17/17697	0.003167	0.015264	0.009446	2
MF	GO:00431	5/88	205/17697	0.003576	0.016997	0.010519	5
MF	GO:00047	3/88	62/17697	0.003636	0.017051	0.010552	3
MF	GO:00048	3/88	63/17697	0.003804	0.017602	0.010893	3
MF	GO:00332	3/88	64/17697	0.003977	0.01816	0.011238	3
MF	GO:00020	3/88	66/17697	0.004337	0.019296	0.011941	3
MF	GO:00423	3/88	66/17697	0.004337	0.019296	0.011941	3
MF	GO:00192	3/88	67/17697	0.004524	0.019625	0.012145	3
MF	GO:00303	3/88	67/17697	0.004524	0.019625	0.012145	3
MF	GO:00611	5/88	219/17697	0.004729	0.020258	0.012537	5
MF	GO:00081	2/88	21/17697	0.004828	0.02043	0.012643	2
MF	GO:00037	6/88	319/17697	0.005147	0.021519	0.013317	6
MF	GO:00010	3/88	72/17697	0.005533	0.02254	0.013949	3
MF	GO:00010	3/88	72/17697	0.005533	0.02254	0.013949	3
MF	GO:00012	2/88	23/17697	0.005779	0.02254	0.013949	2
MF	GO:00441	2/88	23/17697	0.005779	0.02254	0.013949	2
MF	GO:00704	2/88	23/17697	0.005779	0.02254	0.013949	2
MF	GO:00162	4/88	144/17697	0.005781	0.02254	0.013949	4
MF	GO:00016	3/88	74/17697	0.005972	0.022771	0.014092	3
MF	GO:01040	3/88	74/17697	0.005972	0.022771	0.014092	3
MF	GO:00352	4/88	152/17697	0.006981	0.026331	0.016295	4
MF	GO:00191	3/88	79/17697	0.007157	0.026703	0.016525	3
MF	GO:00040	2/88	26/17697	0.007352	0.026781	0.016573	2
MF	GO:00302	3/88	80/17697	0.007409	0.026781	0.016573	3
MF	GO:00463	3/88	80/17697	0.007409	0.026781	0.016573	3

MF	GO:00080	3/88	82/17697	0.00793	0.028367	0.017555	3
MF	GO:00162	3/88	86/17697	0.009034	0.031253	0.01934	3
MF	GO:00192	3/88	86/17697	0.009034	0.031253	0.01934	3
MF	GO:00350	2/88	29/17697	0.009097	0.031253	0.01934	2
MF	GO:00510	2/88	29/17697	0.009097	0.031253	0.01934	2
MF	GO:00303	2/88	30/17697	0.009715	0.03273	0.020254	2
MF	GO:00433	2/88	30/17697	0.009715	0.03273	0.020254	2
MF	GO:00051	2/88	31/17697	0.010352	0.034539	0.021374	2
MF	GO:00352	3/88	92/17697	0.010853	0.035865	0.022195	3
MF	GO:00048	6/88	375/17697	0.010987	0.035967	0.022258	6
MF	GO:00166	2/88	35/17697	0.013077	0.042408	0.026244	2
MF	GO:00082	2/88	37/17697	0.014544	0.046729	0.028918	2
MF	GO:00042	3/88	103/17697	0.0147	0.046797	0.02896	3
MF	GO:00165	2/88	38/17697	0.015303	0.048273	0.029873	2
MF	GO:00422	5/88	295/17697	0.015853	0.049557	0.030668	5
MF	GO:00017	2/88	40/17697	0.016871	0.052268	0.032346	2
MF	GO:00055	4/88	200/17697	0.01767	0.054261	0.033579	4
MF	GO:00303	2/88	42/17697	0.018504	0.055833	0.034552	2
MF	GO:00305	2/88	42/17697	0.018504	0.055833	0.034552	2
MF	GO:00012	2/88	43/17697	0.019344	0.056886	0.035203	2
MF	GO:00047	2/88	43/17697	0.019344	0.056886	0.035203	2
MF	GO:00169	2/88	43/17697	0.019344	0.056886	0.035203	2
MF	GO:00041	3/88	116/17697	0.020123	0.058677	0.036312	3
MF	GO:00991	3/88	118/17697	0.021042	0.059937	0.037091	3
MF	GO:00010	2/88	45/17697	0.021073	0.059937	0.037091	2
MF	GO:00481	2/88	45/17697	0.021073	0.059937	0.037091	2
MF	GO:00046	6/88	439/17697	0.022166	0.062532	0.038697	6
MF	GO:00165	2/88	49/17697	0.024715	0.069161	0.0428	2
MF	GO:00708	2/88	50/17697	0.025662	0.071239	0.044085	2
MF	GO:00453	2/88	51/17697	0.026625	0.073324	0.045376	2
MF	GO:00506	2/88	52/17697	0.027602	0.075416	0.04667	2
MF	GO:00047	3/88	134/17697	0.029219	0.078597	0.048639	3
MF	GO:00708	3/88	134/17697	0.029219	0.078597	0.048639	3

KEGG analysis

ID	GeneRatic	BgRatio	pvalue	p.adjust	qvalue	Count
hsa05417	29/85	215/8108	3.53E-25	7.83E-23	2.79E-23	29
hsa05418	24/85	139/8108	2.01E-23	2.24E-21	7.95E-22	24
hsa04933	20/85	100/8108	6.71E-21	4.96E-19	1.76E-19	20
hsa04657	16/85	94/8108	1.17E-15	6.49E-14	2.31E-14	16
hsa01522	16/85	98/8108	2.34E-15	1.04E-13	3.69E-14	16
hsa05161	18/85	162/8108	3.79E-14	1.39E-12	4.95E-13	18
hsa05215	15/85	97/8108	4.39E-14	1.39E-12	4.95E-13	15
hsa05210	14/85	86/8108	1.58E-13	4.39E-12	1.56E-12	14
hsa05160	17/85	157/8108	3.26E-13	7.14E-12	2.54E-12	17
hsa05207	19/85	212/8108	3.55E-13	7.14E-12	2.54E-12	19
hsa01524	13/85	73/8108	3.72E-13	7.14E-12	2.54E-12	13
hsa04668	15/85	112/8108	3.93E-13	7.14E-12	2.54E-12	15
hsa05222	14/85	92/8108	4.18E-13	7.14E-12	2.54E-12	14
hsa05167	18/85	194/8108	8.90E-13	1.41E-11	5.02E-12	18
hsa05219	10/85	41/8108	8.02E-12	1.19E-10	4.22E-11	10
hsa05162	15/85	139/8108	9.80E-12	1.36E-10	4.84E-11	15
hsa05163	17/85	225/8108	1.13E-10	1.47E-09	5.23E-10	17
hsa04115	11/85	73/8108	1.82E-10	2.24E-09	7.97E-10	11
hsa05224	14/85	147/8108	2.80E-10	3.02E-09	1.07E-09	14
hsa05133	11/85	76/8108	2.85E-10	3.02E-09	1.07E-09	11
hsa05212	11/85	76/8108	2.85E-10	3.02E-09	1.07E-09	11
hsa05142	12/85	102/8108	5.00E-10	5.05E-09	1.79E-09	12

hsa04932	14/85	155/8108	5.70E-10	5.50E-09	1.96E-09	14
hsa04620	12/85	104/8108	6.29E-10	5.82E-09	2.07E-09	12
hsa04215	8/85	32/8108	8.94E-10	7.94E-09	2.82E-09	8
hsa04066	12/85	109/8108	1.09E-09	9.34E-09	3.32E-09	12
hsa04210	13/85	136/8108	1.23E-09	1.01E-08	3.60E-09	13
hsa04915	13/85	138/8108	1.48E-09	1.17E-08	4.17E-09	13
hsa05225	14/85	168/8108	1.66E-09	1.27E-08	4.51E-09	14
hsa05169	15/85	202/8108	2.04E-09	1.51E-08	5.37E-09	15
hsa05205	15/85	205/8108	2.50E-09	1.79E-08	6.37E-09	15
hsa05152	14/85	180/8108	4.09E-09	2.84E-08	1.01E-08	14
hsa01521	10/85	79/8108	7.47E-09	5.03E-08	1.79E-08	10
hsa04926	12/85	129/8108	7.72E-09	5.04E-08	1.79E-08	12
hsa04151	18/85	354/8108	1.79E-08	1.14E-07	4.04E-08	18
hsa05145	11/85	112/8108	1.93E-08	1.19E-07	4.23E-08	11
hsa05164	13/85	172/8108	2.19E-08	1.32E-07	4.68E-08	13
hsa04010	16/85	294/8108	4.88E-08	2.81E-07	1.00E-07	16
hsa05223	9/85	72/8108	4.94E-08	2.81E-07	1.00E-07	9
hsa04380	11/85	128/8108	7.81E-08	4.33E-07	1.54E-07	11
hsa05140	9/85	77/8108	8.98E-08	4.86E-07	1.73E-07	9
hsa04064	10/85	104/8108	1.09E-07	5.79E-07	2.06E-07	10
hsa05213	8/85	58/8108	1.31E-07	6.75E-07	2.40E-07	8
hsa05165	16/85	331/8108	2.51E-07	1.27E-06	4.50E-07	16
hsa05226	11/85	149/8108	3.71E-07	1.82E-06	6.49E-07	11
hsa05166	13/85	219/8108	3.78E-07	1.82E-06	6.49E-07	13
hsa05323	9/85	93/8108	4.67E-07	2.21E-06	7.85E-07	9
hsa04917	8/85	70/8108	5.82E-07	2.67E-06	9.49E-07	8
hsa04218	11/85	156/8108	5.89E-07	2.67E-06	9.49E-07	11
hsa05218	8/85	72/8108	7.25E-07	3.22E-06	1.14E-06	8
hsa05144	7/85	50/8108	7.52E-07	3.27E-06	1.16E-06	7
hsa05130	12/85	197/8108	8.43E-07	3.60E-06	1.28E-06	12
hsa05214	8/85	75/8108	9.96E-07	4.17E-06	1.48E-06	8
hsa05146	9/85	102/8108	1.03E-06	4.24E-06	1.51E-06	9
hsa05220	8/85	76/8108	1.10E-06	4.46E-06	1.58E-06	8
hsa05135	10/85	137/8108	1.45E-06	5.76E-06	2.05E-06	10
hsa05131	13/85	247/8108	1.49E-06	5.80E-06	2.06E-06	13
hsa04659	9/85	107/8108	1.55E-06	5.92E-06	2.10E-06	9
hsa05132	13/85	249/8108	1.63E-06	6.13E-06	2.18E-06	13
hsa04370	7/85	59/8108	2.38E-06	8.81E-06	3.13E-06	7
hsa04012	8/85	85/8108	2.61E-06	9.50E-06	3.38E-06	8
hsa05202	11/85	192/8108	4.56E-06	1.63E-05	5.81E-06	11
hsa05171	12/85	232/8108	4.70E-06	1.66E-05	5.89E-06	12
hsa04510	11/85	201/8108	7.09E-06	2.46E-05	8.75E-06	11
hsa05170	11/85	212/8108	1.18E-05	3.94E-05	1.40E-05	11
hsa04625	8/85	104/8108	1.19E-05	3.94E-05	1.40E-05	8
hsa04660	8/85	104/8108	1.19E-05	3.94E-05	1.40E-05	8
hsa04621	10/85	185/8108	2.13E-05	6.95E-05	2.47E-05	10
hsa05020	12/85	273/8108	2.45E-05	7.87E-05	2.80E-05	12
hsa05134	6/85	57/8108	2.63E-05	8.34E-05	2.96E-05	6
hsa04722	8/85	119/8108	3.19E-05	9.97E-05	3.54E-05	8
hsa05416	6/85	60/8108	3.54E-05	0.000109	3.88E-05	6
hsa05235	7/85	89/8108	3.73E-05	0.000113	4.02E-05	7
hsa05216	5/85	37/8108	3.77E-05	0.000113	4.02E-05	5
hsa05415	10/85	203/8108	4.73E-05	0.00014	4.98E-05	10
hsa05231	7/85	98/8108	6.94E-05	0.000203	7.21E-05	7
hsa05206	12/85	310/8108	8.46E-05	0.000244	8.67E-05	12
hsa04137	6/85	72/8108	9.98E-05	0.000284	0.000101	6
hsa05022	15/85	476/8108	0.000108	0.000304	0.000108	15
hsa04913	5/85	51/8108	0.000181	0.000502	0.000179	5

hsa04071	7/85	119/8108	0.000236	0.000646	0.00023	7
hsa04217	8/85	159/8108	0.000245	0.000664	0.000236	8
hsa04919	7/85	121/8108	0.000261	0.000698	0.000248	7
hsa05203	9/85	204/8108	0.000267	0.000705	0.000251	9
hsa04068	7/85	131/8108	0.000424	0.001108	0.000394	7
hsa05143	4/85	37/8108	0.000571	0.001473	0.000524	4
hsa04371	7/85	138/8108	0.000581	0.001482	0.000527	7
hsa04914	6/85	100/8108	0.000603	0.001522	0.000541	6
hsa05211	5/85	69/8108	0.000747	0.001863	0.000662	5
hsa05120	5/85	70/8108	0.000798	0.001946	0.000692	5
hsa05230	5/85	70/8108	0.000798	0.001946	0.000692	5
hsa04928	6/85	106/8108	0.000821	0.001981	0.000705	6
hsa04931	6/85	108/8108	0.000906	0.002162	0.000769	6
hsa04921	7/85	154/8108	0.001112	0.002626	0.000934	7
hsa04934	7/85	155/8108	0.001155	0.002698	0.00096	7
hsa04670	6/85	114/8108	0.001201	0.002777	0.000988	6
hsa04630	7/85	162/8108	0.001492	0.003396	0.001208	7
hsa04935	6/85	119/8108	0.001499	0.003396	0.001208	6
hsa05010	11/85	369/8108	0.001541	0.003455	0.001229	11
hsa04662	5/85	82/8108	0.00163	0.003619	0.001287	5
hsa04110	6/85	124/8108	0.001851	0.004068	0.001447	6
hsa04610	5/85	85/8108	0.001912	0.004162	0.00148	5
hsa04658	5/85	92/8108	0.002708	0.005836	0.002076	5
hsa04912	5/85	93/8108	0.002839	0.006059	0.002155	5
hsa04014	8/85	232/8108	0.002877	0.006083	0.002163	8
hsa04910	6/85	137/8108	0.003058	0.006404	0.002277	6
hsa04140	6/85	141/8108	0.003527	0.007317	0.002602	6
hsa00140	4/85	61/8108	0.003728	0.007663	0.002725	4
hsa04062	7/85	192/8108	0.003891	0.007925	0.002818	7
hsa05217	4/85	63/8108	0.004188	0.008453	0.003006	4
hsa04929	4/85	64/8108	0.004432	0.008865	0.003152	4
hsa05321	4/85	65/8108	0.004686	0.009288	0.003303	4
hsa05221	4/85	67/8108	0.005222	0.010258	0.003648	4
hsa04920	4/85	69/8108	0.005797	0.011191	0.00398	4
hsa05204	4/85	69/8108	0.005797	0.011191	0.00398	4
hsa04622	4/85	70/8108	0.0061	0.011674	0.004152	4
hsa04024	7/85	219/8108	0.007905	0.014999	0.005334	7
hsa03320	4/85	76/8108	0.008141	0.015316	0.005447	4
hsa05332	3/85	42/8108	0.00951	0.017684	0.006289	3
hsa04611	5/85	124/8108	0.009559	0.017684	0.006289	5
hsa04940	3/85	43/8108	0.010147	0.018616	0.00662	3
hsa04060	8/85	295/8108	0.011866	0.021592	0.007679	8
hsa04930	3/85	46/8108	0.012204	0.022027	0.007833	3
hsa05014	9/85	365/8108	0.013836	0.024771	0.008809	9
hsa04211	4/85	89/8108	0.013988	0.024842	0.008834	4
hsa05168	11/85	498/8108	0.014431	0.025426	0.009042	11
hsa05016	8/85	306/8108	0.014548	0.025431	0.009044	8
hsa05012	7/85	249/8108	0.015294	0.026526	0.009433	7
hsa00330	3/85	51/8108	0.016129	0.027757	0.009871	3
hsa04072	5/85	148/8108	0.019328	0.033006	0.011738	5
hsa04640	4/85	99/8108	0.019941	0.033792	0.012017	4
hsa04061	4/85	100/8108	0.02061	0.034662	0.012326	4
hsa04923	3/85	57/8108	0.021671	0.036173	0.012864	3
hsa00220	2/85	22/8108	0.021902	0.036285	0.012904	2
hsa04310	5/85	166/8108	0.029907	0.048963	0.017412	5
hsa00790	2/85	26/8108	0.029995	0.048963	0.017412	2
hsa04725	4/85	113/8108	0.030576	0.0492	0.017496	4
hsa04022	5/85	167/8108	0.030584	0.0492	0.017496	5

hsa04141	5/85	171/8108	0.033387	0.053323	0.018963	5
hsa04664	3/85	68/8108	0.03421	0.054248	0.019291	3
hsa04152	4/85	120/8108	0.036942	0.058164	0.020684	4
hsa04020	6/85	240/8108	0.039716	0.062091	0.022081	6
hsa04114	4/85	129/8108	0.046172	0.071679	0.025491	4
hsa00980	3/85	78/8108	0.048238	0.073569	0.026162	3
hsa04612	3/85	78/8108	0.048238	0.073569	0.026162	3
hsa04650	4/85	131/8108	0.048383	0.073569	0.026162	4
hsa04728	4/85	132/8108	0.04951	0.074771	0.02659	4
