# Supplementary material for: Identification of Bioactive Compounds and Potential Mechanisms of Kuntai Capsule in the Treatment of Polycystic Ovary Syndrome by Integrating Network Pharmacology and Bioinformatics
Source: Oxid Med Cell Longev. 2022 Apr 28;2022:3145938. doi: 10.1155/2022/3145938 (PMC9073551; doi:10.1155/2022/3145938)
Supplement: Supplementary 6 — Supplement Table 6: molecular docking results of TP53, MAPK1, MAPK8, JUN, AKT1, and their corresponding effective ingredients. [file 3145938.f6.pdf]

AKT1-baicalein

mode	affinity(kcal/mol)	dist from best mode（rmsd l.b.）
1	-7.3	0
2	-7.2	1.041
3	-6.8	2.674
4	-6.7	2.623
5	-6.7	3.115
6	-6.6	10.512
7	-6.4	24
8	-6.3	10.385
9	-6.3	1.901
10	-6.3	1.63
11	-6.2	28.275
12	-6.1	18.641
13	-5.7	25.874
14	-5.6	21.896
15	-5.4	23.252
16	-5.4	19.621
17	-5.3	25.372
18	-5.3	28.741
19	-5.1	11.59
20	-5.1	10.351

AKT1-kaempferol

mode	affinity(kcal/mol)	dist from best mode（rmsd l.b.）
1	-6.7	0
2	-6.6	2.876
3	-6.6	1.525
4	-6.5	3.773
5	-6.5	3.325
6	-6.1	3.394
7	-6.1	1.684
8	-6	26.605
9	-6	17.48
10	-5.9	22.62
11	-5.8	23.467
12	-5.7	24.194
13	-5.7	3.203
14	-5.6	26.36
15	-5.6	24.202
16	-5.6	17.49
17	-5.6	26.571
18	-5.6	22.325
19	-5.6	24.821
20	-5.5	24.2

AKT1-quercetin

mode	affinity(kcal/mol)	dist from best mode（rmsd l.b.）
1	-6.9	0
2	-6.8	1.536
3	-6.7	2.891
4	-6.5	2.385
5	-6.4	9.906
6	-6.3	16.784
7	-6.1	2.231
8	-6	26.61
9	-6	22.319
10	-5.9	22.686

11	-5.9	2.735
12	-5.8	24.921
13	-5.7	21.217
14	-5.7	26.72
15	-5.7	18.136
16	-5.7	20.703
17	-5.6	24.245
18	-5.6	24.174
19	-5.4	18.536
20	-5.3	24.419

AKT1-wogonin

mode	affinity(kcal/mol)	dist from best mode（rmsd l.b.）
1	-7.3	0
2	-6.8	2.057
3	-6.7	1.268
4	-6.6	2.676
5	-6.1	10.281
6	-5.9	26.089
7	-5.9	20.786
8	-5.9	18.168
9	-5.8	28.225
10	-5.8	28.708
11	-5.4	20.425
12	-5.4	25.571
13	-5.3	23.052
14	-5.3	28.654
15	-5.2	19.216
16	-5.2	24.422
17	-5.2	18.987
18	-5.1	25.42
19	-5.1	25.322
20	-5.1	18.642

JUN-beta-sitostero

mode	affinity(kcal/mol)	dist from best mode（rmsd l.b.）
1	-6.9	0
2	-6.8	2.188
3	-6.8	15.638
4	-6.7	13.929
5	-6.6	13.927
6	-6.5	3.488
7	-6.5	13.397
8	-6.3	14.011
9	-6.3	13.563
10	-6.2	5.598
11	-6.1	13.846
12	-6.1	13.568
13	-5.9	13.694
14	-5.9	15.252
15	-5.8	6.48
16	-5.7	14.968
17	-5.6	14.286
18	-5.6	35.943
19	-5.5	7.896
20	-5.4	4.75

JUN-kaempferol

mode	affinity(kcal/mol)	dist from best mode（rmsd l.b.）
1	-8.1	0
2	-8	13.267
3	-7.9	23.7
4	-7.9	24.012
5	-7.8	10.793
6	-7.8	24.424
7	-7.7	5.769
8	-7.7	27.715
9	-7.6	25.357
10	-7.5	8.045
11	-7.5	18.545
12	-7.4	29.246
13	-7.4	14.139
14	-7.4	26.102
15	-7.3	21.982
16	-7.3	5.955
17	-7.2	1.561
18	-7.2	1.616
19	-7.2	26.649
20	-7.2	14.816

JUN-quercetin

mode	affinity(kcal/mol)	dist from best mode（rmsd l.b.）
1	-8.6	0
2	-8.5	23.601
3	-8.5	25.423
4	-8.3	10.894
5	-8.2	23.974
6	-8.1	25.671
7	-8	5.92
8	-8	1.669
9	-8	5.743
10	-7.9	24.435
11	-7.9	29.058
12	-7.7	26.353
13	-7.7	30.953
14	-7.6	1.799
15	-7.6	1.8
16	-7.5	4.397
17	-7.5	2.399
18	-7.5	3.849
19	-7.4	1.728
20	-7.2	10.996

JUN-wogonin

mode	affinity(kcal/mol)	dist from best mode（rmsd l.b.）
1	-7.5	0
2	-7.2	14.141
3	-7.1	13.969
4	-6.9	28.399
5	-6.9	31.786
6	-6.9	13.666
7	-6.8	13.679
8	-6.7	13.449
9	-6.6	32.281
10	-6.5	13.844
11	-6.5	33.369

12	-6.5	14.037
13	-6.4	31.305
14	-6.4	14.276
15	-6.2	13.833
16	-6.2	9.722
17	-6.1	13.06
18	-5.9	14.376
19	-5.8	14.969
20	-5.7	9.697

MAPK1-quercetin

mode	affinity(kcal/mol)	dist from best mode（rmsd l.b.）
1	-8.7	0
2	-8.5	20.693
3	-8.5	17.348
4	-8.3	27.892
5	-8.3	1.504
6	-8.2	4.042
7	-8.1	3.448
8	-8	21.13
9	-8	25.074
10	-8	21.405
11	-7.9	24.65
12	-7.9	19.37
13	-7.8	28.246
14	-7.7	19.288
15	-7.6	23.782
16	-7.4	22.866
17	-7.4	4.48
18	-7.3	28.042
19	-7.2	22.611
20	-7.1	28.406

MAPK8-kaempferol

mode	affinity(kcal/mol)	dist from best mode（rmsd l.b.）
1	-8.7	0
2	-8.5	39.353
3	-8.4	36.843
4	-8.3	51.938
5	-8.3	59.526
6	-8.1	59.906
7	-8	60.521
8	-8	65.102
9	-7.9	20.735
10	-7.8	60.368
11	-7.8	63.855
12	-7.8	21.387
13	-7.6	65.348
14	-7.6	32.16
15	-7.5	60.262
16	-7.5	65.551
17	-7.4	53.465
18	-7.4	51.454
19	-7.4	21.232
20	-7.3	21.193

TP53-acacetin

mode	affinity(kcal/mol)	dist from best mode（rmsd l.b.）

1	-8.6	0
2	-8.3	3.093
3	-8.2	33.399
4	-8.2	59.862
5	-8.2	60.809
6	-8	51.92
7	-7.9	3.976
8	-7.7	99.405
9	-7.6	2.01
10	-7.5	17.693
11	-7.5	100.592
12	-7.3	64.589
13	-7.3	48.659
14	-7.3	54.169
15	-7.2	18.23
16	-7.2	95.197
17	-7.1	60.215
18	-7	41.456
19	-6.9	2.152
20	-6.9	22.353

TP53-baicalein

mode	affinity(kcal/mol)	dist from best mode（rmsd l.b.）
1	-8.5	0
2	-8.5	70.024
3	-8.4	33.178
4	-8.2	23.665
5	-8.2	74.457
6	-8.1	52.113
7	-8	70.282
8	-7.9	70.553
9	-7.9	51.947
10	-7.9	74.52
11	-7.9	75.991
12	-7.8	74.032
13	-7.7	55.672
14	-7.7	23.307
15	-7.6	59.773
16	-7.5	57.265
17	-7.5	50.658
18	-7.4	49.578
19	-7.3	31.57
20	-7.3	30.853

TP53-quercetin

mode	affinity(kcal/mol)	dist from best mode（rmsd l.b.）
1	-8.9	0
2	-8.5	74.875
3	-8.4	113.018
4	-8.3	3.173
5	-8	26.093
6	-8	25.546
7	-8	75.845
8	-8	110.861
9	-8	25.102
10	-8	51.699
11	-7.9	50.184
12	-7.9	113.803

13	-7.9	3.773
14	-7.8	108.292
15	-7.5	103.603
16	-7.4	50.964
17	-7.3	24.537
18	-7.3	22.349
19	-7.2	103.073
20	-7.1	104.204

TP53-wogonin

mode	affinity(kcal/mol)	dist from best mode（rmsd l.b.）	
1	-8.8	0.000	0.000
2	-8.4	33.622	36.969
3	-7.8	29.434	32.483
4	-7.7	21.954	25.289
5	-7.6	32.867	34.065
6	-7.6	22.487	25.179
7	-7.4	1.594	1.889
8	-7.3	81.258	84.607
9	-7.3	22.939	25.711
10	-7.3	80.945	84.244
11	-7.2	32.048	35.004
12	-7.2	24.870	26.223
13	-7.2	6.008	9.455
14	-7.2	24.798	25.808
15	-7.2	72.797	75.730
16	-7.1	31.872	34.353
17	-7.0	7.893	11.267
18	-6.8	22.496	24.781
19	-6.6	12.148	14.275
20	-6.5	32.303	34.805

dist from best mode（rmsd u.b.）
0
2.622
4.553
4.452
4.198
10.768
25.493
10.897
2.815
3.304
30.614
20.326
27.221
23.24
25.071
22.629
26.303
31.031
11.833
10.833

dist from best mode（rmsd u.b.）
0
6.529
3.297
6.439
6.788
6.886
6.471
27.955
18.97
24.574
24.87
26.382
6.881
27.522
25.834
19.107
28.196
24.373
26.184
26.834

dist from best mode（rmsd u.b.）
0
2.983
6.903
6.646
12.783
18.767
2.982
28.658
24.454
24.732

6.576
26.465
23.553
28.42
20.024
23.151
26.354
25.841
20.525
26.861

dist from best mode （rmsd u.b.）
0
4.86
3.393
4.759
11.328
27.227
23.063
19.869
30.672
30.378
22.745
26.66
25.443
29.684
20.523
26.824
21.163
25.924
27.023
19.977

dist from best mode （rmsd u.b.）
0
4.495
17.334
17.629
16.81
6.707
15.26
17.562
16.578
10.05
18.163
15.494
16.077
19.151
13.358
16.937
16.849
39.051
14.256
8.158

dist from best mode（rmsd u.b.）
0
16.578
25.729
26.03
14.941
25.542
11.072
29.316
27.163
11.934
21.761
31.938
16.033
28.295
24.755
10.788
3.24
6.335
27.949
17.781

dist from best mode（rmsd u.b.）
0
25.601
27.441
14.954
26.046
28.031
10.95
2.001
9.449
25.597
31.743
27.74
34.243
6.699
3.691
9.43
4.724
7.032
6.435
15.432

dist from best mode（rmsd u.b.）
0
16.543
15.854
31.034
34.681
15.786
16.118
15.386
35.157
16.291
36.212

16.62
32.68
16.552
16.087
12.816
14.769
16.162
16.913
13.191

dist from best mode （rmsd u.b.）
0
21.618
22.12
29.259
6.483
6.98
6.447
22.199
26.323
22.46
25.449
23.955
30.045
24.409
24.842
23.878
6.913
29.88
23.572
29.444

dist from best mode （rmsd u.b.）
0
41.583
38.936
54.608
61.524
61.782
62.517
66.364
22.697
62.187
65.951
22.323
66.997
33.772
62.259
67.564
54.961
54.524
22.97
22.155

dist from best mode （rmsd u.b.）

0
4.257
37.293
61.431
62.846
53.394
6.03
101.278
2.539
19.782
102.862
66.348
50.506
56.122
21.218
96.06
61.644
43.484
8.854
23.107

dist from best mode（ rmsd u.b.)
0
71.809
35.04
25.776
75.845
53.344
70.568
71.618
53.465
76.635
78.142
76.562
57.681
25.54
61.797
59.194
52.757
51.602
33.69
33.821

dist from best mode（ rmsd u.b.)
0
76.793
116.243
6.417
27.443
27.171
76.544
113.5
26.691
53.228
52.061
117.842

6.412
111.952
107.706
52.781
27.216
26.224
106.84
107.765

dist from best mode（rmsd u.b.）
