# Supplementary material for: Identification of Bioactive Compounds and Potential Mechanisms of Kuntai Capsule in the Treatment of Polycystic Ovary Syndrome by Integrating Network Pharmacology and Bioinformatics
Source: Oxid Med Cell Longev. 2022 Apr 28;2022:3145938. doi: 10.1155/2022/3145938 (PMC9073551; doi:10.1155/2022/3145938)
Supplement: Supplementary 7 — Supplement Table 7: potential therapeutic targets of the active ingredients of KTC. [file 3145938.f7.pdf]

Number	Active ingredients	Number of intersections with 88 potential therapeutic targets	Intersection with 88 potential therapeutic targets
1	(+)-catechin	5	ESR1，PTGS2，HSP90AA1，NPPB，HAS2
2	(2R)-2-[(3S,5R,10S,13R,14R,16R,17R)-3,16-dihydroxy-4,4,10,13,14-pentamethyl-2,3,5,6,12,15,16,17-octahydro-1H-cyclopenta[a]phenanthren-17-yl]-6-methylhept-5-enoic acid	1	NR3C2
3	(2R)-7-hydroxy-5-methoxy-2-phenylchroman-4-one	4	ESR1，PTGS2，ADRB2，HSP90AA1
4	(3S,5R,8R,9R,10S,14S)-3,17-dihydroxy-4,4,8,10,14-pentamethyl-2,3,5,6,7,9-hexahydro-1H-cyclopenta[a]phenanthrene-15,16-dione	2	PGR，NR3C2
5	(R)-Canadine	4	PTGS2，ADRB2，HSP90AA1，SLC6A2
6	11,13-Eicosadienoic acid, methyl ester	0	
7	5,2',6'-Trihydroxy-7,8-dimethoxyflavone	8	NOS2，AR，PTGS2，TOP2A，ESR2，DPP4，HSP90AA1，CDK2
8	5,2'-Dihydroxy-6,7,8-trimethoxyflavone	10	NOS2，F2，AR，PTGS2，TOP2A，ESR2，DPP4，PPARD，HSP90AA1，KDR
9	5,7,2,5-tetrahydroxy-8,6-dimethoxyflavone	7	NOS2，F2，AR，PTGS2，TOP2A，DPP4，HSP90AA1
10	5,7,2',6'-Tetrahydroxyflavone	4	AR，PTGS2，DPP4，HSP90AA1
11	5,7,4'-trihydroxy-6-methoxyflavanone	2	PTGS2，HSP90AA1

12	5,7,4'-trihydroxy-8-methoxyflavanone	2	PTGS2，HSP90AA1
13	5,7,4'-Trihydroxy-8-methoxyflavone	9	NOS2，ESR1，AR，PPARG，PTGS2，DPP4，GSK3B，HSP90AA1，CDK2
14	acacetin	14	NOS2，AR，PTGS2，DPP4，HSP90AA1，CDK2，ADRB2，BCL2，CDKN1A，BAX，CASP3，TP53，CASP8，CYP19A1
15	baicalein	15	AR，PTGS2，HSP90AA1，DPP4，AKT1，NPPB，BCL2，FOS，BAX，MMP9，CASP3，TP53，HIF1A，FOSL2，IGF2
16	berberine	6	NOS2，ESR1，AR，PTGS2，ADRB2，HSP90AA1
17	berberrubine	4	NOS2，ESR1，AR，PTGS2
18	Berlambine	5	NOS2，AR，PTGS2，ADRB2，HSP90AA1
19	beta-sitosterol	11	PGR，PTGS2，HSP90AA1，ADRB2，BCL2，BAX，CASP9，JUN，CASP3，CASP8，PON1
20	bis[(2S)-2-ethylhexyl] benzene-1,2-dicarboxylate	0	
21	Carthamidin	2	PTGS2，HSP90AA1
22	Cerevisterol	1	NR3C2
23	coptisine	4	NOS2，ESR1，AR，PTGS2
24	Corchoroside A_qt	1	NR3C2
25	Dihydrobaicalin_qt	2	PTGS2，HSP90AA1
26	DIHYDROOROXYLIN	3	PTGS2，ADRB2，HSP90AA1
27	Diop	1	ADRB2
28	ent-Epicatechin	3	ESR1，PTGS2，

			HSP90AA1
29	epiberberine	4	NOS2, ESR1, AR, PTGS2
30	ergosta-7,22E-dien-3beta-ol	1	PGR
31	Ergosterol peroxide	1	PGR
32	Eriodyctiol (flavanone)	2	PTGS2, HSP90AA1
33	hederagenin	4	PGR, NPPB, PTGS2, SLC6A2
34	kaempferol	28	NOS2, AR, PPARG, PTGS2, HSP90AA1, DPP4, PGR, F2, SLC6A2, TOP2A, AKT1, BCL2, BAX, JUN, CASP3, MAPK8, MMP1, HMOX1, NPPB, CYP1A1, ICAM1, VCAM1, CYP1B1, HAS2, SLC2A4, GSTM1, AKR1C3, SLPI
35	Magnograndiolide	0	
36	Mairin	1	PGR
37	Moslosooflavone	11	NOS2, F2, AR, PPARG, PTGS2, ESR2, DPP4, GSK3B, HSP90AA1, CDK2, ADRB2
38	NEOBAICALEIN	11	NOS2, F2, ESR1, AR, PPARG, PTGS2, TOP2A, ESR2, DPP4, GSK3B, HSP90AA1
39	Norwogonin	7	NOS2, AR, PPARG, PTGS2, DPP4, HSP90AA1, CDK2
40	oroxylin a	10	NOS2, AR, PTGS2, ADRB2, DPP4, HSP90AA1, BCL2, IL6R, CASP3, NPPB
41	paeoniflorin	3	IL6R, CD14, LBP
42	palmatine	8	NOS2, ESR1, AR, PTGS2, ADRB2, ESR2, HSP90AA1,

			CDK2
43	Panicolin	7	NOS2, AR, PTGS2, ESR2 , DPP4 , HSP90AA1, CDK2
44	quercetin	71	AR , PTGS2 , HSP90AA1, DPP4, AKR1B1 , F2 , ADRB2 , EGFR , AKT1 , NPPB , BCL2 , BCL2L1 , FOS , CDKN1A , BAX , CASP9 , MMP2 , MMP9 , MAPK1, RB1, JUN, IL6R, CASP3, TP53, ODC1 , CASP8 , SOD1 , MMP1 , HIF1A , HSPA5 , PPARG, ACACA, HMOX1, CAV1, F3, CYP1A1, ICAM1, IL1B , CCL2 , VCAM1, CXCL8, BIRC5 , NOS3 , HSPB1, CYP1B1, PLAT , THBD , SERPINE1, IFNG, IL1A , TOP2A , NCF1 , HAS2 , NQO1 , SLC2A4 , COL3A1, CLDN4, PPARA , PPARD , CXCL10 , SPP1 , RUNX2 , E2F1 , IGFBP3 , IGF2 , CD40LG , IRF1 , ERBB3 , PON1 , RASA1, GSTM1
45	rivularin	9	NOS2 , F2 , AR , PTGS2 , KDR , TOP2A , ESR2 , DPP4, HSP90AA1
46	Salvigenin	6	NOS2, F2, PTGS2, ADRB2 , DPP4 ,

			HSP90AA1
47	sitosterol	2	PGR，NR3C2
48	Skullcapflavone II	8	NOS2，F2，AR，PTGS2，KDR，TOP2A，DPP4，HSP90AA1
49	Stigmasterol	7	PGR，NR3C2，NPPB，PTGS2，SLC6A2，ADRB2，AKR1B1
50	trametenolic acid	1	NR3C2
51	wogonin	24	NOS2，ESR1，AR，PPARG，PTGS2，DPP4，GSK3B，HSP90AA1，CDK2，ADRB2，AKT1，BCL2，CDKN1A，BAX，CASP9，KDR，JUN，IL6R，CASP3，TP53，MMP1，CCL2，FN1，CXCL8
52	Worenine	4	NOS2，ESR1，AR，PTGS2
