# Supplementary material for: Identification of Bioactive Compounds and Potential Mechanisms of Kuntai Capsule in the Treatment of Polycystic Ovary Syndrome by Integrating Network Pharmacology and Bioinformatics
Source: Oxid Med Cell Longev. 2022 Apr 28;2022:3145938. doi: 10.1155/2022/3145938 (PMC9073551; doi:10.1155/2022/3145938)
Supplement: Supplementary 8 — Supplement Table 8: analysis of PPI network, GO, and KEGG of quercetin's potential therapeutic targets. [file 3145938.f8.pdf]

PPI network

node1	node2	node1_string	node2_string_id
ADRB2	IL1B	9606.ENSP00(	9606.ENSP00000263341
ADRB2	EGFR	9606.ENSP00(	9606.ENSP00000275493
ADRB2	F2	9606.ENSP00(	9606.ENSP00000308541
AKT1	MAPK1	9606.ENSP00(	9606.ENSP00000215832
AKT1	HMOX1	9606.ENSP00(	9606.ENSP00000216117
AKT1	MMP2	9606.ENSP00(	9606.ENSP00000219070
AKT1	SERPINE1	9606.ENSP00(	9606.ENSP00000223095
AKT1	CCL2	9606.ENSP00(	9606.ENSP00000225831
AKT1	HSPB1	9606.ENSP00(	9606.ENSP00000248553
AKT1	ICAM1	9606.ENSP00(	9606.ENSP00000264832
AKT1	ERBB3	9606.ENSP00(	9606.ENSP00000267101
AKT1	RB1	9606.ENSP00(	9606.ENSP00000267163
AKT1	TP53	9606.ENSP00(	9606.ENSP00000269305
AKT1	SOD1	9606.ENSP00(	9606.ENSP00000270142
AKT1	NCF1	9606.ENSP00(	9606.ENSP00000289473
AKT1	BAX	9606.ENSP00(	9606.ENSP00000293288
AKT1	VCAM1	9606.ENSP00(	9606.ENSP00000294728
AKT1	NOS3	9606.ENSP00(	9606.ENSP00000297494
AKT1	BCL2L1	9606.ENSP00(	9606.ENSP00000302564
AKT1	FOS	9606.ENSP00(	9606.ENSP00000306245
AKT1	CXCL8	9606.ENSP00(	9606.ENSP00000306512
AKT1	CASP3	9606.ENSP00(	9606.ENSP00000311032
AKT1	SLC2A4	9606.ENSP00(	9606.ENSP00000320935
AKT1	HSPA5	9606.ENSP00(	9606.ENSP00000324173
AKT1	CASP9	9606.ENSP00(	9606.ENSP00000330237
AKT1	HSP90AA1	9606.ENSP00(	9606.ENSP00000335153
AKT1	CAV1	9606.ENSP00(	9606.ENSP00000339191
AKT1	E2F1	9606.ENSP00(	9606.ENSP00000345571
AKT1	CASP8	9606.ENSP00(	9606.ENSP00000351273
AKT1	PTGS2	9606.ENSP00(	9606.ENSP00000356438
AKT1	CD40LG	9606.ENSP00(	9606.ENSP00000359663
AKT1	JUN	9606.ENSP00(	9606.ENSP00000360266
AKT1	RUNX2	9606.ENSP00(	9606.ENSP00000360493
AKT1	MMP9	9606.ENSP00(	9606.ENSP00000361405
AKT1	AR	9606.ENSP00(	9606.ENSP00000363822
AKT1	IGFBP3	9606.ENSP00(	9606.ENSP00000370473
AKT1	SPP1	9606.ENSP00(	9606.ENSP00000378517
AKT1	BCL2	9606.ENSP00(	9606.ENSP00000381185
AKT1	CDKN1A	9606.ENSP00(	9606.ENSP00000384849
AKT1	IGF2	9606.ENSP00(	9606.ENSP00000391826
AKT1	HIF1A	9606.ENSP00(	9606.ENSP00000437955
AR	MAPK1	9606.ENSP00(	9606.ENSP00000215832
AR	ERBB3	9606.ENSP00(	9606.ENSP00000267101
AR	RB1	9606.ENSP00(	9606.ENSP00000267163
AR	TP53	9606.ENSP00(	9606.ENSP00000269305
AR	EGFR	9606.ENSP00(	9606.ENSP00000275493
AR	BCL2L1	9606.ENSP00(	9606.ENSP00000302564
AR	CASP3	9606.ENSP00(	9606.ENSP00000311032
AR	HSP90AA1	9606.ENSP00(	9606.ENSP00000335153
AR	CAV1	9606.ENSP00(	9606.ENSP00000339191
AR	CASP8	9606.ENSP00(	9606.ENSP00000351273
AR	JUN	9606.ENSP00(	9606.ENSP00000360266
AR	RUNX2	9606.ENSP00(	9606.ENSP00000360493
AR	MMP9	9606.ENSP00(	9606.ENSP00000361405
BAX	TP53	9606.ENSP00(	9606.ENSP00000269305
BAX	CASP8	9606.ENSP00(	9606.ENSP00000351273
BAX	CASP9	9606.ENSP00(	9606.ENSP00000330237
BAX	CASP3	9606.ENSP00(	9606.ENSP00000311032
BAX	BCL2	9606.ENSP00(	9606.ENSP00000381185
BAX	BCL2L1	9606.ENSP00(	9606.ENSP00000302564
BCL2	MAPK1	9606.ENSP00(	9606.ENSP00000215832

BCL2	TP53	9606.ENSP00(	9606.ENSP00000269305
BCL2	SOD1	9606.ENSP00(	9606.ENSP00000270142
BCL2	BCL2L1	9606.ENSP00(	9606.ENSP00000302564
BCL2	CASP3	9606.ENSP00(	9606.ENSP00000311032
BCL2	CASP8	9606.ENSP00(	9606.ENSP00000351273
BCL2L1	MAPK1	9606.ENSP00(	9606.ENSP00000215832
BCL2L1	TP53	9606.ENSP00(	9606.ENSP00000269305
BCL2L1	CXCL8	9606.ENSP00(	9606.ENSP00000306512
BCL2L1	CASP3	9606.ENSP00(	9606.ENSP00000311032
BCL2L1	CASP9	9606.ENSP00(	9606.ENSP00000330237
BCL2L1	CASP8	9606.ENSP00(	9606.ENSP00000351273
BIRC5	TP53	9606.ENSP00(	9606.ENSP00000269305
BIRC5	RASA1	9606.ENSP00(	9606.ENSP00000274376
BIRC5	CASP3	9606.ENSP00(	9606.ENSP00000311032
BIRC5	CASP9	9606.ENSP00(	9606.ENSP00000330237
BIRC5	TOP2A	9606.ENSP00(	9606.ENSP00000411532
CASP3	MAPK1	9606.ENSP00(	9606.ENSP00000215832
CASP3	HMOX1	9606.ENSP00(	9606.ENSP00000216117
CASP3	HSPB1	9606.ENSP00(	9606.ENSP00000248553
CASP3	IL1B	9606.ENSP00(	9606.ENSP00000263341
CASP3	RB1	9606.ENSP00(	9606.ENSP00000267163
CASP3	TP53	9606.ENSP00(	9606.ENSP00000269305
CASP3	EGFR	9606.ENSP00(	9606.ENSP00000275493
CASP3	NOS3	9606.ENSP00(	9606.ENSP00000297494
CASP3	MMP9	9606.ENSP00(	9606.ENSP00000361405
CASP3	HSPA5	9606.ENSP00(	9606.ENSP00000324173
CASP3	JUN	9606.ENSP00(	9606.ENSP00000360266
CASP3	CASP9	9606.ENSP00(	9606.ENSP00000330237
CASP3	CDKN1A	9606.ENSP00(	9606.ENSP00000384849
CASP3	CASP8	9606.ENSP00(	9606.ENSP00000351273
CASP8	MAPK1	9606.ENSP00(	9606.ENSP00000215832
CASP8	IL1B	9606.ENSP00(	9606.ENSP00000263341
CASP8	TP53	9606.ENSP00(	9606.ENSP00000269305
CASP8	EGFR	9606.ENSP00(	9606.ENSP00000275493
CASP8	COL3A1	9606.ENSP00(	9606.ENSP00000304408
CASP8	HSPA5	9606.ENSP00(	9606.ENSP00000324173
CASP8	CASP9	9606.ENSP00(	9606.ENSP00000330237
CASP8	HSP90AA1	9606.ENSP00(	9606.ENSP00000335153
CASP9	MAPK1	9606.ENSP00(	9606.ENSP00000215832
CASP9	TP53	9606.ENSP00(	9606.ENSP00000269305
CASP9	JUN	9606.ENSP00(	9606.ENSP00000360266
CAV1	TP53	9606.ENSP00(	9606.ENSP00000269305
CAV1	EGFR	9606.ENSP00(	9606.ENSP00000275493
CAV1	PPARG	9606.ENSP00(	9606.ENSP00000287820
CAV1	NOS3	9606.ENSP00(	9606.ENSP00000297494
CAV1	HSP90AA1	9606.ENSP00(	9606.ENSP00000335153
CAV1	PTGS2	9606.ENSP00(	9606.ENSP00000356438
CCL2	MAPK1	9606.ENSP00(	9606.ENSP00000215832
CCL2	HMOX1	9606.ENSP00(	9606.ENSP00000216117
CCL2	MMP2	9606.ENSP00(	9606.ENSP00000219070
CCL2	SERPINE1	9606.ENSP00(	9606.ENSP00000223095
CCL2	IL1A	9606.ENSP00(	9606.ENSP00000263339
CCL2	IL6R	9606.ENSP00(	9606.ENSP00000357470
CCL2	TP53	9606.ENSP00(	9606.ENSP00000269305
CCL2	F3	9606.ENSP00(	9606.ENSP00000334145
CCL2	CD40LG	9606.ENSP00(	9606.ENSP00000359663
CCL2	IFNG	9606.ENSP00(	9606.ENSP00000229135
CCL2	SPP1	9606.ENSP00(	9606.ENSP00000378517
CCL2	MMP9	9606.ENSP00(	9606.ENSP00000361405
CCL2	PTGS2	9606.ENSP00(	9606.ENSP00000356438
CCL2	MMP1	9606.ENSP00(	9606.ENSP00000322788
CCL2	ICAM1	9606.ENSP00(	9606.ENSP00000264832
CCL2	VCAM1	9606.ENSP00(	9606.ENSP00000294728

CCL2	CXCL10	9606.ENSP00(	9606.ENSP00000305651
CCL2	IL1B	9606.ENSP00(	9606.ENSP00000263341
CCL2	FOS	9606.ENSP00(	9606.ENSP00000306245
CCL2	JUN	9606.ENSP00(	9606.ENSP00000360266
CCL2	CXCL8	9606.ENSP00(	9606.ENSP00000306512
CD40LG	IFNG	9606.ENSP00(	9606.ENSP00000229135
CD40LG	ICAM1	9606.ENSP00(	9606.ENSP00000264832
CD40LG	VCAM1	9606.ENSP00(	9606.ENSP00000294728
CDKN1A	RB1	9606.ENSP00(	9606.ENSP00000267163
CDKN1A	TP53	9606.ENSP00(	9606.ENSP00000269305
CDKN1A	HSP90AA1	9606.ENSP00(	9606.ENSP00000335153
CDKN1A	E2F1	9606.ENSP00(	9606.ENSP00000345571
CDKN1A	JUN	9606.ENSP00(	9606.ENSP00000360266
CDKN1A	HIF1A	9606.ENSP00(	9606.ENSP00000437955
COL3A1	MMP2	9606.ENSP00(	9606.ENSP00000219070
CXCL10	IFNG	9606.ENSP00(	9606.ENSP00000229135
CXCL10	IRF1	9606.ENSP00(	9606.ENSP00000245414
CXCL10	IL1B	9606.ENSP00(	9606.ENSP00000263341
CXCL10	ICAM1	9606.ENSP00(	9606.ENSP00000264832
CXCL10	MMP9	9606.ENSP00(	9606.ENSP00000361405
CXCL10	CXCL8	9606.ENSP00(	9606.ENSP00000306512
CXCL8	MAPK1	9606.ENSP00(	9606.ENSP00000215832
CXCL8	HMOX1	9606.ENSP00(	9606.ENSP00000216117
CXCL8	MMP2	9606.ENSP00(	9606.ENSP00000219070
CXCL8	SERPINE1	9606.ENSP00(	9606.ENSP00000223095
CXCL8	IFNG	9606.ENSP00(	9606.ENSP00000229135
CXCL8	IL1A	9606.ENSP00(	9606.ENSP00000263339
CXCL8	IL1B	9606.ENSP00(	9606.ENSP00000263341
CXCL8	ICAM1	9606.ENSP00(	9606.ENSP00000264832
CXCL8	EGFR	9606.ENSP00(	9606.ENSP00000275493
CXCL8	VCAM1	9606.ENSP00(	9606.ENSP00000294728
CXCL8	FOS	9606.ENSP00(	9606.ENSP00000306245
CXCL8	MMP1	9606.ENSP00(	9606.ENSP00000322788
CXCL8	MMP9	9606.ENSP00(	9606.ENSP00000361405
CXCL8	PTGS2	9606.ENSP00(	9606.ENSP00000356438
CXCL8	JUN	9606.ENSP00(	9606.ENSP00000360266
CYP1A1	TP53	9606.ENSP00(	9606.ENSP00000269305
CYP1A1	GSTM1	9606.ENSP00(	9606.ENSP00000311469
CYP1A1	NQO1	9606.ENSP00(	9606.ENSP00000319788
CYP1A1	CYP1B1	9606.ENSP00(	9606.ENSP00000478561
CYP1A1	PPARA	9606.ENSP00(	9606.ENSP00000385523
CYP1B1	GSTM1	9606.ENSP00(	9606.ENSP00000311469
CYP1B1	NQO1	9606.ENSP00(	9606.ENSP00000319788
E2F1	RB1	9606.ENSP00(	9606.ENSP00000267163
E2F1	TP53	9606.ENSP00(	9606.ENSP00000269305
E2F1	PPARG	9606.ENSP00(	9606.ENSP00000287820
EGFR	MAPK1	9606.ENSP00(	9606.ENSP00000215832
EGFR	SERPINE1	9606.ENSP00(	9606.ENSP00000223095
EGFR	IFNG	9606.ENSP00(	9606.ENSP00000229135
EGFR	HSPB1	9606.ENSP00(	9606.ENSP00000248553
EGFR	ERBB3	9606.ENSP00(	9606.ENSP00000267101
EGFR	TP53	9606.ENSP00(	9606.ENSP00000269305
EGFR	RASA1	9606.ENSP00(	9606.ENSP00000274376
EGFR	PPARG	9606.ENSP00(	9606.ENSP00000287820
EGFR	MMP9	9606.ENSP00(	9606.ENSP00000361405
EGFR	IGF2	9606.ENSP00(	9606.ENSP00000391826
EGFR	IGFBP3	9606.ENSP00(	9606.ENSP00000370473
EGFR	PTGS2	9606.ENSP00(	9606.ENSP00000356438
EGFR	JUN	9606.ENSP00(	9606.ENSP00000360266
EGFR	FOS	9606.ENSP00(	9606.ENSP00000306245
EGFR	HIF1A	9606.ENSP00(	9606.ENSP00000437955
EGFR	HSP90AA1	9606.ENSP00(	9606.ENSP00000335153
ERBB3	IGF2	9606.ENSP00(	9606.ENSP00000391826

ERBB3	HSP90AA1	9606.ENSP00(	9606.ENSP00000335153
F2	MAPK1	9606.ENSP00(	9606.ENSP00000215832
F2	PLAT	9606.ENSP00(	9606.ENSP00000220809
F2	SERPINE1	9606.ENSP00(	9606.ENSP00000223095
F2	SPP1	9606.ENSP00(	9606.ENSP00000378517
F2	PPARA	9606.ENSP00(	9606.ENSP00000385523
F2	IGF2	9606.ENSP00(	9606.ENSP00000391826
F2	IGFBP3	9606.ENSP00(	9606.ENSP00000370473
F2	F3	9606.ENSP00(	9606.ENSP00000334145
F2	THBD	9606.ENSP00(	9606.ENSP00000366307
F3	PLAT	9606.ENSP00(	9606.ENSP00000220809
F3	SERPINE1	9606.ENSP00(	9606.ENSP00000223095
F3	THBD	9606.ENSP00(	9606.ENSP00000366307
FOS	MAPK1	9606.ENSP00(	9606.ENSP00000215832
FOS	HMOX1	9606.ENSP00(	9606.ENSP00000216117
FOS	IL1A	9606.ENSP00(	9606.ENSP00000263339
FOS	IL1B	9606.ENSP00(	9606.ENSP00000263341
FOS	TP53	9606.ENSP00(	9606.ENSP00000269305
FOS	PPARG	9606.ENSP00(	9606.ENSP00000287820
FOS	PTGS2	9606.ENSP00(	9606.ENSP00000356438
FOS	MMP1	9606.ENSP00(	9606.ENSP00000322788
FOS	JUN	9606.ENSP00(	9606.ENSP00000360266
GSTM1	NQO1	9606.ENSP00(	9606.ENSP00000319788
HIF1A	MAPK1	9606.ENSP00(	9606.ENSP00000215832
HIF1A	HMOX1	9606.ENSP00(	9606.ENSP00000216117
HIF1A	MMP2	9606.ENSP00(	9606.ENSP00000219070
HIF1A	TP53	9606.ENSP00(	9606.ENSP00000269305
HIF1A	NOS3	9606.ENSP00(	9606.ENSP00000297494
HIF1A	HSP90AA1	9606.ENSP00(	9606.ENSP00000335153
HIF1A	JUN	9606.ENSP00(	9606.ENSP00000360266
HMOX1	MAPK1	9606.ENSP00(	9606.ENSP00000215832
HMOX1	SOD1	9606.ENSP00(	9606.ENSP00000270142
HMOX1	PPARG	9606.ENSP00(	9606.ENSP00000287820
HMOX1	MMP9	9606.ENSP00(	9606.ENSP00000361405
HMOX1	TP53	9606.ENSP00(	9606.ENSP00000269305
HMOX1	IL1B	9606.ENSP00(	9606.ENSP00000263341
HMOX1	PTGS2	9606.ENSP00(	9606.ENSP00000356438
HMOX1	NOS3	9606.ENSP00(	9606.ENSP00000297494
HMOX1	NQO1	9606.ENSP00(	9606.ENSP00000319788
HMOX1	JUN	9606.ENSP00(	9606.ENSP00000360266
HSP90AA1	MAPK1	9606.ENSP00(	9606.ENSP00000215832
HSP90AA1	MMP2	9606.ENSP00(	9606.ENSP00000219070
HSP90AA1	IFNG	9606.ENSP00(	9606.ENSP00000229135
HSP90AA1	IRF1	9606.ENSP00(	9606.ENSP00000245414
HSP90AA1	HSPB1	9606.ENSP00(	9606.ENSP00000248553
HSP90AA1	RB1	9606.ENSP00(	9606.ENSP00000267163
HSP90AA1	TP53	9606.ENSP00(	9606.ENSP00000269305
HSP90AA1	SOD1	9606.ENSP00(	9606.ENSP00000270142
HSP90AA1	NOS3	9606.ENSP00(	9606.ENSP00000297494
HSP90AA1	HSPA5	9606.ENSP00(	9606.ENSP00000324173
HSP90AA1	PPARA	9606.ENSP00(	9606.ENSP00000385523
HSPA5	HSPB1	9606.ENSP00(	9606.ENSP00000248553
HSPB1	MAPK1	9606.ENSP00(	9606.ENSP00000215832
HSPB1	TP53	9606.ENSP00(	9606.ENSP00000269305
ICAM1	MAPK1	9606.ENSP00(	9606.ENSP00000215832
ICAM1	MMP2	9606.ENSP00(	9606.ENSP00000219070
ICAM1	IFNG	9606.ENSP00(	9606.ENSP00000229135
ICAM1	IRF1	9606.ENSP00(	9606.ENSP00000245414
ICAM1	IL1A	9606.ENSP00(	9606.ENSP00000263339
ICAM1	IL1B	9606.ENSP00(	9606.ENSP00000263341
ICAM1	NOS3	9606.ENSP00(	9606.ENSP00000297494
ICAM1	PTGS2	9606.ENSP00(	9606.ENSP00000356438
ICAM1	PPARG	9606.ENSP00(	9606.ENSP00000287820

ICAM1	SPP1	9606.ENSP00(	9606.ENSP00000378517
ICAM1	TP53	9606.ENSP00(	9606.ENSP00000269305
ICAM1	MMP9	9606.ENSP00(	9606.ENSP00000361405
ICAM1	JUN	9606.ENSP00(	9606.ENSP00000360266
ICAM1	VCAM1	9606.ENSP00(	9606.ENSP00000294728
IFNG	IL1A	9606.ENSP00(	9606.ENSP00000263339
IFNG	MMP9	9606.ENSP00(	9606.ENSP00000361405
IFNG	IL6R	9606.ENSP00(	9606.ENSP00000357470
IFNG	IL1B	9606.ENSP00(	9606.ENSP00000263341
IFNG	JUN	9606.ENSP00(	9606.ENSP00000360266
IFNG	IRF1	9606.ENSP00(	9606.ENSP00000245414
IGF2	MMP2	9606.ENSP00(	9606.ENSP00000219070
IGF2	SERPINE1	9606.ENSP00(	9606.ENSP00000223095
IGF2	TP53	9606.ENSP00(	9606.ENSP00000269305
IGF2	MMP1	9606.ENSP00(	9606.ENSP00000322788
IGF2	IGFBP3	9606.ENSP00(	9606.ENSP00000370473
IGFBP3	MMP2	9606.ENSP00(	9606.ENSP00000219070
IGFBP3	TP53	9606.ENSP00(	9606.ENSP00000269305
IGFBP3	PPARG	9606.ENSP00(	9606.ENSP00000287820
IGFBP3	MMP1	9606.ENSP00(	9606.ENSP00000322788
IGFBP3	SPP1	9606.ENSP00(	9606.ENSP00000378517
IL1A	VCAM1	9606.ENSP00(	9606.ENSP00000294728
IL1A	MMP1	9606.ENSP00(	9606.ENSP00000322788
IL1A	PTGS2	9606.ENSP00(	9606.ENSP00000356438
IL1A	JUN	9606.ENSP00(	9606.ENSP00000360266
IL1A	IL1B	9606.ENSP00(	9606.ENSP00000263341
IL1B	MAPK1	9606.ENSP00(	9606.ENSP00000215832
IL1B	MMP2	9606.ENSP00(	9606.ENSP00000219070
IL1B	IRF1	9606.ENSP00(	9606.ENSP00000245414
IL1B	SPP1	9606.ENSP00(	9606.ENSP00000378517
IL1B	NOS3	9606.ENSP00(	9606.ENSP00000297494
IL1B	PPARG	9606.ENSP00(	9606.ENSP00000287820
IL1B	MMP1	9606.ENSP00(	9606.ENSP00000322788
IL1B	VCAM1	9606.ENSP00(	9606.ENSP00000294728
IL1B	MMP9	9606.ENSP00(	9606.ENSP00000361405
IL1B	JUN	9606.ENSP00(	9606.ENSP00000360266
IL1B	PTGS2	9606.ENSP00(	9606.ENSP00000356438
IL6R	MAPK1	9606.ENSP00(	9606.ENSP00000215832
IRF1	TP53	9606.ENSP00(	9606.ENSP00000269305
IRF1	JUN	9606.ENSP00(	9606.ENSP00000360266
IRF1	VCAM1	9606.ENSP00(	9606.ENSP00000294728
JUN	MAPK1	9606.ENSP00(	9606.ENSP00000215832
JUN	RB1	9606.ENSP00(	9606.ENSP00000267163
JUN	TP53	9606.ENSP00(	9606.ENSP00000269305
JUN	PPARG	9606.ENSP00(	9606.ENSP00000287820
JUN	NOS3	9606.ENSP00(	9606.ENSP00000297494
JUN	MMP1	9606.ENSP00(	9606.ENSP00000322788
JUN	PTGS2	9606.ENSP00(	9606.ENSP00000356438
JUN	MMP9	9606.ENSP00(	9606.ENSP00000361405
JUN	PPARA	9606.ENSP00(	9606.ENSP00000385523
MAPK1	NCF1	9606.ENSP00(	9606.ENSP00000289473
MAPK1	MMP1	9606.ENSP00(	9606.ENSP00000322788
MAPK1	NOS3	9606.ENSP00(	9606.ENSP00000297494
MAPK1	MMP2	9606.ENSP00(	9606.ENSP00000219070
MAPK1	PTGS2	9606.ENSP00(	9606.ENSP00000356438
MAPK1	MMP9	9606.ENSP00(	9606.ENSP00000361405
MAPK1	RASA1	9606.ENSP00(	9606.ENSP00000274376
MAPK1	PPARA	9606.ENSP00(	9606.ENSP00000385523
MAPK1	RB1	9606.ENSP00(	9606.ENSP00000267163
MAPK1	RUNX2	9606.ENSP00(	9606.ENSP00000360493
MAPK1	TP53	9606.ENSP00(	9606.ENSP00000269305
MMP1	MMP2	9606.ENSP00(	9606.ENSP00000219070
MMP1	TP53	9606.ENSP00(	9606.ENSP00000269305

MMP1	MMP9	9606.ENSP00(	9606.ENSP00000361405
MMP2	PTGS2	9606.ENSP00(	9606.ENSP00000356438
MMP2	SERPINE1	9606.ENSP00(	9606.ENSP00000223095
MMP2	SPP1	9606.ENSP00(	9606.ENSP00000378517
MMP2	TP53	9606.ENSP00(	9606.ENSP00000269305
MMP2	NCF1	9606.ENSP00(	9606.ENSP00000289473
MMP2	MMP9	9606.ENSP00(	9606.ENSP00000361405
MMP9	PLAT	9606.ENSP00(	9606.ENSP00000220809
MMP9	SERPINE1	9606.ENSP00(	9606.ENSP00000223095
MMP9	TP53	9606.ENSP00(	9606.ENSP00000269305
MMP9	PPARG	9606.ENSP00(	9606.ENSP00000287820
MMP9	NCF1	9606.ENSP00(	9606.ENSP00000289473
MMP9	NOS3	9606.ENSP00(	9606.ENSP00000297494
MMP9	PTGS2	9606.ENSP00(	9606.ENSP00000356438
MMP9	RUNX2	9606.ENSP00(	9606.ENSP00000360493
MMP9	SPP1	9606.ENSP00(	9606.ENSP00000378517
NCF1	VCAM1	9606.ENSP00(	9606.ENSP00000294728
NOS3	TP53	9606.ENSP00(	9606.ENSP00000269305
NOS3	PPARG	9606.ENSP00(	9606.ENSP00000287820
NOS3	VCAM1	9606.ENSP00(	9606.ENSP00000294728
NOS3	PTGS2	9606.ENSP00(	9606.ENSP00000356438
NQO1	ODC1	9606.ENSP00(	9606.ENSP00000234111
NQO1	TP53	9606.ENSP00(	9606.ENSP00000269305
PLAT	THBD	9606.ENSP00(	9606.ENSP00000366307
PLAT	SERPINE1	9606.ENSP00(	9606.ENSP00000223095
PPARA	RB1	9606.ENSP00(	9606.ENSP00000267163
PPARG	SERPINE1	9606.ENSP00(	9606.ENSP00000223095
PPARG	RB1	9606.ENSP00(	9606.ENSP00000267163
PPARG	TP53	9606.ENSP00(	9606.ENSP00000269305
PPARG	RUNX2	9606.ENSP00(	9606.ENSP00000360493
PPARG	PTGS2	9606.ENSP00(	9606.ENSP00000356438
PPARG	SLC2A4	9606.ENSP00(	9606.ENSP00000320935
PTGS2	TP53	9606.ENSP00(	9606.ENSP00000269305
PTGS2	VCAM1	9606.ENSP00(	9606.ENSP00000294728
RB1	RUNX2	9606.ENSP00(	9606.ENSP00000360493
RB1	TP53	9606.ENSP00(	9606.ENSP00000269305
RUNX2	TP53	9606.ENSP00(	9606.ENSP00000269305
RUNX2	SPP1	9606.ENSP00(	9606.ENSP00000378517
SERPINE1	THBD	9606.ENSP00(	9606.ENSP00000366307
SERPINE1	TP53	9606.ENSP00(	9606.ENSP00000269305
SPP1	TP53	9606.ENSP00(	9606.ENSP00000269305
TOP2A	TP53	9606.ENSP00(	9606.ENSP00000269305

GO analysis

ONTOLOGY	ID	GeneRatio	BgRatio	pvalue	p.adjust	qvalue	Count
BP	GO:0010038	22/71	364/18670	6.63E-21	2.22E-17	9.95E-18	22
BP	GO:0070997	21/71	348/18670	6.14E-20	1.03E-16	4.61E-17	21
BP	GO:0097191	18/71	224/18670	2.36E-19	2.64E-16	1.18E-16	18
BP	GO:0031667	23/71	499/18670	3.23E-19	2.71E-16	1.21E-16	23
BP	GO:0072593	19/71	284/18670	6.74E-19	4.52E-16	2.03E-16	19
BP	GO:2001233	21/71	406/18670	1.47E-18	8.22E-16	3.68E-16	21
BP	GO:1901214	19/71	313/18670	4.19E-18	2.01E-15	9.00E-16	19
BP	GO:2001234	17/71	230/18670	1.10E-17	4.20E-15	1.88E-15	17
BP	GO:0032496	19/71	330/18670	1.13E-17	4.20E-15	1.88E-15	19
BP	GO:0070482	20/71	394/18670	1.60E-17	5.37E-15	2.40E-15	20
BP	GO:0002237	19/71	343/18670	2.31E-17	7.05E-15	3.16E-15	19
BP	GO:0044706	16/71	222/18670	1.67E-16	4.65E-14	2.08E-14	16
BP	GO:0071496	18/71	339/18670	3.81E-16	9.83E-14	4.40E-14	18
BP	GO:0007565	15/71	192/18670	4.84E-16	1.13E-13	5.05E-14	15
BP	GO:0097193	17/71	289/18670	5.14E-16	1.13E-13	5.05E-14	17
BP	GO:0051402	16/71	239/18670	5.38E-16	1.13E-13	5.05E-14	16
BP	GO:2000377	15/71	195/18670	6.11E-16	1.21E-13	5.40E-14	15

BP	GO:0001666	18/71	359/18670	1.04E-15	1.89E-13	8.45E-14	18
BP	GO:0034599	17/71	302/18670	1.07E-15	1.89E-13	8.45E-14	17
BP	GO:0048608	19/71	431/18670	1.55E-15	2.61E-13	1.17E-13	19
BP	GO:0036293	18/71	370/18670	1.76E-15	2.69E-13	1.20E-13	18
BP	GO:0061458	19/71	434/18670	1.76E-15	2.69E-13	1.20E-13	19
BP	GO:0048660	14/71	169/18670	2.21E-15	3.23E-13	1.45E-13	14
BP	GO:0048659	14/71	171/18670	2.61E-15	3.65E-13	1.63E-13	14
BP	GO:0009314	19/71	448/18670	3.14E-15	4.22E-13	1.89E-13	19
BP	GO:0006979	19/71	451/18670	3.55E-15	4.58E-13	2.05E-13	19
BP	GO:0046677	17/71	327/18670	4.00E-15	4.96E-13	2.22E-13	17
BP	GO:0042110	19/71	464/18670	5.94E-15	7.12E-13	3.19E-13	19
BP	GO:0000302	15/71	232/18670	8.15E-15	9.43E-13	4.22E-13	15
BP	GO:0071276	9/71	37/18670	1.12E-14	1.26E-12	5.63E-13	9
BP	GO:0033002	15/71	239/18670	1.27E-14	1.37E-12	6.13E-13	15
BP	GO:1901654	14/71	193/18670	1.42E-14	1.49E-12	6.68E-13	14
BP	GO:0009266	15/71	243/18670	1.62E-14	1.64E-12	7.36E-13	15
BP	GO:1903409	12/71	122/18670	3.23E-14	3.18E-12	1.43E-12	12
BP	GO:0043523	14/71	210/18670	4.60E-14	4.40E-12	1.97E-12	14
BP	GO:2000379	11/71	102/18670	1.45E-13	1.35E-11	6.06E-12	11
BP	GO:2001237	11/71	104/18670	1.81E-13	1.64E-11	7.34E-12	11
BP	GO:0071248	13/71	190/18670	2.88E-13	2.52E-11	1.13E-11	13
BP	GO:0006809	10/71	77/18670	2.93E-13	2.52E-11	1.13E-11	10
BP	GO:1903037	15/71	304/18670	4.28E-13	3.59E-11	1.61E-11	15
BP	GO:0046209	10/71	82/18670	5.63E-13	4.61E-11	2.06E-11	10
BP	GO:2001236	12/71	155/18670	5.85E-13	4.67E-11	2.09E-11	12
BP	GO:2001057	10/71	85/18670	8.17E-13	6.37E-11	2.85E-11	10
BP	GO:0048545	16/71	385/18670	8.94E-13	6.81E-11	3.05E-11	16
BP	GO:0007568	15/71	321/18670	9.40E-13	7.00E-11	3.14E-11	15
BP	GO:0062012	17/71	459/18670	9.98E-13	7.27E-11	3.26E-11	17
BP	GO:0071214	15/71	331/18670	1.46E-12	1.02E-10	4.58E-11	15
BP	GO:0104004	15/71	331/18670	1.46E-12	1.02E-10	4.58E-11	15
BP	GO:0034614	12/71	168/18670	1.53E-12	1.05E-10	4.70E-11	12
BP	GO:0071241	13/71	217/18670	1.58E-12	1.06E-10	4.75E-11	13
BP	GO:0007159	15/71	337/18670	1.89E-12	1.24E-10	5.58E-11	15
BP	GO:0046686	9/71	63/18670	1.98E-12	1.28E-10	5.71E-11	9
BP	GO:0048871	17/71	485/18670	2.41E-12	1.52E-10	6.82E-11	17
BP	GO:0050673	16/71	434/18670	5.49E-12	3.41E-10	1.53E-10	16
BP	GO:0035296	11/71	143/18670	6.24E-12	3.67E-10	1.64E-10	11
BP	GO:0050880	11/71	143/18670	6.24E-12	3.67E-10	1.64E-10	11
BP	GO:0097746	11/71	143/18670	6.24E-12	3.67E-10	1.64E-10	11
BP	GO:0035150	11/71	144/18670	6.73E-12	3.87E-10	1.73E-10	11
BP	GO:0038034	9/71	72/18670	6.92E-12	3.87E-10	1.73E-10	9
BP	GO:0097192	9/71	72/18670	6.92E-12	3.87E-10	1.73E-10	9
BP	GO:0042136	10/71	106/18670	7.81E-12	4.29E-10	1.92E-10	10
BP	GO:0051098	15/71	373/18670	8.07E-12	4.37E-10	1.96E-10	15
BP	GO:0010212	11/71	147/18670	8.44E-12	4.49E-10	2.01E-10	11
BP	GO:0001890	11/71	152/18670	1.22E-11	6.38E-10	2.86E-10	11
BP	GO:1901215	12/71	208/18670	1.91E-11	9.87E-10	4.42E-10	12
BP	GO:0031668	13/71	268/18670	2.27E-11	1.16E-09	5.18E-10	13
BP	GO:0022407	15/71	403/18670	2.42E-11	1.21E-09	5.42E-10	15
BP	GO:2001242	11/71	165/18670	2.98E-11	1.47E-09	6.57E-10	11
BP	GO:1903039	12/71	218/18670	3.31E-11	1.61E-09	7.21E-10	12
BP	GO:0007584	12/71	219/18670	3.50E-11	1.67E-09	7.50E-10	12
BP	GO:0001101	14/71	343/18670	3.58E-11	1.69E-09	7.58E-10	14
BP	GO:1901342	15/71	422/18670	4.62E-11	2.15E-09	9.64E-10	15
BP	GO:0010631	14/71	351/18670	4.87E-11	2.24E-09	1.00E-09	14
BP	GO:0003018	11/71	173/18670	4.97E-11	2.25E-09	1.01E-09	11
BP	GO:0090132	14/71	354/18670	5.45E-11	2.44E-09	1.09E-09	14
BP	GO:0009408	11/71	176/18670	5.99E-11	2.64E-09	1.18E-09	11
BP	GO:1904018	12/71	230/18670	6.19E-11	2.69E-09	1.21E-09	12
BP	GO:0090130	14/71	360/18670	6.80E-11	2.93E-09	1.31E-09	14
BP	GO:0097305	12/71	233/18670	7.19E-11	3.05E-09	1.37E-09	12
BP	GO:1901655	9/71	93/18670	7.35E-11	3.08E-09	1.38E-09	9

BP	GO:0071453	12/71	234/18670	7.56E-11	3.13E-09	1.40E-09	12
BP	GO:1901216	9/71	94/18670	8.10E-11	3.31E-09	1.48E-09	9
BP	GO:0071216	12/71	236/18670	8.34E-11	3.37E-09	1.51E-09	12
BP	GO:0032768	8/71	64/18670	1.09E-10	4.35E-09	1.95E-09	8
BP	GO:0050678	14/71	378/18670	1.29E-10	5.11E-09	2.29E-09	14
BP	GO:0045765	14/71	383/18670	1.54E-10	6.00E-09	2.69E-09	14
BP	GO:0048661	9/71	101/18670	1.56E-10	6.00E-09	2.69E-09	9
BP	GO:0050863	13/71	314/18670	1.63E-10	6.21E-09	2.78E-09	13
BP	GO:0022409	12/71	255/18670	2.04E-10	7.68E-09	3.44E-09	12
BP	GO:0043524	10/71	147/18670	2.06E-10	7.68E-09	3.44E-09	10
BP	GO:0045766	11/71	204/18670	2.92E-10	1.08E-08	4.82E-09	11
BP	GO:0042133	10/71	153/18670	3.06E-10	1.10E-08	4.94E-09	10
BP	GO:0062013	10/71	153/18670	3.06E-10	1.10E-08	4.94E-09	10
BP	GO:0051249	15/71	485/18670	3.22E-10	1.15E-08	5.14E-09	15
BP	GO:0009612	11/71	210/18670	3.98E-10	1.40E-08	6.29E-09	11
BP	GO:0061614	7/71	47/18670	4.73E-10	1.65E-08	7.41E-09	7
BP	GO:0060249	14/71	437/18670	8.60E-10	2.92E-08	1.31E-08	14
BP	GO:0007566	7/71	51/18670	8.61E-10	2.92E-08	1.31E-08	7
BP	GO:0050999	7/71	51/18670	8.61E-10	2.92E-08	1.31E-08	7
BP	GO:0001894	11/71	227/18670	9.09E-10	3.02E-08	1.35E-08	11
BP	GO:0010634	10/71	171/18670	9.10E-10	3.02E-08	1.35E-08	10
BP	GO:0009410	12/71	292/18670	9.62E-10	3.16E-08	1.42E-08	12
BP	GO:0008637	9/71	124/18670	9.88E-10	3.21E-08	1.44E-08	9
BP	GO:0032102	13/71	365/18670	1.03E-09	3.32E-08	1.49E-08	13
BP	GO:0048511	12/71	295/18670	1.08E-09	3.45E-08	1.55E-08	12
BP	GO:0008625	8/71	86/18670	1.22E-09	3.86E-08	1.73E-08	8
BP	GO:0048771	10/71	179/18670	1.42E-09	4.41E-08	1.97E-08	10
BP	GO:1903034	10/71	179/18670	1.42E-09	4.41E-08	1.97E-08	10
BP	GO:0031669	11/71	237/18670	1.43E-09	4.41E-08	1.97E-08	11
BP	GO:0010332	7/71	56/18670	1.70E-09	5.18E-08	2.32E-08	7
BP	GO:0001667	14/71	461/18670	1.71E-09	5.18E-08	2.32E-08	14
BP	GO:2000045	10/71	184/18670	1.86E-09	5.53E-08	2.48E-08	10
BP	GO:0001819	14/71	464/18670	1.86E-09	5.53E-08	2.48E-08	14
BP	GO:0034349	5/71	15/18670	2.01E-09	5.91E-08	2.65E-08	5
BP	GO:0034612	12/71	312/18670	2.04E-09	5.94E-08	2.66E-08	12
BP	GO:0001836	7/71	59/18670	2.48E-09	7.16E-08	3.21E-08	7
BP	GO:0001503	13/71	398/18670	2.93E-09	8.40E-08	3.76E-08	13
BP	GO:0008585	8/71	96/18670	2.96E-09	8.42E-08	3.77E-08	8
BP	GO:0009411	9/71	141/18670	3.09E-09	8.72E-08	3.90E-08	9
BP	GO:0016049	14/71	484/18670	3.20E-09	8.94E-08	4.00E-08	14
BP	GO:0019216	13/71	410/18670	4.19E-09	1.16E-07	5.18E-08	13
BP	GO:0055123	9/71	146/18670	4.21E-09	1.16E-07	5.18E-08	9
BP	GO:0051251	12/71	334/18670	4.39E-09	1.19E-07	5.34E-08	12
BP	GO:0060135	7/71	64/18670	4.44E-09	1.19E-07	5.34E-08	7
BP	GO:0046545	8/71	101/18670	4.45E-09	1.19E-07	5.34E-08	8
BP	GO:0050870	10/71	202/18670	4.58E-09	1.21E-07	5.41E-08	10
BP	GO:1902806	10/71	202/18670	4.58E-09	1.21E-07	5.41E-08	10
BP	GO:0007596	12/71	336/18670	4.69E-09	1.22E-07	5.48E-08	12
BP	GO:0050900	14/71	499/18670	4.72E-09	1.22E-07	5.48E-08	14
BP	GO:0061041	9/71	148/18670	4.74E-09	1.22E-07	5.48E-08	9
BP	GO:0001558	13/71	416/18670	4.98E-09	1.28E-07	5.72E-08	13
BP	GO:0071222	10/71	205/18670	5.27E-09	1.34E-07	6.00E-08	10
BP	GO:0045428	7/71	66/18670	5.54E-09	1.39E-07	6.21E-08	7
BP	GO:0007599	12/71	341/18670	5.54E-09	1.39E-07	6.21E-08	12
BP	GO:0008630	8/71	104/18670	5.62E-09	1.39E-07	6.25E-08	8
BP	GO:0050817	12/71	342/18670	5.72E-09	1.41E-07	6.32E-08	12
BP	GO:0071456	10/71	207/18670	5.79E-09	1.42E-07	6.35E-08	10
BP	GO:0046651	11/71	272/18670	6.06E-09	1.47E-07	6.60E-08	11
BP	GO:0032943	11/71	274/18670	6.54E-09	1.58E-07	7.07E-08	11
BP	GO:0051341	8/71	107/18670	7.04E-09	1.69E-07	7.56E-08	8
BP	GO:1902042	6/71	39/18670	7.20E-09	1.71E-07	7.67E-08	6
BP	GO:0071219	10/71	212/18670	7.28E-09	1.72E-07	7.70E-08	10
BP	GO:0051090	13/71	432/18670	7.82E-09	1.83E-07	8.22E-08	13

BP	GO:0048732	13/71	434/18670	8.27E-09	1.92E-07	8.62E-08	13
BP	GO:0036294	10/71	217/18670	9.10E-09	2.10E-07	9.43E-08	10
BP	GO:1904019	8/71	111/18670	9.43E-09	2.17E-07	9.70E-08	8
BP	GO:1902893	6/71	41/18670	9.87E-09	2.25E-07	1.01E-07	6
BP	GO:0042063	11/71	290/18670	1.18E-08	2.67E-07	1.20E-07	11
BP	GO:0010632	11/71	291/18670	1.22E-08	2.75E-07	1.23E-07	11
BP	GO:0046660	8/71	115/18670	1.25E-08	2.79E-07	1.25E-07	8
BP	GO:0030198	12/71	368/18670	1.29E-08	2.87E-07	1.29E-07	12
BP	GO:0008631	6/71	43/18670	1.33E-08	2.90E-07	1.30E-07	6
BP	GO:0045429	6/71	43/18670	1.33E-08	2.90E-07	1.30E-07	6
BP	GO:0035690	12/71	369/18670	1.33E-08	2.90E-07	1.30E-07	12
BP	GO:0051100	9/71	169/18670	1.52E-08	3.28E-07	1.47E-07	9
BP	GO:1904407	6/71	44/18670	1.54E-08	3.30E-07	1.48E-07	6
BP	GO:0070661	11/71	298/18670	1.56E-08	3.31E-07	1.48E-07	11
BP	GO:0090068	11/71	298/18670	1.56E-08	3.31E-07	1.48E-07	11
BP	GO:0002696	12/71	380/18670	1.85E-08	3.89E-07	1.74E-07	12
BP	GO:0030193	7/71	79/18670	1.98E-08	4.15E-07	1.86E-07	7
BP	GO:1900046	7/71	80/18670	2.16E-08	4.51E-07	2.02E-07	7
BP	GO:2001235	9/71	179/18670	2.50E-08	5.18E-07	2.32E-07	9
BP	GO:0071466	9/71	180/18670	2.62E-08	5.40E-07	2.42E-07	9
BP	GO:0009416	11/71	314/18670	2.67E-08	5.46E-07	2.44E-07	11
BP	GO:0010959	12/71	394/18670	2.75E-08	5.56E-07	2.49E-07	12
BP	GO:0050867	12/71	394/18670	2.75E-08	5.56E-07	2.49E-07	12
BP	GO:0006970	7/71	83/18670	2.80E-08	5.60E-07	2.51E-07	7
BP	GO:0048145	7/71	83/18670	2.80E-08	5.60E-07	2.51E-07	7
BP	GO:0008217	9/71	182/18670	2.89E-08	5.70E-07	2.55E-07	9
BP	GO:0010821	9/71	182/18670	2.89E-08	5.70E-07	2.55E-07	9
BP	GO:0035094	6/71	49/18670	3.00E-08	5.84E-07	2.62E-07	6
BP	GO:0051205	6/71	49/18670	3.00E-08	5.84E-07	2.62E-07	6
BP	GO:0048144	7/71	84/18670	3.05E-08	5.88E-07	2.63E-07	7
BP	GO:0050818	7/71	84/18670	3.05E-08	5.88E-07	2.63E-07	7
BP	GO:0042098	9/71	184/18670	3.17E-08	6.08E-07	2.72E-07	9
BP	GO:0045926	10/71	249/18670	3.36E-08	6.41E-07	2.87E-07	10
BP	GO:0009409	6/71	50/18670	3.40E-08	6.43E-07	2.88E-07	6
BP	GO:0045785	12/71	403/18670	3.53E-08	6.65E-07	2.98E-07	12
BP	GO:0032103	11/71	323/18670	3.56E-08	6.67E-07	2.99E-07	11
BP	GO:1905475	9/71	187/18670	3.65E-08	6.80E-07	3.05E-07	9
BP	GO:1903829	11/71	324/18670	3.68E-08	6.81E-07	3.05E-07	11
BP	GO:0048565	8/71	134/18670	4.15E-08	7.65E-07	3.43E-07	8
BP	GO:0097194	7/71	88/18670	4.22E-08	7.74E-07	3.47E-07	7
BP	GO:0034605	8/71	137/18670	4.93E-08	8.96E-07	4.01E-07	8
BP	GO:1903035	7/71	90/18670	4.94E-08	8.96E-07	4.01E-07	7
BP	GO:0033135	8/71	139/18670	5.53E-08	9.96E-07	4.46E-07	8
BP	GO:0043062	12/71	422/18670	5.84E-08	1.05E-06	4.69E-07	12
BP	GO:0033273	7/71	93/18670	6.21E-08	1.11E-06	4.96E-07	7
BP	GO:0048638	11/71	347/18670	7.38E-08	1.31E-06	5.87E-07	11
BP	GO:0043525	6/71	57/18670	7.59E-08	1.33E-06	5.96E-07	6
BP	GO:1903428	6/71	57/18670	7.59E-08	1.33E-06	5.96E-07	6
BP	GO:0010575	5/71	29/18670	7.62E-08	1.33E-06	5.96E-07	5
BP	GO:0051384	8/71	146/18670	8.10E-08	1.41E-06	6.30E-07	8
BP	GO:1902041	6/71	58/18670	8.44E-08	1.46E-06	6.54E-07	6
BP	GO:2001243	7/71	98/18670	8.93E-08	1.54E-06	6.88E-07	7
BP	GO:0050670	9/71	208/18670	9.11E-08	1.55E-06	6.96E-07	9
BP	GO:0001844	5/71	30/18670	9.12E-08	1.55E-06	6.96E-07	5
BP	GO:0010623	4/71	12/18670	9.29E-08	1.57E-06	7.05E-07	4
BP	GO:0097755	6/71	59/18670	9.37E-08	1.58E-06	7.07E-07	6
BP	GO:0014074	8/71	149/18670	9.48E-08	1.58E-06	7.10E-07	8
BP	GO:0032944	9/71	209/18670	9.49E-08	1.58E-06	7.10E-07	9
BP	GO:0000082	10/71	279/18670	9.78E-08	1.62E-06	7.27E-07	10
BP	GO:1903426	7/71	101/18670	1.10E-07	1.82E-06	8.14E-07	7
BP	GO:2001244	6/71	61/18670	1.15E-07	1.89E-06	8.45E-07	6
BP	GO:0051348	10/71	285/18670	1.19E-07	1.95E-06	8.74E-07	10
BP	GO:0043281	9/71	215/18670	1.21E-07	1.97E-06	8.82E-07	9

BP	GO:0045907	5/71	32/18670	1.28E-07	2.08E-06	9.30E-07	5
BP	GO:0008406	9/71	217/18670	1.31E-07	2.11E-06	9.45E-07	9
BP	GO:1904951	12/71	456/18670	1.35E-07	2.17E-06	9.73E-07	12
BP	GO:0010001	9/71	218/18670	1.36E-07	2.17E-06	9.73E-07	9
BP	GO:0033138	7/71	105/18670	1.44E-07	2.29E-06	1.02E-06	7
BP	GO:0071356	10/71	291/18670	1.45E-07	2.29E-06	1.03E-06	10
BP	GO:0002526	9/71	220/18670	1.47E-07	2.31E-06	1.04E-06	9
BP	GO:0051204	5/71	33/18670	1.51E-07	2.36E-06	1.06E-06	5
BP	GO:0070663	9/71	222/18670	1.59E-07	2.48E-06	1.11E-06	9
BP	GO:0045137	9/71	223/18670	1.65E-07	2.56E-06	1.15E-06	9
BP	GO:0010574	5/71	34/18670	1.76E-07	2.72E-06	1.22E-06	5
BP	GO:0042326	12/71	468/18670	1.79E-07	2.76E-06	1.23E-06	12
BP	GO:0044843	10/71	298/18670	1.80E-07	2.76E-06	1.23E-06	10
BP	GO:0031960	8/71	162/18670	1.81E-07	2.76E-06	1.23E-06	8
BP	GO:0006631	11/71	383/18670	1.99E-07	3.03E-06	1.36E-06	11
BP	GO:0042108	6/71	67/18670	2.03E-07	3.05E-06	1.37E-06	6
BP	GO:0048662	6/71	67/18670	2.03E-07	3.05E-06	1.37E-06	6
BP	GO:0001893	5/71	35/18670	2.05E-07	3.05E-06	1.37E-06	5
BP	GO:1901030	5/71	35/18670	2.05E-07	3.05E-06	1.37E-06	5
BP	GO:1901653	11/71	385/18670	2.10E-07	3.12E-06	1.40E-06	11
BP	GO:0045787	11/71	389/18670	2.33E-07	3.44E-06	1.54E-06	11
BP	GO:0010573	5/71	36/18670	2.37E-07	3.46E-06	1.55E-06	5
BP	GO:1901099	5/71	36/18670	2.37E-07	3.46E-06	1.55E-06	5
BP	GO:2001240	5/71	36/18670	2.37E-07	3.46E-06	1.55E-06	5
BP	GO:0032963	7/71	115/18670	2.69E-07	3.90E-06	1.75E-06	7
BP	GO:2000116	9/71	239/18670	2.97E-07	4.29E-06	1.92E-06	9
BP	GO:0010822	7/71	117/18670	3.02E-07	4.35E-06	1.95E-06	7
BP	GO:0001936	8/71	176/18670	3.41E-07	4.89E-06	2.19E-06	8
BP	GO:0048872	9/71	246/18670	3.78E-07	5.40E-06	2.42E-06	9
BP	GO:0150076	6/71	75/18670	3.99E-07	5.67E-06	2.54E-06	6
BP	GO:1905477	7/71	122/18670	4.02E-07	5.69E-06	2.55E-06	7
BP	GO:0042180	9/71	248/18670	4.05E-07	5.70E-06	2.56E-06	9
BP	GO:0006352	9/71	249/18670	4.19E-07	5.88E-06	2.63E-06	9
BP	GO:0010565	8/71	181/18670	4.22E-07	5.90E-06	2.64E-06	8
BP	GO:0046902	6/71	76/18670	4.32E-07	5.99E-06	2.68E-06	6
BP	GO:0061045	6/71	76/18670	4.32E-07	5.99E-06	2.68E-06	6
BP	GO:0031649	4/71	17/18670	4.40E-07	6.07E-06	2.72E-06	4
BP	GO:0051101	7/71	124/18670	4.49E-07	6.18E-06	2.77E-06	7
BP	GO:0051235	10/71	330/18670	4.62E-07	6.32E-06	2.83E-06	10
BP	GO:0008202	10/71	331/18670	4.74E-07	6.44E-06	2.89E-06	10
BP	GO:2000134	7/71	125/18670	4.75E-07	6.44E-06	2.89E-06	7
BP	GO:0051924	9/71	254/18670	4.95E-07	6.69E-06	3.00E-06	9
BP	GO:0030336	10/71	334/18670	5.15E-07	6.94E-06	3.11E-06	10
BP	GO:0030308	8/71	186/18670	5.20E-07	6.97E-06	3.12E-06	8
BP	GO:0050730	9/71	256/18670	5.28E-07	7.06E-06	3.16E-06	9
BP	GO:0043536	6/71	79/18670	5.45E-07	7.22E-06	3.23E-06	6
BP	GO:0071260	6/71	79/18670	5.45E-07	7.22E-06	3.23E-06	6
BP	GO:0006367	8/71	188/18670	5.64E-07	7.44E-06	3.33E-06	8
BP	GO:0045930	10/71	338/18670	5.74E-07	7.55E-06	3.38E-06	10
BP	GO:0001933	11/71	429/18670	6.15E-07	8.06E-06	3.61E-06	11
BP	GO:0050671	7/71	130/18670	6.19E-07	8.08E-06	3.62E-06	7
BP	GO:0001935	8/71	191/18670	6.35E-07	8.26E-06	3.70E-06	8
BP	GO:0032946	7/71	131/18670	6.52E-07	8.41E-06	3.77E-06	7
BP	GO:1902807	7/71	131/18670	6.52E-07	8.41E-06	3.77E-06	7
BP	GO:1901028	5/71	44/18670	6.67E-07	8.57E-06	3.84E-06	5
BP	GO:0045913	6/71	83/18670	7.31E-07	9.35E-06	4.19E-06	6
BP	GO:0007006	7/71	134/18670	7.61E-07	9.66E-06	4.33E-06	7
BP	GO:0046683	7/71	134/18670	7.61E-07	9.66E-06	4.33E-06	7
BP	GO:2000146	10/71	349/18670	7.69E-07	9.73E-06	4.36E-06	10
BP	GO:0002685	8/71	196/18670	7.73E-07	9.74E-06	4.36E-06	8
BP	GO:0007548	9/71	270/18670	8.24E-07	1.04E-05	4.64E-06	9
BP	GO:0031099	8/71	198/18670	8.34E-07	1.04E-05	4.68E-06	8
BP	GO:1904705	6/71	85/18670	8.42E-07	1.05E-05	4.68E-06	6

BP	GO:1990874	6/71	85/18670	8.42E-07	1.05E-05	4.68E-06	6
BP	GO:1901990	11/71	444/18670	8.62E-07	1.07E-05	4.78E-06	11
BP	GO:0001505	10/71	354/18670	8.75E-07	1.08E-05	4.83E-06	10
BP	GO:0072655	7/71	137/18670	8.83E-07	1.08E-05	4.86E-06	7
BP	GO:0070542	6/71	86/18670	9.02E-07	1.10E-05	4.92E-06	6
BP	GO:0090559	6/71	86/18670	9.02E-07	1.10E-05	4.92E-06	6
BP	GO:0043542	9/71	273/18670	9.04E-07	1.10E-05	4.92E-06	9
BP	GO:0006953	5/71	47/18670	9.34E-07	1.13E-05	5.05E-06	5
BP	GO:2001239	5/71	47/18670	9.34E-07	1.13E-05	5.05E-06	5
BP	GO:0043270	9/71	275/18670	9.60E-07	1.15E-05	5.17E-06	9
BP	GO:0001892	6/71	87/18670	9.66E-07	1.16E-05	5.18E-06	6
BP	GO:0070665	7/71	139/18670	9.74E-07	1.16E-05	5.21E-06	7
BP	GO:0052547	11/71	452/18670	1.03E-06	1.22E-05	5.47E-06	11
BP	GO:0090199	5/71	48/18670	1.04E-06	1.23E-05	5.52E-06	5
BP	GO:0031331	10/71	361/18670	1.04E-06	1.23E-05	5.53E-06	10
BP	GO:0070585	7/71	141/18670	1.07E-06	1.26E-05	5.65E-06	7
BP	GO:0055093	4/71	21/18670	1.09E-06	1.28E-05	5.75E-06	4
BP	GO:0038127	7/71	142/18670	1.12E-06	1.30E-05	5.84E-06	7
BP	GO:0006109	8/71	206/18670	1.12E-06	1.30E-05	5.84E-06	8
BP	GO:0050679	8/71	206/18670	1.12E-06	1.30E-05	5.84E-06	8
BP	GO:0007623	8/71	208/18670	1.21E-06	1.40E-05	6.26E-06	8
BP	GO:0071229	8/71	209/18670	1.25E-06	1.44E-05	6.44E-06	8
BP	GO:0014909	6/71	91/18670	1.26E-06	1.44E-05	6.44E-06	6
BP	GO:0045639	6/71	91/18670	1.26E-06	1.44E-05	6.44E-06	6
BP	GO:0060333	6/71	91/18670	1.26E-06	1.44E-05	6.44E-06	6
BP	GO:0034976	9/71	285/18670	1.29E-06	1.47E-05	6.57E-06	9
BP	GO:0071901	7/71	145/18670	1.29E-06	1.47E-05	6.57E-06	7
BP	GO:0010869	4/71	22/18670	1.33E-06	1.51E-05	6.74E-06	4
BP	GO:0042542	7/71	146/18670	1.36E-06	1.52E-05	6.81E-06	7
BP	GO:0045834	7/71	146/18670	1.36E-06	1.52E-05	6.81E-06	7
BP	GO:0001701	10/71	373/18670	1.40E-06	1.57E-05	7.03E-06	10
BP	GO:0036473	6/71	93/18670	1.43E-06	1.60E-05	7.15E-06	6
BP	GO:0014065	7/71	148/18670	1.48E-06	1.65E-05	7.38E-06	7
BP	GO:0043393	8/71	217/18670	1.66E-06	1.84E-05	8.23E-06	8
BP	GO:0030195	5/71	53/18670	1.72E-06	1.89E-05	8.48E-06	5
BP	GO:0019217	6/71	96/18670	1.73E-06	1.90E-05	8.50E-06	6
BP	GO:0051271	10/71	384/18670	1.82E-06	2.00E-05	8.94E-06	10
BP	GO:0015908	6/71	97/18670	1.83E-06	2.00E-05	8.98E-06	6
BP	GO:1901987	11/71	480/18670	1.84E-06	2.01E-05	8.99E-06	11
BP	GO:0030168	7/71	153/18670	1.85E-06	2.01E-05	9.01E-06	7
BP	GO:0002763	5/71	54/18670	1.89E-06	2.02E-05	9.05E-06	5
BP	GO:0097345	5/71	54/18670	1.89E-06	2.02E-05	9.05E-06	5
BP	GO:1900047	5/71	54/18670	1.89E-06	2.02E-05	9.05E-06	5
BP	GO:1904645	5/71	54/18670	1.89E-06	2.02E-05	9.05E-06	5
BP	GO:0018105	9/71	299/18670	1.92E-06	2.05E-05	9.17E-06	9
BP	GO:0046697	4/71	24/18670	1.93E-06	2.05E-05	9.18E-06	4
BP	GO:0050727	11/71	485/18670	2.04E-06	2.16E-05	9.68E-06	11
BP	GO:0001541	5/71	55/18670	2.07E-06	2.19E-05	9.80E-06	5
BP	GO:0007259	7/71	156/18670	2.11E-06	2.21E-05	9.91E-06	7
BP	GO:0042129	7/71	156/18670	2.11E-06	2.21E-05	9.91E-06	7
BP	GO:0043535	7/71	156/18670	2.11E-06	2.21E-05	9.91E-06	7
BP	GO:0043271	7/71	157/18670	2.20E-06	2.29E-05	1.03E-05	7
BP	GO:1905952	7/71	157/18670	2.20E-06	2.29E-05	1.03E-05	7
BP	GO:0040013	10/71	396/18670	2.40E-06	2.49E-05	1.12E-05	10
BP	GO:0050819	5/71	57/18670	2.48E-06	2.56E-05	1.15E-05	5
BP	GO:0032091	6/71	103/18670	2.61E-06	2.69E-05	1.21E-05	6
BP	GO:0030194	4/71	26/18670	2.69E-06	2.72E-05	1.22E-05	4
BP	GO:0032800	4/71	26/18670	2.69E-06	2.72E-05	1.22E-05	4
BP	GO:1900048	4/71	26/18670	2.69E-06	2.72E-05	1.22E-05	4
BP	GO:1900739	4/71	26/18670	2.69E-06	2.72E-05	1.22E-05	4
BP	GO:1900740	4/71	26/18670	2.69E-06	2.72E-05	1.22E-05	4
BP	GO:0019229	5/71	58/18670	2.70E-06	2.72E-05	1.22E-05	5
BP	GO:0031663	5/71	58/18670	2.70E-06	2.72E-05	1.22E-05	5

BP	GO:0032722	5/71	58/18670	2.70E-06	2.72E-05	1.22E-05	5
BP	GO:0014812	6/71	104/18670	2.76E-06	2.76E-05	1.24E-05	6
BP	GO:0071887	6/71	104/18670	2.76E-06	2.76E-05	1.24E-05	6
BP	GO:0045931	7/71	163/18670	2.83E-06	2.82E-05	1.26E-05	7
BP	GO:0006469	8/71	235/18670	3.01E-06	2.99E-05	1.34E-05	8
BP	GO:0007093	7/71	165/18670	3.07E-06	3.04E-05	1.36E-05	7
BP	GO:0050820	4/71	27/18670	3.15E-06	3.11E-05	1.39E-05	4
BP	GO:1902175	4/71	27/18670	3.15E-06	3.11E-05	1.39E-05	4
BP	GO:0097696	7/71	166/18670	3.19E-06	3.13E-05	1.40E-05	7
BP	GO:1902110	5/71	60/18670	3.20E-06	3.13E-05	1.40E-05	5
BP	GO:0007050	8/71	237/18670	3.20E-06	3.13E-05	1.40E-05	8
BP	GO:0090303	5/71	61/18670	3.48E-06	3.39E-05	1.52E-05	5
BP	GO:0018209	9/71	322/18670	3.52E-06	3.42E-05	1.53E-05	9
BP	GO:0032770	4/71	28/18670	3.67E-06	3.54E-05	1.58E-05	4
BP	GO:0036296	4/71	28/18670	3.67E-06	3.54E-05	1.58E-05	4
BP	GO:0042730	4/71	28/18670	3.67E-06	3.54E-05	1.58E-05	4
BP	GO:0030099	10/71	416/18670	3.72E-06	3.57E-05	1.60E-05	10
BP	GO:0002260	5/71	62/18670	3.77E-06	3.59E-05	1.61E-05	5
BP	GO:0046824	5/71	62/18670	3.77E-06	3.59E-05	1.61E-05	5
BP	GO:1902686	5/71	62/18670	3.77E-06	3.59E-05	1.61E-05	5
BP	GO:0009615	9/71	326/18670	3.89E-06	3.69E-05	1.65E-05	9
BP	GO:0010506	9/71	327/18670	3.99E-06	3.78E-05	1.69E-05	9
BP	GO:0001655	9/71	330/18670	4.29E-06	4.06E-05	1.82E-05	9
BP	GO:0009896	10/71	423/18670	4.31E-06	4.06E-05	1.82E-05	10
BP	GO:0035794	5/71	64/18670	4.42E-06	4.15E-05	1.86E-05	5
BP	GO:0043200	6/71	113/18670	4.47E-06	4.19E-05	1.88E-05	6
BP	GO:0052548	10/71	425/18670	4.49E-06	4.20E-05	1.88E-05	10
BP	GO:0090150	9/71	332/18670	4.51E-06	4.20E-05	1.88E-05	9
BP	GO:0042035	6/71	114/18670	4.71E-06	4.37E-05	1.96E-05	6
BP	GO:0071902	9/71	334/18670	4.73E-06	4.38E-05	1.96E-05	9
BP	GO:0071383	8/71	250/18670	4.75E-06	4.39E-05	1.97E-05	8
BP	GO:0048568	10/71	428/18670	4.78E-06	4.40E-05	1.97E-05	10
BP	GO:0001782	4/71	30/18670	4.88E-06	4.47E-05	2.00E-05	4
BP	GO:0071480	4/71	30/18670	4.88E-06	4.47E-05	2.00E-05	4
BP	GO:1902108	5/71	66/18670	5.15E-06	4.69E-05	2.10E-05	5
BP	GO:1905710	5/71	66/18670	5.15E-06	4.69E-05	2.10E-05	5
BP	GO:0007569	6/71	116/18670	5.21E-06	4.73E-05	2.12E-05	6
BP	GO:0071347	7/71	179/18670	5.25E-06	4.76E-05	2.13E-05	7
BP	GO:0043534	7/71	180/18670	5.45E-06	4.91E-05	2.20E-05	7
BP	GO:0071346	7/71	180/18670	5.45E-06	4.91E-05	2.20E-05	7
BP	GO:0010165	4/71	31/18670	5.59E-06	5.01E-05	2.25E-05	4
BP	GO:1902895	4/71	31/18670	5.59E-06	5.01E-05	2.25E-05	4
BP	GO:0048015	7/71	181/18670	5.65E-06	5.04E-05	2.26E-05	7
BP	GO:0006839	8/71	256/18670	5.65E-06	5.04E-05	2.26E-05	8
BP	GO:0033673	8/71	257/18670	5.82E-06	5.17E-05	2.32E-05	8
BP	GO:0019915	5/71	68/18670	5.97E-06	5.29E-05	2.37E-05	5
BP	GO:0001660	3/71	10/18670	6.20E-06	5.46E-05	2.45E-05	3
BP	GO:0032025	3/71	10/18670	6.20E-06	5.46E-05	2.45E-05	3
BP	GO:0051918	3/71	10/18670	6.20E-06	5.46E-05	2.45E-05	3
BP	GO:0048017	7/71	184/18670	6.29E-06	5.52E-05	2.47E-05	7
BP	GO:0042698	5/71	69/18670	6.41E-06	5.61E-05	2.52E-05	5
BP	GO:0050766	5/71	70/18670	6.89E-06	6.01E-05	2.69E-05	5
BP	GO:0010039	4/71	33/18670	7.23E-06	6.30E-05	2.82E-05	4
BP	GO:0042089	6/71	123/18670	7.30E-06	6.34E-05	2.84E-05	6
BP	GO:0030098	9/71	353/18670	7.40E-06	6.41E-05	2.87E-05	9
BP	GO:0014066	6/71	124/18670	7.65E-06	6.59E-05	2.95E-05	6
BP	GO:0042107	6/71	124/18670	7.65E-06	6.59E-05	2.95E-05	6
BP	GO:0051881	5/71	72/18670	7.91E-06	6.79E-05	3.04E-05	5
BP	GO:0061180	5/71	72/18670	7.91E-06	6.79E-05	3.04E-05	5
BP	GO:0045471	6/71	125/18670	8.01E-06	6.84E-05	3.06E-05	6
BP	GO:0042594	7/71	191/18670	8.04E-06	6.84E-05	3.06E-05	7
BP	GO:0071478	7/71	191/18670	8.04E-06	6.84E-05	3.06E-05	7
BP	GO:0043491	8/71	269/18670	8.12E-06	6.90E-05	3.09E-05	8

BP	GO:0043276	4/71	34/18670	8.17E-06	6.92E-05	3.10E-05	4
BP	GO:0050731	7/71	192/18670	8.32E-06	7.02E-05	3.15E-05	7
BP	GO:0006801	5/71	73/18670	8.47E-06	7.12E-05	3.19E-05	5
BP	GO:1903524	5/71	73/18670	8.47E-06	7.12E-05	3.19E-05	5
BP	GO:0046886	3/71	11/18670	8.51E-06	7.13E-05	3.19E-05	3
BP	GO:0032368	6/71	127/18670	8.78E-06	7.34E-05	3.29E-05	6
BP	GO:1900182	5/71	74/18670	9.06E-06	7.54E-05	3.38E-05	5
BP	GO:1903036	5/71	74/18670	9.06E-06	7.54E-05	3.38E-05	5
BP	GO:0010595	6/71	128/18670	9.18E-06	7.62E-05	3.41E-05	6
BP	GO:0018108	9/71	363/18670	9.26E-06	7.65E-05	3.43E-05	9
BP	GO:0045862	9/71	363/18670	9.26E-06	7.65E-05	3.43E-05	9
BP	GO:0006066	9/71	364/18670	9.47E-06	7.80E-05	3.49E-05	9
BP	GO:0006869	9/71	365/18670	9.68E-06	7.95E-05	3.56E-05	9
BP	GO:0018212	9/71	366/18670	9.89E-06	8.11E-05	3.63E-05	9
BP	GO:0001822	8/71	278/18670	1.03E-05	8.43E-05	3.77E-05	8
BP	GO:0042310	5/71	76/18670	1.03E-05	8.43E-05	3.77E-05	5
BP	GO:0017038	7/71	199/18670	1.05E-05	8.53E-05	3.82E-05	7
BP	GO:0034341	7/71	199/18670	1.05E-05	8.53E-05	3.82E-05	7
BP	GO:0048678	5/71	77/18670	1.10E-05	8.92E-05	4.00E-05	5
BP	GO:0030100	8/71	281/18670	1.12E-05	9.02E-05	4.04E-05	8
BP	GO:0045080	3/71	12/18670	1.13E-05	9.12E-05	4.08E-05	3
BP	GO:0034405	4/71	37/18670	1.15E-05	9.21E-05	4.12E-05	4
BP	GO:0043029	4/71	37/18670	1.15E-05	9.21E-05	4.12E-05	4
BP	GO:0045923	4/71	37/18670	1.15E-05	9.21E-05	4.12E-05	4
BP	GO:2000273	4/71	37/18670	1.15E-05	9.21E-05	4.12E-05	4
BP	GO:0072332	5/71	78/18670	1.17E-05	9.34E-05	4.18E-05	5
BP	GO:0032409	8/71	283/18670	1.17E-05	9.34E-05	4.18E-05	8
BP	GO:0032355	6/71	134/18670	1.19E-05	9.46E-05	4.24E-05	6
BP	GO:0002573	7/71	204/18670	1.23E-05	9.74E-05	4.36E-05	7
BP	GO:0034764	7/71	204/18670	1.23E-05	9.74E-05	4.36E-05	7
BP	GO:0050921	6/71	135/18670	1.25E-05	9.80E-05	4.39E-05	6
BP	GO:0046425	6/71	137/18670	1.35E-05	0.000106314	4.76E-05	6
BP	GO:0070555	7/71	207/18670	1.36E-05	0.000106314	4.76E-05	7
BP	GO:0007162	8/71	289/18670	1.37E-05	0.00010689	4.79E-05	8
BP	GO:0070838	10/71	483/18670	1.37E-05	0.000107181	4.80E-05	10
BP	GO:0048708	5/71	81/18670	1.41E-05	0.000109801	4.92E-05	5
BP	GO:0051701	7/71	209/18670	1.44E-05	0.0001121	5.02E-05	7
BP	GO:0090399	3/71	13/18670	1.47E-05	0.000113052	5.06E-05	3
BP	GO:0090594	3/71	13/18670	1.47E-05	0.000113052	5.06E-05	3
BP	GO:1902947	3/71	13/18670	1.47E-05	0.000113052	5.06E-05	3
BP	GO:0032642	5/71	82/18670	1.50E-05	0.000114987	5.15E-05	5
BP	GO:0034644	5/71	82/18670	1.50E-05	0.000114987	5.15E-05	5
BP	GO:0072001	8/71	293/18670	1.51E-05	0.000115396	5.17E-05	8
BP	GO:0009165	9/71	386/18670	1.51E-05	0.000115396	5.17E-05	9
BP	GO:0072511	10/71	489/18670	1.53E-05	0.000116526	5.22E-05	10
BP	GO:0071470	4/71	40/18670	1.58E-05	0.000120301	5.39E-05	4
BP	GO:1901293	9/71	390/18670	1.64E-05	0.000124371	5.57E-05	9
BP	GO:0014910	5/71	84/18670	1.69E-05	0.0001273	5.70E-05	5
BP	GO:0097756	5/71	84/18670	1.69E-05	0.0001273	5.70E-05	5
BP	GO:0006914	10/71	496/18670	1.73E-05	0.000129373	5.80E-05	10
BP	GO:0061919	10/71	496/18670	1.73E-05	0.000129373	5.80E-05	10
BP	GO:0006606	6/71	143/18670	1.73E-05	0.000129373	5.80E-05	6
BP	GO:0030879	6/71	143/18670	1.73E-05	0.000129373	5.80E-05	6
BP	GO:0000075	7/71	216/18670	1.79E-05	0.000133447	5.98E-05	7
BP	GO:1904064	6/71	144/18670	1.80E-05	0.000133998	6.00E-05	6
BP	GO:0050920	7/71	217/18670	1.84E-05	0.000136872	6.13E-05	7
BP	GO:0051917	3/71	14/18670	1.86E-05	0.000138095	6.19E-05	3
BP	GO:0001776	5/71	86/18670	1.89E-05	0.000139618	6.25E-05	5
BP	GO:0006919	5/71	86/18670	1.89E-05	0.000139618	6.25E-05	5
BP	GO:0035264	6/71	146/18670	1.94E-05	0.000142999	6.41E-05	6
BP	GO:1904892	6/71	146/18670	1.94E-05	0.000142999	6.41E-05	6
BP	GO:0060326	8/71	304/18670	1.97E-05	0.000144471	6.47E-05	8
BP	GO:0010876	9/71	400/18670	2.00E-05	0.000146612	6.57E-05	9

BP	GO:0002262	6/71	147/18670	2.02E-05	0.000147669	6.62E-05	6
BP	GO:1904035	5/71	88/18670	2.11E-05	0.000154072	6.90E-05	5
BP	GO:0006775	4/71	43/18670	2.12E-05	0.000154072	6.90E-05	4
BP	GO:0009895	8/71	308/18670	2.16E-05	0.000156952	7.03E-05	8
BP	GO:0009267	6/71	149/18670	2.18E-05	0.000158035	7.08E-05	6
BP	GO:0045444	7/71	223/18670	2.19E-05	0.00015857	7.10E-05	7
BP	GO:0032602	5/71	89/18670	2.23E-05	0.000160694	7.20E-05	5
BP	GO:0046427	5/71	89/18670	2.23E-05	0.000160694	7.20E-05	5
BP	GO:0042113	8/71	310/18670	2.27E-05	0.000162639	7.29E-05	8
BP	GO:0030730	3/71	15/18670	2.32E-05	0.000165406	7.41E-05	3
BP	GO:0032352	3/71	15/18670	2.32E-05	0.000165406	7.41E-05	3
BP	GO:0045073	3/71	15/18670	2.32E-05	0.000165406	7.41E-05	3
BP	GO:0046688	4/71	44/18670	2.32E-05	0.000165406	7.41E-05	4
BP	GO:0007517	9/71	410/18670	2.43E-05	0.00017278	7.74E-05	9
BP	GO:0045807	6/71	153/18670	2.53E-05	0.000179679	8.05E-05	6
BP	GO:0010594	7/71	229/18670	2.60E-05	0.000184053	8.25E-05	7
BP	GO:1904894	5/71	92/18670	2.62E-05	0.000185138	8.29E-05	5
BP	GO:0006984	4/71	46/18670	2.78E-05	0.00019521	8.75E-05	4
BP	GO:0045776	4/71	46/18670	2.78E-05	0.00019521	8.75E-05	4
BP	GO:0042445	7/71	232/18670	2.83E-05	0.000196515	8.80E-05	7
BP	GO:0034767	6/71	156/18670	2.83E-05	0.000196515	8.80E-05	6
BP	GO:0051222	9/71	418/18670	2.83E-05	0.000196515	8.80E-05	9
BP	GO:0010885	3/71	16/18670	2.85E-05	0.000196515	8.80E-05	3
BP	GO:0042033	3/71	16/18670	2.85E-05	0.000196515	8.80E-05	3
BP	GO:0045986	3/71	16/18670	2.85E-05	0.000196515	8.80E-05	3
BP	GO:0050755	3/71	16/18670	2.85E-05	0.000196515	8.80E-05	3
BP	GO:0071380	3/71	16/18670	2.85E-05	0.000196515	8.80E-05	3
BP	GO:1900451	3/71	16/18670	2.85E-05	0.000196515	8.80E-05	3
BP	GO:0070231	4/71	47/18670	3.03E-05	0.000208389	9.34E-05	4
BP	GO:0043467	6/71	158/18670	3.04E-05	0.000208756	9.35E-05	6
BP	GO:0042100	5/71	95/18670	3.06E-05	0.000209169	9.37E-05	5
BP	GO:0042102	5/71	95/18670	3.06E-05	0.000209169	9.37E-05	5
BP	GO:1903076	5/71	95/18670	3.06E-05	0.000209169	9.37E-05	5
BP	GO:0048010	5/71	96/18670	3.22E-05	0.000219567	9.84E-05	5
BP	GO:0051188	8/71	326/18670	3.24E-05	0.000220583	9.88E-05	8
BP	GO:0051972	4/71	48/18670	3.29E-05	0.000223471	0.000100112	4
BP	GO:0019058	8/71	328/18670	3.39E-05	0.000229445	0.000102789	8
BP	GO:0070242	3/71	17/18670	3.45E-05	0.000232707	0.00010425	3
BP	GO:2001267	3/71	17/18670	3.45E-05	0.000232707	0.00010425	3
BP	GO:0051052	9/71	429/18670	3.47E-05	0.000233411	0.000104565	9
BP	GO:0015718	6/71	162/18670	3.50E-05	0.000234947	0.000105253	6
BP	GO:0050764	5/71	98/18670	3.56E-05	0.000238636	0.000106906	5
BP	GO:0097300	4/71	49/18670	3.57E-05	0.000239249	0.000107181	4
BP	GO:0031214	6/71	163/18670	3.62E-05	0.000241266	0.000108084	6
BP	GO:0051170	6/71	163/18670	3.62E-05	0.000241266	0.000108084	6
BP	GO:0032147	8/71	333/18670	3.77E-05	0.000250866	0.000112385	8
BP	GO:0006816	9/71	434/18670	3.79E-05	0.000251903	0.00011285	9
BP	GO:0002931	4/71	50/18670	3.87E-05	0.000256295	0.000114817	4
BP	GO:0050680	6/71	165/18670	3.88E-05	0.000256295	0.000114817	6
BP	GO:0043434	9/71	436/18670	3.93E-05	0.000259527	0.000116265	9
BP	GO:0010878	3/71	18/18670	4.13E-05	0.000271921	0.000121818	3
BP	GO:1901568	6/71	167/18670	4.15E-05	0.000272564	0.000122105	6
BP	GO:0048146	4/71	51/18670	4.19E-05	0.000275082	0.000123233	4
BP	GO:0097237	7/71	247/18670	4.22E-05	0.000276038	0.000123662	7
BP	GO:0010522	5/71	102/18670	4.31E-05	0.000281932	0.000126302	5
BP	GO:1901991	7/71	248/18670	4.32E-05	0.000282102	0.000126378	7
BP	GO:0050728	6/71	169/18670	4.43E-05	0.00028827	0.000129142	6
BP	GO:0019318	7/71	249/18670	4.44E-05	0.00028827	0.000129142	7
BP	GO:1904062	8/71	342/18670	4.55E-05	0.000295284	0.000132284	8
BP	GO:0072330	8/71	343/18670	4.65E-05	0.000300845	0.000134775	8
BP	GO:0016241	6/71	171/18670	4.73E-05	0.000305291	0.000136767	6
BP	GO:0046822	5/71	104/18670	4.73E-05	0.000305291	0.000136767	5
BP	GO:0045840	4/71	53/18670	4.88E-05	0.000311619	0.000139602	4

BP	GO:0010544	3/71	19/18670	4.89E-05	0.000311619	0.000139602	3
BP	GO:0010888	3/71	19/18670	4.89E-05	0.000311619	0.000139602	3
BP	GO:0033189	3/71	19/18670	4.89E-05	0.000311619	0.000139602	3
BP	GO:0051546	3/71	19/18670	4.89E-05	0.000311619	0.000139602	3
BP	GO:1902176	3/71	19/18670	4.89E-05	0.000311619	0.000139602	3
BP	GO:0001659	6/71	173/18670	5.05E-05	0.000321235	0.000143909	6
BP	GO:0044774	5/71	106/18670	5.19E-05	0.000329386	0.000147561	5
BP	GO:0010524	4/71	54/18670	5.26E-05	0.000332767	0.000149076	4
BP	GO:0051353	4/71	54/18670	5.26E-05	0.000332767	0.000149076	4
BP	GO:0007611	7/71	256/18670	5.29E-05	0.000333835	0.000149554	7
BP	GO:0006986	6/71	176/18670	5.56E-05	0.000350182	0.000156878	6
BP	GO:2000278	5/71	108/18670	5.67E-05	0.000356804	0.000159844	5
BP	GO:0048469	6/71	177/18670	5.73E-05	0.000360038	0.000161293	6
BP	GO:0051091	7/71	261/18670	5.97E-05	0.000374328	0.000167695	7
BP	GO:0006977	4/71	56/18670	6.07E-05	0.000379255	0.000169902	4
BP	GO:0031295	4/71	56/18670	6.07E-05	0.000379255	0.000169902	4
BP	GO:0051099	6/71	179/18670	6.10E-05	0.00038038	0.000170406	6
BP	GO:0034504	7/71	262/18670	6.12E-05	0.000380606	0.000170507	7
BP	GO:0046394	9/71	462/18670	6.15E-05	0.000382006	0.000171134	9
BP	GO:0016101	5/71	110/18670	6.19E-05	0.000383711	0.000171898	5
BP	GO:0016053	9/71	463/18670	6.26E-05	0.000386973	0.00017336	9
BP	GO:0031294	4/71	57/18670	6.51E-05	0.000400007	0.000179199	4
BP	GO:0071398	4/71	57/18670	6.51E-05	0.000400007	0.000179199	4
BP	GO:0072431	4/71	57/18670	6.51E-05	0.000400007	0.000179199	4
BP	GO:1902400	4/71	57/18670	6.51E-05	0.000400007	0.000179199	4
BP	GO:0036499	3/71	21/18670	6.67E-05	0.000408865	0.000183167	3
BP	GO:0043254	9/71	467/18670	6.68E-05	0.000408865	0.000183167	9
BP	GO:0046883	7/71	266/18670	6.73E-05	0.00040979	0.000183581	7
BP	GO:0001938	5/71	112/18670	6.75E-05	0.00040979	0.000183581	5
BP	GO:0043279	5/71	112/18670	6.75E-05	0.00040979	0.000183581	5
BP	GO:1990748	5/71	112/18670	6.75E-05	0.00040979	0.000183581	5
BP	GO:1901988	7/71	267/18670	6.89E-05	0.000417786	0.000187163	7
BP	GO:0045861	8/71	363/18670	6.92E-05	0.000418683	0.000187565	8
BP	GO:0061900	4/71	58/18670	6.98E-05	0.000420703	0.00018847	4
BP	GO:1903749	4/71	58/18670	6.98E-05	0.000420703	0.00018847	4
BP	GO:0031334	7/71	268/18670	7.05E-05	0.000424634	0.000190231	7
BP	GO:1903708	6/71	185/18670	7.33E-05	0.000439434	0.000196862	6
BP	GO:0002688	5/71	114/18670	7.34E-05	0.000439434	0.000196862	5
BP	GO:0006690	5/71	114/18670	7.34E-05	0.000439434	0.000196862	5
BP	GO:0045927	7/71	270/18670	7.39E-05	0.000441732	0.000197891	7
BP	GO:0043388	4/71	59/18670	7.46E-05	0.000442059	0.000198037	4
BP	GO:0072413	4/71	59/18670	7.46E-05	0.000442059	0.000198037	4
BP	GO:1902402	4/71	59/18670	7.46E-05	0.000442059	0.000198037	4
BP	GO:1902403	4/71	59/18670	7.46E-05	0.000442059	0.000198037	4
BP	GO:2000351	4/71	59/18670	7.46E-05	0.000442059	0.000198037	4
BP	GO:1904375	5/71	115/18670	7.65E-05	0.000451704	0.000202358	5
BP	GO:0035809	3/71	22/18670	7.71E-05	0.000451704	0.000202358	3
BP	GO:0042359	3/71	22/18670	7.71E-05	0.000451704	0.000202358	3
BP	GO:0043496	3/71	22/18670	7.71E-05	0.000451704	0.000202358	3
BP	GO:0071379	3/71	22/18670	7.71E-05	0.000451704	0.000202358	3
BP	GO:1901522	3/71	22/18670	7.71E-05	0.000451704	0.000202358	3
BP	GO:0032868	7/71	272/18670	7.74E-05	0.000452159	0.000202562	7
BP	GO:1902105	7/71	272/18670	7.74E-05	0.000452159	0.000202562	7
BP	GO:0021782	5/71	116/18670	7.97E-05	0.000464033	0.000207881	5
BP	GO:1900180	5/71	116/18670	7.97E-05	0.000464033	0.000207881	5
BP	GO:0002761	5/71	117/18670	8.30E-05	0.000480822	0.000215403	5
BP	GO:0010906	5/71	117/18670	8.30E-05	0.000480822	0.000215403	5
BP	GO:1900371	5/71	117/18670	8.30E-05	0.000480822	0.000215403	5
BP	GO:1905953	4/71	61/18670	8.51E-05	0.000491813	0.000220326	4
BP	GO:0030808	5/71	118/18670	8.64E-05	0.000498067	0.000223128	5
BP	GO:0034765	9/71	483/18670	8.65E-05	0.000498067	0.000223128	9
BP	GO:0034695	3/71	23/18670	8.84E-05	0.00050827	0.000227699	3
BP	GO:0060485	7/71	278/18670	8.87E-05	0.000509374	0.000228193	7

BP	GO:0043112	6/71	192/18670	8.99E-05	0.000515499	0.000230938	6
BP	GO:0070059	4/71	62/18670	9.07E-05	0.000517976	0.000232047	4
BP	GO:0070265	4/71	62/18670	9.07E-05	0.000517976	0.000232047	4
BP	GO:0003014	5/71	120/18670	9.36E-05	0.000532003	0.000238331	5
BP	GO:0006721	5/71	120/18670	9.36E-05	0.000532003	0.000238331	5
BP	GO:0022612	5/71	120/18670	9.36E-05	0.000532003	0.000238331	5
BP	GO:0031571	4/71	63/18670	9.66E-05	0.000546854	0.000244984	4
BP	GO:0044819	4/71	63/18670	9.66E-05	0.000546854	0.000244984	4
BP	GO:0043618	5/71	121/18670	9.74E-05	0.000550507	0.000246621	5
BP	GO:0021700	7/71	284/18670	0.000101348	0.000572086	0.000256288	7
BP	GO:0044783	4/71	64/18670	0.000102694	0.000576773	0.000258388	4
BP	GO:0045600	4/71	64/18670	0.000102694	0.000576773	0.000258388	4
BP	GO:2000378	4/71	64/18670	0.000102694	0.000576773	0.000258388	4
BP	GO:0010952	6/71	197/18670	0.000103598	0.000580876	0.000260226	6
BP	GO:0051928	5/71	123/18670	0.000105204	0.000588895	0.000263818	5
BP	GO:0044262	7/71	286/18670	0.000105869	0.000591633	0.000265045	7
BP	GO:0046890	6/71	198/18670	0.000106518	0.000594265	0.000266224	6
BP	GO:0002548	4/71	65/18670	0.000109115	0.000604879	0.000270979	4
BP	GO:0006940	4/71	65/18670	0.000109115	0.000604879	0.000270979	4
BP	GO:0072577	4/71	65/18670	0.000109115	0.000604879	0.000270979	4
BP	GO:0007179	6/71	199/18670	0.000109502	0.000604879	0.000270979	6
BP	GO:0035966	6/71	199/18670	0.000109502	0.000604879	0.000270979	6
BP	GO:0050866	6/71	199/18670	0.000109502	0.000604879	0.000270979	6
BP	GO:0009299	3/71	25/18670	0.000114148	0.000626414	0.000280626	3
BP	GO:0019430	3/71	25/18670	0.000114148	0.000626414	0.000280626	3
BP	GO:0032461	3/71	25/18670	0.000114148	0.000626414	0.000280626	3
BP	GO:0060571	3/71	25/18670	0.000114148	0.000626414	0.000280626	3
BP	GO:0040014	4/71	66/18670	0.000115823	0.0006325	0.000283353	4
BP	GO:0051785	4/71	66/18670	0.000115823	0.0006325	0.000283353	4
BP	GO:0051926	4/71	66/18670	0.000115823	0.0006325	0.000283353	4
BP	GO:0005996	7/71	292/18670	0.00012043	0.000656586	0.000294143	7
BP	GO:0030278	6/71	203/18670	0.000122114	0.000664689	0.000297773	6
BP	GO:0043620	5/71	127/18670	0.000122338	0.000664831	0.000297837	5
BP	GO:0019748	4/71	67/18670	0.000122825	0.000666307	0.000298498	4
BP	GO:0051146	7/71	293/18670	0.000123008	0.000666307	0.000298498	7
BP	GO:0035265	6/71	204/18670	0.00012544	0.00067839	0.000303911	6
BP	GO:0002687	5/71	128/18670	0.000126938	0.000685385	0.000307045	5
BP	GO:0033627	4/71	68/18670	0.000130129	0.000700356	0.000313751	4
BP	GO:0071230	4/71	68/18670	0.000130129	0.000700356	0.000313751	4
BP	GO:0050890	7/71	296/18670	0.000131012	0.00070398	0.000315375	7
BP	GO:0071482	5/71	129/18670	0.00013167	0.000706382	0.000316451	5
BP	GO:0002064	6/71	207/18670	0.000135853	0.000727662	0.000325984	6
BP	GO:0070227	4/71	69/18670	0.000137742	0.000736601	0.000329989	4
BP	GO:0090276	6/71	208/18670	0.000139473	0.000744669	0.000333603	6
BP	GO:0030183	5/71	131/18670	0.000141537	0.000753292	0.000337466	5
BP	GO:0098754	5/71	131/18670	0.000141537	0.000753292	0.000337466	5
BP	GO:0006164	7/71	300/18670	0.000142338	0.000756357	0.000338839	7
BP	GO:0006006	6/71	209/18670	0.000143168	0.000757585	0.000339389	6
BP	GO:0009651	3/71	27/18670	0.000144377	0.000757585	0.000339389	3
BP	GO:0035902	3/71	27/18670	0.000144377	0.000757585	0.000339389	3
BP	GO:0045932	3/71	27/18670	0.000144377	0.000757585	0.000339389	3
BP	GO:0046885	3/71	27/18670	0.000144377	0.000757585	0.000339389	3
BP	GO:0048143	3/71	27/18670	0.000144377	0.000757585	0.000339389	3
BP	GO:0071450	3/71	27/18670	0.000144377	0.000757585	0.000339389	3
BP	GO:0071451	3/71	27/18670	0.000144377	0.000757585	0.000339389	3
BP	GO:0043280	5/71	132/18670	0.000146678	0.000767258	0.000343723	5
BP	GO:0045598	5/71	132/18670	0.000146678	0.000767258	0.000343723	5
BP	GO:0097529	6/71	210/18670	0.000146941	0.000767434	0.000343802	6
BP	GO:0042770	5/71	133/18670	0.000151961	0.00079242	0.000354995	5
BP	GO:0071479	4/71	71/18670	0.000153928	0.000801432	0.000359032	4
BP	GO:0060537	8/71	408/18670	0.000155194	0.000806767	0.000361422	8
BP	GO:0030260	5/71	134/18670	0.000157389	0.000813135	0.000364275	5
BP	GO:0044409	5/71	134/18670	0.000157389	0.000813135	0.000364275	5

BP	GO:0051806	5/71	134/18670	0.000157389	0.000813135	0.000364275	5
BP	GO:0051828	5/71	134/18670	0.000157389	0.000813135	0.000364275	5
BP	GO:1903747	4/71	72/18670	0.000162518	0.00083834	0.000375567	4
BP	GO:0031100	4/71	73/18670	0.000171448	0.000878997	0.000393781	4
BP	GO:0043627	4/71	73/18670	0.000171448	0.000878997	0.000393781	4
BP	GO:0072401	4/71	73/18670	0.000171448	0.000878997	0.000393781	4
BP	GO:0072422	4/71	73/18670	0.000171448	0.000878997	0.000393781	4
BP	GO:0000303	3/71	29/18670	0.00017938	0.00091826	0.00041137	3
BP	GO:0008584	5/71	138/18670	0.000180596	0.000920939	0.00041257	5
BP	GO:0031670	4/71	74/18670	0.000180727	0.000920939	0.00041257	4
BP	GO:0072395	4/71	74/18670	0.000180727	0.000920939	0.00041257	4
BP	GO:0046879	7/71	312/18670	0.000181178	0.000921837	0.000412972	7
BP	GO:0072522	7/71	313/18670	0.000184768	0.000938676	0.000420516	7
BP	GO:0006720	5/71	139/18670	0.000186786	0.000946061	0.000423825	5
BP	GO:0046546	5/71	139/18670	0.000186786	0.000946061	0.000423825	5
BP	GO:1903201	4/71	75/18670	0.000190364	0.00096273	0.000431292	4
BP	GO:0031098	7/71	315/18670	0.000192119	0.000970145	0.000434614	7
BP	GO:0000305	3/71	30/18670	0.000198768	0.000994731	0.000445628	3
BP	GO:0046320	3/71	30/18670	0.000198768	0.000994731	0.000445628	3
BP	GO:0048147	3/71	30/18670	0.000198768	0.000994731	0.000445628	3
BP	GO:0048873	3/71	30/18670	0.000198768	0.000994731	0.000445628	3
BP	GO:0071549	3/71	30/18670	0.000198768	0.000994731	0.000445628	3
BP	GO:0090200	3/71	30/18670	0.000198768	0.000994731	0.000445628	3
BP	GO:0010675	5/71	142/18670	0.000206331	0.001029503	0.000461206	5
BP	GO:1900542	5/71	142/18670	0.000206331	0.001029503	0.000461206	5
BP	GO:0030595	6/71	224/18670	0.00020854	0.001038981	0.000465451	6
BP	GO:0001649	6/71	225/18670	0.000213617	0.001062695	0.000476075	6
BP	GO:0051047	8/71	428/18670	0.000214978	0.001067882	0.000478399	8
BP	GO:0010543	3/71	31/18670	0.000219466	0.001083507	0.000485398	3
BP	GO:0033198	3/71	31/18670	0.000219466	0.001083507	0.000485398	3
BP	GO:0034694	3/71	31/18670	0.000219466	0.001083507	0.000485398	3
BP	GO:0045742	3/71	31/18670	0.000219466	0.001083507	0.000485398	3
BP	GO:0009914	7/71	322/18670	0.000219739	0.001083507	0.000485398	7
BP	GO:1902107	5/71	144/18670	0.000220201	0.001084193	0.000485706	5
BP	GO:0051054	6/71	228/18670	0.000229435	0.001127998	0.00050533	6
BP	GO:0006110	4/71	79/18670	0.000232646	0.00114211	0.000511652	4
BP	GO:0006140	5/71	146/18670	0.000234772	0.001150863	0.000515573	5
BP	GO:0042391	8/71	434/18670	0.000236217	0.001156255	0.000517989	8
BP	GO:0009743	6/71	230/18670	0.000240483	0.0011736	0.000525759	6
BP	GO:0038128	3/71	32/18670	0.00024151	0.0011736	0.000525759	3
BP	GO:0042311	3/71	32/18670	0.00024151	0.0011736	0.000525759	3
BP	GO:0046685	3/71	32/18670	0.00024151	0.0011736	0.000525759	3
BP	GO:0051968	3/71	32/18670	0.00024151	0.0011736	0.000525759	3
BP	GO:0019359	5/71	147/18670	0.000242327	0.001174164	0.000526012	5
BP	GO:0019363	5/71	147/18670	0.000242327	0.001174164	0.000526012	5
BP	GO:0022617	4/71	80/18670	0.000244191	0.001181491	0.000529294	4
BP	GO:0016485	7/71	328/18670	0.00024589	0.001187998	0.000532209	7
BP	GO:0051592	5/71	148/18670	0.000250065	0.00120643	0.000540467	5
BP	GO:0019932	8/71	439/18670	0.000255207	0.001229466	0.000550786	8
BP	GO:0030811	4/71	81/18670	0.000256143	0.001230442	0.000551224	4
BP	GO:1902930	4/71	81/18670	0.000256143	0.001230442	0.000551224	4
BP	GO:0051092	5/71	149/18670	0.00025799	0.001235774	0.000553612	5
BP	GO:2001056	5/71	149/18670	0.00025799	0.001235774	0.000553612	5
BP	GO:0016242	3/71	33/18670	0.000264938	0.00126364	0.000566096	3
BP	GO:0036003	3/71	33/18670	0.000264938	0.00126364	0.000566096	3
BP	GO:1901186	3/71	33/18670	0.000264938	0.00126364	0.000566096	3
BP	GO:0072525	5/71	150/18670	0.000266105	0.001267401	0.000567781	5
BP	GO:0071158	4/71	82/18670	0.00026851	0.001277043	0.0005721	4
BP	GO:0032872	6/71	237/18670	0.000282496	0.001341657	0.000601047	6
BP	GO:0090316	5/71	153/18670	0.000291617	0.001383015	0.000619575	5
BP	GO:0010507	4/71	84/18670	0.000294523	0.001386988	0.000621354	4
BP	GO:0043154	4/71	84/18670	0.000294523	0.001386988	0.000621354	4
BP	GO:0045445	4/71	84/18670	0.000294523	0.001386988	0.000621354	4

BP	GO:0046889	4/71	84/18670	0.000294523	0.001386988	0.000621354	4
BP	GO:1905897	4/71	84/18670	0.000294523	0.001386988	0.000621354	4
BP	GO:0031348	6/71	239/18670	0.000295501	0.001387698	0.000621673	6
BP	GO:0070302	6/71	239/18670	0.000295501	0.001387698	0.000621673	6
BP	GO:0030217	6/71	240/18670	0.000302178	0.001417068	0.00063483	6
BP	GO:0030262	3/71	35/18670	0.000316093	0.001474073	0.000660368	3
BP	GO:0032735	3/71	35/18670	0.000316093	0.001474073	0.000660368	3
BP	GO:0071312	3/71	35/18670	0.000316093	0.001474073	0.000660368	3
BP	GO:1904031	3/71	35/18670	0.000316093	0.001474073	0.000660368	3
BP	GO:0030856	5/71	156/18670	0.000318945	0.001485308	0.000665401	5
BP	GO:0034637	4/71	86/18670	0.000322297	0.001493043	0.000668866	4
BP	GO:0042058	4/71	86/18670	0.000322297	0.001493043	0.000668866	4
BP	GO:0042446	4/71	86/18670	0.000322297	0.001493043	0.000668866	4
BP	GO:0006913	7/71	343/18670	0.000322387	0.001493043	0.000668866	7
BP	GO:0010721	7/71	344/18670	0.000328098	0.001517034	0.000679614	7
BP	GO:0031570	5/71	157/18670	0.000328472	0.001517034	0.000679614	5
BP	GO:0051896	6/71	244/18670	0.00033008	0.001522363	0.000682001	6
BP	GO:0002690	4/71	87/18670	0.000336865	0.001549395	0.000694111	4
BP	GO:0014068	4/71	87/18670	0.000336865	0.001549395	0.000694111	4
BP	GO:0060402	5/71	158/18670	0.000338213	0.001553466	0.000695935	5
BP	GO:0051169	7/71	346/18670	0.000339764	0.001558453	0.000698169	7
BP	GO:0042554	3/71	36/18670	0.00034389	0.001575225	0.000705683	3
BP	GO:0034103	4/71	88/18670	0.0003519	0.001603152	0.000718193	4
BP	GO:0043470	4/71	88/18670	0.0003519	0.001603152	0.000718193	4
BP	GO:0070098	4/71	88/18670	0.0003519	0.001603152	0.000718193	4
BP	GO:1900407	4/71	88/18670	0.0003519	0.001603152	0.000718193	4
BP	GO:0007178	7/71	349/18670	0.000357886	0.001628121	0.00072938	7
BP	GO:0046661	5/71	160/18670	0.000358352	0.001628121	0.00072938	5
BP	GO:0071560	6/71	249/18670	0.000367758	0.001668595	0.000747511	6
BP	GO:0006921	3/71	37/18670	0.000373216	0.001675226	0.000750482	3
BP	GO:0014037	3/71	37/18670	0.000373216	0.001675226	0.000750482	3
BP	GO:0014912	3/71	37/18670	0.000373216	0.001675226	0.000750482	3
BP	GO:0030212	3/71	37/18670	0.000373216	0.001675226	0.000750482	3
BP	GO:0032885	3/71	37/18670	0.000373216	0.001675226	0.000750482	3
BP	GO:0033280	3/71	37/18670	0.000373216	0.001675226	0.000750482	3
BP	GO:0042307	3/71	37/18670	0.000373216	0.001675226	0.000750482	3
BP	GO:0046006	3/71	37/18670	0.000373216	0.001675226	0.000750482	3
BP	GO:0003012	8/71	465/18670	0.000375284	0.001681792	0.000753423	8
BP	GO:0030072	6/71	250/18670	0.000375682	0.001681792	0.000753423	6
BP	GO:0042475	4/71	90/18670	0.000383401	0.001711009	0.000766512	4
BP	GO:0071674	4/71	90/18670	0.000383401	0.001711009	0.000766512	4
BP	GO:0045637	6/71	251/18670	0.00038374	0.001711009	0.000766512	6
BP	GO:0031589	7/71	354/18670	0.00038981	0.001735765	0.000777603	7
BP	GO:1901992	4/71	91/18670	0.000399885	0.001778268	0.000796643	4
BP	GO:0035821	5/71	164/18670	0.00040134	0.001782374	0.000798483	5
BP	GO:0006959	7/71	356/18670	0.000403201	0.001782845	0.000798694	7
BP	GO:0032094	3/71	38/18670	0.000404104	0.001782845	0.000798694	3
BP	GO:0032350	3/71	38/18670	0.000404104	0.001782845	0.000798694	3
BP	GO:0048009	3/71	38/18670	0.000404104	0.001782845	0.000798694	3
BP	GO:1904706	3/71	38/18670	0.000404104	0.001782845	0.000798694	3
BP	GO:0071695	5/71	165/18670	0.000412671	0.001818245	0.000814553	5
BP	GO:0050708	8/71	472/18670	0.000414486	0.001823845	0.000817062	8
BP	GO:2000117	4/71	92/18670	0.000416869	0.001831567	0.000820521	4
BP	GO:0071559	6/71	255/18670	0.000417333	0.001831567	0.000820521	6
BP	GO:0016125	5/71	166/18670	0.000424242	0.001859453	0.000833014	5
BP	GO:1903706	8/71	475/18670	0.000432278	0.001892204	0.000847685	8
BP	GO:0003073	4/71	93/18670	0.000434363	0.001896378	0.000849556	4
BP	GO:1901184	4/71	93/18670	0.000434363	0.001896378	0.000849556	4
BP	GO:0010661	3/71	39/18670	0.00043659	0.001901149	0.000851693	3
BP	GO:1904591	3/71	39/18670	0.00043659	0.001901149	0.000851693	3
BP	GO:0010948	7/71	361/18670	0.000438299	0.001906118	0.000853919	7
BP	GO:0001654	7/71	362/18670	0.000445604	0.001935374	0.000867025	7
BP	GO:0050806	5/71	168/18670	0.000448117	0.001943772	0.000870788	5

BP	GO:0090257	6/71	259/18670	0.000453185	0.001963214	0.000879497	6
BP	GO:0051281	3/71	40/18670	0.000470706	0.002029524	0.000909203	3
BP	GO:0071548	3/71	40/18670	0.000470706	0.002029524	0.000909203	3
BP	GO:0050810	4/71	95/18670	0.000470913	0.002029524	0.000909203	4
BP	GO:0051196	4/71	95/18670	0.000470913	0.002029524	0.000909203	4
BP	GO:0009108	6/71	261/18670	0.00047199	0.002031555	0.000910113	6
BP	GO:0150063	7/71	366/18670	0.000475802	0.002045341	0.000916289	7
BP	GO:0060401	5/71	171/18670	0.000485814	0.002085704	0.000934371	5
BP	GO:0010660	4/71	96/18670	0.000489986	0.002095566	0.000938789	4
BP	GO:0097327	4/71	96/18670	0.000489986	0.002095566	0.000938789	4
BP	GO:2001169	4/71	96/18670	0.000489986	0.002095566	0.000938789	4
BP	GO:0021543	5/71	172/18670	0.000498896	0.002130854	0.000954598	5
BP	GO:0006909	7/71	369/18670	0.000499508	0.002130854	0.000954598	7
BP	GO:0031330	6/71	264/18670	0.000501334	0.002135926	0.00095687	6
BP	GO:0042572	3/71	41/18670	0.000506487	0.002138552	0.000958047	3
BP	GO:0050798	3/71	41/18670	0.000506487	0.002138552	0.000958047	3
BP	GO:0050873	3/71	41/18670	0.000506487	0.002138552	0.000958047	3
BP	GO:0150077	3/71	41/18670	0.000506487	0.002138552	0.000958047	3
BP	GO:1900117	3/71	41/18670	0.000506487	0.002138552	0.000958047	3
BP	GO:1903053	3/71	41/18670	0.000506487	0.002138552	0.000958047	3
BP	GO:0043255	4/71	97/18670	0.000509604	0.002138552	0.000958047	4
BP	GO:0044773	4/71	97/18670	0.000509604	0.002138552	0.000958047	4
BP	GO:0051591	4/71	97/18670	0.000509604	0.002138552	0.000958047	4
BP	GO:1902882	4/71	97/18670	0.000509604	0.002138552	0.000958047	4
BP	GO:1990868	4/71	97/18670	0.000509604	0.002138552	0.000958047	4
BP	GO:1990869	4/71	97/18670	0.000509604	0.002138552	0.000958047	4
BP	GO:0010469	5/71	173/18670	0.000512241	0.002146932	0.000961801	5
BP	GO:0048880	7/71	371/18670	0.00051583	0.002159272	0.000967329	7
BP	GO:0072331	6/71	267/18670	0.000532083	0.002224532	0.000996565	6
BP	GO:0046165	5/71	175/18670	0.000539737	0.002253722	0.001009641	5
BP	GO:0022898	6/71	268/18670	0.000542653	0.002260117	0.001012506	6
BP	GO:0050714	6/71	268/18670	0.000542653	0.002260117	0.001012506	6
BP	GO:0010907	3/71	42/18670	0.000543965	0.002260117	0.001012506	3
BP	GO:0042149	3/71	42/18670	0.000543965	0.002260117	0.001012506	3
BP	GO:0019395	4/71	99/18670	0.000550508	0.002284473	0.001023417	4
BP	GO:0050796	5/71	176/18670	0.000553894	0.002292849	0.00102717	5
BP	GO:0051897	5/71	176/18670	0.000553894	0.002292849	0.00102717	5
BP	GO:0045165	6/71	270/18670	0.000564283	0.002332972	0.001045144	6
BP	GO:0001959	5/71	177/18670	0.000568329	0.002346807	0.001051342	5
BP	GO:0010657	4/71	100/18670	0.000571812	0.002358284	0.001056484	4
BP	GO:1901617	6/71	271/18670	0.000575346	0.002369946	0.001061709	6
BP	GO:0010950	5/71	178/18670	0.000583045	0.002387523	0.001069583	5
BP	GO:2001257	5/71	178/18670	0.000583045	0.002387523	0.001069583	5
BP	GO:0032459	3/71	43/18670	0.000583174	0.002387523	0.001069583	3
BP	GO:0032881	3/71	43/18670	0.000583174	0.002387523	0.001069583	3
BP	GO:1904646	3/71	43/18670	0.000583174	0.002387523	0.001069583	3
BP	GO:0032611	4/71	101/18670	0.000593695	0.002424677	0.001086227	4
BP	GO:0034440	4/71	101/18670	0.000593695	0.002424677	0.001086227	4
BP	GO:0042176	7/71	381/18670	0.000603946	0.002463542	0.001103638	7
BP	GO:0002791	8/71	500/18670	0.00060614	0.002469485	0.001106301	8
BP	GO:0000079	4/71	102/18670	0.000616167	0.002504252	0.001121876	4
BP	GO:0098869	4/71	102/18670	0.000616167	0.002504252	0.001121876	4
BP	GO:0014002	3/71	44/18670	0.000624145	0.002521394	0.001129556	3
BP	GO:0030225	3/71	44/18670	0.000624145	0.002521394	0.001129556	3
BP	GO:0032965	3/71	44/18670	0.000624145	0.002521394	0.001129556	3
BP	GO:0034105	3/71	44/18670	0.000624145	0.002521394	0.001129556	3
BP	GO:0070266	3/71	44/18670	0.000624145	0.002521394	0.001129556	3
BP	GO:0033483	2/71	10/18670	0.000629129	0.002535421	0.001135839	2
BP	GO:1903800	2/71	10/18670	0.000629129	0.002535421	0.001135839	2
BP	GO:0042692	7/71	385/18670	0.000642382	0.002585721	0.001158373	7
BP	GO:0001523	4/71	104/18670	0.000662912	0.002661968	0.001192531	4
BP	GO:0030593	4/71	104/18670	0.000662912	0.002661968	0.001192531	4
BP	GO:0032570	3/71	45/18670	0.00066691	0.002668437	0.001195429	3

BP	GO:0042771	3/71	45/18670	0.00066691	0.002668437	0.001195429	3
BP	GO:0048538	3/71	45/18670	0.00066691	0.002668437	0.001195429	3
BP	GO:0043401	5/71	184/18670	0.00067744	0.002704113	0.001211411	5
BP	GO:0048639	5/71	184/18670	0.00067744	0.002704113	0.001211411	5
BP	GO:0009152	6/71	280/18670	0.000682699	0.002718634	0.001217917	6
BP	GO:0030522	6/71	280/18670	0.000682699	0.002718634	0.001217917	6
BP	GO:0007009	4/71	105/18670	0.000687203	0.002733323	0.001224497	4
BP	GO:0014706	7/71	390/18670	0.000693137	0.002753661	0.001233608	7
BP	GO:0046328	5/71	185/18670	0.000694222	0.002754706	0.001234076	5
BP	GO:0010883	3/71	46/18670	0.000711501	0.002792668	0.001251083	3
BP	GO:0022602	3/71	46/18670	0.000711501	0.002792668	0.001251083	3
BP	GO:0030810	3/71	46/18670	0.000711501	0.002792668	0.001251083	3
BP	GO:0033628	3/71	46/18670	0.000711501	0.002792668	0.001251083	3
BP	GO:0060443	3/71	46/18670	0.000711501	0.002792668	0.001251083	3
BP	GO:0060711	3/71	46/18670	0.000711501	0.002792668	0.001251083	3
BP	GO:1900373	3/71	46/18670	0.000711501	0.002792668	0.001251083	3
BP	GO:1904036	3/71	46/18670	0.000711501	0.002792668	0.001251083	3
BP	GO:1901989	4/71	106/18670	0.000712118	0.002792668	0.001251083	4
BP	GO:1904029	4/71	106/18670	0.000712118	0.002792668	0.001251083	4
BP	GO:0031647	6/71	284/18670	0.000735126	0.002879528	0.001289995	6
BP	GO:0030330	4/71	107/18670	0.000737666	0.002882744	0.001291436	4
BP	GO:0062014	4/71	107/18670	0.000737666	0.002882744	0.001291436	4
BP	GO:0030574	3/71	47/18670	0.000757949	0.00294605	0.001319797	3
BP	GO:0070849	3/71	47/18670	0.000757949	0.00294605	0.001319797	3
BP	GO:0051403	6/71	286/18670	0.000762482	0.00294605	0.001319797	6
BP	GO:0071156	4/71	108/18670	0.000763856	0.00294605	0.001319797	4
BP	GO:1904659	4/71	108/18670	0.000763856	0.00294605	0.001319797	4
BP	GO:0019362	5/71	189/18670	0.000764485	0.00294605	0.001319797	5
BP	GO:0046496	5/71	189/18670	0.000764485	0.00294605	0.001319797	5
BP	GO:0031652	2/71	11/18670	0.000767045	0.00294605	0.001319797	2
BP	GO:0033629	2/71	11/18670	0.000767045	0.00294605	0.001319797	2
BP	GO:0043619	2/71	11/18670	0.000767045	0.00294605	0.001319797	2
BP	GO:0051974	2/71	11/18670	0.000767045	0.00294605	0.001319797	2
BP	GO:0072584	2/71	11/18670	0.000767045	0.00294605	0.001319797	2
BP	GO:0072683	2/71	11/18670	0.000767045	0.00294605	0.001319797	2
BP	GO:1990440	2/71	11/18670	0.000767045	0.00294605	0.001319797	2
BP	GO:2001269	2/71	11/18670	0.000767045	0.00294605	0.001319797	2
BP	GO:0051604	7/71	397/18670	0.000769497	0.002952087	0.001322501	7
BP	GO:0060759	5/71	190/18670	0.000782852	0.00299989	0.001343916	5
BP	GO:0002793	6/71	288/18670	0.000790621	0.003026201	0.001355703	6
BP	GO:1903532	7/71	399/18670	0.000792496	0.00302992	0.001357369	7
BP	GO:0006939	4/71	110/18670	0.000818199	0.003121071	0.001398204	4
BP	GO:0033559	4/71	110/18670	0.000818199	0.003121071	0.001398204	4
BP	GO:0072676	4/71	111/18670	0.000846369	0.00322486	0.0014447	4
BP	GO:0090287	6/71	292/18670	0.000849306	0.003232375	0.001448066	6
BP	GO:0002686	3/71	49/18670	0.000856536	0.003237843	0.001450516	3
BP	GO:0010712	3/71	49/18670	0.000856536	0.003237843	0.001450516	3
BP	GO:0030857	3/71	49/18670	0.000856536	0.003237843	0.001450516	3
BP	GO:0048546	3/71	49/18670	0.000856536	0.003237843	0.001450516	3
BP	GO:1904707	3/71	49/18670	0.000856536	0.003237843	0.001450516	3
BP	GO:1990090	3/71	49/18670	0.000856536	0.003237843	0.001450516	3
BP	GO:0009260	6/71	293/18670	0.00086449	0.003261656	0.001461184	6
BP	GO:0043900	7/71	405/18670	0.000864782	0.003261656	0.001461184	7
BP	GO:0002065	4/71	112/18670	0.000875218	0.003286232	0.001472194	4
BP	GO:0008645	4/71	112/18670	0.000875218	0.003286232	0.001472194	4
BP	GO:0032411	4/71	112/18670	0.000875218	0.003286232	0.001472194	4
BP	GO:0051817	4/71	112/18670	0.000875218	0.003286232	0.001472194	4
BP	GO:0072524	5/71	195/18670	0.000879683	0.003299303	0.001478049	5
BP	GO:0016236	6/71	295/18670	0.000895486	0.003354822	0.001502922	6
BP	GO:0006694	5/71	196/18670	0.000900074	0.003364491	0.001507253	5
BP	GO:0071897	5/71	196/18670	0.000900074	0.003364491	0.001507253	5
BP	GO:0046620	4/71	113/18670	0.000904753	0.003374458	0.001511718	4
BP	GO:0048640	4/71	113/18670	0.000904753	0.003374458	0.001511718	4

BP	GO:1900087	3/71	50/18670	0.000908736	0.003383182	0.001515627	3
BP	GO:0006983	2/71	12/18670	0.000918191	0.003383182	0.001515627	2
BP	GO:0010889	2/71	12/18670	0.000918191	0.003383182	0.001515627	2
BP	GO:0031392	2/71	12/18670	0.000918191	0.003383182	0.001515627	2
BP	GO:0042368	2/71	12/18670	0.000918191	0.003383182	0.001515627	2
BP	GO:0051549	2/71	12/18670	0.000918191	0.003383182	0.001515627	2
BP	GO:0070243	2/71	12/18670	0.000918191	0.003383182	0.001515627	2
BP	GO:0070486	2/71	12/18670	0.000918191	0.003383182	0.001515627	2
BP	GO:0097284	2/71	12/18670	0.000918191	0.003383182	0.001515627	2
BP	GO:1900402	2/71	12/18670	0.000918191	0.003383182	0.001515627	2
BP	GO:1903894	2/71	12/18670	0.000918191	0.003383182	0.001515627	2
BP	GO:1903522	6/71	297/18670	0.000927335	0.003413121	0.001529039	6
BP	GO:0015749	4/71	114/18670	0.000934985	0.003433738	0.001538275	4
BP	GO:0042752	4/71	114/18670	0.000934985	0.003433738	0.001538275	4
BP	GO:0019369	3/71	51/18670	0.000962912	0.003528571	0.001580759	3
BP	GO:0031103	3/71	51/18670	0.000962912	0.003528571	0.001580759	3
BP	GO:0006096	4/71	115/18670	0.000965921	0.003531878	0.001582241	4
BP	GO:0032612	4/71	115/18670	0.000965921	0.003531878	0.001582241	4
BP	GO:0046390	6/71	300/18670	0.000976738	0.003567542	0.001598217	6
BP	GO:0006757	4/71	116/18670	0.000997571	0.003631765	0.001626989	4
BP	GO:0021987	4/71	116/18670	0.000997571	0.003631765	0.001626989	4
BP	GO:0034219	4/71	116/18670	0.000997571	0.003631765	0.001626989	4
BP	GO:0032731	3/71	52/18670	0.001019093	0.003702078	0.001658488	3
BP	GO:1990089	3/71	52/18670	0.001019093	0.003702078	0.001658488	3
BP	GO:0009746	5/71	202/18670	0.001029913	0.003733409	0.001672524	5
BP	GO:0010508	4/71	117/18670	0.001029944	0.003733409	0.001672524	4
BP	GO:1990266	4/71	118/18670	0.001063049	0.003849249	0.001724419	4
BP	GO:0031396	5/71	204/18670	0.001076139	0.003852991	0.001726095	5
BP	GO:0031638	3/71	53/18670	0.001077308	0.003852991	0.001726095	3
BP	GO:0032964	3/71	53/18670	0.001077308	0.003852991	0.001726095	3
BP	GO:0045661	3/71	53/18670	0.001077308	0.003852991	0.001726095	3
BP	GO:0070228	3/71	53/18670	0.001077308	0.003852991	0.001726095	3
BP	GO:0010745	2/71	13/18670	0.001082469	0.003852991	0.001726095	2
BP	GO:0010870	2/71	13/18670	0.001082469	0.003852991	0.001726095	2
BP	GO:0030656	2/71	13/18670	0.001082469	0.003852991	0.001726095	2
BP	GO:0031650	2/71	13/18670	0.001082469	0.003852991	0.001726095	2
BP	GO:0031953	2/71	13/18670	0.001082469	0.003852991	0.001726095	2
BP	GO:0042178	2/71	13/18670	0.001082469	0.003852991	0.001726095	2
BP	GO:0043568	2/71	13/18670	0.001082469	0.003852991	0.001726095	2
BP	GO:0045086	2/71	13/18670	0.001082469	0.003852991	0.001726095	2
BP	GO:0060576	2/71	13/18670	0.001082469	0.003852991	0.001726095	2
BP	GO:0061029	2/71	13/18670	0.001082469	0.003852991	0.001726095	2
BP	GO:1902894	2/71	13/18670	0.001082469	0.003852991	0.001726095	2
BP	GO:0007173	4/71	119/18670	0.001096895	0.003891947	0.001743547	4
BP	GO:0042866	4/71	119/18670	0.001096895	0.003891947	0.001743547	4
BP	GO:0051193	4/71	119/18670	0.001096895	0.003891947	0.001743547	4
BP	GO:0032386	7/71	423/18670	0.001113403	0.003946343	0.001767916	7
BP	GO:0030199	3/71	54/18670	0.001137585	0.004015075	0.001798707	3
BP	GO:0032655	3/71	54/18670	0.001137585	0.004015075	0.001798707	3
BP	GO:0043392	3/71	54/18670	0.001137585	0.004015075	0.001798707	3
BP	GO:0050994	3/71	54/18670	0.001137585	0.004015075	0.001798707	3
BP	GO:0006733	5/71	207/18670	0.001148345	0.004040295	0.001810005	5
BP	GO:0030073	5/71	207/18670	0.001148345	0.004040295	0.001810005	5
BP	GO:0034284	5/71	207/18670	0.001148345	0.004040295	0.001810005	5
BP	GO:0030968	4/71	121/18670	0.001166846	0.004096791	0.001835315	4
BP	GO:0046718	4/71	121/18670	0.001166846	0.004096791	0.001835315	4
BP	GO:1990778	6/71	311/18670	0.001175456	0.004122703	0.001846923	6
BP	GO:0010823	3/71	55/18670	0.001199951	0.004192885	0.001878364	3
BP	GO:0042306	3/71	55/18670	0.001199951	0.004192885	0.001878364	3
BP	GO:0019079	4/71	122/18670	0.001202969	0.004192885	0.001878364	4
BP	GO:0034101	4/71	122/18670	0.001202969	0.004192885	0.001878364	4
BP	GO:0043500	4/71	122/18670	0.001202969	0.004192885	0.001878364	4
BP	GO:1903578	4/71	122/18670	0.001202969	0.004192885	0.001878364	4

BP	GO:0043010	6/71	314/18670	0.001234687	0.004298967	0.001925888	6
BP	GO:0071621	4/71	123/18670	0.001239869	0.004312531	0.001931964	4
BP	GO:0030213	2/71	14/18670	0.001259777	0.004350189	0.001948835	2
BP	GO:0035810	2/71	14/18670	0.001259777	0.004350189	0.001948835	2
BP	GO:0036295	2/71	14/18670	0.001259777	0.004350189	0.001948835	2
BP	GO:0043374	2/71	14/18670	0.001259777	0.004350189	0.001948835	2
BP	GO:0045651	2/71	14/18670	0.001259777	0.004350189	0.001948835	2
BP	GO:0051547	2/71	14/18670	0.001259777	0.004350189	0.001948835	2
BP	GO:2001279	2/71	14/18670	0.001259777	0.004350189	0.001948835	2
BP	GO:0032615	3/71	56/18670	0.001264435	0.004352824	0.001950015	3
BP	GO:1903078	3/71	56/18670	0.001264435	0.004352824	0.001950015	3
BP	GO:1903202	3/71	56/18670	0.001264435	0.004352824	0.001950015	3
BP	GO:0014013	4/71	124/18670	0.001277554	0.004388975	0.00196621	4
BP	GO:0019218	4/71	124/18670	0.001277554	0.004388975	0.00196621	4
BP	GO:0070371	6/71	317/18670	0.001296182	0.004448413	0.001992837	6
BP	GO:0006805	4/71	125/18670	0.001316035	0.004511926	0.002021291	4
BP	GO:0050804	7/71	436/18670	0.001325606	0.004517261	0.002023681	7
BP	GO:0007254	5/71	214/18670	0.001330756	0.004517261	0.002023681	5
BP	GO:0016051	5/71	214/18670	0.001330756	0.004517261	0.002023681	5
BP	GO:0045732	5/71	214/18670	0.001330756	0.004517261	0.002023681	5
BP	GO:2001020	5/71	214/18670	0.001330756	0.004517261	0.002023681	5
BP	GO:0002066	3/71	57/18670	0.001331063	0.004517261	0.002023681	3
BP	GO:0042743	3/71	57/18670	0.001331063	0.004517261	0.002023681	3
BP	GO:0045981	3/71	57/18670	0.001331063	0.004517261	0.002023681	3
BP	GO:1900408	3/71	57/18670	0.001331063	0.004517261	0.002023681	3
BP	GO:1900544	3/71	57/18670	0.001331063	0.004517261	0.002023681	3
BP	GO:0099177	7/71	437/18670	0.001343158	0.004553698	0.002040004	7
BP	GO:0046031	4/71	126/18670	0.001355319	0.004590287	0.002056395	4
BP	GO:0032388	5/71	215/18670	0.001358464	0.004596298	0.002059088	5
BP	GO:0071375	6/71	321/18670	0.001381797	0.004670528	0.002092343	6
BP	GO:0032869	5/71	216/18670	0.001386597	0.004682034	0.002097497	5
BP	GO:0071385	3/71	58/18670	0.001399861	0.004712584	0.002111183	3
BP	GO:0098930	3/71	58/18670	0.001399861	0.004712584	0.002111183	3
BP	GO:1904589	3/71	58/18670	0.001399861	0.004712584	0.002111183	3
BP	GO:0034763	4/71	128/18670	0.001436334	0.004828118	0.002162941	4
BP	GO:0030812	2/71	15/18670	0.001450019	0.004828118	0.002162941	2
BP	GO:0035635	2/71	15/18670	0.001450019	0.004828118	0.002162941	2
BP	GO:0042362	2/71	15/18670	0.001450019	0.004828118	0.002162941	2
BP	GO:0046321	2/71	15/18670	0.001450019	0.004828118	0.002162941	2
BP	GO:0048070	2/71	15/18670	0.001450019	0.004828118	0.002162941	2
BP	GO:0051044	2/71	15/18670	0.001450019	0.004828118	0.002162941	2
BP	GO:0051198	2/71	15/18670	0.001450019	0.004828118	0.002162941	2
BP	GO:0051770	2/71	15/18670	0.001450019	0.004828118	0.002162941	2
BP	GO:1900119	2/71	15/18670	0.001450019	0.004828118	0.002162941	2
BP	GO:2000402	2/71	15/18670	0.001450019	0.004828118	0.002162941	2
BP	GO:0031102	3/71	59/18670	0.001470856	0.004882951	0.002187505	3
BP	GO:0032732	3/71	59/18670	0.001470856	0.004882951	0.002187505	3
BP	GO:1902883	3/71	59/18670	0.001470856	0.004882951	0.002187505	3
BP	GO:0048762	5/71	219/18670	0.001473581	0.004887158	0.00218939	5
BP	GO:0034754	4/71	129/18670	0.001478084	0.004892413	0.002191744	4
BP	GO:1903038	4/71	129/18670	0.001478084	0.004892413	0.002191744	4
BP	GO:0044282	7/71	445/18670	0.001490225	0.004927735	0.002207568	7
BP	GO:1902808	3/71	60/18670	0.001544074	0.005100767	0.002285085	3
BP	GO:0046887	4/71	131/18670	0.00156411	0.005158171	0.002310801	4
BP	GO:0043903	5/71	222/18670	0.001564527	0.005158171	0.002310801	5
BP	GO:0042476	4/71	132/18670	0.001608404	0.005297623	0.002373274	4
BP	GO:0006879	3/71	61/18670	0.001619539	0.005308225	0.002378024	3
BP	GO:0030888	3/71	61/18670	0.001619539	0.005308225	0.002378024	3
BP	GO:0032370	3/71	61/18670	0.001619539	0.005308225	0.002378024	3
BP	GO:0045123	3/71	61/18670	0.001619539	0.005308225	0.002378024	3
BP	GO:0071384	3/71	61/18670	0.001619539	0.005308225	0.002378024	3
BP	GO:0002070	2/71	16/18670	0.001653096	0.005381388	0.0024108	2
BP	GO:0019372	2/71	16/18670	0.001653096	0.005381388	0.0024108	2

BP	GO:0043217	2/71	16/18670	0.001653096	0.005381388	0.0024108	2
BP	GO:0060965	2/71	16/18670	0.001653096	0.005381388	0.0024108	2
BP	GO:0070293	2/71	16/18670	0.001653096	0.005381388	0.0024108	2
BP	GO:1902001	2/71	16/18670	0.001653096	0.005381388	0.0024108	2
BP	GO:1902004	2/71	16/18670	0.001653096	0.005381388	0.0024108	2
BP	GO:0007160	5/71	225/18670	0.001659545	0.005391914	0.002415515	5
BP	GO:0033157	5/71	225/18670	0.001659545	0.005391914	0.002415515	5
BP	GO:0015849	6/71	333/18670	0.001664777	0.005393234	0.002416107	6
BP	GO:0016042	6/71	333/18670	0.001664777	0.005393234	0.002416107	6
BP	GO:0046942	6/71	333/18670	0.001664777	0.005393234	0.002416107	6
BP	GO:0010676	3/71	62/18670	0.001697278	0.005484855	0.002457152	3
BP	GO:0045453	3/71	62/18670	0.001697278	0.005484855	0.002457152	3
BP	GO:0006165	4/71	134/18670	0.001699602	0.005484855	0.002457152	4
BP	GO:0006766	4/71	134/18670	0.001699602	0.005484855	0.002457152	4
BP	GO:0001889	4/71	135/18670	0.001746522	0.005630854	0.002522557	4
BP	GO:0043405	6/71	337/18670	0.001768284	0.005695538	0.002551535	6
BP	GO:1904377	3/71	63/18670	0.001777314	0.005713647	0.002559648	3
BP	GO:2000401	3/71	63/18670	0.001777314	0.005713647	0.002559648	3
BP	GO:0007292	4/71	136/18670	0.001794335	0.005757326	0.002579216	4
BP	GO:0046939	4/71	136/18670	0.001794335	0.005757326	0.002579216	4
BP	GO:0023061	7/71	462/18670	0.001844518	0.005911748	0.002648395	7
BP	GO:1900449	3/71	64/18670	0.001859672	0.005911748	0.002648395	3
BP	GO:1903320	5/71	231/18670	0.001862233	0.005911748	0.002648395	5
BP	GO:0002683	7/71	463/18670	0.001867235	0.005911748	0.002648395	7
BP	GO:0006089	2/71	17/18670	0.001868909	0.005911748	0.002648395	2
BP	GO:0006978	2/71	17/18670	0.001868909	0.005911748	0.002648395	2
BP	GO:0010224	2/71	17/18670	0.001868909	0.005911748	0.002648395	2
BP	GO:0032966	2/71	17/18670	0.001868909	0.005911748	0.002648395	2
BP	GO:0045091	2/71	17/18670	0.001868909	0.005911748	0.002648395	2
BP	GO:0045725	2/71	17/18670	0.001868909	0.005911748	0.002648395	2
BP	GO:0050665	2/71	17/18670	0.001868909	0.005911748	0.002648395	2
BP	GO:0060644	2/71	17/18670	0.001868909	0.005911748	0.002648395	2
BP	GO:0061298	2/71	17/18670	0.001868909	0.005911748	0.002648395	2
BP	GO:0071850	2/71	17/18670	0.001868909	0.005911748	0.002648395	2
BP	GO:2000811	2/71	17/18670	0.001868909	0.005911748	0.002648395	2
BP	GO:0009135	4/71	138/18670	0.001892676	0.005958818	0.002669482	4
BP	GO:0009179	4/71	138/18670	0.001892676	0.005958818	0.002669482	4
BP	GO:0009308	4/71	138/18670	0.001892676	0.005958818	0.002669482	4
BP	GO:0046631	4/71	138/18670	0.001892676	0.005958818	0.002669482	4
BP	GO:0061008	4/71	138/18670	0.001892676	0.005958818	0.002669482	4
BP	GO:0033044	6/71	342/18670	0.001904472	0.005990333	0.0026836	6
BP	GO:0051346	7/71	466/18670	0.001936696	0.006085981	0.002726449	7
BP	GO:0072678	3/71	65/18670	0.001944377	0.006104396	0.002734699	3
BP	GO:0009185	4/71	140/18670	0.001994693	0.006250661	0.002800224	4
BP	GO:0034620	4/71	140/18670	0.001994693	0.006250661	0.002800224	4
BP	GO:0009755	5/71	235/18670	0.002007083	0.006283613	0.002814986	5
BP	GO:0097530	4/71	141/18670	0.002047103	0.006402927	0.002868437	4
BP	GO:0043122	5/71	237/18670	0.002082524	0.00647556	0.002900976	5
BP	GO:0010713	2/71	18/18670	0.002097363	0.00647556	0.002900976	2
BP	GO:0031998	2/71	18/18670	0.002097363	0.00647556	0.002900976	2
BP	GO:0035994	2/71	18/18670	0.002097363	0.00647556	0.002900976	2
BP	GO:0039692	2/71	18/18670	0.002097363	0.00647556	0.002900976	2
BP	GO:0042772	2/71	18/18670	0.002097363	0.00647556	0.002900976	2
BP	GO:0045780	2/71	18/18670	0.002097363	0.00647556	0.002900976	2
BP	GO:0046852	2/71	18/18670	0.002097363	0.00647556	0.002900976	2
BP	GO:0060749	2/71	18/18670	0.002097363	0.00647556	0.002900976	2
BP	GO:0061377	2/71	18/18670	0.002097363	0.00647556	0.002900976	2
BP	GO:0070230	2/71	18/18670	0.002097363	0.00647556	0.002900976	2
BP	GO:0070875	2/71	18/18670	0.002097363	0.00647556	0.002900976	2
BP	GO:0071318	2/71	18/18670	0.002097363	0.00647556	0.002900976	2
BP	GO:0090026	2/71	18/18670	0.002097363	0.00647556	0.002900976	2
BP	GO:0030258	5/71	238/18670	0.002121013	0.006536541	0.002928295	5
BP	GO:0051607	5/71	238/18670	0.002121013	0.006536541	0.002928295	5

BP	GO:0050769	7/71	474/18670	0.002131783	0.006563699	0.002940461	7
BP	GO:0044344	4/71	143/18670	0.002154769	0.006628387	0.002969441	4
BP	GO:0016239	3/71	68/18670	0.0022128	0.006782009	0.003038262	3
BP	GO:0035924	3/71	68/18670	0.0022128	0.006782009	0.003038262	3
BP	GO:0050891	3/71	68/18670	0.0022128	0.006782009	0.003038262	3
BP	GO:0050918	3/71	68/18670	0.0022128	0.006782009	0.003038262	3
BP	GO:0042593	5/71	241/18670	0.002239603	0.006857889	0.003072255	5
BP	GO:0000077	4/71	145/18670	0.002266288	0.006920641	0.003100367	4
BP	GO:0007605	4/71	145/18670	0.002266288	0.006920641	0.003100367	4
BP	GO:0007612	4/71	145/18670	0.002266288	0.006920641	0.003100367	4
BP	GO:0033500	5/71	242/18670	0.002280188	0.006956751	0.003116544	5
BP	GO:0008088	3/71	69/18670	0.002307121	0.007026136	0.003147628	3
BP	GO:0015909	3/71	69/18670	0.002307121	0.007026136	0.003147628	3
BP	GO:0003085	2/71	19/18670	0.002338361	0.00705083	0.00315869	2
BP	GO:0030949	2/71	19/18670	0.002338361	0.00705083	0.00315869	2
BP	GO:0032930	2/71	19/18670	0.002338361	0.00705083	0.00315869	2
BP	GO:0034138	2/71	19/18670	0.002338361	0.00705083	0.00315869	2
BP	GO:0045076	2/71	19/18670	0.002338361	0.00705083	0.00315869	2
BP	GO:0060149	2/71	19/18670	0.002338361	0.00705083	0.00315869	2
BP	GO:0060252	2/71	19/18670	0.002338361	0.00705083	0.00315869	2
BP	GO:0060716	2/71	19/18670	0.002338361	0.00705083	0.00315869	2
BP	GO:0060967	2/71	19/18670	0.002338361	0.00705083	0.00315869	2
BP	GO:0090201	2/71	19/18670	0.002338361	0.00705083	0.00315869	2
BP	GO:1903798	2/71	19/18670	0.002338361	0.00705083	0.00315869	2
BP	GO:0071236	4/71	147/18670	0.002381729	0.007175145	0.003214382	4
BP	GO:0033077	3/71	70/18670	0.002403902	0.007222476	0.003235586	3
BP	GO:0051966	3/71	70/18670	0.002403902	0.007222476	0.003235586	3
BP	GO:2001259	3/71	70/18670	0.002403902	0.007222476	0.003235586	3
BP	GO:0008643	4/71	148/18670	0.002440942	0.007327196	0.003282499	4
BP	GO:0033692	3/71	71/18670	0.002503166	0.007500549	0.003360159	3
BP	GO:1904427	3/71	71/18670	0.002503166	0.007500549	0.003360159	3
BP	GO:0046434	5/71	248/18670	0.002535061	0.00758934	0.003399937	5
BP	GO:0008203	4/71	150/18670	0.002562396	0.007650679	0.003427416	4
BP	GO:0008360	4/71	150/18670	0.002562396	0.007650679	0.003427416	4
BP	GO:0071774	4/71	150/18670	0.002562396	0.007650679	0.003427416	4
BP	GO:0002827	2/71	20/18670	0.002591806	0.007670189	0.003436156	2
BP	GO:0010042	2/71	20/18670	0.002591806	0.007670189	0.003436156	2
BP	GO:0019373	2/71	20/18670	0.002591806	0.007670189	0.003436156	2
BP	GO:0030220	2/71	20/18670	0.002591806	0.007670189	0.003436156	2
BP	GO:0034393	2/71	20/18670	0.002591806	0.007670189	0.003436156	2
BP	GO:0042535	2/71	20/18670	0.002591806	0.007670189	0.003436156	2
BP	GO:0051767	2/71	20/18670	0.002591806	0.007670189	0.003436156	2
BP	GO:0051769	2/71	20/18670	0.002591806	0.007670189	0.003436156	2
BP	GO:0097709	2/71	20/18670	0.002591806	0.007670189	0.003436156	2
BP	GO:1902993	2/71	20/18670	0.002591806	0.007670189	0.003436156	2
BP	GO:0006635	3/71	72/18670	0.002604933	0.00770224	0.003450514	3
BP	GO:0010951	5/71	250/18670	0.002624449	0.007746887	0.003470516	5
BP	GO:0016999	4/71	151/18670	0.002624654	0.007746887	0.003470516	4
BP	GO:0021537	5/71	251/18670	0.002669991	0.007873774	0.00352736	5
BP	GO:0000187	4/71	152/18670	0.002687944	0.007912797	0.003544842	4
BP	GO:0016202	4/71	152/18670	0.002687944	0.007912797	0.003544842	4
BP	GO:0061844	3/71	73/18670	0.002709225	0.007968449	0.003569773	3
BP	GO:0050729	4/71	153/18670	0.002752275	0.008087973	0.003623318	4
BP	GO:0014015	3/71	74/18670	0.002816063	0.008243976	0.003693206	3
BP	GO:0030104	3/71	74/18670	0.002816063	0.008243976	0.003693206	3
BP	GO:0032507	3/71	74/18670	0.002816063	0.008243976	0.003693206	3
BP	GO:0099601	3/71	74/18670	0.002816063	0.008243976	0.003693206	3
BP	GO:0006090	4/71	154/18670	0.002817655	0.008243976	0.003693206	4
BP	GO:0010893	2/71	21/18670	0.002857603	0.008288532	0.003713166	2
BP	GO:0017000	2/71	21/18670	0.002857603	0.008288532	0.003713166	2
BP	GO:0030728	2/71	21/18670	0.002857603	0.008288532	0.003713166	2
BP	GO:0036344	2/71	21/18670	0.002857603	0.008288532	0.003713166	2
BP	GO:0046716	2/71	21/18670	0.002857603	0.008288532	0.003713166	2

BP	GO:0046827	2/71	21/18670	0.002857603	0.008288532	0.003713166	2
BP	GO:0051412	2/71	21/18670	0.002857603	0.008288532	0.003713166	2
BP	GO:0070584	2/71	21/18670	0.002857603	0.008288532	0.003713166	2
BP	GO:0070920	2/71	21/18670	0.002857603	0.008288532	0.003713166	2
BP	GO:0071498	2/71	21/18670	0.002857603	0.008288532	0.003713166	2
BP	GO:1901861	4/71	155/18670	0.002884093	0.008350917	0.003741115	4
BP	GO:1902652	4/71	155/18670	0.002884093	0.008350917	0.003741115	4
BP	GO:0090596	5/71	256/18670	0.002906365	0.008408146	0.003766752	5
BP	GO:0007422	3/71	75/18670	0.002925467	0.008456113	0.003788241	3
BP	GO:0048634	4/71	156/18670	0.002951596	0.008524292	0.003818784	4
BP	GO:0043406	5/71	258/18670	0.003005033	0.008671148	0.003884574	5
BP	GO:0001937	3/71	76/18670	0.003037458	0.008757176	0.003923114	3
BP	GO:0003007	5/71	259/18670	0.003055266	0.008800951	0.003942724	5
BP	GO:0009132	4/71	158/18670	0.003089836	0.008892893	0.003983914	4
BP	GO:0032412	5/71	260/18670	0.003106104	0.008924394	0.003998025	5
BP	GO:0072659	5/71	260/18670	0.003106104	0.008924394	0.003998025	5
BP	GO:0032469	2/71	22/18670	0.003135657	0.008940357	0.004005176	2
BP	GO:0032928	2/71	22/18670	0.003135657	0.008940357	0.004005176	2
BP	GO:0042094	2/71	22/18670	0.003135657	0.008940357	0.004005176	2
BP	GO:0045649	2/71	22/18670	0.003135657	0.008940357	0.004005176	2
BP	GO:0045723	2/71	22/18670	0.003135657	0.008940357	0.004005176	2
BP	GO:0051000	2/71	22/18670	0.003135657	0.008940357	0.004005176	2
BP	GO:0051195	2/71	22/18670	0.003135657	0.008940357	0.004005176	2
BP	GO:0060575	2/71	22/18670	0.003135657	0.008940357	0.004005176	2
BP	GO:0090312	2/71	22/18670	0.003135657	0.008940357	0.004005176	2
BP	GO:0002673	4/71	159/18670	0.003160589	0.009003785	0.004033591	4
BP	GO:0010466	5/71	262/18670	0.003209613	0.00913568	0.004092679	5
BP	GO:0006754	4/71	160/18670	0.003232441	0.009185064	0.004114802	4
BP	GO:0007519	4/71	160/18670	0.003232441	0.009185064	0.004114802	4
BP	GO:0030900	6/71	381/18670	0.003257928	0.009249647	0.004143735	6
BP	GO:0000271	3/71	78/18670	0.003269277	0.009266174	0.004151139	3
BP	GO:0031016	3/71	78/18670	0.003269277	0.009266174	0.004151139	3
BP	GO:0035967	4/71	161/18670	0.003305401	0.009360651	0.004193463	4
BP	GO:0140014	5/71	264/18670	0.003315592	0.009381585	0.004202842	5
BP	GO:0001570	3/71	79/18670	0.003389144	0.009573545	0.004288838	3
BP	GO:0055072	3/71	79/18670	0.003389144	0.009573545	0.004288838	3
BP	GO:0036037	2/71	23/18670	0.003425875	0.00963671	0.004317135	2
BP	GO:0045821	2/71	23/18670	0.003425875	0.00963671	0.004317135	2
BP	GO:0050995	2/71	23/18670	0.003425875	0.00963671	0.004317135	2
BP	GO:0051043	2/71	23/18670	0.003425875	0.00963671	0.004317135	2
BP	GO:2000637	2/71	23/18670	0.003425875	0.00963671	0.004317135	2
BP	GO:0006633	4/71	164/18670	0.003531012	0.009915815	0.00444217	4
BP	GO:0007088	4/71	164/18670	0.003531012	0.009915815	0.00444217	4
BP	GO:0007249	5/71	269/18670	0.003591566	0.010077423	0.004514569	5
BP	GO:0050954	4/71	165/18670	0.003608486	0.010116433	0.004532045	4
BP	GO:0030512	3/71	81/18670	0.003636887	0.010187537	0.004563899	3
BP	GO:0009110	2/71	24/18670	0.003728161	0.010391126	0.004655104	2
BP	GO:0042104	2/71	24/18670	0.003728161	0.010391126	0.004655104	2
BP	GO:0050996	2/71	24/18670	0.003728161	0.010391126	0.004655104	2
BP	GO:0060148	2/71	24/18670	0.003728161	0.010391126	0.004655104	2
BP	GO:0071677	2/71	24/18670	0.003728161	0.010391126	0.004655104	2
BP	GO:2000209	2/71	24/18670	0.003728161	0.010391126	0.004655104	2
BP	GO:0051279	3/71	82/18670	0.0037648	0.010472954	0.004691762	3
BP	GO:1905954	3/71	82/18670	0.0037648	0.010472954	0.004691762	3
BP	GO:0035051	4/71	167/18670	0.00376689	0.010472954	0.004691762	4
BP	GO:0051302	4/71	168/18670	0.003847836	0.010689141	0.004788612	4
BP	GO:0042509	3/71	83/18670	0.003895431	0.010785616	0.004831831	3
BP	GO:0071277	3/71	83/18670	0.003895431	0.010785616	0.004831831	3
BP	GO:1903845	3/71	83/18670	0.003895431	0.010785616	0.004831831	3
BP	GO:2000106	3/71	83/18670	0.003895431	0.010785616	0.004831831	3
BP	GO:0030307	4/71	169/18670	0.003929955	0.010863263	0.004866616	4
BP	GO:0060538	4/71	169/18670	0.003929955	0.010863263	0.004866616	4
BP	GO:0016054	5/71	275/18670	0.003944125	0.010884486	0.004876124	5

BP	GO:0046395	5/71	275/18670	0.003944125	0.010884486	0.004876124	5
BP	GO:0009404	2/71	25/18670	0.004042423	0.011028678	0.00494072	2
BP	GO:0019054	2/71	25/18670	0.004042423	0.011028678	0.00494072	2
BP	GO:0030813	2/71	25/18670	0.004042423	0.011028678	0.00494072	2
BP	GO:0031639	2/71	25/18670	0.004042423	0.011028678	0.00494072	2
BP	GO:0045662	2/71	25/18670	0.004042423	0.011028678	0.00494072	2
BP	GO:0045672	2/71	25/18670	0.004042423	0.011028678	0.00494072	2
BP	GO:0050927	2/71	25/18670	0.004042423	0.011028678	0.00494072	2
BP	GO:0051197	2/71	25/18670	0.004042423	0.011028678	0.00494072	2
BP	GO:0060330	2/71	25/18670	0.004042423	0.011028678	0.00494072	2
BP	GO:0060334	2/71	25/18670	0.004042423	0.011028678	0.00494072	2
BP	GO:0090025	2/71	25/18670	0.004042423	0.011028678	0.00494072	2
BP	GO:1900101	2/71	25/18670	0.004042423	0.011028678	0.00494072	2
BP	GO:2000353	2/71	25/18670	0.004042423	0.011028678	0.00494072	2
BP	GO:2000679	2/71	25/18670	0.004042423	0.011028678	0.00494072	2
BP	GO:0006937	4/71	171/18670	0.004097744	0.011161444	0.005000198	4
BP	GO:0009206	4/71	171/18670	0.004097744	0.011161444	0.005000198	4
BP	GO:0002028	3/71	85/18670	0.004164918	0.011316832	0.00506981	3
BP	GO:0014031	3/71	85/18670	0.004164918	0.011316832	0.00506981	3
BP	GO:0034308	3/71	85/18670	0.004164918	0.011316832	0.00506981	3
BP	GO:0009145	4/71	172/18670	0.004183431	0.011357931	0.005088222	4
BP	GO:0016525	4/71	173/18670	0.004270323	0.011584462	0.005189705	4
BP	GO:0006732	6/71	403/18670	0.004284291	0.011612956	0.00520247	6
BP	GO:0007260	3/71	86/18670	0.004303808	0.011647028	0.005217734	3
BP	GO:0048013	3/71	86/18670	0.004303808	0.011647028	0.005217734	3
BP	GO:2001252	4/71	174/18670	0.004358429	0.011727629	0.005253842	4
BP	GO:0002360	2/71	26/18670	0.004368568	0.011727629	0.005253842	2
BP	GO:0006309	2/71	26/18670	0.004368568	0.011727629	0.005253842	2
BP	GO:0022011	2/71	26/18670	0.004368568	0.011727629	0.005253842	2
BP	GO:0032292	2/71	26/18670	0.004368568	0.011727629	0.005253842	2
BP	GO:0043567	2/71	26/18670	0.004368568	0.011727629	0.005253842	2
BP	GO:0050926	2/71	26/18670	0.004368568	0.011727629	0.005253842	2
BP	GO:0060544	2/71	26/18670	0.004368568	0.011727629	0.005253842	2
BP	GO:0060706	2/71	26/18670	0.004368568	0.011727629	0.005253842	2
BP	GO:1903579	2/71	26/18670	0.004368568	0.011727629	0.005253842	2
BP	GO:0045844	3/71	87/18670	0.004445485	0.011902092	0.005332	3
BP	GO:0048636	3/71	87/18670	0.004445485	0.011902092	0.005332	3
BP	GO:0051781	3/71	87/18670	0.004445485	0.011902092	0.005332	3
BP	GO:2000181	4/71	175/18670	0.004447755	0.011902092	0.005332	4
BP	GO:0000280	6/71	407/18670	0.004494021	0.01201631	0.005383168	6
BP	GO:0015980	5/71	285/18670	0.004585907	0.012233823	0.005480611	5
BP	GO:0050829	3/71	88/18670	0.004589964	0.012233823	0.005480611	3
BP	GO:0061097	3/71	88/18670	0.004589964	0.012233823	0.005480611	3
BP	GO:1901863	3/71	88/18670	0.004589964	0.012233823	0.005480611	3
BP	GO:0009201	4/71	177/18670	0.004630101	0.012331	0.005524145	4
BP	GO:0002026	2/71	27/18670	0.004706503	0.012455332	0.005579845	2
BP	GO:0002825	2/71	27/18670	0.004706503	0.012455332	0.005579845	2
BP	GO:0072378	2/71	27/18670	0.004706503	0.012455332	0.005579845	2
BP	GO:1901623	2/71	27/18670	0.004706503	0.012455332	0.005579845	2
BP	GO:1905208	2/71	27/18670	0.004706503	0.012455332	0.005579845	2
BP	GO:1905563	2/71	27/18670	0.004706503	0.012455332	0.005579845	2
BP	GO:2000144	2/71	27/18670	0.004706503	0.012455332	0.005579845	2
BP	GO:2000191	2/71	27/18670	0.004706503	0.012455332	0.005579845	2
BP	GO:0097306	3/71	89/18670	0.004737262	0.012526846	0.005611882	3
BP	GO:0032651	3/71	90/18670	0.004887396	0.012893342	0.005776068	3
BP	GO:0045778	3/71	90/18670	0.004887396	0.012893342	0.005776068	3
BP	GO:0046849	3/71	90/18670	0.004887396	0.012893342	0.005776068	3
BP	GO:0002285	4/71	181/18670	0.005009787	0.013185099	0.005906772	4
BP	GO:0022408	4/71	181/18670	0.005009787	0.013185099	0.005906772	4
BP	GO:0051147	4/71	181/18670	0.005009787	0.013185099	0.005906772	4
BP	GO:0051899	3/71	91/18670	0.005040379	0.013193173	0.005910389	3
BP	GO:0001516	2/71	28/18670	0.005056137	0.013193173	0.005910389	2
BP	GO:0003180	2/71	28/18670	0.005056137	0.013193173	0.005910389	2

BP	GO:0033137	2/71	28/18670	0.005056137	0.013193173	0.005910389	2
BP	GO:0033598	2/71	28/18670	0.005056137	0.013193173	0.005910389	2
BP	GO:0046457	2/71	28/18670	0.005056137	0.013193173	0.005910389	2
BP	GO:1900543	2/71	28/18670	0.005056137	0.013193173	0.005910389	2
BP	GO:1902003	2/71	28/18670	0.005056137	0.013193173	0.005910389	2
BP	GO:1902932	2/71	28/18670	0.005056137	0.013193173	0.005910389	2
BP	GO:1990776	2/71	28/18670	0.005056137	0.013193173	0.005910389	2
BP	GO:2000108	2/71	28/18670	0.005056137	0.013193173	0.005910389	2
BP	GO:0009166	4/71	182/18670	0.005107877	0.013317816	0.005966228	4
BP	GO:0045833	3/71	92/18670	0.005196228	0.013534811	0.006063439	3
BP	GO:0009127	4/71	183/18670	0.005207249	0.013534811	0.006063439	4
BP	GO:0009168	4/71	183/18670	0.005207249	0.013534811	0.006063439	4
BP	GO:0043123	4/71	183/18670	0.005207249	0.013534811	0.006063439	4
BP	GO:0050768	5/71	295/18670	0.005299002	0.01376263	0.006165499	5
BP	GO:0007589	3/71	93/18670	0.005354956	0.01387571	0.006216158	3
BP	GO:0035249	3/71	93/18670	0.005354956	0.01387571	0.006216158	3
BP	GO:0046634	3/71	93/18670	0.005354956	0.01387571	0.006216158	3
BP	GO:0000083	2/71	29/18670	0.005417378	0.013951206	0.006249979	2
BP	GO:0005979	2/71	29/18670	0.005417378	0.013951206	0.006249979	2
BP	GO:0007263	2/71	29/18670	0.005417378	0.013951206	0.006249979	2
BP	GO:0010962	2/71	29/18670	0.005417378	0.013951206	0.006249979	2
BP	GO:0014044	2/71	29/18670	0.005417378	0.013951206	0.006249979	2
BP	GO:0044068	2/71	29/18670	0.005417378	0.013951206	0.006249979	2
BP	GO:0045980	2/71	29/18670	0.005417378	0.013951206	0.006249979	2
BP	GO:1900027	2/71	29/18670	0.005417378	0.013951206	0.006249979	2
BP	GO:0070167	3/71	94/18670	0.00551658	0.014195773	0.006359542	3
BP	GO:1901343	4/71	187/18670	0.005617714	0.014444935	0.006471164	4
BP	GO:0045069	3/71	95/18670	0.005681113	0.014580741	0.006532003	3
BP	GO:0051651	3/71	95/18670	0.005681113	0.014580741	0.006532003	3
BP	GO:0070372	5/71	300/18670	0.005683575	0.014580741	0.006532003	5
BP	GO:0009142	4/71	188/18670	0.005723611	0.014649824	0.006562952	4
BP	GO:0051783	4/71	188/18670	0.005723611	0.014649824	0.006562952	4
BP	GO:1901292	4/71	188/18670	0.005723611	0.014649824	0.006562952	4
BP	GO:0010743	2/71	30/18670	0.005790136	0.014752528	0.006608962	2
BP	GO:0032743	2/71	30/18670	0.005790136	0.014752528	0.006608962	2
BP	GO:0042759	2/71	30/18670	0.005790136	0.014752528	0.006608962	2
BP	GO:0045940	2/71	30/18670	0.005790136	0.014752528	0.006608962	2
BP	GO:0070168	2/71	30/18670	0.005790136	0.014752528	0.006608962	2
BP	GO:0097421	2/71	30/18670	0.005790136	0.014752528	0.006608962	2
BP	GO:0090277	3/71	96/18670	0.005848568	0.014890091	0.006670589	3
BP	GO:0002675	2/71	31/18670	0.00617432	0.015636327	0.007004894	2
BP	GO:0040018	2/71	31/18670	0.00617432	0.015636327	0.007004894	2
BP	GO:0043457	2/71	31/18670	0.00617432	0.015636327	0.007004894	2
BP	GO:0045737	2/71	31/18670	0.00617432	0.015636327	0.007004894	2
BP	GO:0045879	2/71	31/18670	0.00617432	0.015636327	0.007004894	2
BP	GO:1902230	2/71	31/18670	0.00617432	0.015636327	0.007004894	2
BP	GO:1902253	2/71	31/18670	0.00617432	0.015636327	0.007004894	2
BP	GO:0002042	3/71	98/18670	0.006192303	0.015670033	0.007019994	3
BP	GO:1901570	3/71	99/18670	0.00636861	0.016104033	0.007214421	3
BP	GO:0007215	3/71	100/18670	0.006547892	0.016463884	0.00737563	3
BP	GO:0030301	3/71	100/18670	0.006547892	0.016463884	0.00737563	3
BP	GO:0055024	3/71	100/18670	0.006547892	0.016463884	0.00737563	3
BP	GO:0001975	2/71	32/18670	0.006569841	0.016463884	0.00737563	2
BP	GO:0003176	2/71	32/18670	0.006569841	0.016463884	0.00737563	2
BP	GO:0008210	2/71	32/18670	0.006569841	0.016463884	0.00737563	2
BP	GO:0039694	2/71	32/18670	0.006569841	0.016463884	0.00737563	2
BP	GO:0045589	2/71	32/18670	0.006569841	0.016463884	0.00737563	2
BP	GO:0051385	2/71	32/18670	0.006569841	0.016463884	0.00737563	2
BP	GO:0055094	2/71	32/18670	0.006569841	0.016463884	0.00737563	2
BP	GO:0060674	2/71	32/18670	0.006569841	0.016463884	0.00737563	2
BP	GO:1900745	2/71	32/18670	0.006569841	0.016463884	0.00737563	2
BP	GO:0046632	3/71	101/18670	0.006730163	0.016845698	0.007546678	3
BP	GO:0009156	4/71	197/18670	0.006737274	0.016845698	0.007546678	4

BP	GO:0009749	4/71	197/18670	0.006737274	0.016845698	0.007546678	4
BP	GO:0051961	5/71	315/18670	0.006955466	0.017356145	0.007775352	5
BP	GO:0045736	2/71	33/18670	0.006976609	0.017356145	0.007775352	2
BP	GO:0051194	2/71	33/18670	0.006976609	0.017356145	0.007775352	2
BP	GO:0070232	2/71	33/18670	0.006976609	0.017356145	0.007775352	2
BP	GO:1902692	2/71	33/18670	0.006976609	0.017356145	0.007775352	2
BP	GO:2000352	2/71	33/18670	0.006976609	0.017356145	0.007775352	2
BP	GO:0016052	4/71	199/18670	0.006977657	0.017356145	0.007775352	4
BP	GO:0006898	5/71	316/18670	0.007046785	0.017515101	0.007846563	5
BP	GO:0032652	3/71	103/18670	0.007103722	0.017607884	0.007888129	3
BP	GO:0034766	3/71	103/18670	0.007103722	0.017607884	0.007888129	3
BP	GO:0044264	3/71	103/18670	0.007103722	0.017607884	0.007888129	3
BP	GO:0007265	6/71	448/18670	0.00710512	0.017607884	0.007888129	6
BP	GO:0048285	6/71	449/18670	0.00718011	0.017780584	0.007965496	6
BP	GO:0019233	3/71	104/18670	0.007295033	0.018038529	0.008081053	3
BP	GO:0032414	3/71	104/18670	0.007295033	0.018038529	0.008081053	3
BP	GO:0007204	5/71	319/18670	0.007325787	0.018101225	0.00810914	5
BP	GO:0000737	2/71	34/18670	0.007394535	0.018177329	0.008143234	2
BP	GO:0034205	2/71	34/18670	0.007394535	0.018177329	0.008143234	2
BP	GO:0042533	2/71	34/18670	0.007394535	0.018177329	0.008143234	2
BP	GO:0042534	2/71	34/18670	0.007394535	0.018177329	0.008143234	2
BP	GO:0045066	2/71	34/18670	0.007394535	0.018177329	0.008143234	2
BP	GO:0071402	2/71	34/18670	0.007394535	0.018177329	0.008143234	2
BP	GO:1904030	2/71	34/18670	0.007394535	0.018177329	0.008143234	2
BP	GO:0008593	3/71	105/18670	0.007489381	0.018383525	0.008235607	3
BP	GO:0045185	3/71	105/18670	0.007489381	0.018383525	0.008235607	3
BP	GO:0060562	5/71	322/18670	0.007612437	0.01867191	0.0083648	5
BP	GO:0002286	3/71	106/18670	0.007686777	0.01884047	0.008440313	3
BP	GO:0060251	2/71	35/18670	0.007823532	0.0191337	0.008571676	2
BP	GO:0070873	2/71	35/18670	0.007823532	0.0191337	0.008571676	2
BP	GO:1902991	2/71	35/18670	0.007823532	0.0191337	0.008571676	2
BP	GO:0006275	3/71	108/18670	0.008090757	0.019744039	0.008845101	3
BP	GO:0009062	3/71	108/18670	0.008090757	0.019744039	0.008845101	3
BP	GO:0046916	3/71	108/18670	0.008090757	0.019744039	0.008845101	3
BP	GO:0009124	4/71	208/18670	0.008129712	0.019810266	0.00887477	4
BP	GO:0050792	4/71	208/18670	0.008129712	0.019810266	0.00887477	4
BP	GO:0003091	2/71	36/18670	0.008263511	0.01993349	0.008929973	2
BP	GO:0010742	2/71	36/18670	0.008263511	0.01993349	0.008929973	2
BP	GO:0030224	2/71	36/18670	0.008263511	0.01993349	0.008929973	2
BP	GO:0030947	2/71	36/18670	0.008263511	0.01993349	0.008929973	2
BP	GO:0034390	2/71	36/18670	0.008263511	0.01993349	0.008929973	2
BP	GO:0034391	2/71	36/18670	0.008263511	0.01993349	0.008929973	2
BP	GO:0045740	2/71	36/18670	0.008263511	0.01993349	0.008929973	2
BP	GO:0051973	2/71	36/18670	0.008263511	0.01993349	0.008929973	2
BP	GO:0071634	2/71	36/18670	0.008263511	0.01993349	0.008929973	2
BP	GO:0090077	2/71	36/18670	0.008263511	0.01993349	0.008929973	2
BP	GO:0090322	2/71	36/18670	0.008263511	0.01993349	0.008929973	2
BP	GO:1903131	2/71	36/18670	0.008263511	0.01993349	0.008929973	2
BP	GO:2000310	2/71	36/18670	0.008263511	0.01993349	0.008929973	2
BP	GO:2001171	2/71	36/18670	0.008263511	0.01993349	0.008929973	2
BP	GO:0001676	3/71	109/18670	0.008297363	0.02000076	0.008960109	3
BP	GO:0008544	6/71	464/18670	0.008375221	0.020173935	0.00903769	6
BP	GO:0001662	2/71	37/18670	0.008714385	0.020913932	0.0093692	2
BP	GO:0038083	2/71	37/18670	0.008714385	0.020913932	0.0093692	2
BP	GO:0090050	2/71	37/18670	0.008714385	0.020913932	0.0093692	2
BP	GO:1905314	2/71	37/18670	0.008714385	0.020913932	0.0093692	2
BP	GO:2000142	2/71	37/18670	0.008714385	0.020913932	0.0093692	2
BP	GO:0022037	3/71	111/18670	0.008719856	0.020913932	0.0093692	3
BP	GO:0015918	3/71	112/18670	0.008935763	0.021370623	0.009573792	3
BP	GO:0042303	3/71	112/18670	0.008935763	0.021370623	0.009573792	3
BP	GO:0042633	3/71	112/18670	0.008935763	0.021370623	0.009573792	3
BP	GO:1903510	3/71	112/18670	0.008935763	0.021370623	0.009573792	3
BP	GO:0010810	4/71	215/18670	0.009107549	0.021750435	0.009743944	4

BP	GO:0070374	4/71	215/18670	0.009107549	0.021750435	0.009743944	4
BP	GO:0060964	3/71	113/18670	0.009154791	0.021774489	0.009754719	3
BP	GO:0002209	2/71	38/18670	0.009176067	0.021774489	0.009754719	2
BP	GO:0006778	2/71	38/18670	0.009176067	0.021774489	0.009754719	2
BP	GO:0010939	2/71	38/18670	0.009176067	0.021774489	0.009754719	2
BP	GO:0046825	2/71	38/18670	0.009176067	0.021774489	0.009754719	2
BP	GO:0071604	2/71	38/18670	0.009176067	0.021774489	0.009754719	2
BP	GO:1904037	2/71	38/18670	0.009176067	0.021774489	0.009754719	2
BP	GO:1905898	2/71	38/18670	0.009176067	0.021774489	0.009754719	2
BP	GO:2000279	2/71	38/18670	0.009176067	0.021774489	0.009754719	2
BP	GO:0005976	3/71	114/18670	0.009376948	0.022204029	0.009947149	3
BP	GO:0030218	3/71	114/18670	0.009376948	0.022204029	0.009947149	3
BP	GO:0030282	3/71	114/18670	0.009376948	0.022204029	0.009947149	3
BP	GO:0044242	4/71	217/18670	0.009400421	0.022243904	0.009965012	4
BP	GO:0019722	4/71	218/18670	0.009549136	0.022579869	0.01011552	4
BP	GO:0019048	2/71	39/18670	0.009648471	0.022734592	0.010184834	2
BP	GO:0033574	2/71	39/18670	0.009648471	0.022734592	0.010184834	2
BP	GO:0060969	2/71	39/18670	0.009648471	0.022734592	0.010184834	2
BP	GO:0072595	2/71	39/18670	0.009648471	0.022734592	0.010184834	2
BP	GO:1902229	2/71	39/18670	0.009648471	0.022734592	0.010184834	2
BP	GO:0015711	6/71	482/18670	0.009991478	0.023526283	0.010539503	6
BP	GO:0031398	3/71	117/18670	0.010062284	0.023626638	0.010584461	3
BP	GO:0051153	3/71	117/18670	0.010062284	0.023626638	0.010584461	3
BP	GO:0060147	3/71	117/18670	0.010062284	0.023626638	0.010584461	3
BP	GO:0060966	3/71	117/18670	0.010062284	0.023626638	0.010584461	3
BP	GO:0042026	2/71	40/18670	0.010131512	0.023739314	0.010634938	2
BP	GO:0042596	2/71	40/18670	0.010131512	0.023739314	0.010634938	2
BP	GO:0045124	2/71	40/18670	0.010131512	0.023739314	0.010634938	2
BP	GO:0022604	6/71	484/18670	0.010183892	0.023845384	0.010682457	6
BP	GO:0001952	3/71	119/18670	0.010534982	0.02465024	0.011043023	3
BP	GO:0006308	2/71	41/18670	0.010625103	0.024774665	0.011098764	2
BP	GO:0030890	2/71	41/18670	0.010625103	0.024774665	0.011098764	2
BP	GO:0043267	2/71	41/18670	0.010625103	0.024774665	0.011098764	2
BP	GO:0061028	2/71	41/18670	0.010625103	0.024774665	0.011098764	2
BP	GO:0097178	2/71	41/18670	0.010625103	0.024774665	0.011098764	2
BP	GO:0017015	3/71	120/18670	0.010776097	0.025109279	0.011248667	3
BP	GO:0034404	4/71	226/18670	0.010794349	0.02512623	0.011256261	4
BP	GO:0051260	5/71	351/18670	0.010798359	0.02512623	0.011256261	5
BP	GO:0006457	4/71	227/18670	0.010957024	0.02547774	0.011413733	4
BP	GO:0006509	2/71	42/18670	0.011129159	0.025824271	0.011568975	2
BP	GO:0051154	2/71	42/18670	0.011129159	0.025824271	0.011568975	2
BP	GO:1903146	2/71	42/18670	0.011129159	0.025824271	0.011568975	2
BP	GO:0019730	3/71	122/18670	0.011267898	0.026110064	0.011697007	3
BP	GO:1903844	3/71	122/18670	0.011267898	0.026110064	0.011697007	3
BP	GO:0022412	5/71	357/18670	0.011556337	0.026741476	0.011979872	5
BP	GO:0051480	5/71	357/18670	0.011556337	0.026741476	0.011979872	5
BP	GO:0001953	2/71	43/18670	0.011643598	0.026795459	0.012004056	2
BP	GO:0006692	2/71	43/18670	0.011643598	0.026795459	0.012004056	2
BP	GO:0006693	2/71	43/18670	0.011643598	0.026795459	0.012004056	2
BP	GO:0034198	2/71	43/18670	0.011643598	0.026795459	0.012004056	2
BP	GO:0042088	2/71	43/18670	0.011643598	0.026795459	0.012004056	2
BP	GO:0043616	2/71	43/18670	0.011643598	0.026795459	0.012004056	2
BP	GO:0045746	2/71	43/18670	0.011643598	0.026795459	0.012004056	2
BP	GO:0071364	2/71	43/18670	0.011643598	0.026795459	0.012004056	2
BP	GO:0051209	3/71	124/18670	0.011772509	0.027073541	0.012128634	3
BP	GO:0003197	2/71	44/18670	0.012168334	0.02788819	0.012493587	2
BP	GO:0042987	2/71	44/18670	0.012168334	0.02788819	0.012493587	2
BP	GO:0061756	2/71	44/18670	0.012168334	0.02788819	0.012493587	2
BP	GO:0090311	2/71	44/18670	0.012168334	0.02788819	0.012493587	2
BP	GO:1903573	2/71	44/18670	0.012168334	0.02788819	0.012493587	2
BP	GO:0018107	3/71	126/18670	0.012289985	0.028109358	0.012592668	3
BP	GO:0051283	3/71	126/18670	0.012289985	0.028109358	0.012592668	3
BP	GO:0090101	3/71	126/18670	0.012289985	0.028109358	0.012592668	3

BP	GO:0046777	4/71	235/18670	0.01231576	0.028149109	0.012610476	4
BP	GO:0035270	3/71	127/18670	0.012553563	0.02867309	0.012845213	3
BP	GO:0001974	2/71	45/18670	0.012703284	0.028955887	0.012971903	2
BP	GO:0031018	2/71	45/18670	0.012703284	0.028955887	0.012971903	2
BP	GO:0035987	2/71	45/18670	0.012703284	0.028955887	0.012971903	2
BP	GO:0002576	3/71	128/18670	0.012820376	0.029183111	0.013073696	3
BP	GO:0051282	3/71	128/18670	0.012820376	0.029183111	0.013073696	3
BP	GO:0003206	3/71	129/18670	0.013090431	0.029757434	0.013330987	3
BP	GO:0050853	3/71	129/18670	0.013090431	0.029757434	0.013330987	3
BP	GO:0005978	2/71	46/18670	0.013248365	0.029913647	0.013400968	2
BP	GO:0007157	2/71	46/18670	0.013248365	0.029913647	0.013400968	2
BP	GO:0009250	2/71	46/18670	0.013248365	0.029913647	0.013400968	2
BP	GO:0014075	2/71	46/18670	0.013248365	0.029913647	0.013400968	2
BP	GO:0035196	2/71	46/18670	0.013248365	0.029913647	0.013400968	2
BP	GO:0043330	2/71	46/18670	0.013248365	0.029913647	0.013400968	2
BP	GO:0044003	2/71	46/18670	0.013248365	0.029913647	0.013400968	2
BP	GO:0071354	2/71	46/18670	0.013248365	0.029913647	0.013400968	2
BP	GO:0071675	2/71	46/18670	0.013248365	0.029913647	0.013400968	2
BP	GO:1990928	2/71	46/18670	0.013248365	0.029913647	0.013400968	2
BP	GO:0038093	4/71	241/18670	0.013402824	0.030242038	0.013548084	4
BP	GO:0051208	3/71	131/18670	0.013640286	0.030736478	0.013769587	3
BP	GO:0055076	3/71	131/18670	0.013640286	0.030736478	0.013769587	3
BP	GO:0014911	2/71	47/18670	0.013803494	0.030917245	0.013850569	2
BP	GO:0031952	2/71	47/18670	0.013803494	0.030917245	0.013850569	2
BP	GO:0035722	2/71	47/18670	0.013803494	0.030917245	0.013850569	2
BP	GO:0042551	2/71	47/18670	0.013803494	0.030917245	0.013850569	2
BP	GO:0048066	2/71	47/18670	0.013803494	0.030917245	0.013850569	2
BP	GO:0048806	2/71	47/18670	0.013803494	0.030917245	0.013850569	2
BP	GO:0050435	2/71	47/18670	0.013803494	0.030917245	0.013850569	2
BP	GO:1900744	2/71	47/18670	0.013803494	0.030917245	0.013850569	2
BP	GO:1903580	2/71	47/18670	0.013803494	0.030917245	0.013850569	2
BP	GO:0003158	3/71	132/18670	0.013920097	0.031136815	0.013948934	3
BP	GO:0072329	3/71	132/18670	0.013920097	0.031136815	0.013948934	3
BP	GO:0001774	2/71	48/18670	0.01436859	0.031927026	0.014302939	2
BP	GO:0002269	2/71	48/18670	0.01436859	0.031927026	0.014302939	2
BP	GO:0007595	2/71	48/18670	0.01436859	0.031927026	0.014302939	2
BP	GO:0008089	2/71	48/18670	0.01436859	0.031927026	0.014302939	2
BP	GO:0035272	2/71	48/18670	0.01436859	0.031927026	0.014302939	2
BP	GO:0045912	2/71	48/18670	0.01436859	0.031927026	0.014302939	2
BP	GO:0046850	2/71	48/18670	0.01436859	0.031927026	0.014302939	2
BP	GO:0060324	2/71	48/18670	0.01436859	0.031927026	0.014302939	2
BP	GO:0101023	2/71	48/18670	0.01436859	0.031927026	0.014302939	2
BP	GO:1905562	2/71	48/18670	0.01436859	0.031927026	0.014302939	2
BP	GO:0007272	3/71	134/18670	0.014489512	0.032131835	0.014394691	3
BP	GO:0008366	3/71	134/18670	0.014489512	0.032131835	0.014394691	3
BP	GO:0018210	3/71	134/18670	0.014489512	0.032131835	0.014394691	3
BP	GO:0060359	3/71	135/18670	0.014779126	0.032730785	0.014663014	3
BP	GO:1903322	3/71	135/18670	0.014779126	0.032730785	0.014663014	3
BP	GO:0003044	2/71	49/18670	0.014943571	0.03300777	0.014787099	2
BP	GO:0032757	2/71	49/18670	0.014943571	0.03300777	0.014787099	2
BP	GO:0061912	2/71	49/18670	0.014943571	0.03300777	0.014787099	2
BP	GO:0071349	2/71	49/18670	0.014943571	0.03300777	0.014787099	2
BP	GO:0001706	2/71	50/18670	0.015528356	0.034164421	0.015305266	2
BP	GO:0014009	2/71	50/18670	0.015528356	0.034164421	0.015305266	2
BP	GO:0045540	2/71	50/18670	0.015528356	0.034164421	0.015305266	2
BP	GO:0070671	2/71	50/18670	0.015528356	0.034164421	0.015305266	2
BP	GO:0070741	2/71	50/18670	0.015528356	0.034164421	0.015305266	2
BP	GO:0106118	2/71	50/18670	0.015528356	0.034164421	0.015305266	2
BP	GO:2000027	4/71	253/18670	0.015756533	0.034643708	0.015519981	4
BP	GO:0002700	3/71	139/18670	0.015970387	0.035044966	0.01569974	3
BP	GO:0045580	3/71	139/18670	0.015970387	0.035044966	0.01569974	3
BP	GO:0050715	3/71	139/18670	0.015970387	0.035044966	0.01569974	3
BP	GO:0031050	2/71	51/18670	0.016122864	0.035287182	0.01580825	2

BP	GO:0070918	2/71	51/18670	0.016122864	0.035287182	0.01580825	2
BP	GO:0097366	2/71	51/18670	0.016122864	0.035287182	0.01580825	2
BP	GO:2001258	2/71	51/18670	0.016122864	0.035287182	0.01580825	2
BP	GO:0001837	3/71	140/18670	0.016276424	0.035530501	0.015917254	3
BP	GO:0046330	3/71	140/18670	0.016276424	0.035530501	0.015917254	3
BP	GO:0097553	3/71	140/18670	0.016276424	0.035530501	0.015917254	3
BP	GO:1903364	3/71	140/18670	0.016276424	0.035530501	0.015917254	3
BP	GO:0008286	3/71	141/18670	0.016585757	0.036179705	0.01620809	3
BP	GO:0048863	4/71	257/18670	0.016595403	0.036179705	0.01620809	4
BP	GO:0003179	2/71	52/18670	0.016727015	0.03639564	0.016304826	2
BP	GO:0031529	2/71	52/18670	0.016727015	0.03639564	0.016304826	2
BP	GO:2000677	2/71	52/18670	0.016727015	0.03639564	0.016304826	2
BP	GO:0031056	3/71	143/18670	0.017214324	0.03738318	0.016747233	3
BP	GO:0106106	3/71	143/18670	0.017214324	0.03738318	0.016747233	3
BP	GO:0120161	3/71	143/18670	0.017214324	0.03738318	0.016747233	3
BP	GO:0000186	2/71	53/18670	0.017340729	0.037536129	0.016815752	2
BP	GO:0038066	2/71	53/18670	0.017340729	0.037536129	0.016815752	2
BP	GO:0043331	2/71	53/18670	0.017340729	0.037536129	0.016815752	2
BP	GO:0045744	2/71	53/18670	0.017340729	0.037536129	0.016815752	2
BP	GO:0060563	2/71	53/18670	0.017340729	0.037536129	0.016815752	2
BP	GO:0060041	3/71	144/18670	0.017533564	0.037929059	0.01699178	3
BP	GO:0007281	4/71	262/18670	0.017682851	0.038202705	0.01711437	4
BP	GO:0015850	4/71	262/18670	0.017682851	0.038202705	0.01711437	4
BP	GO:0006636	2/71	54/18670	0.017963927	0.038760004	0.017364034	2
BP	GO:0032663	2/71	54/18670	0.017963927	0.038760004	0.017364034	2
BP	GO:0002224	3/71	146/18670	0.018181971	0.039205241	0.017563495	3
BP	GO:0042304	2/71	55/18670	0.018596529	0.040021928	0.017929361	2
BP	GO:0046456	2/71	55/18670	0.018596529	0.040021928	0.017929361	2
BP	GO:0061098	2/71	55/18670	0.018596529	0.040021928	0.017929361	2
BP	GO:0008277	3/71	149/18670	0.019179434	0.04119708	0.018455816	3
BP	GO:0048592	3/71	149/18670	0.019179434	0.04119708	0.018455816	3
BP	GO:1903900	3/71	149/18670	0.019179434	0.04119708	0.018455816	3
BP	GO:0006749	2/71	56/18670	0.019238459	0.041270987	0.018488925	2
BP	GO:0090183	2/71	56/18670	0.019238459	0.041270987	0.018488925	2
BP	GO:0016331	3/71	150/18670	0.019518557	0.041818353	0.018734139	3
BP	GO:0050777	3/71	150/18670	0.019518557	0.041818353	0.018734139	3
BP	GO:0055088	3/71	151/18670	0.019861001	0.042497726	0.01903849	3
BP	GO:1903169	3/71	151/18670	0.019861001	0.042497726	0.01903849	3
BP	GO:0048008	2/71	57/18670	0.019889637	0.042531858	0.019053781	2
BP	GO:0030902	3/71	152/18670	0.020206767	0.043182467	0.019345246	3
BP	GO:0061448	4/71	273/18670	0.020229524	0.043203564	0.019354697	4
BP	GO:0034250	3/71	153/18670	0.020555857	0.043872558	0.019654399	3
BP	GO:0055067	3/71	154/18670	0.020908272	0.044567982	0.019965941	3
BP	GO:1990845	3/71	154/18670	0.020908272	0.044567982	0.019965941	3
BP	GO:0001885	2/71	59/18670	0.021219429	0.045116516	0.020211678	2
BP	GO:0032890	2/71	59/18670	0.021219429	0.045116516	0.020211678	2
BP	GO:0033619	2/71	59/18670	0.021219429	0.045116516	0.020211678	2
BP	GO:0061077	2/71	59/18670	0.021219429	0.045116516	0.020211678	2
BP	GO:0060968	3/71	155/18670	0.021264012	0.045182657	0.020241308	3
BP	GO:0043588	5/71	419/18670	0.02160219	0.045872162	0.020550198	5
BP	GO:0033013	2/71	60/18670	0.02189789	0.046470648	0.020818314	2
BP	GO:0003170	2/71	61/18670	0.022585292	0.047778222	0.021404092	2
BP	GO:0007405	2/71	61/18670	0.022585292	0.047778222	0.021404092	2
BP	GO:0030521	2/71	61/18670	0.022585292	0.047778222	0.021404092	2
BP	GO:0034113	2/71	61/18670	0.022585292	0.047778222	0.021404092	2
BP	GO:0048645	2/71	61/18670	0.022585292	0.047778222	0.021404092	2
BP	GO:0016579	4/71	283/18670	0.022732015	0.048058288	0.021529558	4
BP	GO:0002819	3/71	160/18670	0.023092624	0.048759174	0.021843547	3
BP	GO:0030203	3/71	160/18670	0.023092624	0.048759174	0.021843547	3
BP	GO:0006521	2/71	62/18670	0.023281559	0.048881068	0.021898154	2
BP	GO:0010830	2/71	62/18670	0.023281559	0.048881068	0.021898154	2
BP	GO:0031640	2/71	62/18670	0.023281559	0.048881068	0.021898154	2
BP	GO:0032623	2/71	62/18670	0.023281559	0.048881068	0.021898154	2

BP	GO:0032835	2/71	62/18670	0.023281559	0.048881068	0.021898154	2
BP	GO:0044364	2/71	62/18670	0.023281559	0.048881068	0.021898154	2
BP	GO:0046635	2/71	62/18670	0.023281559	0.048881068	0.021898154	2
BP	GO:0048857	2/71	62/18670	0.023281559	0.048881068	0.021898154	2
BP	GO:0055025	2/71	62/18670	0.023281559	0.048881068	0.021898154	2
BP	GO:0010970	3/71	161/18670	0.023468331	0.049211578	0.022046218	3
BP	GO:0099111	3/71	161/18670	0.023468331	0.049211578	0.022046218	3
BP	GO:0007588	2/71	63/18670	0.023986616	0.050110357	0.022448861	2
BP	GO:0032371	2/71	63/18670	0.023986616	0.050110357	0.022448861	2
BP	GO:0032374	2/71	63/18670	0.023986616	0.050110357	0.022448861	2
BP	GO:0042982	2/71	63/18670	0.023986616	0.050110357	0.022448861	2
BP	GO:0046622	2/71	63/18670	0.023986616	0.050110357	0.022448861	2
BP	GO:0090181	2/71	63/18670	0.023986616	0.050110357	0.022448861	2
BP	GO:0048562	4/71	288/18670	0.024051197	0.050213987	0.022495286	4
BP	GO:0036498	2/71	64/18670	0.024700388	0.05137742	0.023016491	2
BP	GO:0043550	2/71	64/18670	0.024700388	0.05137742	0.023016491	2
BP	GO:0045669	2/71	64/18670	0.024700388	0.05137742	0.023016491	2
BP	GO:0045670	2/71	64/18670	0.024700388	0.05137742	0.023016491	2
BP	GO:0048247	2/71	64/18670	0.024700388	0.05137742	0.023016491	2
BP	GO:1903672	2/71	64/18670	0.024700388	0.05137742	0.023016491	2
BP	GO:0002244	3/71	166/18670	0.02539678	0.052716545	0.023616403	3
BP	GO:0090288	3/71	166/18670	0.02539678	0.052716545	0.023616403	3
BP	GO:0014823	2/71	65/18670	0.025422801	0.052716545	0.023616403	2
BP	GO:0032729	2/71	65/18670	0.025422801	0.052716545	0.023616403	2
BP	GO:0051148	2/71	65/18670	0.025422801	0.052716545	0.023616403	2
BP	GO:0010256	5/71	438/18670	0.025553889	0.052955618	0.023723505	5
BP	GO:0046626	2/71	66/18670	0.026153781	0.054131869	0.024250452	2
BP	GO:1905207	2/71	66/18670	0.026153781	0.054131869	0.024250452	2
BP	GO:0001906	3/71	168/18670	0.026191441	0.054176374	0.02427039	3
BP	GO:0038095	3/71	169/18670	0.026593757	0.054940769	0.02461283	3
BP	GO:0045619	3/71	169/18670	0.026593757	0.054940769	0.02461283	3
BP	GO:2000573	2/71	67/18670	0.026893254	0.055525295	0.024874691	2
BP	GO:0006022	3/71	170/18670	0.026999395	0.055675875	0.024942149	3
BP	GO:0043433	3/71	170/18670	0.026999395	0.055675875	0.024942149	3
BP	GO:0070646	4/71	299/18670	0.027114906	0.055879704	0.025033462	4
BP	GO:0003205	3/71	171/18670	0.027408353	0.056449759	0.02528884	3
BP	GO:0010812	2/71	68/18670	0.027641146	0.056789684	0.025441122	2
BP	GO:0035914	2/71	68/18670	0.027641146	0.056789684	0.025441122	2
BP	GO:0046323	2/71	68/18670	0.027641146	0.056789684	0.025441122	2
BP	GO:0071242	2/71	68/18670	0.027641146	0.056789684	0.025441122	2
BP	GO:0030324	3/71	172/18670	0.02782063	0.057088478	0.025574978	3
BP	GO:0032874	3/71	172/18670	0.02782063	0.057088478	0.025574978	3
BP	GO:0070304	3/71	173/18670	0.028236224	0.057905846	0.02594115	3
BP	GO:0045088	5/71	452/18670	0.028745948	0.058915138	0.026393301	5
BP	GO:0046034	4/71	305/18670	0.028880497	0.059154738	0.026500639	4
BP	GO:0002695	3/71	175/18670	0.029077354	0.059521592	0.026664986	3
BP	GO:0007004	2/71	70/18670	0.0291619	0.059549239	0.026677371	2
BP	GO:0034121	2/71	70/18670	0.0291619	0.059549239	0.026677371	2
BP	GO:0060395	2/71	70/18670	0.0291619	0.059549239	0.026677371	2
BP	GO:0072091	2/71	70/18670	0.0291619	0.059549239	0.026677371	2
BP	GO:0030323	3/71	176/18670	0.029502887	0.060208873	0.02697288	3
BP	GO:0043488	3/71	177/18670	0.029931728	0.060978597	0.027317707	3
BP	GO:0042440	2/71	71/18670	0.029934617	0.060978597	0.027317707	2
BP	GO:0042531	2/71	71/18670	0.029934617	0.060978597	0.027317707	2
BP	GO:0002697	5/71	458/18670	0.03018835	0.061420836	0.027515825	5
BP	GO:0006874	5/71	458/18670	0.03018835	0.061420836	0.027515825	5
BP	GO:0022406	3/71	178/18670	0.030363875	0.061703074	0.027642264	3
BP	GO:0050821	3/71	178/18670	0.030363875	0.061703074	0.027642264	3
BP	GO:0002532	2/71	72/18670	0.030715465	0.062304268	0.027911592	2
BP	GO:0032024	2/71	72/18670	0.030715465	0.062304268	0.027911592	2
BP	GO:0050805	2/71	72/18670	0.030715465	0.062304268	0.027911592	2
BP	GO:0030705	3/71	180/18670	0.031238078	0.063249562	0.028335073	3
BP	GO:0070613	3/71	180/18670	0.031238078	0.063249562	0.028335073	3

BP	GO:1901796	3/71	180/18670	0.031238078	0.063249562	0.028335073	3
BP	GO:0033555	2/71	73/18670	0.031504373	0.0637118	0.02854215	2
BP	GO:0045685	2/71	73/18670	0.031504373	0.0637118	0.02854215	2
BP	GO:0009566	3/71	182/18670	0.032125472	0.064889584	0.029069784	3
BP	GO:1903317	3/71	182/18670	0.032125472	0.064889584	0.029069784	3
BP	GO:0001895	2/71	74/18670	0.032301271	0.065087837	0.029158599	2
BP	GO:0005977	2/71	74/18670	0.032301271	0.065087837	0.029158599	2
BP	GO:0032677	2/71	74/18670	0.032301271	0.065087837	0.029158599	2
BP	GO:1900076	2/71	74/18670	0.032301271	0.065087837	0.029158599	2
BP	GO:0002040	3/71	183/18670	0.032574109	0.06551949	0.029351974	3
BP	GO:0038061	3/71	183/18670	0.032574109	0.06551949	0.029351974	3
BP	GO:0043487	3/71	183/18670	0.032574109	0.06551949	0.029351974	3
BP	GO:0043409	3/71	184/18670	0.033026035	0.06634889	0.029723536	3
BP	GO:0050864	3/71	184/18670	0.033026035	0.06634889	0.029723536	3
BP	GO:0001960	2/71	75/18670	0.033106087	0.066350694	0.029724344	2
BP	GO:0006073	2/71	75/18670	0.033106087	0.066350694	0.029724344	2
BP	GO:0006695	2/71	75/18670	0.033106087	0.066350694	0.029724344	2
BP	GO:0044042	2/71	75/18670	0.033106087	0.066350694	0.029724344	2
BP	GO:0055074	5/71	471/18670	0.033469051	0.066982471	0.030007373	5
BP	GO:0007369	3/71	185/18670	0.033481247	0.066982471	0.030007373	3
BP	GO:1902275	3/71	185/18670	0.033481247	0.066982471	0.030007373	3
BP	GO:0000422	2/71	76/18670	0.033918753	0.067535379	0.03025507	2
BP	GO:0007492	2/71	76/18670	0.033918753	0.067535379	0.03025507	2
BP	GO:0019674	2/71	76/18670	0.033918753	0.067535379	0.03025507	2
BP	GO:0030500	2/71	76/18670	0.033918753	0.067535379	0.03025507	2
BP	GO:0045454	2/71	76/18670	0.033918753	0.067535379	0.03025507	2
BP	GO:0060411	2/71	76/18670	0.033918753	0.067535379	0.03025507	2
BP	GO:0061726	2/71	76/18670	0.033918753	0.067535379	0.03025507	2
BP	GO:1902653	2/71	76/18670	0.033918753	0.067535379	0.03025507	2
BP	GO:0022900	3/71	186/18670	0.03393974	0.067537062	0.030255824	3
BP	GO:0006278	2/71	77/18670	0.034739198	0.068923392	0.030876883	2
BP	GO:0031397	2/71	77/18670	0.034739198	0.068923392	0.030876883	2
BP	GO:0051851	2/71	77/18670	0.034739198	0.068923392	0.030876883	2
BP	GO:0061418	2/71	77/18670	0.034739198	0.068923392	0.030876883	2
BP	GO:1901224	2/71	77/18670	0.034739198	0.068923392	0.030876883	2
BP	GO:0043902	3/71	188/18670	0.03486656	0.069135171	0.030971758	3
BP	GO:0008306	2/71	78/18670	0.035567354	0.070151376	0.031427006	2
BP	GO:0008589	2/71	78/18670	0.035567354	0.070151376	0.031427006	2
BP	GO:0010827	2/71	78/18670	0.035567354	0.070151376	0.031427006	2
BP	GO:0030433	2/71	78/18670	0.035567354	0.070151376	0.031427006	2
BP	GO:0032413	2/71	78/18670	0.035567354	0.070151376	0.031427006	2
BP	GO:0042246	2/71	78/18670	0.035567354	0.070151376	0.031427006	2
BP	GO:0043407	2/71	78/18670	0.035567354	0.070151376	0.031427006	2
BP	GO:0055021	2/71	78/18670	0.035567354	0.070151376	0.031427006	2
BP	GO:0090398	2/71	78/18670	0.035567354	0.070151376	0.031427006	2
BP	GO:0043087	5/71	479/18670	0.035594896	0.070164424	0.031432852	5
BP	GO:0003151	2/71	79/18670	0.036403152	0.071198508	0.031896109	2
BP	GO:0021675	2/71	79/18670	0.036403152	0.071198508	0.031896109	2
BP	GO:1900034	2/71	79/18670	0.036403152	0.071198508	0.031896109	2
BP	GO:0034248	5/71	483/18670	0.03668866	0.071198508	0.031896109	5
BP	GO:0007219	3/71	193/18670	0.037240781	0.071198508	0.031896109	3
BP	GO:0002291	1/71	10/18670	0.03739355	0.071198508	0.031896109	1
BP	GO:0007168	1/71	10/18670	0.03739355	0.071198508	0.031896109	1
BP	GO:0010739	1/71	10/18670	0.03739355	0.071198508	0.031896109	1
BP	GO:0010749	1/71	10/18670	0.03739355	0.071198508	0.031896109	1
BP	GO:0010918	1/71	10/18670	0.03739355	0.071198508	0.031896109	1
BP	GO:0014041	1/71	10/18670	0.03739355	0.071198508	0.031896109	1
BP	GO:0016102	1/71	10/18670	0.03739355	0.071198508	0.031896109	1
BP	GO:0019062	1/71	10/18670	0.03739355	0.071198508	0.031896109	1
BP	GO:0021548	1/71	10/18670	0.03739355	0.071198508	0.031896109	1
BP	GO:0030388	1/71	10/18670	0.03739355	0.071198508	0.031896109	1
BP	GO:0030647	1/71	10/18670	0.03739355	0.071198508	0.031896109	1
BP	GO:0031284	1/71	10/18670	0.03739355	0.071198508	0.031896109	1

BP	GO:0032000	1/71	10/18670	0.03739355	0.071198508	0.031896109	1
BP	GO:0032070	1/71	10/18670	0.03739355	0.071198508	0.031896109	1
BP	GO:0032308	1/71	10/18670	0.03739355	0.071198508	0.031896109	1
BP	GO:0034085	1/71	10/18670	0.03739355	0.071198508	0.031896109	1
BP	GO:0034350	1/71	10/18670	0.03739355	0.071198508	0.031896109	1
BP	GO:0035865	1/71	10/18670	0.03739355	0.071198508	0.031896109	1
BP	GO:0036500	1/71	10/18670	0.03739355	0.071198508	0.031896109	1
BP	GO:0043653	1/71	10/18670	0.03739355	0.071198508	0.031896109	1
BP	GO:0044359	1/71	10/18670	0.03739355	0.071198508	0.031896109	1
BP	GO:0044557	1/71	10/18670	0.03739355	0.071198508	0.031896109	1
BP	GO:0045348	1/71	10/18670	0.03739355	0.071198508	0.031896109	1
BP	GO:0045657	1/71	10/18670	0.03739355	0.071198508	0.031896109	1
BP	GO:0045713	1/71	10/18670	0.03739355	0.071198508	0.031896109	1
BP	GO:0045741	1/71	10/18670	0.03739355	0.071198508	0.031896109	1
BP	GO:0045792	1/71	10/18670	0.03739355	0.071198508	0.031896109	1
BP	GO:0045945	1/71	10/18670	0.03739355	0.071198508	0.031896109	1
BP	GO:0046322	1/71	10/18670	0.03739355	0.071198508	0.031896109	1
BP	GO:0051133	1/71	10/18670	0.03739355	0.071198508	0.031896109	1
BP	GO:0052205	1/71	10/18670	0.03739355	0.071198508	0.031896109	1
BP	GO:0060068	1/71	10/18670	0.03739355	0.071198508	0.031896109	1
BP	GO:0060346	1/71	10/18670	0.03739355	0.071198508	0.031896109	1
BP	GO:0060513	1/71	10/18670	0.03739355	0.071198508	0.031896109	1
BP	GO:0060768	1/71	10/18670	0.03739355	0.071198508	0.031896109	1
BP	GO:0061299	1/71	10/18670	0.03739355	0.071198508	0.031896109	1
BP	GO:0070099	1/71	10/18670	0.03739355	0.071198508	0.031896109	1
BP	GO:0070341	1/71	10/18670	0.03739355	0.071198508	0.031896109	1
BP	GO:0070344	1/71	10/18670	0.03739355	0.071198508	0.031896109	1
BP	GO:0070942	1/71	10/18670	0.03739355	0.071198508	0.031896109	1
BP	GO:0071281	1/71	10/18670	0.03739355	0.071198508	0.031896109	1
BP	GO:0072124	1/71	10/18670	0.03739355	0.071198508	0.031896109	1
BP	GO:0090336	1/71	10/18670	0.03739355	0.071198508	0.031896109	1
BP	GO:0090557	1/71	10/18670	0.03739355	0.071198508	0.031896109	1
BP	GO:0106049	1/71	10/18670	0.03739355	0.071198508	0.031896109	1
BP	GO:0110096	1/71	10/18670	0.03739355	0.071198508	0.031896109	1
BP	GO:0140052	1/71	10/18670	0.03739355	0.071198508	0.031896109	1
BP	GO:1900122	1/71	10/18670	0.03739355	0.071198508	0.031896109	1
BP	GO:1900222	1/71	10/18670	0.03739355	0.071198508	0.031896109	1
BP	GO:1902101	1/71	10/18670	0.03739355	0.071198508	0.031896109	1
BP	GO:1903332	1/71	10/18670	0.03739355	0.071198508	0.031896109	1
BP	GO:1903799	1/71	10/18670	0.03739355	0.071198508	0.031896109	1
BP	GO:1903897	1/71	10/18670	0.03739355	0.071198508	0.031896109	1
BP	GO:2000425	1/71	10/18670	0.03739355	0.071198508	0.031896109	1
BP	GO:2001054	1/71	10/18670	0.03739355	0.071198508	0.031896109	1
BP	GO:0014032	2/71	81/18670	0.0380974	0.072210617	0.032349522	2
BP	GO:0014855	2/71	81/18670	0.0380974	0.072210617	0.032349522	2
BP	GO:0016126	2/71	81/18670	0.0380974	0.072210617	0.032349522	2
BP	GO:0032436	2/71	81/18670	0.0380974	0.072210617	0.032349522	2
BP	GO:0033238	2/71	81/18670	0.0380974	0.072210617	0.032349522	2
BP	GO:0060761	2/71	81/18670	0.0380974	0.072210617	0.032349522	2
BP	GO:0110110	2/71	81/18670	0.0380974	0.072210617	0.032349522	2
BP	GO:2001021	2/71	81/18670	0.0380974	0.072210617	0.032349522	2
BP	GO:0009205	4/71	335/18670	0.038723468	0.073355812	0.032862556	4
BP	GO:0010833	2/71	82/18670	0.038955715	0.073587894	0.032966526	2
BP	GO:0030279	2/71	82/18670	0.038955715	0.073587894	0.032966526	2
BP	GO:0032637	2/71	82/18670	0.038955715	0.073587894	0.032966526	2
BP	GO:0051702	2/71	82/18670	0.038955715	0.073587894	0.032966526	2
BP	GO:0090049	2/71	82/18670	0.038955715	0.073587894	0.032966526	2
BP	GO:0002221	3/71	197/18670	0.039198688	0.07400518	0.033153466	3
BP	GO:0072503	5/71	493/18670	0.039513667	0.074557864	0.033401062	5
BP	GO:0060541	3/71	198/18670	0.039696256	0.074818182	0.033517681	3
BP	GO:0072376	3/71	198/18670	0.039696256	0.074818182	0.033517681	3
BP	GO:0061013	3/71	199/18670	0.040197051	0.075638154	0.033885019	3
BP	GO:0009167	4/71	340/18670	0.040529792	0.075638154	0.033885019	4

BP	GO:0002718	2/71	84/18670	0.040694391	0.075638154	0.033885019	2
BP	GO:0050886	2/71	84/18670	0.040694391	0.075638154	0.033885019	2
BP	GO:0060420	2/71	84/18670	0.040694391	0.075638154	0.033885019	2
BP	GO:0009126	4/71	341/18670	0.040896766	0.075638154	0.033885019	4
BP	GO:0009199	4/71	341/18670	0.040896766	0.075638154	0.033885019	4
BP	GO:0002887	1/71	11/18670	0.0410562	0.075638154	0.033885019	1
BP	GO:0006182	1/71	11/18670	0.0410562	0.075638154	0.033885019	1
BP	GO:0006620	1/71	11/18670	0.0410562	0.075638154	0.033885019	1
BP	GO:0006853	1/71	11/18670	0.0410562	0.075638154	0.033885019	1
BP	GO:0009629	1/71	11/18670	0.0410562	0.075638154	0.033885019	1
BP	GO:0016264	1/71	11/18670	0.0410562	0.075638154	0.033885019	1
BP	GO:0019371	1/71	11/18670	0.0410562	0.075638154	0.033885019	1
BP	GO:0031915	1/71	11/18670	0.0410562	0.075638154	0.033885019	1
BP	GO:0032306	1/71	11/18670	0.0410562	0.075638154	0.033885019	1
BP	GO:0033148	1/71	11/18670	0.0410562	0.075638154	0.033885019	1
BP	GO:0033327	1/71	11/18670	0.0410562	0.075638154	0.033885019	1
BP	GO:0033632	1/71	11/18670	0.0410562	0.075638154	0.033885019	1
BP	GO:0035747	1/71	11/18670	0.0410562	0.075638154	0.033885019	1
BP	GO:0036462	1/71	11/18670	0.0410562	0.075638154	0.033885019	1
BP	GO:0040015	1/71	11/18670	0.0410562	0.075638154	0.033885019	1
BP	GO:0045899	1/71	11/18670	0.0410562	0.075638154	0.033885019	1
BP	GO:0046007	1/71	11/18670	0.0410562	0.075638154	0.033885019	1
BP	GO:0048548	1/71	11/18670	0.0410562	0.075638154	0.033885019	1
BP	GO:0048742	1/71	11/18670	0.0410562	0.075638154	0.033885019	1
BP	GO:0060088	1/71	11/18670	0.0410562	0.075638154	0.033885019	1
BP	GO:0060439	1/71	11/18670	0.0410562	0.075638154	0.033885019	1
BP	GO:0060736	1/71	11/18670	0.0410562	0.075638154	0.033885019	1
BP	GO:0060767	1/71	11/18670	0.0410562	0.075638154	0.033885019	1
BP	GO:0060992	1/71	11/18670	0.0410562	0.075638154	0.033885019	1
BP	GO:0061307	1/71	11/18670	0.0410562	0.075638154	0.033885019	1
BP	GO:0061308	1/71	11/18670	0.0410562	0.075638154	0.033885019	1
BP	GO:0070673	1/71	11/18670	0.0410562	0.075638154	0.033885019	1
BP	GO:0072110	1/71	11/18670	0.0410562	0.075638154	0.033885019	1
BP	GO:0090193	1/71	11/18670	0.0410562	0.075638154	0.033885019	1
BP	GO:1900102	1/71	11/18670	0.0410562	0.075638154	0.033885019	1
BP	GO:1901096	1/71	11/18670	0.0410562	0.075638154	0.033885019	1
BP	GO:1901201	1/71	11/18670	0.0410562	0.075638154	0.033885019	1
BP	GO:1905383	1/71	11/18670	0.0410562	0.075638154	0.033885019	1
BP	GO:1905820	1/71	11/18670	0.0410562	0.075638154	0.033885019	1
BP	GO:0009144	4/71	342/18670	0.041265645	0.075982266	0.034039177	4
BP	GO:0048864	2/71	85/18670	0.04157462	0.076509165	0.034275222	2
BP	GO:0001942	2/71	86/18670	0.04246202	0.078013782	0.034949273	2
BP	GO:0030101	2/71	86/18670	0.04246202	0.078013782	0.034949273	2
BP	GO:0032760	2/71	86/18670	0.04246202	0.078013782	0.034949273	2
BP	GO:0097164	3/71	205/18670	0.043269296	0.079453423	0.035594215	3
BP	GO:0006112	2/71	87/18670	0.043356527	0.079570024	0.035646451	2
BP	GO:0009791	2/71	88/18670	0.044258074	0.080373316	0.036006317	2
BP	GO:0022404	2/71	88/18670	0.044258074	0.080373316	0.036006317	2
BP	GO:0022405	2/71	88/18670	0.044258074	0.080373316	0.036006317	2
BP	GO:0098773	2/71	88/18670	0.044258074	0.080373316	0.036006317	2
BP	GO:1903321	2/71	88/18670	0.044258074	0.080373316	0.036006317	2
BP	GO:1903557	2/71	88/18670	0.044258074	0.080373316	0.036006317	2
BP	GO:2000177	2/71	88/18670	0.044258074	0.080373316	0.036006317	2
BP	GO:0002024	1/71	12/18670	0.04470511	0.080373316	0.036006317	1
BP	GO:0002674	1/71	12/18670	0.04470511	0.080373316	0.036006317	1
BP	GO:0006527	1/71	12/18670	0.04470511	0.080373316	0.036006317	1
BP	GO:0009886	1/71	12/18670	0.04470511	0.080373316	0.036006317	1
BP	GO:0010649	1/71	12/18670	0.04470511	0.080373316	0.036006317	1
BP	GO:0010960	1/71	12/18670	0.04470511	0.080373316	0.036006317	1
BP	GO:0031282	1/71	12/18670	0.04470511	0.080373316	0.036006317	1
BP	GO:0032429	1/71	12/18670	0.04470511	0.080373316	0.036006317	1
BP	GO:0033197	1/71	12/18670	0.04470511	0.080373316	0.036006317	1
BP	GO:0034086	1/71	12/18670	0.04470511	0.080373316	0.036006317	1

BP	GO:0034088	1/71	12/18670	0.04470511	0.080373316	0.036006317	1
BP	GO:0035437	1/71	12/18670	0.04470511	0.080373316	0.036006317	1
BP	GO:0042118	1/71	12/18670	0.04470511	0.080373316	0.036006317	1
BP	GO:0042659	1/71	12/18670	0.04470511	0.080373316	0.036006317	1
BP	GO:0043922	1/71	12/18670	0.04470511	0.080373316	0.036006317	1
BP	GO:0044650	1/71	12/18670	0.04470511	0.080373316	0.036006317	1
BP	GO:0051095	1/71	12/18670	0.04470511	0.080373316	0.036006317	1
BP	GO:0051132	1/71	12/18670	0.04470511	0.080373316	0.036006317	1
BP	GO:0051873	1/71	12/18670	0.04470511	0.080373316	0.036006317	1
BP	GO:0060742	1/71	12/18670	0.04470511	0.080373316	0.036006317	1
BP	GO:0071472	1/71	12/18670	0.04470511	0.080373316	0.036006317	1
BP	GO:0071639	1/71	12/18670	0.04470511	0.080373316	0.036006317	1
BP	GO:0072520	1/71	12/18670	0.04470511	0.080373316	0.036006317	1
BP	GO:0097201	1/71	12/18670	0.04470511	0.080373316	0.036006317	1
BP	GO:0099550	1/71	12/18670	0.04470511	0.080373316	0.036006317	1
BP	GO:0099640	1/71	12/18670	0.04470511	0.080373316	0.036006317	1
BP	GO:1902337	1/71	12/18670	0.04470511	0.080373316	0.036006317	1
BP	GO:1902510	1/71	12/18670	0.04470511	0.080373316	0.036006317	1
BP	GO:1905461	1/71	12/18670	0.04470511	0.080373316	0.036006317	1
BP	GO:2001053	1/71	12/18670	0.04470511	0.080373316	0.036006317	1
BP	GO:2001223	1/71	12/18670	0.04470511	0.080373316	0.036006317	1
BP	GO:0032410	2/71	89/18670	0.045166599	0.081159489	0.036358513	2
BP	GO:0009161	4/71	354/18670	0.045840705	0.082326665	0.036881395	4
BP	GO:0050707	3/71	210/18670	0.04591726	0.082420006	0.036923211	3
BP	GO:0001656	2/71	90/18670	0.046082035	0.082494962	0.03695679	2
BP	GO:0014033	2/71	90/18670	0.046082035	0.082494962	0.03695679	2
BP	GO:0030641	2/71	90/18670	0.046082035	0.082494962	0.03695679	2
BP	GO:0031058	2/71	90/18670	0.046082035	0.082494962	0.03695679	2
BP	GO:0051304	2/71	90/18670	0.046082035	0.082494962	0.03695679	2
BP	GO:1904063	2/71	91/18670	0.04700432	0.084101112	0.037676327	2
BP	GO:0019319	2/71	92/18670	0.047933388	0.084507366	0.037858324	2
BP	GO:0006936	4/71	360/18670	0.048231009	0.084507366	0.037858324	4
BP	GO:0001765	1/71	13/18670	0.04834033	0.084507366	0.037858324	1
BP	GO:0002093	1/71	13/18670	0.04834033	0.084507366	0.037858324	1
BP	GO:0006596	1/71	13/18670	0.04834033	0.084507366	0.037858324	1
BP	GO:0007171	1/71	13/18670	0.04834033	0.084507366	0.037858324	1
BP	GO:0007494	1/71	13/18670	0.04834033	0.084507366	0.037858324	1
BP	GO:0009635	1/71	13/18670	0.04834033	0.084507366	0.037858324	1
BP	GO:0010763	1/71	13/18670	0.04834033	0.084507366	0.037858324	1
BP	GO:0010838	1/71	13/18670	0.04834033	0.084507366	0.037858324	1
BP	GO:0014745	1/71	13/18670	0.04834033	0.084507366	0.037858324	1
BP	GO:0014842	1/71	13/18670	0.04834033	0.084507366	0.037858324	1
BP	GO:0019896	1/71	13/18670	0.04834033	0.084507366	0.037858324	1
BP	GO:0032096	1/71	13/18670	0.04834033	0.084507366	0.037858324	1
BP	GO:0032099	1/71	13/18670	0.04834033	0.084507366	0.037858324	1
BP	GO:0032725	1/71	13/18670	0.04834033	0.084507366	0.037858324	1
BP	GO:0033004	1/71	13/18670	0.04834033	0.084507366	0.037858324	1
BP	GO:0033127	1/71	13/18670	0.04834033	0.084507366	0.037858324	1
BP	GO:0035641	1/71	13/18670	0.04834033	0.084507366	0.037858324	1
BP	GO:0042415	1/71	13/18670	0.04834033	0.084507366	0.037858324	1
BP	GO:0042532	1/71	13/18670	0.04834033	0.084507366	0.037858324	1
BP	GO:0042538	1/71	13/18670	0.04834033	0.084507366	0.037858324	1
BP	GO:0043301	1/71	13/18670	0.04834033	0.084507366	0.037858324	1
BP	GO:0045820	1/71	13/18670	0.04834033	0.084507366	0.037858324	1
BP	GO:0047484	1/71	13/18670	0.04834033	0.084507366	0.037858324	1
BP	GO:0048681	1/71	13/18670	0.04834033	0.084507366	0.037858324	1
BP	GO:0050722	1/71	13/18670	0.04834033	0.084507366	0.037858324	1
BP	GO:0050872	1/71	13/18670	0.04834033	0.084507366	0.037858324	1
BP	GO:0051001	1/71	13/18670	0.04834033	0.084507366	0.037858324	1
BP	GO:0060100	1/71	13/18670	0.04834033	0.084507366	0.037858324	1
BP	GO:0060601	1/71	13/18670	0.04834033	0.084507366	0.037858324	1
BP	GO:0060712	1/71	13/18670	0.04834033	0.084507366	0.037858324	1
BP	GO:0061042	1/71	13/18670	0.04834033	0.084507366	0.037858324	1

BP	GO:0061052	1/71	13/18670	0.04834033	0.084507366	0.037858324	1
BP	GO:0061430	1/71	13/18670	0.04834033	0.084507366	0.037858324	1
BP	GO:0071287	1/71	13/18670	0.04834033	0.084507366	0.037858324	1
BP	GO:0072044	1/71	13/18670	0.04834033	0.084507366	0.037858324	1
BP	GO:0097152	1/71	13/18670	0.04834033	0.084507366	0.037858324	1
BP	GO:0098903	1/71	13/18670	0.04834033	0.084507366	0.037858324	1
BP	GO:1900103	1/71	13/18670	0.04834033	0.084507366	0.037858324	1
BP	GO:1901550	1/71	13/18670	0.04834033	0.084507366	0.037858324	1
BP	GO:1903140	1/71	13/18670	0.04834033	0.084507366	0.037858324	1
BP	GO:1903960	1/71	13/18670	0.04834033	0.084507366	0.037858324	1
BP	GO:1905155	1/71	13/18670	0.04834033	0.084507366	0.037858324	1
BP	GO:0002460	4/71	361/18670	0.048636047	0.084950037	0.038056635	4
BP	GO:0019935	3/71	215/18670	0.04864422	0.084950037	0.038056635	3
BP	GO:0006476	2/71	93/18670	0.048869178	0.085254087	0.038192846	2
BP	GO:0032092	2/71	93/18670	0.048869178	0.085254087	0.038192846	2
BP	GO:0009141	4/71	362/18670	0.049042984	0.085512805	0.038308749	4
BP	GO:0002699	3/71	216/18670	0.049199023	0.085740293	0.038410661	3
BP	GO:0060348	3/71	217/18670	0.049756947	0.086667556	0.038826064	3
BP	GO:0051289	2/71	94/18670	0.049811626	0.086717748	0.038848549	2
CC	GO:0045121	12/71	315/19717	1.24E-09	1.39E-07	1.07E-07	12
CC	GO:0098857	12/71	316/19717	1.28E-09	1.39E-07	1.07E-07	12
CC	GO:0098589	12/71	328/19717	1.95E-09	1.41E-07	1.08E-07	12
CC	GO:0090575	9/71	163/19717	6.94E-09	3.77E-07	2.89E-07	9
CC	GO:0044798	9/71	201/19717	4.29E-08	1.86E-06	1.43E-06	9
CC	GO:0005667	10/71	365/19717	7.08E-07	2.56E-05	1.96E-05	10
CC	GO:0044853	6/71	109/19717	2.66E-06	8.24E-05	6.31E-05	6
CC	GO:0000790	9/71	377/19717	8.13E-06	0.000220522	0.000169015	9
CC	GO:0005901	5/71	80/19717	1.02E-05	0.000246647	0.000189038	5
CC	GO:0045177	8/71	384/19717	7.02E-05	0.001523222	0.001167446	8
CC	GO:0009897	8/71	393/19717	8.25E-05	0.001627235	0.001247165	8
CC	GO:0120111	4/71	84/19717	0.000239666	0.004125275	0.003161743	4
CC	GO:0009925	3/71	34/19717	0.000247136	0.004125275	0.003161743	3
CC	GO:0005925	7/71	405/19717	0.000628868	0.00882527	0.006763971	7
CC	GO:0062023	7/71	406/19717	0.000638087	0.00882527	0.006763971	7
CC	GO:0005924	7/71	408/19717	0.000656849	0.00882527	0.006763971	7
CC	GO:0030055	7/71	412/19717	0.000695688	0.00882527	0.006763971	7
CC	GO:0043209	3/71	49/19717	0.00073205	0.00882527	0.006763971	3
CC	GO:0030139	6/71	303/19717	0.000776667	0.008870356	0.006798527	6
CC	GO:0045178	3/71	51/19717	0.000823201	0.008885141	0.006809858	3
CC	GO:0005788	6/71	309/19717	0.000859852	0.008885141	0.006809858	6
CC	GO:0032838	5/71	208/19717	0.000921699	0.009091306	0.00696787	5
CC	GO:0016324	6/71	318/19717	0.000997353	0.009409807	0.007211979	6
CC	GO:1904115	3/71	57/19717	0.001138916	0.010297703	0.007892491	3
CC	GO:0016327	2/71	18/19717	0.001884461	0.016357126	0.012536628	2
CC	GO:0046930	2/71	23/19717	0.003080117	0.025707132	0.019702774	2
CC	GO:0005741	4/71	178/19717	0.003897899	0.031327562	0.024010452	4
CC	GO:0032839	2/71	32/19717	0.005913662	0.044760773	0.034306098	2
CC	GO:0031968	4/71	201/19717	0.005981854	0.044760773	0.034306098	4
CC	GO:0019867	4/71	203/19717	0.006192095	0.044789486	0.034328104	4
CC	GO:0031983	5/71	339/19717	0.007523074	0.052661515	0.040361482	5
CC	GO:0016323	4/71	217/19717	0.007802794	0.052912694	0.040553993	4
CC	GO:0005819	5/71	347/19717	0.00827541	0.054417092	0.041707012	5
CC	GO:0005769	5/71	350/19717	0.008570567	0.054700383	0.041924135	5
CC	GO:0043025	6/71	497/19717	0.008952508	0.055505552	0.042541243	6
CC	GO:0000307	2/71	42/19717	0.010030515	0.059641167	0.045710911	2
CC	GO:1904813	3/71	124/19717	0.010169231	0.059641167	0.045710911	3
MF	GO:0031625	11/71	290/17697	2.03E-08	5.79E-06	3.62E-06	11
MF	GO:0044389	11/71	308/17697	3.76E-08	5.79E-06	3.62E-06	11
MF	GO:0001085	8/71	155/17697	1.93E-07	1.55E-05	9.68E-06	8
MF	GO:0005126	10/71	286/17697	2.01E-07	1.55E-05	9.68E-06	10
MF	GO:0048018	12/71	482/17697	4.33E-07	2.67E-05	1.67E-05	12
MF	GO:0019902	8/71	185/17697	7.43E-07	3.50E-05	2.19E-05	8
MF	GO:0002020	7/71	128/17697	7.95E-07	3.50E-05	2.19E-05	7

MF	GO:0005178	7/71	132/17697	9.79E-07	3.77E-05	2.36E-05	7
MF	GO:0005125	8/71	220/17697	2.73E-06	9.33E-05	5.84E-05	8
MF	GO:0004252	7/71	160/17697	3.54E-06	0.000109141	6.83E-05	7
MF	GO:0051400	3/71	10/17697	7.28E-06	0.000176925	0.000110653	3
MF	GO:0070513	3/71	10/17697	7.28E-06	0.000176925	0.000110653	3
MF	GO:0004175	10/71	427/17697	7.47E-06	0.000176925	0.000110653	10
MF	GO:0008236	7/71	182/17697	8.28E-06	0.000182101	0.000113891	7
MF	GO:0017171	7/71	186/17697	9.54E-06	0.000195902	0.000122523	7
MF	GO:0019903	6/71	140/17697	2.07E-05	0.000397815	0.000248804	6
MF	GO:0097153	3/71	15/17697	2.72E-05	0.000492791	0.000308205	3
MF	GO:0004879	4/71	47/17697	3.72E-05	0.00060368	0.000377558	4
MF	GO:0098531	4/71	47/17697	3.72E-05	0.00060368	0.000377558	4
MF	GO:0051087	5/71	102/17697	5.55E-05	0.000854604	0.000534493	5
MF	GO:0003707	4/71	56/17697	7.46E-05	0.001094529	0.000684548	4
MF	GO:0019887	6/71	180/17697	8.43E-05	0.001180891	0.000738561	6
MF	GO:0019207	6/71	207/17697	0.000181235	0.002421368	0.00151439	6
MF	GO:0070491	4/71	71/17697	0.000188678	0.002421368	0.00151439	4
MF	GO:0020037	5/71	135/17697	0.000208546	0.002569281	0.001606898	5
MF	GO:0019838	5/71	137/17697	0.00022332	0.002645479	0.001654554	5
MF	GO:0030291	3/71	32/17697	0.000282347	0.003085698	0.001929879	3
MF	GO:0051721	3/71	32/17697	0.000282347	0.003085698	0.001929879	3
MF	GO:0046906	5/71	145/17697	0.000290536	0.003085698	0.001929879	5
MF	GO:0097718	3/71	33/17697	0.000309691	0.003179492	0.001988541	3
MF	GO:0005504	3/71	34/17697	0.000338686	0.00331252	0.00207174	3
MF	GO:0051219	4/71	83/17697	0.000344158	0.00331252	0.00207174	4
MF	GO:0001228	8/71	439/17697	0.000364611	0.003317004	0.002074544	8
MF	GO:0001103	3/71	35/17697	0.000369375	0.003317004	0.002074544	3
MF	GO:0033613	4/71	85/17697	0.000376932	0.003317004	0.002074544	4
MF	GO:0016705	5/71	159/17697	0.000443848	0.003797366	0.002374976	5
MF	GO:0016248	3/71	38/17697	0.000472012	0.003929178	0.002457415	3
MF	GO:0032813	3/71	46/17697	0.000830075	0.00656557	0.004106286	3
MF	GO:0050839	8/71	499/17697	0.000846245	0.00656557	0.004106286	8
MF	GO:0045236	2/71	11/17697	0.000852671	0.00656557	0.004106286	2
MF	GO:0008009	3/71	49/17697	0.000998835	0.007384713	0.0046186	3
MF	GO:0004861	2/71	12/17697	0.001020553	0.007384713	0.0046186	2
MF	GO:0042826	4/71	111/17697	0.001030983	0.007384713	0.0046186	4
MF	GO:0015459	3/71	52/17697	0.001187868	0.008227779	0.005145877	3
MF	GO:0016653	2/71	13/17697	0.001202981	0.008227779	0.005145877	2
MF	GO:0001102	3/71	53/17697	0.001255538	0.008227779	0.005145877	3
MF	GO:0005080	3/71	53/17697	0.001255538	0.008227779	0.005145877	3
MF	GO:0031072	4/71	119/17697	0.001334495	0.008563007	0.005355537	4
MF	GO:0036041	2/71	14/17697	0.001399841	0.008799	0.005503134	2
MF	GO:0005507	3/71	59/17697	0.00171267	0.010343184	0.006468909	3
MF	GO:0097110	3/71	59/17697	0.00171267	0.010343184	0.006468909	3
MF	GO:0005149	2/71	16/17697	0.001836393	0.010877098	0.006802833	2
MF	GO:0004860	3/71	63/17697	0.002068286	0.011840072	0.007405103	3
MF	GO:0005123	2/71	17/17697	0.002075857	0.011840072	0.007405103	2
MF	GO:0033293	3/71	64/17697	0.002163807	0.01211732	0.007578502	3
MF	GO:0042379	3/71	66/17697	0.00236298	0.012996388	0.008128295	3
MF	GO:0019210	3/71	67/17697	0.002466683	0.013098936	0.008192431	3
MF	GO:0030374	3/71	67/17697	0.002466683	0.013098936	0.008192431	3
MF	GO:0016247	4/71	144/17697	0.002678373	0.013982015	0.008744733	4
MF	GO:0001091	2/71	20/17697	0.002877638	0.014771876	0.009238733	2
MF	GO:0001618	3/71	74/17697	0.003271773	0.016253325	0.010165272	3
MF	GO:0104005	3/71	74/17697	0.003271773	0.016253325	0.010165272	3
MF	GO:0044183	2/71	23/17697	0.003802158	0.018297884	0.011443995	2
MF	GO:0070412	2/71	23/17697	0.003802158	0.018297884	0.011443995	2
MF	GO:0030295	3/71	80/17697	0.004076348	0.019022958	0.011897475	3
MF	GO:0046332	3/71	80/17697	0.004076348	0.019022958	0.011897475	3
MF	GO:0016209	3/71	86/17697	0.004991452	0.022608343	0.014139873	3
MF	GO:0019209	3/71	86/17697	0.004991452	0.022608343	0.014139873	3
MF	GO:0035035	2/71	29/17697	0.006007566	0.02681638	0.016771694	2
MF	GO:0043325	2/71	30/17697	0.006420074	0.028248326	0.017667272	2

MF	GO:0051427	4/71	185/17697	0.006515069	0.028262554	0.01767617	4
MF	GO:0005164	2/71	31/17697	0.00684514	0.029281989	0.018313753	2
MF	GO:0016712	2/71	32/17697	0.00728266	0.030683892	0.019190541	2
MF	GO:0004497	3/71	99/17697	0.007372104	0.030683892	0.019190541	3
MF	GO:0031406	4/71	193/17697	0.00754547	0.030986729	0.019379943	4
MF	GO:0004222	3/71	103/17697	0.008218243	0.033305512	0.020830173	3
MF	GO:0003713	5/71	319/17697	0.009105038	0.035693702	0.022323813	5
MF	GO:0016651	3/71	107/17697	0.009119357	0.035693702	0.022323813	3
MF	GO:0019825	2/71	36/17697	0.009155203	0.035693702	0.022323813	2
MF	GO:0043177	4/71	205/17697	0.009284081	0.03574371	0.022355089	4
MF	GO:0008200	2/71	37/17697	0.009653443	0.03670692	0.022957506	2
MF	GO:0008395	2/71	38/17697	0.010163523	0.038175183	0.023875798	2
MF	GO:0016709	2/71	39/17697	0.010685341	0.039651628	0.024799207	2
MF	GO:0009055	3/71	114/17697	0.010830747	0.03971274	0.024837428	3
MF	GO:0001784	2/71	40/17697	0.011218799	0.040651649	0.025424647	2
MF	GO:0004197	3/71	116/17697	0.011351532	0.040654323	0.025426319	3
MF	GO:0051879	2/71	41/17697	0.011763797	0.041603053	0.026019681	2
MF	GO:0099106	3/71	118/17697	0.011886587	0.041603053	0.026019681	3
MF	GO:0030544	2/71	42/17697	0.012320237	0.042636327	0.026665919	2
MF	GO:0004712	2/71	43/17697	0.012888021	0.044105673	0.027584888	2
MF	GO:0001046	2/71	45/17697	0.014057233	0.047578326	0.02975678	2
MF	GO:0001227	4/71	242/17697	0.016224775	0.054317724	0.033971782	4
MF	GO:0016538	2/71	49/17697	0.016527541	0.054678282	0.034197285	2
MF	GO:0070851	3/71	134/17697	0.016687528	0.054678282	0.034197285	3
MF	GO:0070888	2/71	50/17697	0.01717204	0.055526364	0.034727699	2
MF	GO:0004857	5/71	375/17697	0.017306919	0.055526364	0.034727699	5
MF	GO:0045309	2/71	51/17697	0.017827121	0.056605705	0.035402748	2
MF	GO:0001047	2/71	55/17697	0.020551416	0.064590165	0.040396446	2
MF	GO:0043621	2/71	56/17697	0.021258025	0.066136078	0.041363303	2
MF	GO:0035257	3/71	152/17697	0.023211867	0.07149255	0.044713385	3
MF	GO:0004714	2/71	62/17697	0.025705269	0.078388346	0.049026204	2

KEGG analysis

ID	GeneRatio	BgRatio	pvalue	p.adjust	qvalue	Count
hsa05418	22/69	139/8111	7.62E-23	1.56E-20	5.95E-21	22
hsa05417	25/69	215/8111	1.41E-22	1.56E-20	5.95E-21	25
hsa04933	18/69	100/8111	9.12E-20	6.72E-18	2.56E-18	18
hsa01524	13/69	73/8111	2.16E-14	1.18E-12	4.50E-13	13
hsa04657	14/69	94/8111	2.67E-14	1.18E-12	4.50E-13	14
hsa05161	16/69	162/8111	2.33E-13	8.60E-12	3.28E-12	16
hsa04668	14/69	112/8111	3.28E-13	1.04E-11	3.95E-12	14
hsa05219	10/69	41/8111	9.19E-13	2.46E-11	9.36E-12	10
hsa05215	13/69	97/8111	1.00E-12	2.46E-11	9.36E-12	13
hsa01522	13/69	98/8111	1.15E-12	2.53E-11	9.65E-12	13
hsa05160	15/69	157/8111	2.36E-12	4.74E-11	1.80E-11	15
hsa05167	16/69	194/8111	3.89E-12	7.16E-11	2.73E-11	16
hsa05210	12/69	86/8111	4.95E-12	8.41E-11	3.21E-11	12
hsa05207	16/69	212/8111	1.52E-11	2.40E-10	9.13E-11	16
hsa05163	16/69	225/8111	3.75E-11	5.53E-10	2.11E-10	16
hsa05222	11/69	92/8111	2.31E-10	3.19E-09	1.22E-09	11
hsa04115	10/69	73/8111	4.11E-10	5.35E-09	2.04E-09	10
hsa05212	10/69	76/8111	6.19E-10	7.60E-09	2.90E-09	10
hsa05225	13/69	168/8111	1.15E-09	1.31E-08	4.98E-09	13
hsa04210	12/69	136/8111	1.18E-09	1.31E-08	4.98E-09	12
hsa04066	11/69	109/8111	1.47E-09	1.47E-08	5.61E-09	11
hsa05162	12/69	139/8111	1.52E-09	1.47E-08	5.61E-09	12
hsa05164	13/69	172/8111	1.53E-09	1.47E-08	5.61E-09	13
hsa04932	12/69	155/8111	5.34E-09	4.91E-08	1.87E-08	12
hsa04215	7/69	32/8111	6.72E-09	5.94E-08	2.26E-08	7
hsa05223	9/69	72/8111	7.59E-09	6.45E-08	2.46E-08	9
hsa05169	13/69	202/8111	1.09E-08	8.95E-08	3.41E-08	13
hsa05142	10/69	102/8111	1.16E-08	9.17E-08	3.50E-08	10

hsa05208	13/69	223/8111	3.58E-08	2.73E-07	1.04E-07	13
hsa05323	9/69	93/8111	7.45E-08	5.49E-07	2.09E-07	9
hsa04926	10/69	129/8111	1.12E-07	7.99E-07	3.04E-07	10
hsa05205	12/69	205/8111	1.23E-07	8.46E-07	3.22E-07	12
hsa05218	8/69	72/8111	1.41E-07	9.42E-07	3.59E-07	8
hsa05144	7/69	50/8111	1.77E-07	1.15E-06	4.37E-07	7
hsa05214	8/69	75/8111	1.94E-07	1.21E-06	4.62E-07	8
hsa04620	9/69	104/8111	1.98E-07	1.21E-06	4.62E-07	9
hsa04915	10/69	138/8111	2.12E-07	1.22E-06	4.66E-07	10
hsa05133	8/69	76/8111	2.16E-07	1.22E-06	4.66E-07	8
hsa05220	8/69	76/8111	2.16E-07	1.22E-06	4.66E-07	8
hsa05140	8/69	77/8111	2.39E-07	1.32E-06	5.03E-07	8
hsa01521	8/69	79/8111	2.92E-07	1.58E-06	6.01E-07	8
hsa05145	9/69	112/8111	3.75E-07	1.97E-06	7.51E-07	9
hsa05224	10/69	147/8111	3.83E-07	1.97E-06	7.51E-07	10
hsa05213	7/69	58/8111	5.04E-07	2.53E-06	9.64E-07	7
hsa04218	10/69	156/8111	6.66E-07	3.27E-06	1.25E-06	10
hsa05130	11/69	197/8111	6.99E-07	3.36E-06	1.28E-06	11
hsa04010	13/69	294/8111	8.91E-07	4.19E-06	1.60E-06	13
hsa04380	9/69	128/8111	1.17E-06	5.37E-06	2.05E-06	9
hsa04151	14/69	354/8111	1.22E-06	5.51E-06	2.10E-06	14
hsa05166	11/69	219/8111	1.99E-06	8.80E-06	3.35E-06	11
hsa04064	8/69	104/8111	2.44E-06	1.05E-05	4.00E-06	8
hsa05152	10/69	180/8111	2.47E-06	1.05E-05	4.00E-06	10
hsa04659	8/69	108/8111	3.25E-06	1.35E-05	5.16E-06	8
hsa05165	13/69	331/8111	3.36E-06	1.38E-05	5.24E-06	13
hsa05171	11/69	232/8111	3.49E-06	1.40E-05	5.35E-06	11
hsa05226	9/69	149/8111	4.15E-06	1.64E-05	6.23E-06	9
hsa05202	10/69	192/8111	4.42E-06	1.71E-05	6.52E-06	10
hsa05132	11/69	249/8111	6.90E-06	2.63E-05	1.00E-05	11
hsa05235	7/69	89/8111	9.38E-06	3.51E-05	1.34E-05	7
hsa04370	6/69	59/8111	9.60E-06	3.54E-05	1.35E-05	6
hsa05206	12/69	310/8111	9.77E-06	3.54E-05	1.35E-05	12
hsa05416	6/69	60/8111	1.06E-05	3.73E-05	1.42E-05	6
hsa05170	10/69	212/8111	1.06E-05	3.73E-05	1.42E-05	10
hsa05216	5/69	37/8111	1.36E-05	4.69E-05	1.79E-05	5
hsa04621	9/69	185/8111	2.40E-05	8.15E-05	3.10E-05	9
hsa04625	7/69	104/8111	2.61E-05	8.75E-05	3.34E-05	7
hsa05131	10/69	247/8111	4.00E-05	0.000131931	5.03E-05	10
hsa04012	6/69	85/8111	7.84E-05	0.00025476	9.71E-05	6
hsa05020	10/69	273/8111	9.30E-05	0.000297874	0.000113503	10
hsa05134	5/69	57/8111	0.000114349	0.000361015	0.000137562	5
hsa05135	7/69	137/8111	0.000151663	0.000472079	0.000179882	7
hsa05231	6/69	98/8111	0.000173014	0.000531056	0.000202355	6
hsa05146	6/69	102/8111	0.000215509	0.000652433	0.000248605	6
hsa04660	6/69	104/8111	0.000239639	0.000715678	0.000272704	6
hsa05143	4/69	37/8111	0.000256373	0.000755444	0.000287857	4
hsa04928	6/69	106/8111	0.000265849	0.000773062	0.00029457	6
hsa05211	5/69	69/8111	0.000283667	0.00081416	0.00031023	5
hsa05415	8/69	203/8111	0.000302604	0.000849002	0.000323506	8
hsa05230	5/69	70/8111	0.000303489	0.000849002	0.000323506	5
hsa04921	7/69	154/8111	0.000312273	0.000853555	0.000325241	7
hsa05203	8/69	204/8111	0.000312842	0.000853555	0.000325241	8
hsa04137	5/69	72/8111	0.000346272	0.000933245	0.000355607	5
hsa04217	7/69	159/8111	0.000379334	0.001010035	0.000384867	7
hsa04670	6/69	114/8111	0.00039407	0.00103678	0.000395058	6
hsa04630	7/69	162/8111	0.000424818	0.001104527	0.000420872	7
hsa04071	6/69	119/8111	0.000495978	0.001259898	0.000480075	6
hsa04722	6/69	119/8111	0.000495978	0.001259898	0.000480075	6
hsa05022	12/69	476/8111	0.000596814	0.001498818	0.000571114	12
hsa04610	5/69	85/8111	0.000745485	0.001851147	0.000705367	5
hsa04371	6/69	138/8111	0.00108236	0.002657796	0.001012735	6
hsa04510	7/69	201/8111	0.001519365	0.003689888	0.001406006	7

hsa05321	4/69	65/8111	0.002198096	0.005280208	0.002011987	4
hsa05204	4/69	69/8111	0.002736193	0.006502137	0.002477594	4
hsa04917	4/69	70/8111	0.00288363	0.006708234	0.002556126	4
hsa05120	4/69	70/8111	0.00288363	0.006708234	0.002556126	4
hsa05168	11/69	498/8111	0.002969283	0.006835538	0.002604635	11
hsa04060	8/69	295/8111	0.003385651	0.007713701	0.002939252	8
hsa04919	5/69	121/8111	0.003570712	0.008052321	0.003068281	5
hsa03320	4/69	76/8111	0.003884023	0.008670394	0.003303794	4
hsa04611	5/69	124/8111	0.003965804	0.008764427	0.003339624	5
hsa04068	5/69	131/8111	0.005008253	0.010958652	0.004175719	5
hsa04662	4/69	82/8111	0.005097209	0.011043953	0.004208222	4
hsa05332	3/69	42/8111	0.005336094	0.011449288	0.004362672	3
hsa04062	6/69	192/8111	0.00566264	0.012000772	0.004572811	6
hsa04940	3/69	43/8111	0.005701724	0.012000772	0.004572811	3
hsa04211	4/69	89/8111	0.006806775	0.014078954	0.005364688	4
hsa04140	5/69	141/8111	0.006816507	0.014078954	0.005364688	5
hsa04658	4/69	92/8111	0.007643018	0.01563988	0.005959468	4
hsa04912	4/69	93/8111	0.007936148	0.016090722	0.006131259	4
hsa04072	5/69	148/8111	0.008327181	0.016730063	0.006374875	5
hsa04913	3/69	51/8111	0.009167864	0.018253134	0.006955231	3
hsa04934	5/69	155/8111	0.010056678	0.019843981	0.007561412	5
hsa04061	4/69	100/8111	0.010196901	0.019942611	0.007598994	4
hsa04024	6/69	219/8111	0.010535432	0.020423951	0.007782406	6
hsa05014	8/69	365/8111	0.011833003	0.022739944	0.008664899	8
hsa04923	3/69	57/8111	0.012424149	0.023670146	0.009019346	3
hsa05010	8/69	369/8111	0.012579527	0.023761328	0.00905409	8
hsa04931	4/69	108/8111	0.01324978	0.024815267	0.009455686	4
hsa04022	5/69	167/8111	0.013571468	0.025204155	0.009603869	5
hsa04014	6/69	232/8111	0.01372053	0.025268643	0.009628442	6
hsa05016	7/69	306/8111	0.014808347	0.027046651	0.01030594	7
hsa04725	4/69	113/8111	0.015425257	0.027942474	0.010647287	4
hsa05217	3/69	63/8111	0.0162647	0.029223567	0.011135439	3
hsa04929	3/69	64/8111	0.016962505	0.030231562	0.011519528	3
hsa04935	4/69	119/8111	0.018318544	0.032387187	0.012340914	4
hsa04152	4/69	120/8111	0.018831406	0.032775424	0.012488849	4
hsa05012	6/69	249/8111	0.018834746	0.032775424	0.012488849	6
hsa05221	3/69	67/8111	0.019155653	0.033073432	0.012602403	3
hsa04920	3/69	69/8111	0.02070112	0.035464709	0.013513583	3
hsa04110	4/69	124/8111	0.020971967	0.035652343	0.01358508	4
hsa04622	3/69	70/8111	0.021498918	0.036269167	0.013820116	3
hsa04650	4/69	131/8111	0.025066702	0.041967737	0.015991517	4
hsa00980	3/69	78/8111	0.028483313	0.04697621	0.017899961	3
hsa04612	3/69	78/8111	0.028483313	0.04697621	0.017899961	3
hsa04910	4/69	137/8111	0.028936666	0.047370394	0.018050162	4
hsa04514	4/69	149/8111	0.037694319	0.061253269	0.023340136	4
hsa04261	4/69	150/8111	0.03848599	0.062083239	0.02365639	4
hsa05330	2/69	38/8111	0.041189592	0.065963042	0.025134762	2
hsa04216	2/69	41/8111	0.047273171	0.075160941	0.028639558	2
hsa04666	3/69	97/8111	0.049304313	0.077381386	0.029485644	3
hsa00380	2/69	42/8111	0.049370025	0.077381386	0.029485644	2
