# Supplementary material for: Identification of Bioactive Compounds and Potential Mechanisms of Kuntai Capsule in the Treatment of Polycystic Ovary Syndrome by Integrating Network Pharmacology and Bioinformatics
Source: Oxid Med Cell Longev. 2022 Apr 28;2022:3145938. doi: 10.1155/2022/3145938 (PMC9073551; doi:10.1155/2022/3145938)
Supplement: Supplementary 9 — Supplement Table 9: the R language scripts used in the study. [file 3145938.f9.pdf]

GO enrichment analysis

```
install.packages("colorspace")
install.packages("stringi")
install.packages("ggplot2")
if (!requireNamespace("BiocManager", quietly = TRUE))
    install.packages("BiocManager")
BiocManager::install("DOSE")
BiocManager::install("clusterProfiler")
BiocManager::install("enrichplot")
library("clusterProfiler")
library("org.Hs.eg.db")
library("enrichplot")
library("ggplot2")
pvalueFilter=0.05
qvalueFilter=0.05
setwd("C:\\Users\\Administrator\\Desktop\\kuntai\\GO")
rt=read.table("id.txt",sep="\t",header=T,check.names=F)
rt=rt[is.na(rt[,"entrezID"])==F,]
gene=rt$entrezID
colorSel="qvalue"
if(qvalueFilter>0.05){
    colorSel="pvalue"
}
kk=enrichGO(gene = gene,OrgDb = org.Hs.eg.db, pvalueCutoff =1, qvalueCutoff = 1, ont="all",
readable =T)
GO=as.data.frame(kk)
GO=GO[(GO$pvalue<pvalueFilter & GO$qvalue<qvalueFilter),]
write.table(GO,file="GO.txt",sep="\t",quote=F,row.names = F)
showNum=10
if(nrow(GO)<30){
    showNum=nrow(GO)
}
pdf(file="barplot.pdf",width = 10,height =7)
bar=barplot(kk, drop = TRUE, showCategory =showNum,split="ONTOLOGY",color = colorSel)
+ facet_grid(ONTOLOGY~., scale='free')
print(bar)
dev.off()
```

KEGG enrichment analysis

```
install.packages("colorspace")
```

```
install.packages("stringi")
install.packages("ggplot2")

if (!requireNamespace("BiocManager", quietly = TRUE))
        install.packages("BiocManager")
BiocManager::install("DOSE")
BiocManager::install("clusterProfiler")
BiocManager::install("enrichplot")
BiocManager::install("pathview")
library("clusterProfiler")
library("org.Hs.eg.db")
library("enrichplot")
library("ggplot2")
library("pathview")
pvalueFilter=0.05
qvalueFilter=0.05
setwd("C:\\Users\\Administrator\\Desktop\\kuntai\\KEGG")
rt=read.table("id.txt",sep="\t",header=T,check.names=F)
rt=rt[is.na(rt[,"entrezID"])==F,]
colnames(rt)[1]="Gene"
gene=rt$entrezID
colorSel="qvalue"
if(qvalueFilter>0.05){
        colorSel="pvalue"
}
kk <- enrichKEGG(gene = gene, organism = "hsa", pvalueCutoff =1, qvalueCutoff =1)
KEGG=as.data.frame(kk)
KEGG$geneID=as.character(sapply(KEGG$geneID,function(x)paste(rt$Gene[match(strsplit(x,"/"
)[[1]],as.character(rt$entrezID))],collapse="/")))
KEGG=KEGG[(KEGG$pvalue<pvalueFilter & KEGG$qvalue<qvalueFilter),]
write.table(KEGG,file="KEGG.txt",sep="\t",quote=F,row.names = F)
showNum=30
if(nrow(KEGG)<showNum){
        showNum=nrow(KEGG)
}
pdf(file="bubble.pdf",width = 9,height = 7)
dotplot(kk, showCategory = showNum, orderBy = "GeneRatio",color = colorSel)
dev.off()
```
